# Borane-induced ring closure reaction of oligomethylene-linked bis-allenes†

**DOI:** 10.1039/c9sc03870a

**Published:** 2019-12-18

**Authors:** Xin Tao, Karel Škoch, Constantin G. Daniliuc, Gerald Kehr, Gerhard Erker

**Affiliations:** Organisch-Chemisches Institut, Westfälische Wilhelms-Universität Münster Corrensstraße 40 48149 Münster Germany erker@uni-muenster.de

## Abstract

The trimethylene-linked bis-allene **3a** reacts with Piers' borane [HB(C_6_F_5_)_2_] by a hydroboration/allylboration sequence to generate the cyclization product **5a**. Its pyridine adduct was isolated and characterized by X-ray diffraction. Compound **5a** undergoes a typical frustrated Lewis pair 1,2-P/B alkene addition reaction with PPh_3_ to give the heterobicyclic bridged olefinic zwitterionic product **9a**. The tetramethylene-linked bis-allene **3b** and its phenylene annulated analogue **3c** react with HB(C_6_F_5_)_2_ to give the analogous seven-membered ring products **5b,c** under mild conditions. The cyclization product **5a** undergoes a series of sequential allylboration reactions with two equivalents of allene followed by ring-closure to give the four-component coupling product **12a**. It undergoes FLP addition to an exo-methylene group upon treatment with PPh_3_. Compound **12a** is oxidatively converted to the boron-free alcohol.

## Abstract 2

The borane induced cyclization of the bis-allene followed by reaction with two molar equivalents of allene gives the functionalized decalin product.

## Graphical abstract

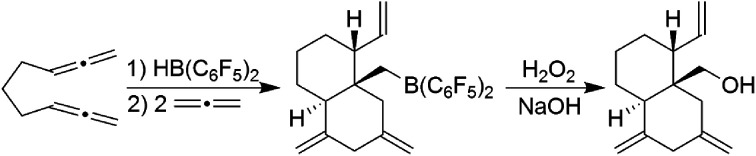

## Introduction

**Scheme 1:**
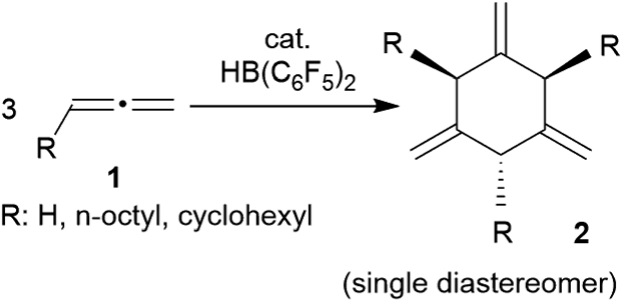
HB(C_6_F_5_)_2_-catalyzed cyclotrimerization of alkyl-substituted allenes.

Allenes are important building blocks in organic synthesis.^1^ They show interesting and useful stereochemical properties.^2^ We had recently shown that the borane [HB(C_6_F_5_)_2_]^3^ catalyses the cyclotrimerization of allene as well as a small series of mono-alkyl-substituted allenes **1** to selectively give the respective 1,3,5-trimethylene cyclohexanes **2** as single isomers under metal-free conditions (Scheme 1).^1,4,5^

**Chart 1:**
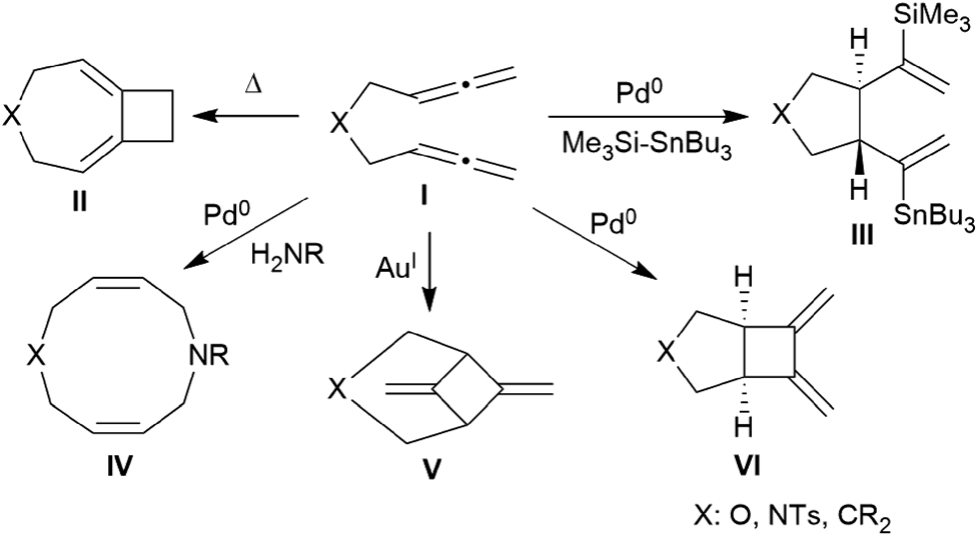
Examples of cyclization reactions of bis-allenes.

There is a rich cyclization chemistry of bis-allenes reported in the literature (see Chart 1). Systems **I** (mostly with X = NTs, less frequently CR_2_ or O) were reported to rearrange to **II** thermally induced.^6*a*^ They added R_3_Si-SnBu_3_/or -GeR_3_ reagents Pd(0) catalysed or radical induced to give the products **III**.^6*b*,*c*^ With H_2_NR nucleophiles cyclization to medium-sized rings (*e.g.***IV**) was reported.^6*d*^ The products **V** and **VI** of internal [2 + 2] cycloaddition were formed under the influence of Au(i)^6*e*^ or Pd(0)^6*a*^ catalysis, respectively. In some cases coupling between two bis-allenes occurred to give hetero-steroidal frameworks.^6*f*,*g*^ It should be noted that the vast majority of these reaction is metal catalysed.

This posed the question what the favoured reaction pathway would be if we treated *e.g.* oligomethylene-linked bis-allenes with HB(C_6_F_5_)_2_,^7^*i.e.* under metal-free conditions. We have now performed these reactions starting from two examples of that bis-allene family. It turned out that a different cyclization type prevailed under these conditions. The outcome of these reactions will be presented and discussed below.

## Results and discussion

### Borane-induced ring-closure reaction of the bis-allenes

**Scheme 2:**
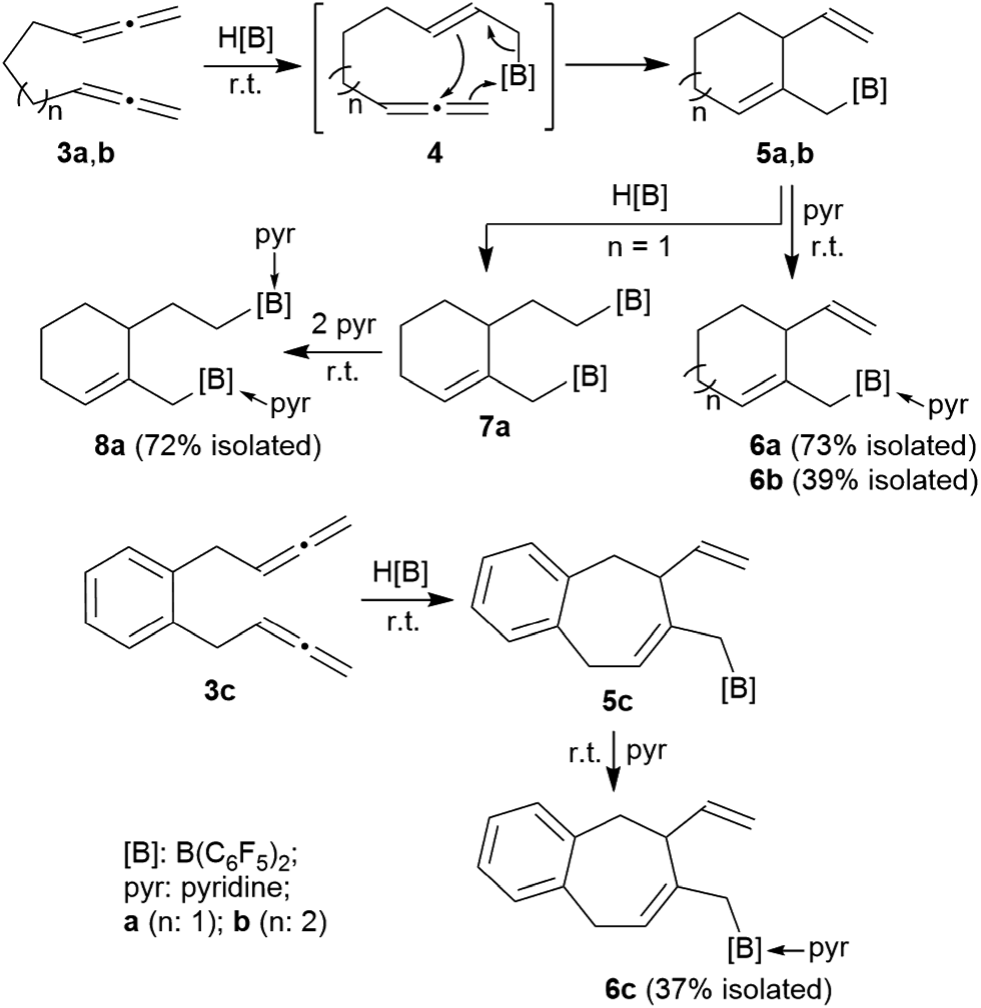
HB(C_6_F_5_)_2_-induced ring closure of the bis-allenes **3**.

The allenes **3a** and **3b** (Scheme 2) were prepared by a variant of the Crabbé reaction as described by Ma *et al.*^8^ Copper(i) induced treatment of 1,6-heptadiyne with *para*-formaldehyde as C_1_-building block and dicyclohexylamine gave the trimethylene-linked bis-allene **3a**^6*h*^ (40% isolated). The analogous reaction starting from 1,7-octadiyne gave the tetramethylene-linked bis-allene **3b**^6*i*^ (63% isolated, see the ESI† for details). The phenylene containing bis-allene **3c** was synthesised analogously.

**Fig. 1:**
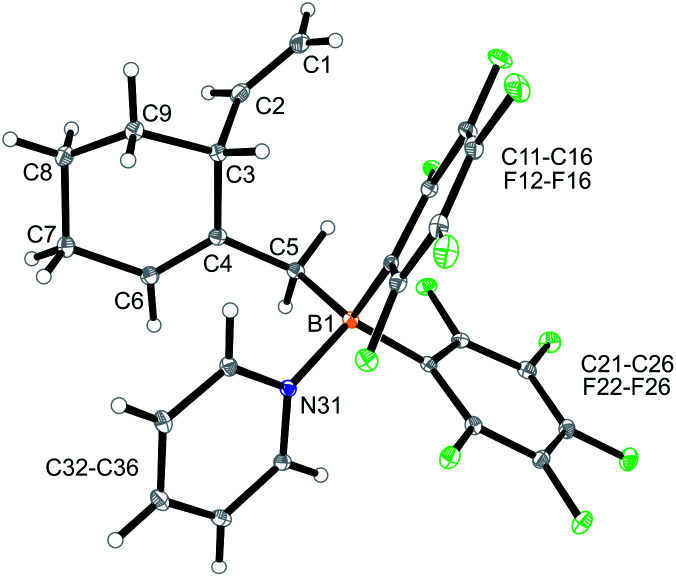
A view of the molecular structure of the borane pyridine adduct **6a**. Selected bond lengths (Å) and angles (°): B1–N31 1.613(3), B1–C5 1.651(3), C1–C2 1.322(4), C4–C6 1.337(3), B1–C5–C4 117.7(2).

We reacted compound **3a** with one molar equivalent of Piers' borane [HB(C_6_F_5_)_2_]. The rapid reaction (r.t., minutes, in CD_2_Cl_2_) gave the cyclized product **5a**. It was not isolated but characterized by spectroscopy [^1^H NMR: *δ* 5.55 (olefinic =CH–) (C6–H, see Fig. 1 for the unsystematic atom numbering scheme), *δ* 5.52, 5.03, (–CH=CH_2_ substituent); ^11^B NMR: *δ* 69.3; Δ*δ*^19^F_*m*,*p*_ = 13.1 ppm]. Compound **5a** was then generated *in situ* on a preparative scale and trapped by subsequent addition of pyridine. We isolated the pyridine adduct **6a** as a white crystalline solid in 73% yield. The X-ray crystal structure analysis of compound **6a** showed the newly formed cyclohexene core that has a vinyl group attached in the allylic position and it bears the –CH_2_B(C_6_F_5_)_2_(pyridine) substituent adjacent to it at the olefinic ring carbon atom C4 (Fig. 1). In solution (CD_2_Cl_2_) we monitored the typical ^1^H/^13^C NMR features of the vinyl group at carbon atom C3 and the central doubly substituted cyclohexene core. The ^1^H NMR features of the coordinated pyridine moiety show up at *δ* 8.67, 8.11 and 7.65 (^11^B: *δ* −0.6). Due to the chiral centre (ring carbon C3) the C_6_F_5_ groups at boron are diastereotopic and give rise to a 1 : 1 set of the respective *o*,*p*,*m*-C_6_F_5_^19^F NMR signals.

We assume that the reaction started with hydroboration of the terminal =CH_2_ unit of one allenyl group by the HB(C_6_F_5_)_2_ reagent. This resulted in the formation of the functionalized borane **4** which contained a reactive allylborane unit opposite to a reactive allene unit. This situation was set up for an internal allylboration reaction of the allene, which directly opened a pathway to the observed cyclization product **5a**. Addition of the pyridine Lewis base to the strongly Lewis acidic B(C_6_F_5_)_2_ group gave **6a** (Scheme 2).

**Fig. 2:**
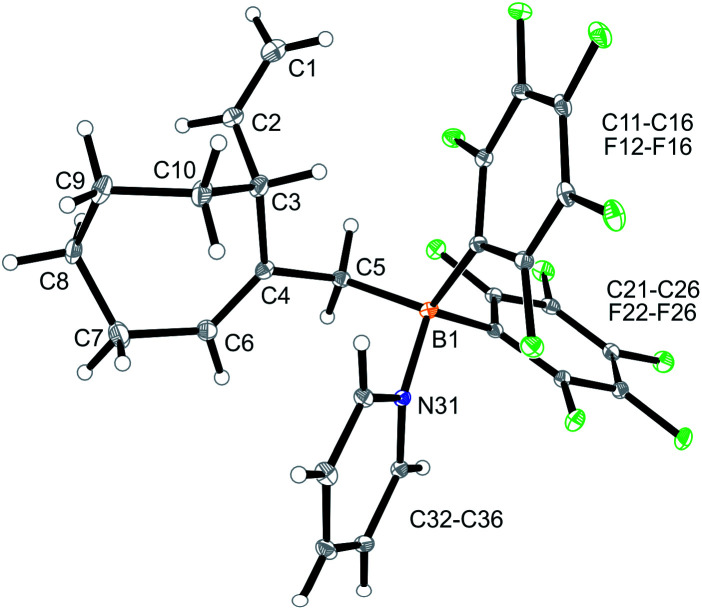
Molecular structure of compound **6b**. Selected bond lengths (Å) and angles (°): B1–N31 1.627(5), B1–C5 1.645(6), C1–C2 1.285(8), C4–C6 1.340(6), B1–C5–C4 119.9(3).

We reacted the tetramethylene-linked bis-allene **3b** with HB(C_6_F_5_)_2_ and found that the seven-membered cyclization product **5b** was formed analogously. Treatment of the *in situ* generated borane **5b** with pyridine gave the respective pyridine adduct **6b**, which we isolated crystalline in 39% yield. Compounds **5b** and **6b** were characterized by spectroscopy (see the ESI† for details); product **6b** was characterized by C, H, N elemental analysis and by an X-ray crystal structure analysis (Fig. 2). It shows the newly formed seven-membered ring in a typical cycloheptene boat-like conformation.^9^ The –CH_2_B(C_6_F_5_)_2_ moiety is attached at the sp^2^-carbon atom C4 (the boron atom bears a pair of C_6_F_5_ groups and the coordinated pyridine ligand in a pseudo-tetrahedral geometry). The vinyl substituent is bonded to carbon atom C3.

The reaction of the bis-allene **3a** responded to the stoichiometry of the HB(C_6_F_5_)_2_ reagent since the primary ring-closure product **5a** contained a reactive pendant vinyl group. Therefore, the reaction of **3a** with two molar equivalents of Piers' borane gave the bis-borane product **7a**. Its ^11^B NMR spectrum showed two signals (*δ* 73.3 and *δ* 69.4) that are attributed to the pair of Lewis acidic planar-tricoordinate boron centres. Consequently, we observed two sets of ^19^F NMR resonances of the pairs of the C_6_F_5_ substituents at the two B(C_6_F_5_)_2_ groups. The *in situ* generated bis-borane **7a** was then treated with two molar equivalents of pyridine to give the respective double pyridine adduct **8a** (isolated as a white solid in 72% yield). It was characterized by C, H, N elemental analysis, by X-ray diffraction (the structure is shown in the ESI†) and by spectroscopy. Due to the chiral centre (C3) the pair of C_6_F_5_ groups at each B(C_6_F_5_)_2_(pyridine) moiety are diastereotopic and, consequently, we observed four sets of *o*,*m*,*p*-C_6_F_5_^19^F NMR signals of compound **8a**. We observed two sets of pyridine ^1^H/^13^C NMR resonances and located the single olefinic ^1^H NMR signal (C6–H) at *δ* 4.35 (t, *J*_HH_ = 3.3 Hz, 1H).

We performed the reaction of the phenylene bridged bis-allene system **3c** in a similar way. The reaction of **3c** with one molar equiv. of HB(C_6_F_5_)_2_ was carried out in CD_2_Cl_2_ at r.t. and the almost instantaneously *in situ* generated product was characterized by NMR spectroscopy [^1^H: *δ* 5.48/5.13/5.08 (vinyl substituent), 5.72 (ring-CH=), ^11^B: *δ* 71.9, ^19^F: Δ*δ*^19^F_*m*,*p*_ = 13.2]. The latter heteroatom NMR signals are typical for the presence of strongly Lewis acidic tricoordinate boron with this substituent pattern. Compound **5c** was treated with pyridine and the respective pyridine/borane Lewis adduct **6c** was isolated in 37% yield after workup involving pentane extraction. The NMR spectra now show a ^11^B NMR resonance in the typical range of tetracoordinate boron (*δ* −0.5) and the C_6_F_5_ substituents at boron are diastereotopic (see the ESI† for further details).

### Subsequent FLP ring-closure reactions

The compounds **5** each contain a sterically encumbered, strongly Lewis acidic borane functionality and in its vicinity an accessible reactive vinyl group. We used this for carrying out a typical frustrated Lewis pair^10^ reaction, namely a 1,2-borane/phosphane addition to the C=C double bond.^11^

**Scheme 3:**
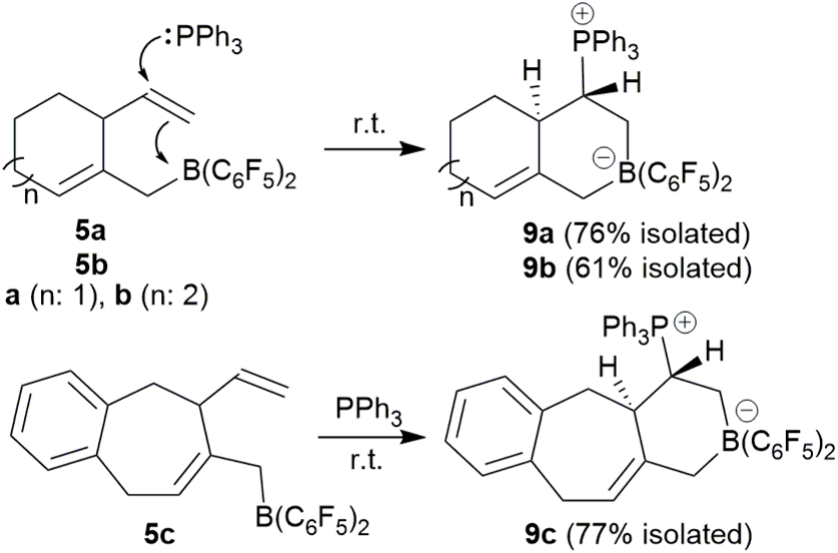
FLP reaction of the boranes **5** with PPh_3_.

**Fig. 3:**
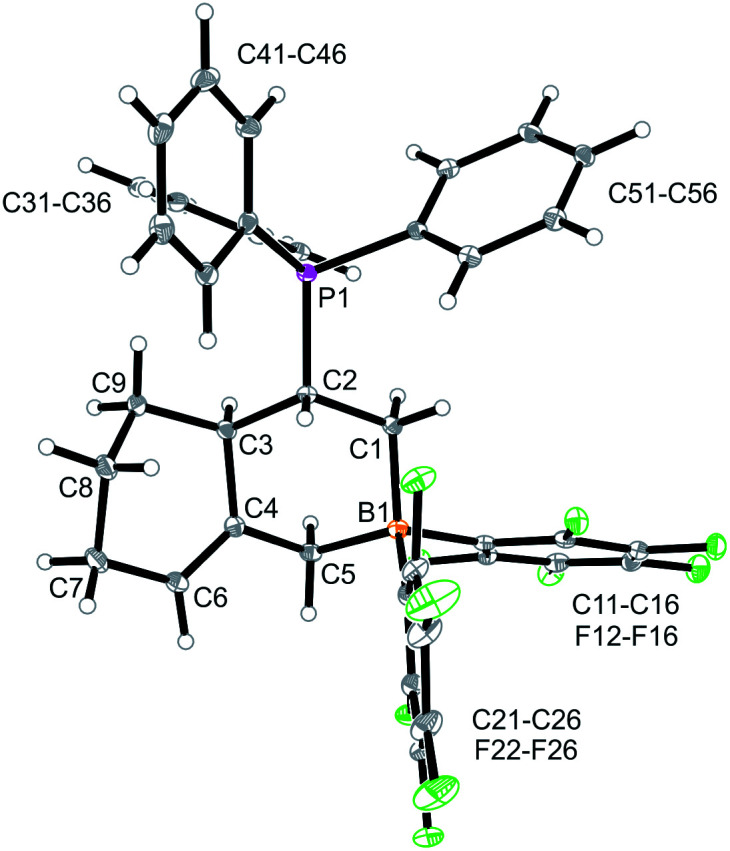
Molecular structure of the P/B FLP addition product **9a**. Selected bond lengths (Å) and angles (°): B1–C1 1.647(2), B1–C5 1.640(2), P1–C2 1.856(2), C1–C2 1.549(2), C4–C6 1.333(2), C1–B1–C5 105.7(1), B1–C1–C2 114.6(1), C1–C2–C3 112.4(1), C2–C3–C4 109.2(1), C3–C4–C5 114.9(1), C4–C5–B1 108.9(1), P1–C2–C1 109.3(1), P1–C2–C3 111.9(1), P1–C2–C3–C4 174.9(1), P1–C2–C1–B1 175.9(1).

We reacted the *in situ* generated cyclization product **5a** with triphenylphosphane at room temperature. The reaction was practically instantaneous under these typical conditions and we isolated the P/B addition product **9a** to the internal vinyl group as a white powder in 76% yield (Scheme 3). Single crystals of compound **9a** that were suited for the X-ray crystal structure analysis were obtained at room temperature from a solution in dichloromethane that was layered with pentane. It showed that 1,2-phosphane/borane addition to the vinyl substituent had occurred. The internal –B(C_6_F_5_)_2_ Lewis acid had been added to the =CH_2_ terminus of the alkene and the external PPh_3_ nucleophile to the –CH= carbon atom. The resulting zwitterionic heterobicyclo[4.4.0]decene type system features a bridgehead C=C double bond (C4–C6). There is a borate system inside the heterocyclic six-membered ring that was formed in the FLP addition reaction. Consequently, the –PPh_3_^+^ phosphonium substituent is found attached at the same ring at carbon atom C2. We have isolated a single diastereoisomer of **9a** from this reaction; it features the hydrogen atoms at carbon atoms C2 and C3 oriented *trans* to each other at the heterobicyclic framework (Fig. 3). In solution compound **9a** shows the NMR heteronuclear resonances at *δ* −12.3 (^11^B) and *δ* 27.9 (^31^P). A diastereotopic pair of C_6_F_5_ substituents is bonded at boron, giving rise to two separate sets of ^19^F NMR resonances. The ^1^H NMR [P]–CH– signal is found at *δ* 3.64 and the single olefinic =CH– signal at *δ* 5.17.

The seven-membered ring compounds **5b** and **5c** react analogously with PPh_3_. Compound **5b** was *in situ* generated and triphenylphosphane was added. We isolated the product **9b** in 61% yield by crystallization. It was characterized by C, H-elemental analysis, by spectroscopy and by X-ray diffraction. The molecular structure is similar to that of **9a**. It also contains a *trans*-relationship of the C3–H and C2–H hydrogen atoms of the heterobicyclic ring system. The hetero-NMR signals occur at *δ* −13.3 (^11^B) and *δ* 30.5 (^31^P). Compound **9c** was prepared analogously from **5c** (see Scheme 3). It was isolated as a white solid in 77% yield after workup and characterized by NMR spectroscopy and by an X-ray crystal structure analysis. For further details of the characterization of the compounds **9b** and **9c** including their depicted molecular structures see the ESI.†

### Subsequent cyclooligomerization reaction with allene

**Fig. 4:**
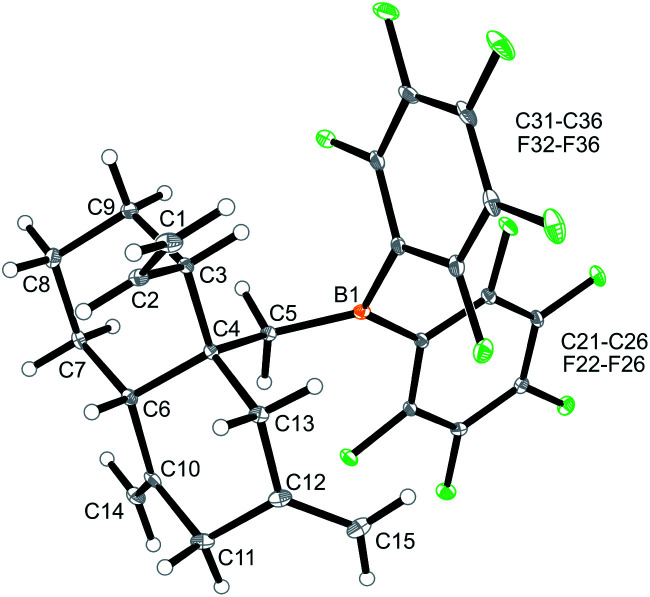
Molecular structure of compound **12a**. Selected bond lengths (Å) and angles (°): B1–C5 1.554(3), C1–C2 1.313(3), C4–C5 1.557(3), C4–C6 1.566(3), C10–C14 1.324(3), C12–C15 1.324(3), B1–C5–C4 123.2(2), C5–C4–C6 109.0(2).

**Scheme 4:**
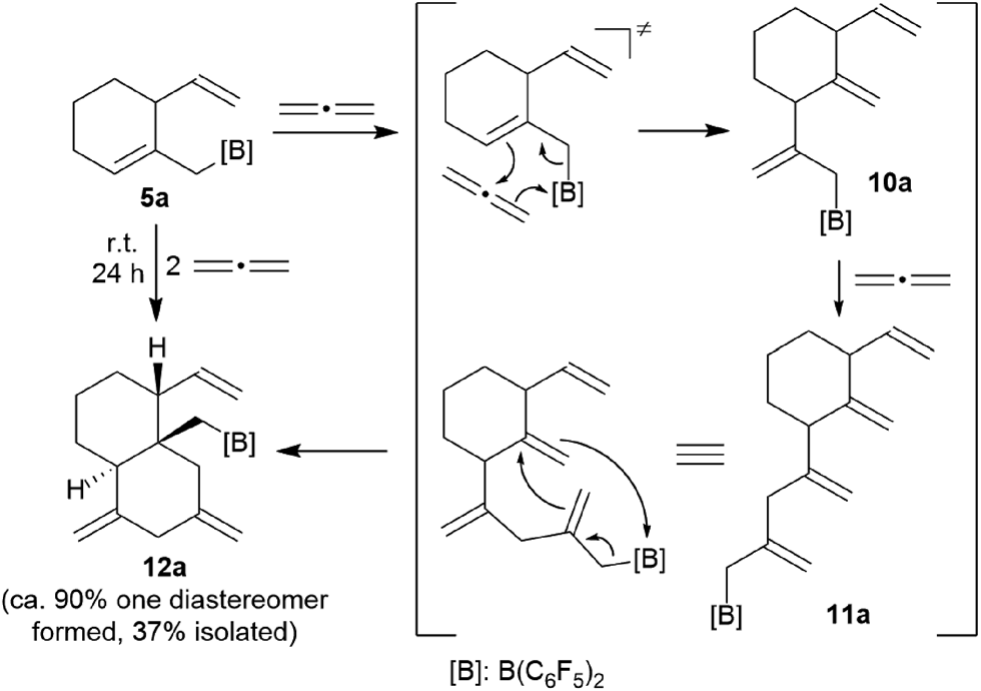
Formation of compound **12a** and allene by sequential allylboration reactions.

Internal allene allylboration^12^ represents the important step of the ring-closure reaction sequence starting from **3** to form the products **5**. The compounds **5** themselves each contain an allylborane functionality which might show the respective reactivity towards added allene reagents. Therefore, we exposed the cyclization product **5a** to an excess of the parent allene H_2_C=C=CH_2_. An NMR experiment revealed a close to complete conversion to the new product **12a** within 24 h at room temperature. We carried out this reaction on a preparative scale under analogous conditions. Workup involving crystallization from pentane at −35 °C (3 d) gave the crystalline product **12a**, which we isolated in 37% yield. Compound **12a** was characterized by C, H elemental analysis, by spectroscopy and by an X-ray crystal structure analysis. This showed (Fig. 4) that the endocyclic allylborane moiety of the starting material **5a** had reacted with two molar equivalents of H_2_C=C=CH_2_ to give the functionalized decalin derivative **12a**. Apparently, compound **5a** had undergone an allylboration reaction with allene to generate the intermediate **10a**. This itself represents an “elongated” allylborane system, that subsequently took up another allene equivalent to give **11a**. The intermediate **11a** could in principle have reacted with further allene, but instead its allylborane “found” the remaining exo-methylene group at the six-membered core with which it underwent a favoured intramolecular allylboration reaction^4^ to directly give the observed product **12a** (Scheme 4).

The X-ray crystal structure analysis of compound **12a** shows the newly formed *trans*-decalin framework that was formed by the consecutive C–C coupling between **5a** and two molar equivalents of allene. The ring carbon atom C3 bears the remaining vinyl substituent; carbon atoms C10 and C12 are both part of the pair of exo-methylene groups 1,3-positioned in the second ring. The –CH_2_B(C_6_F_5_)_2_ group is attached at the bridgehead carbon (C4 in Fig. 4).

In solution (CD_2_Cl_2_) compound **12a** shows the typical NMR features of the vinyl substituent. The pair of =CH_2_ exo-methylene groups shows a total of four ^1^H NMR resonances (*δ* 4.85/4.61 and *δ* 4.68/4.42). The –CH_2_[B] substituent shows the ^1^H NMR signals of a pair of diastereotopic hydrogen atoms (AB system at *δ* 2.30/2.15; ^13^C: *δ* 36.7). The corresponding ^11^B NMR feature is at *δ* 76.4, *i.e.* in a typical range of a strongly Lewis acidic tri-coordinated boron atom in this substituent situation.^13^ Consequently, we observed three ^19^F NMR signals of the pair of the C_6_F_5_ substituents at boron with a large Δ*δ*^19^F_*m*,*p*_ = 11.5 ppm chemical shift separation.

### Some typical reactions of the borane **12a**

**Scheme 5:**
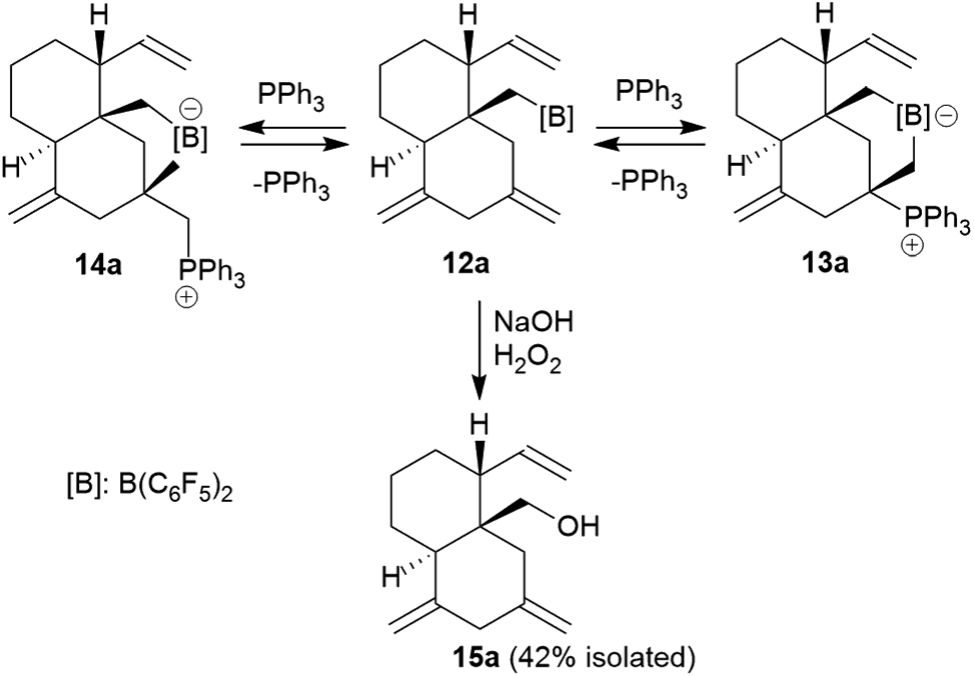
Reaction of compound **12a** with PPh_3_ and oxidative replacement of the boryl group.

**Fig. 5:**
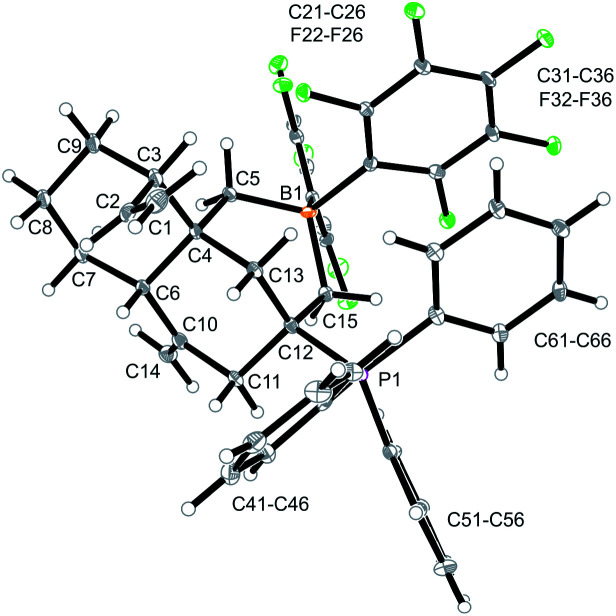
A view of the P/B FLP alkene addition product **13a**. Selected bond lengths (Å) and angles (°): B1–C5 1.640(6), B1–C15 1.667(6), P1–C12 1.858(4), C1–C2 1.302(6), C4–C6 1.565(5), C10–C14 1.328(6), C12–C15 1.555(5), C5–B1–C15 110.3(3), C12–C15–B1 114.3(3), P1–C12–C15–B1 −129.8(3).

Compound **12a** is a reactive borane and it contains C=C double bond functionalities. Therefore, it should be suitable to undergo typical FLP addition to one of the olefinic units in the presence of an external phosphane nucleophile.^11^ We, consequently, reacted the *in situ* generated borane **12a** with triphenylphosphane in dichloromethane. The reaction with PPh_3_ was instantaneous. The volatiles were removed and the residue was washed with pentane to give the P/B addition product **13a**, which we isolated as a white solid in 56% yield (see Scheme 5). Compound **13a** was characterized by C, H-elemental analysis, by spectroscopy and by X-ray diffraction (Fig. 5). It showed that a P/B FLP addition had taken place at the proximal C=CH_2_ moiety (C12=C15 in compound **12a**, see Fig. 4) by using the adjacent pendent internal borane and the external phosphane. Compound **13a** is the isomer that was formed by borane addition to the C=CH_2_ terminus and, consequently, phosphane addition to the sp^2^-ring carbon atom. This resulted in the formation of a heterocyclic six-membered ring-system that had become 1,3-attached at the “lower” six-membered decalin ring of compound **12a**. The phosphonium PPh_3_^+^ moiety is found attached at the new bridgehead atom C12 (Fig. 5).

Compound **13a** shows a typical borate ^11^B NMR resonance at *δ* −15.1 in solution (CD_2_Cl_2_, 273 K) and a phosphonium ^31^P NMR signal at *δ* 31.1. It shows the ^19^F NMR features of a pair of diastereotopic C_6_F_5_ groups at the boron atom (for further details of the NMR characterization of compound **13a** see the ESI†).

Compound **13a** is the P/B FLP addition product that has been formed under kinetic control. When we stored the CD_2_Cl_2_ solution of compound **13a** for 7 days at room temperature the resulting NMR spectra showed the formation of an equilibrium mixture of **13a** (*ca.* 20 mol%), the starting material **12a** (plus PPh_3_, *ca.* 8 mol%) and the new compound **14a** (*ca.* 65 mol%) (plus some minor contaminants). The major product **14a**, apparently formed under thermodynamic control, was prepared similarly on a preparative scale (24 h, r.t., CH_2_Cl_2_ layered with pentane) and crystallized from the mixture. Crystalline compound **14a** was isolated in 60% yield and the product was characterized by C, H-elemental analysis, by spectroscopy and by X-ray diffraction.

**Fig. 6:**
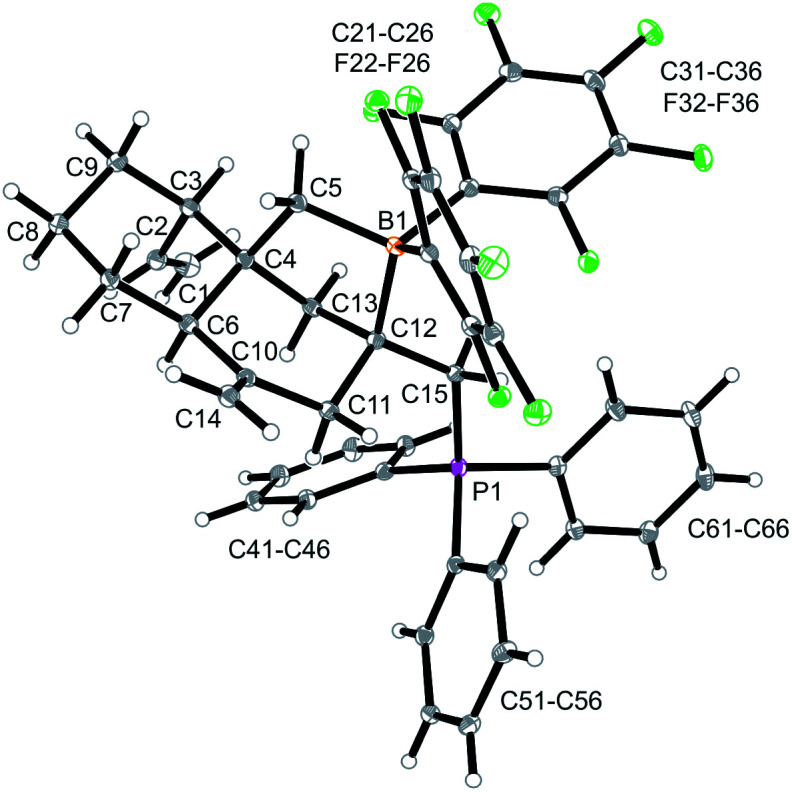
A projection of the molecular structure of compound **14a**. Selected bond lengths (Å) and angles (°): B1–C5 1.657(4), B1–C12 1.695(3), P1–C15 1.823(2), C1–C2 1.301(13), C4–C6 1.556(3), C10–C14 1.325(4), C12–C15 1.540(3), C5–B1–C12 99.9(2), C12–C15–B1 112.1(2), B1–C12–C15–P1 179.1(2).

The X-ray crystal structure analysis of **14a** shows the presence of a five-membered boratacycle that is 1,3-annulated to the “lower” six-membered ring of the *trans*-decalin framework. It was apparently formed by a 1,2-P/B FLP addition to the C12=C15 carbon–carbon double bond of the starting material **12a**, similar as we had seen it in the formation of its isomer **13a**, only that in this case PPh_3_ addition had taken place at the =CH_2_ terminus of the exo-methylene group concurrent with borane addition to its adjacent doubly substituted sp^2^-carbon atom. The structure of the resulting P/B zwitterion **14a** is depicted in Fig. 6.

The ^1^H NMR spectrum of compound **14a** (in CD_2_Cl_2_, at 299 K) shows the P-coupled system of the exocyclic –CH_2_–[P] moiety at *δ* 4.18/2.70 and the resonances of the endocyclic –CH_2_–[B] group at *δ* 1.32/0.35. The heteroatom NMR signals occur at *δ* −6.8 (^11^B) and 19.9 (^31^P), respectively and we observed two sets of ^19^F NMR signals of the pair of diastereotopic C_6_F_5_ groups at boron (for further details see the ESI†).

We eventually converted the borane-induced multi-component cyclization product **12a** to a boron-free derivative.^14^ This was carried out in the usual way of oxidative deborylation as it is done in conventional hydroboration chemistry.^15^ Treatment of the strongly electrophilic –B(C_6_F_5_)_2_ borane **12a** with NaOH/H_2_O_2_ gave the alcohol **15a** that we isolated as a white solid in 42% yield after workup (see the ESI† for its characterization by NMR spectroscopy).

**Scheme 6:**
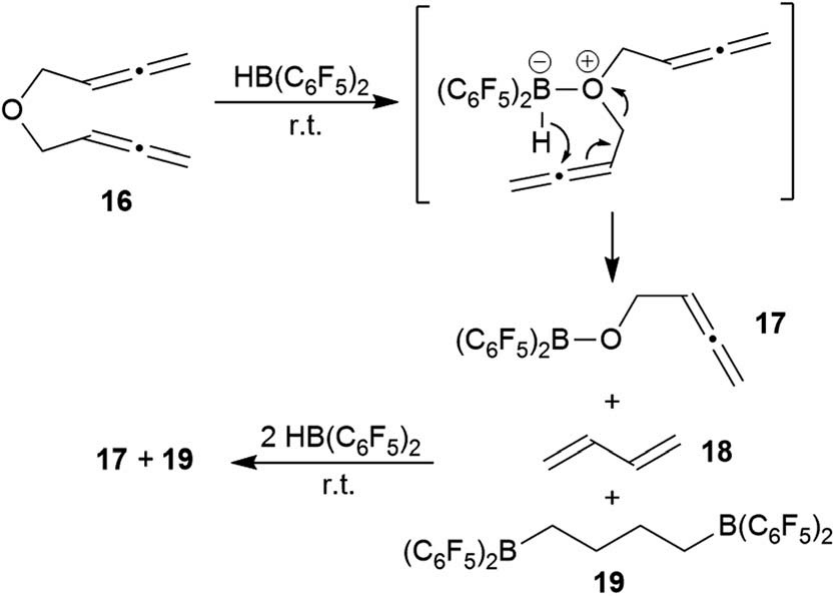
Reaction of the bis-allenic ether **16** with HB(C_6_F_5_)_2_.

We briefly investigated the reaction of the bis-allenic ether **16**^6*c*,*h*,*j*^ with HB(C_6_F_5_)_2_. The reaction was carried out in CD_2_Cl_2_ solution at r.t. The products of the reaction were not isolated but directly identified *in situ* generated from the solution. We subsequently added a total of three molar equivalents of HB(C_6_F_5_) to eventually achieve a complete conversion of compound **16** with a clean product formation. The NMR analysis (for details see the ESI†) revealed that the by far predominant reaction was ether cleavage. This gave the (C_6_F_5_)_2_B–O–CH_2_–CH=C=CH_2_ cleavage product **17** (see Scheme 6) and butadiene (**18**) as primary products. The latter was then subsequently converted by added HB(C_6_F_5_)_2_ to the bis-hydroboration product **19**. The boryl ether **17** also was not stable under the reaction conditions, probably due to subsequent ether cleavage with additional HB(C_6_F_5_)_2_ (see the ESI† for details). We also investigated briefly the reaction of the respective bis-allenic *N*-tosyl amine with HB(C_6_F_5_)_2_, but that gave a complicated mixture of as yet unidentified products.

## Conclusions

With this study we have found a new variant of our borane induced carbon–carbon coupling reactions between allene building blocks. In this case the reaction starts as it is commonly observed in our systems by 1,2-[B]–H addition^16^ to a terminal allene =CH_2_ group by the strongly electrophilic HB(C_6_F_5_)_2_ hydroboration reagent to probably generate a reactive allylborane intermediate *in situ*, which is set for undergoing rapid intramolecular ring-closure with the pendant second allenyl moiety to generate the products **5a** to **5c**, respectively. These are then obviously protected by their special geometry from undergoing further intermolecular allylborane coupling under the applied reaction conditions, so that the reaction stopped at the functionalized six-or seven-membered ring products. The compounds **5** are, however, in principle still active allylboration reagents. This we could show by the rapid reaction of the example **5a** with the parent allene H_2_C=C=CH_2_. Two equivalents of allene were consumed in a sequence of consecutive intramolecular allylboration reactions, followed by a final intramolecular allylboration ring-closure reaction to give the four-component coupling product **12a**. This in turn was oxidatively converted to the boron-free product **15a**. These metal-free reactions are markedly different from the common metal catalysed bis-allenic cyclization reactions reported in the literature (see Chart 1 and the respective references). We will see how the products of our metal free cyclization reactions and their follow-up products (and related systems) might become easily available useful reagents for further external C–C coupling reactions using either of the newly generated functionalities.

## Conflicts of interest

There are no conflicts to declare.

## Supplementary Material

SC-011-C9SC03870A-s001

SC-011-C9SC03870A-s002
