# Supplementary material for: Borane-induced ring closure reaction of oligomethylene-linked bis-allenes
Source: Chem Sci. 2019 Dec 18;11(6):1542–8. doi: 10.1039/c9sc03870a (PMC8148058; doi:10.1039/c9sc03870a)
Supplement: SC-011-C9SC03870A-s001 [file SC-011-C9SC03870A-s001.pdf]

Electronic Supplementary Material (ESI) for Chemical Science.
This journal is © The Royal Society of Chemistry 2020

Borane-induced Ring Closure Reaction of Oligomethylene-linked Bis-allenes

Xin Tao, Karel Škoch, Constantin G. Daniliuc, Gerald Kehr, Gerhard Erker*

Organisch-Chemisches Institut, Westfälische Wilhelm-Universität Münster, Corrensstraße 40, 48149 Münster, Germany

Supporting Information

Table of Contents

A) Synthesis of starting material S3

B) Synthesis of bisallenes **3a**, **3b, 3c** and **16** S5

C) Generation of compound **5a** S10

D) Synthesis of compound **6a** S13

E) Generation of compound **5b** S17

F) Synthesis of compound **6b** S19

G) Generation of compound **5c** S24

H) Synthesis of compound **6c** S27

I) Generation of compound **7a** S31

J) Synthesis of compound **8a** S34

K) Synthesis of compound **9a** S38

L) Synthesis of compound **9b** S44

M) Synthesis of compound **9c** S47

N) Generation and synthesis of compound **12a** S52

O) Synthesis of compound **13a** and **14a** S58

P) Synthesis of compound **15a** S75

Q) Attempted cyclization of bisallene **16** S78

General Information. All reactions involving air- or moisture-sensitive compounds were carried out under an inert gas atmosphere (Argon) by using Schlenk-type glassware or in a glovebox. All solvents were dried and degassed before use, if necessary for the respective reaction. Chemicals: Unless otherwise noted all chemicals were used as purchased. The following instruments were used for physical characterization of the compounds: melting points: elemental analyses: Foss-Heraeus CHNO-Rapid; NMR: Varian UNITY plus NMR spectrometer (^{1}H, 600 MHz; ^{13}C, 151 MHz; ^{11}B, 192 MHz; ^{19}F, 564 MHz; ^{31}P, 243 MHz). NMR chemical shifts are given relative to $SiMe_4$ and referenced to the respective solvent signals (^{1}H and ^{13}C) or external standard [δ($BF_3{\cdot}OEt_2$) = 0 for ^{11}B NMR, δ($CFCl_3{\cdot}OEt_2$) = 0 for ^{19}F NMR]. NMR assignments were supported by additional 2D NMR experiments.

X-Ray diffraction: Data sets for compounds **6a**, **6b**, and **9c** were collected with a Bruker D8 Venture CMOS diffractometer. For compounds **8a**, **9a**, **9b**, **12a**, **13a** and **14a** data sets were collected with a Bruker APEX II CCD diffractometer. Programs used: data collection: APEX3 V2016.1-0 (Bruker AXS Inc., **2016**); cell refinement: SAINT V8.37A (Bruker AXS Inc., **2015**); data reduction: SAINT V8.37A (Bruker AXS Inc., **2015**); absorption correction, SADABS V2014/7 (Bruker AXS Inc., **2014**); structure solution *SHELXT-2015* (Sheldrick, G. M. *Acta Cryst.,* **2015**, *A71*, 3-8); structure refinement *SHELXL-2015* (Sheldrick, G. M. *Acta Cryst.,* **2015**, *C71* (1), 3-8) and graphics, *XP* (Version 5.1, Bruker AXS Inc., Madison, Wisconsin, USA, **1998**). *R*-values are given for observed reflections, and wR^2 values are given for all reflections. *Exceptions and special features*: For compounds **6b** and **9b** part of the seven membered ring and for compound **14a** one dichloromethane molecule and one ethenyl group were found disordered over two positions in the asymmetric unit. Several restraints (SADI, SAME, ISOR and SIMU) were used in order to improve refinement stability. Additionally, for compound **8a** a badly disordered half dichloromethane molecule, for compound **9a** a badly disordered solvent molecule (probably a half dichloromethane molecule), for compound **9b** a badly disordered mixture of dichloromethane and pentane molecules were found in the asymmetrical unit and could not be satisfactorily refined. The program SQUEEZE (Spek, A.L. (2015). Acta Cryst. C71, 9-18.) was therefore used to remove mathematically the effect of the solvent. The quoted formula and derived parameters are not included the squeezed solvent molecules. CCDC deposition numbers are 1922906-1922913 and 1957134.

Materials. $HB(C_6F_5)_2$ (Piers' borane) was prepared according to procedures described in the literatures [a) D. J. Parks, R. E. von H. Spence and W. E. Piers, *Angew. Chem. Int. Ed. Engl.*, 1995, **34**, 809–811; *Angew. Chem.,* 1995, **107**, 895–897; b) D. J. Parks, W. E. Piers and G. P. A. Yap, *Organometallics*, 1998, **17**, 5492–5503]. Bisallene (**3a** and **3b**) were prepared according to procedures described in the literature [J. Kuang and S. Ma, *J. Org. Chem.,* 2009, **74**, 1763-1765]. 1,6-Heptadiyne, 1,7-octadiyne and dipropargylether were purchased from Sigma-Aldrich and used as received.

A) Synthesis of starting material

Scheme S1

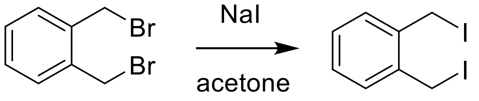

The following procedure was adapted from a method described in the literature [T. Takahashi, S. Li, W. Huang, F. Kong, K. Nakajima, B. Shen, T. Ohe and K. Kanno, *J. Org. Chem.,* 2006, **71**, 7964-7977]:

α,α´-Dibromo-*o*-xylene (3.0 g, 11.4 mmol) and NaI (8.9 g, 68.4 mmol) were suspended in acetone (50 mL) and allowed to react 15 hours at room temperature. Subsequently, volatilities were removed under reduced pressure and the remaining solid residue was treated with diethylether (80 mL) and water (50 mL). Phases were separated and the ethereal fraction was washed with 10 % solution of $Na_2S_2O_3$, brine and then dried over $MgSO_4$. After removal of volatilities in vacuo, the residue was passed through a short silica pad using a mixture of pentane and diethylether (25 : 1) as eluent. Then, after removal of all volatilities in vacuo, a yellow microcrystalline solid was obtained (3.94 g, 11.0 mmol, 96 % yield).

Scheme S2

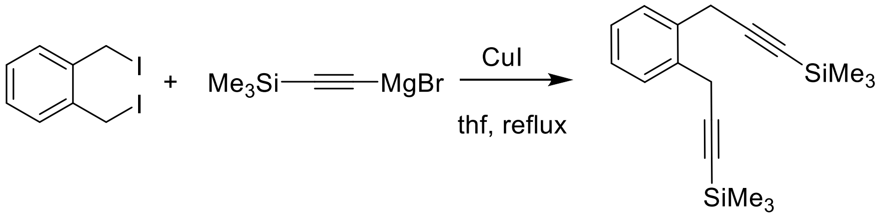

The following procedure was adapted from a method described in the literature [T. Takahashi, S. Li, W. Huang, F. Kong, K. Nakajima, B. Shen, T. Ohe and K. Kanno, *J. Org. Chem.,* 2006, **71**, 7964-7977]:

An ice cooled solution of trimethylsilyl acetylene (7.1 mL, 50 mmol) in tetrahydrofuran (50 ml) was treated with ethyl magnesium bromide (3M solution in diethylether, 16.6 mL, 50 mmol) and allowed to react for 15 minutes while cooling, followed by one hour at room temperature. Subsequently α,α´-diiodo-*o*-xylene (3.94 g, 11.0 mmol) and CuI (0.95 g, 5 mmol) were added to the resulting white suspension. The mixture was heated to reflux for 3 hours, then it was cooled to room temperature and carefully quenched with aqueous solution of $NaHCO_3$ (ca 20 mL). The resulting mixture was diluted with water (50 mL) and diethylether (50 mL). The organic phase was separated and the aqueous fraction was extracted with diethyl ether (3 x 30 mL). The combined organic fractions were washed with brine, dried over $MgSO_4$, filtered and then all volatilities were removed in vacuo. The

obtained oily residue was purified by filtration through a short silica pad using a mixture of pentane and diethyl ether (50:1) as eluent. The product was obtained as a colourless oil (3.05 g, 10.2 mmol, 93%). It was used without further purification in the following step.

Scheme S3

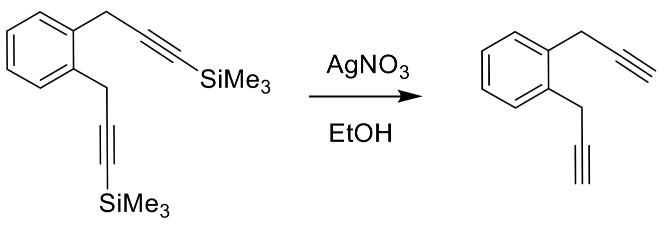

The following procedure was adapted from a method described in the literature [M. Hiller, S. Krieg, N. Ishikawa and M. Enders, *Inorg. Chem.,* 2017, **56**, 15285-15294]:

A solution of $AgNO_3$ (5.14 g, 30.6 mmol) in water (20 mL) was added in portions to a solution of 1,2-bis(3-trimethylsilylprop-2-ynyl)benzene (3.05 g, 10.2 mmol) in untreated ethanol (80 mL). The mixture turned into a thick white suspension, which was allowed to react for 14 hours at room temperature. After the stirred reaction mixture was quenched by addition of a solution of $Na_2S_2O_3$ (14.8 g of pentahydrate, 60 mmol) in water (20 mL), it discoloured after 15 minutes. Subsequently it was passed through a pad of Celite, which was thoroughly washed with acetone (ca. 150 mL). The filtrate was concentrated on the rotary vacuum evaporator (40°C, 200 mbar) to ca. one quarter of the starting volume. This residue was extracted with dichloromethane (3 x 40 mL). The combined extracts were washed with brine and dried over $MgSO_4$. After filtration and removal of all volatiles in vacuo, the remaining residue was purified though a silica column using pentane as eluent. The product was isolated as a colourless oil (1.40 g, 9.1 mmol, 89%; 80% in total over three steps from α,α´-dibromo-*o*-xylene).

^{1}H NMR (600 MHz, 299 K, $CDCl_3$): δ ^{1}H: [7.48, 7.28](each m, each 1H, $CH^{phenylene}$), 3.64 (d, $^4J_{HH}$ = 2.8 Hz, 2H, CH_2), 2.21 (t, $^4J_{HH}$ = 2.8 Hz, 1H, ≡CH).

^{13}C{^{1}H} NMR (151 MHz, 299 K, $CDCl_3$): δ ^{13}C: 133.9 ($C^{phenylene}$), [128.8, 127.4]($CH^{phenylene}$), 81.1 (C≡), 71.0 (≡CH), 22.5 (CH_2).

B) Synthesis of bisallenes **3a**, **3b**, **3c**, and **16**

Scheme S4.

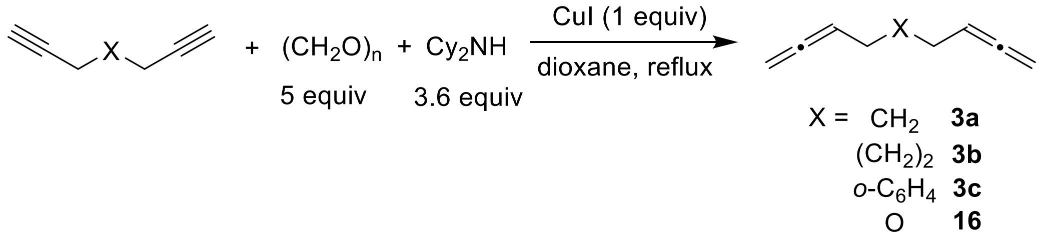

The bisallenes **3a**,**b**,**c** and **16** were synthesized according to a procedure described in the literature [J. Kuang and S. Ma, *J. Org. Chem.,* 2009, **74**, 1763-1765]: $(CH_2O)_n$ (5 equiv.), CuI (1 equiv.), dioxane (50 mL), bisacetylene (5 or 10 mmol) and Cy_2NH (3.6 equiv.) were mixed sequentially into an oven-dried reaction tube equipped with a reflux condenser under an Argon atmosphere. The resulting mixture was stirred under reflux. After the reaction was complete as monitored by TLC, the reaction mixture was cooled down to room temperature. Water (50 mL) and ether (100 mL) were added to the resulting reaction mixture. The aqueous solution was separated and extracted with ether (3 × 50 mL). Then the combined organic layers were washed with brine and dried over anhydrous Na_2SO_4. Drying in vacuo followed by column chromatography on silica gel (eluent: pentane) gave the corresponding bisallenes (**3a**,**b**,**c** or **16**).

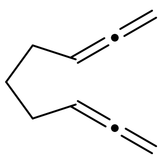

3a

<u>Compound **3a**</u> (240 mg, 2.0 mmol, 40%) was isolated as a colorless liquid.

^{1}H NMR (600 MHz, 299 K, CD_2Cl_2): δ ^{1}H: 5.11 (m, 1H, =CH), 4.66 (dt, $^{4}J_{HH}$ = 6.7 Hz, $^{5}J_{HH}$ = 3.3 Hz, 2H, =CH_2), 2.04 (m, 2H, $^{=CH}CH_2$), 1.54 (m, 1H, CH_2).

^{13}C{^{1}H} NMR (151 MHz, 299 K, CD_2Cl_2): δ ^{13}C: 209.0 (=C=), 89.9 (=CH), 74.7 (=CH_2), 28.9 (CH_2), 28.0 ($^{=CH}CH_2$).

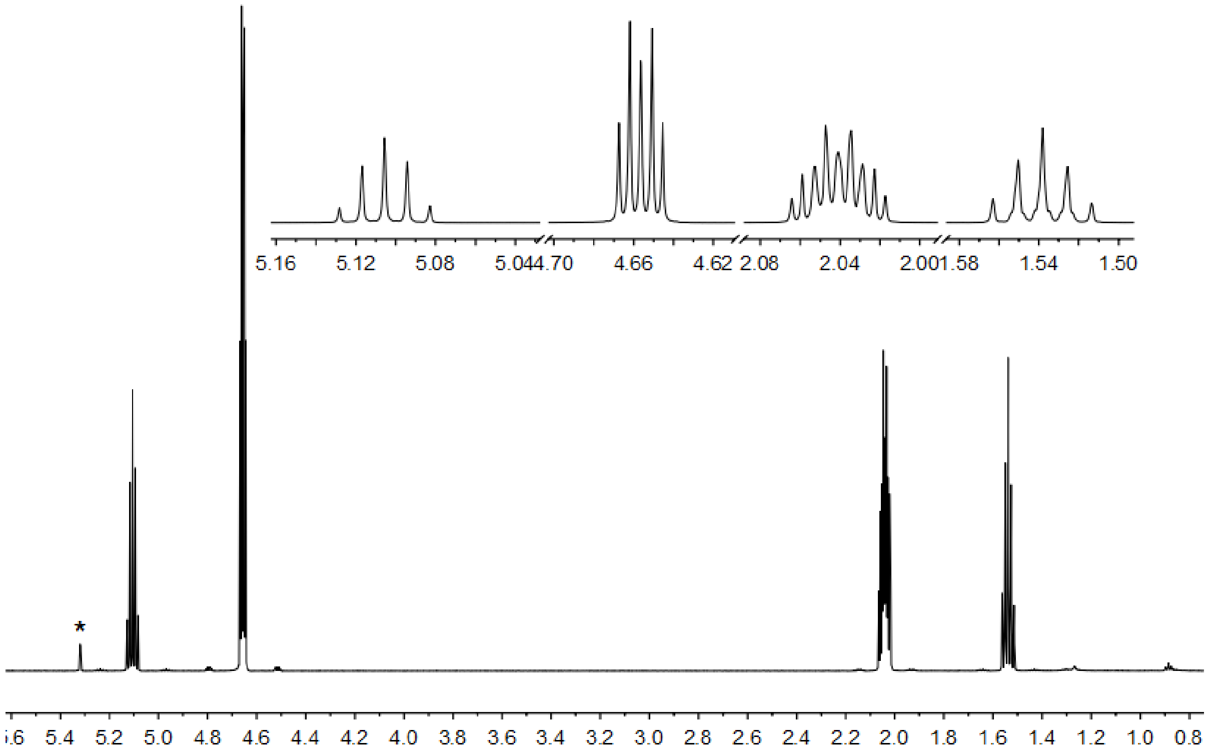

Figure S1. ^{1}H NMR (600 MHz, 299 K, CD_2Cl_2) spectrum of compound **3a**.

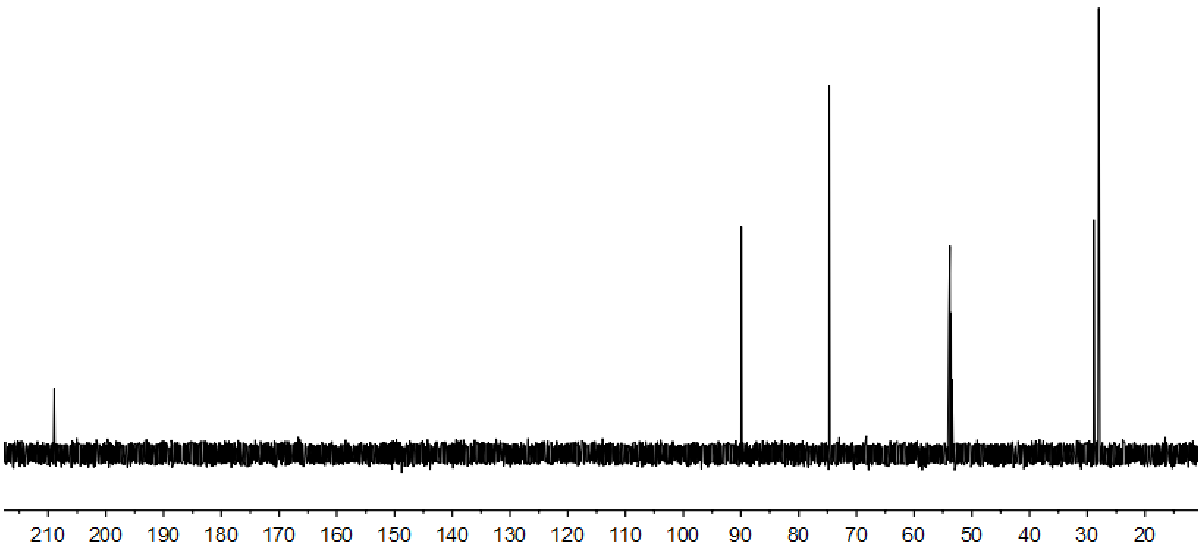

Figure S2. ^{13}C{^{1}H} NMR (151 MHz, 299 K, CD_2Cl_2) spectrum of compound **3a**.

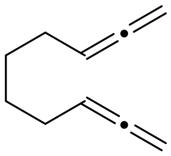

3b

<u>Compound **3b**</u> (422 mg, 6.3 mmol, 63%) was isolated as a colorless liquid.

^{1}H NMR (600 MHz, 299 K, CD_2Cl_2): δ ^{1}H: 5.11 (m, 1H, =CH), 4.65 (dt, $^4J_{HH}$ = 6.7 Hz, $^5J_{HH}$ = 3.2 Hz, 2H, $=CH_2$), 2.00 (m, 2H, $^{=CH}CH_2$), 1.45 (m, 2H, CH_2).

^{13}C{^{1}H} NMR (151 MHz, 299 K, CD_2Cl_2): δ ^{13}C: 208.4 (=C=), 89.8 (=CH), 74.2 ($=CH_2$), 28.5 (CH_2), 28.0 ($^{=CH}CH_2$).

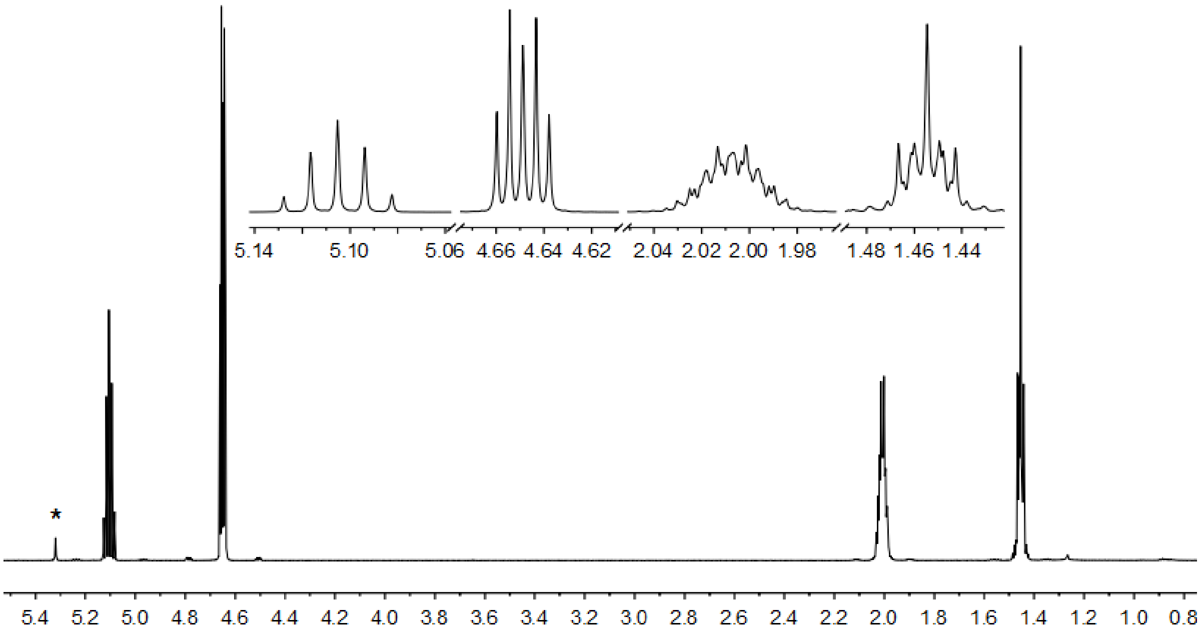

Figure S3. ^{1}H NMR (600 MHz, 299 K, CD_2Cl_2) spectrum of compound **3b**.

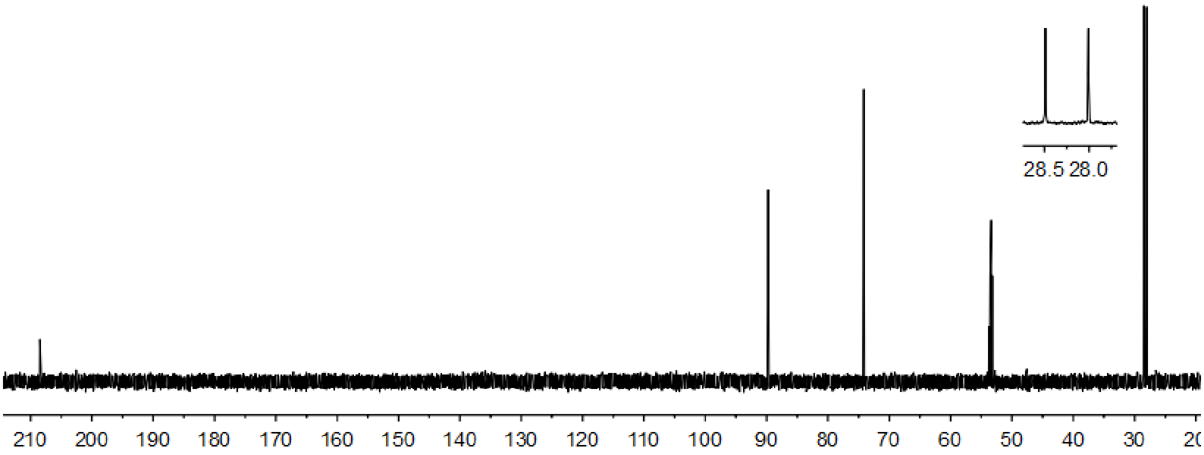

Figure S4. ^{13}C{^{1}H} NMR (151 MHz, 299 K, CD_2Cl_2) spectrum of compound **3b**.

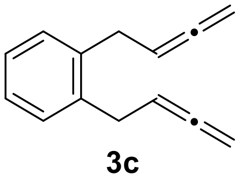

3c

Compound **3c** (550 mg, 3.0 mmol, 60%) was isolated as a colorless liquid.

HRMS (ESI) m/z: calc. for $C_{15}H_{22}O$ $[M-H]^+$: 181.1009. Found: 181.1012.

^{1}H NMR (600 MHz, 299 K, CD_2Cl_2): δ ^{1}H: [7.24, 7.20](each m, each 1H, $CH^{phenylene}$), 5.30 (m, 1H, =CH), 4.73 (dt, $^4J_{HH}$ = 6.7 Hz, $^5J_{HH}$ = 3.1 Hz, 2H, $=CH_2$), 3.42 (dt, $^3J_{HH}$ = 7.1 Hz, $^5J_{HH}$ = 3.1 Hz, 2H, CH_2).

^{13}C{^{1}H} NMR (151 MHz, 299 K, CD_2Cl_2): δ ^{13}C: 209.3 (=C=), 138.7 ($C^{phenylene}$), [129.8, 126.9]($CH^{phenylene}$), 89.7 (=CH), 75.3 ($=CH_2$), 32.6 (CH_2)

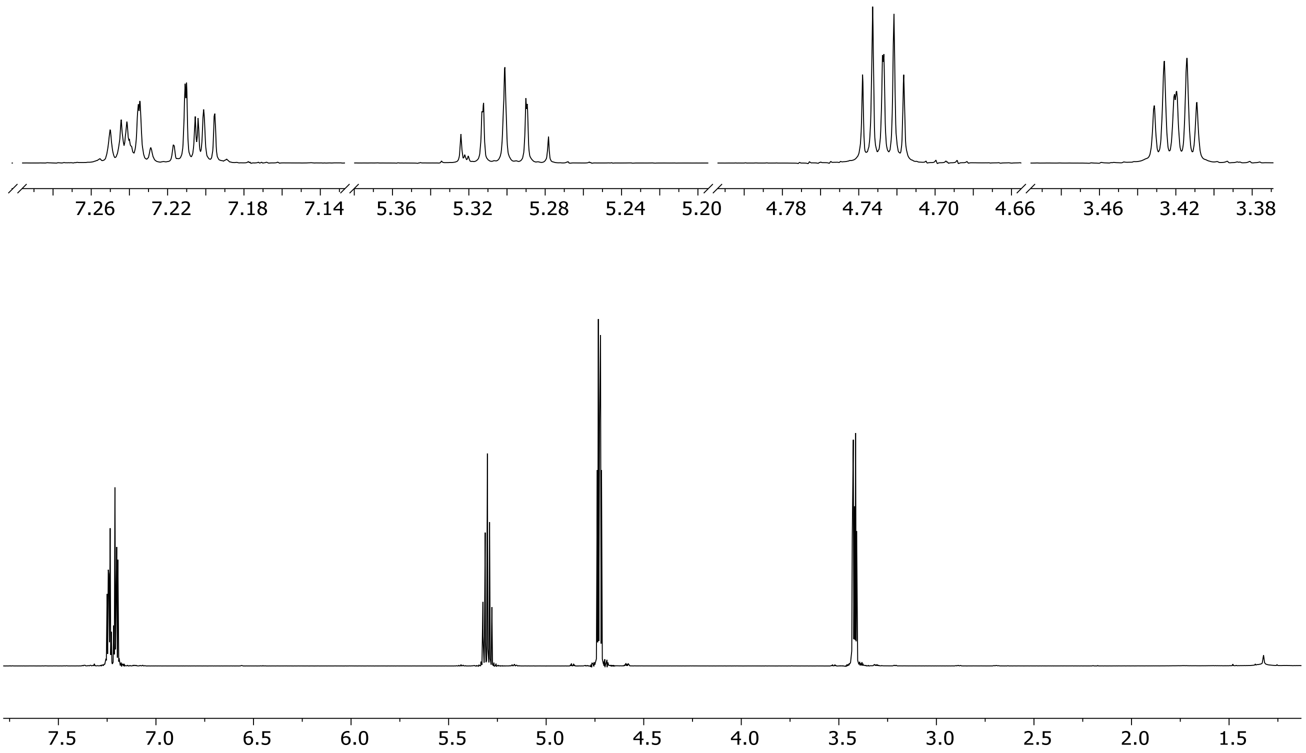

Figure S5. ^{1}H NMR (600 MHz, 299 K, CD_2Cl_2) spectrum of compound **3c**.

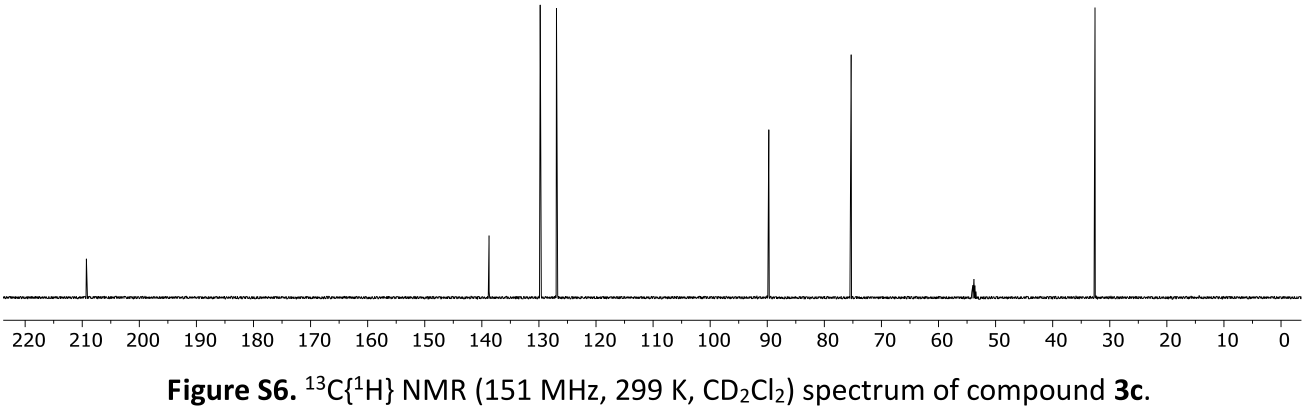

Figure S6. ^{13}C{^{1}H} NMR (151 MHz, 299 K, CD_2Cl_2) spectrum of compound **3c**.

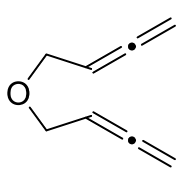

16

<u>Compound **16**</u> (996 mg, 8.2 mmol, 82%) was isolated as a colorless liquid.

^{1}H NMR (600 MHz, 299 K, CD_2Cl_2): δ ^{1}H: 5.22 (quint, $^3J_{HH}$ = $^4J_{HH}$ = 6.7 Hz, 1H, =CH), 4.79 (dt, $^4J_{HH}$ = 6.7 Hz, $^5J_{HH}$ = 2.5 Hz, $=CH_2$), 4.01 (dt, $^3J_{HH}$ =6.7 Hz, $^5J_{HH}$ = 2.5 Hz, OCH_2).

^{13}C{^{1}H} NMR (151 MHz, 299 K, CD_2Cl_2): δ ^{13}C: 209.6 (=C=), 88.0 (=CH), 75.6 ($=CH_2$), 67.8 (OCH_2).

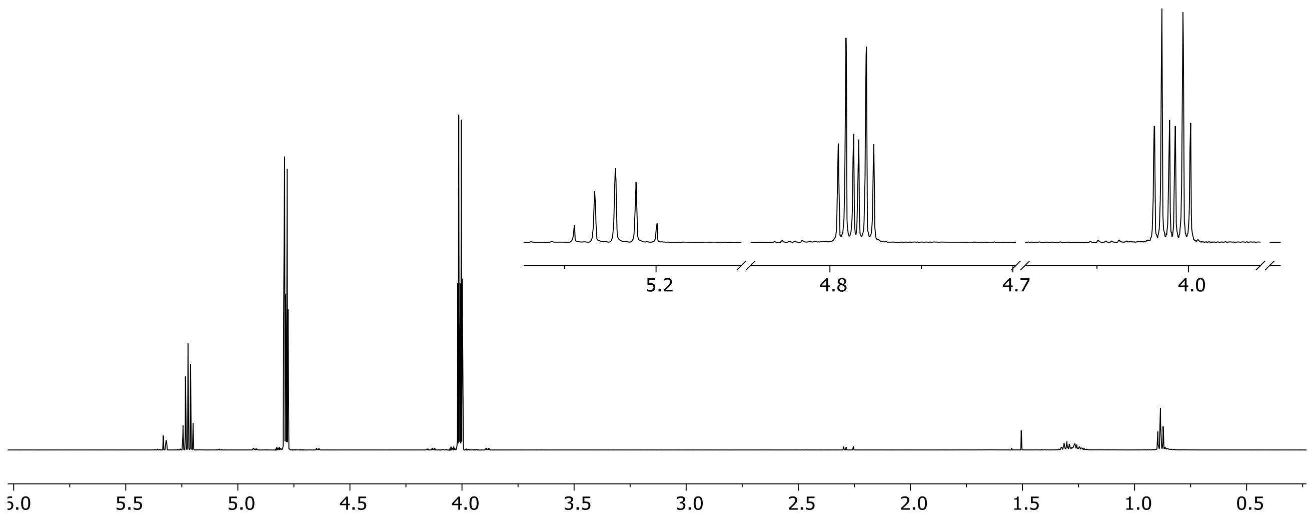

Figure S7. ^{1}H NMR (600 MHz, 299 K, CD_2Cl_2) spectrum of compound **16**.

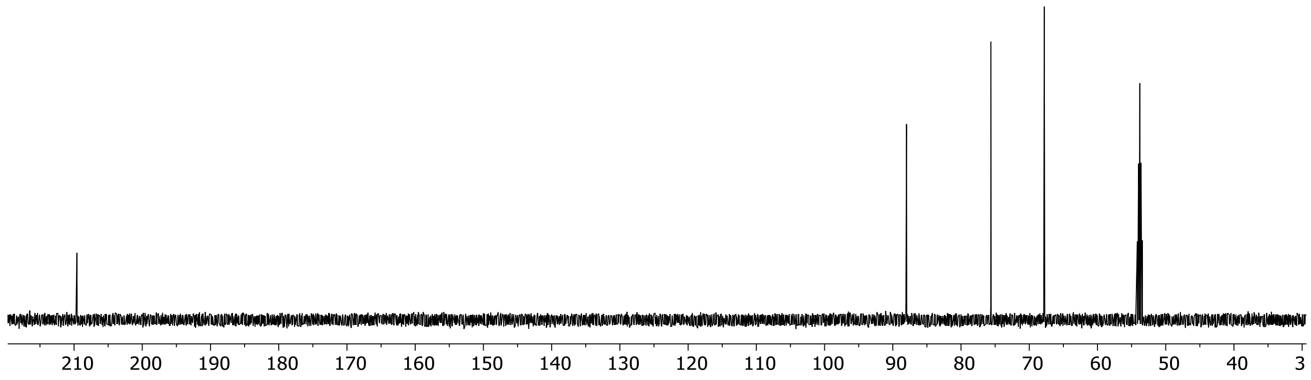

Figure S8. ^{13}C{^{1}H} NMR (151 MHz, 299 K, CD_2Cl_2) spectrum of compound **16**.

C) Generation of compound **5a**

Scheme S5.

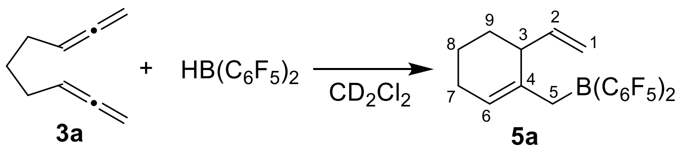

A suspension of $HB(C_6F_5)_2$ (34.6 mg, 0.10 mmol) in CD_2Cl_2 (0.5 mL) was added to a solution of bisallene **3a** (12.0 mg, 0.10 mmol) in CD_2Cl_2 (0.5 mL) at room temperature. Subsequently, the resulting reaction mixture was characterized by NMR experiments.

NMR data of compound **5a** from the reaction mixture:

^{1}H NMR (500 MHz, 299 K, CD_2Cl_2): δ ^{1}H: 5.55 (m, 1H, 6-CH=), 5.52 (m, 1H, 2-CH=), 5.03 (m, 2H, CH_2=), 2.92/2.73 (each d, $^2J_{HH}$ = 16.3 Hz, each 1H, BCH_2), 2.44 (m, 1H, CH), 1.95 (m, 2H, 7-CH_2), 1.60/1.48 (each m, each 1H, 9-CH_2), 1.53/1.42 (each m, each 1H, 8-CH_2).

^{13}C{^{1}H} NMR (126 MHz, 299 K, CD_2Cl_2): δ ^{13}C: 147.5 (dm, $^1J_{FC}$ ~ 250 Hz, C_6F_5), 143.5 (dm, $^1J_{FC}$ ~ 260 Hz, C_6F_5), 142.1 (2-CH=), 137.8 (dm, $^1J_{FC}$ ~ 250 Hz, C_6F_5), 135.6 (C=), 127.2 (6-CH=), 116.6 (CH_2=), 114.6 (br, i-C_6F_5), 45.7 (CH), 39.2 (br, BCH_2), 29.7 (9-CH_2), 26.2 (7-CH_2), 19.2 (8-CH_2).

^{19}F NMR (470 MHz, 299 K, CD_2Cl_2): δ ^{19}F: –129.2 (m, 2F, *o*), –149.2 (tt, $^3J_{FF}$ = 20.0 Hz, $^4J_{FF}$ = 5.0 Hz, 1F, *p*), –162.3 (m, 2F, *m*)(C_6F_5)[$\Delta\delta^{19}F_{m,p}$ = 13.1].

^{11}B{^{1}H} NMR (160 MHz, 299 K, CD_2Cl_2): δ ^{11}B: 69.3 ($\nu_{1/2}$ ~ 600 Hz).

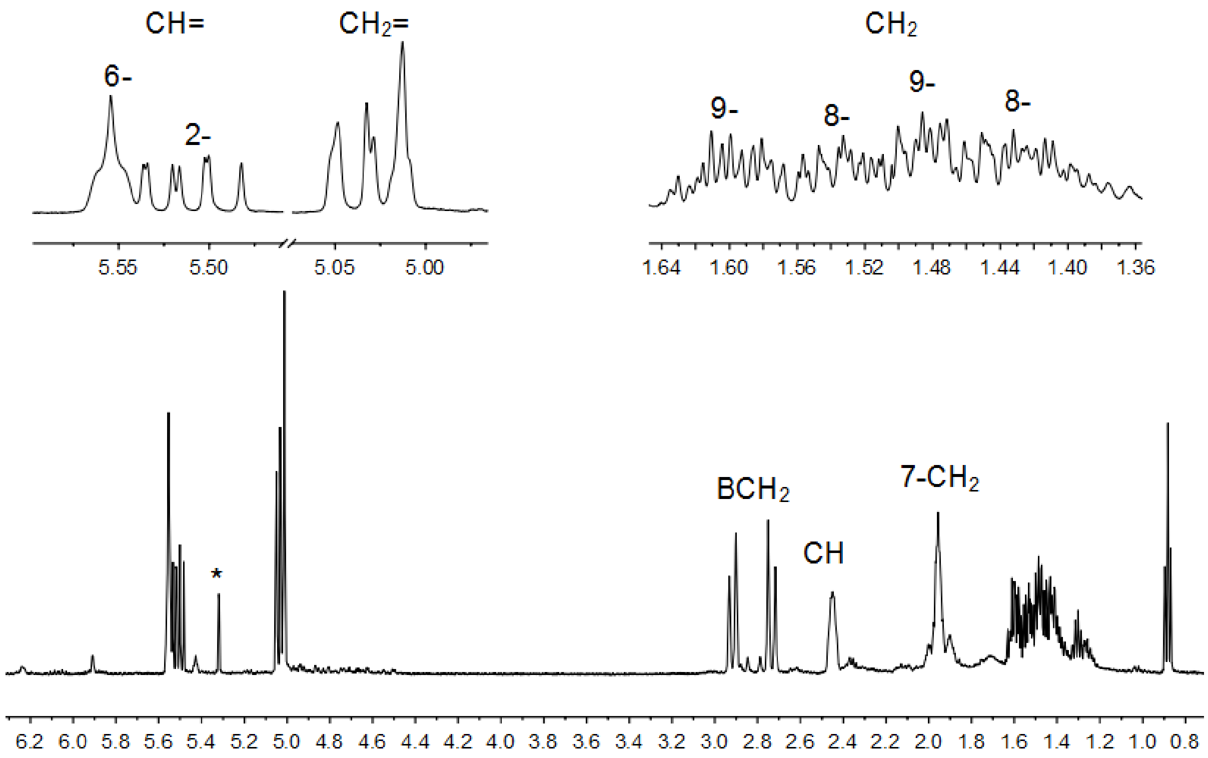

Figure S9. ^{1}H NMR (500 MHz, 299 K, CD_2Cl_2*) spectrum of the reaction mixture. [admixed with pentane]

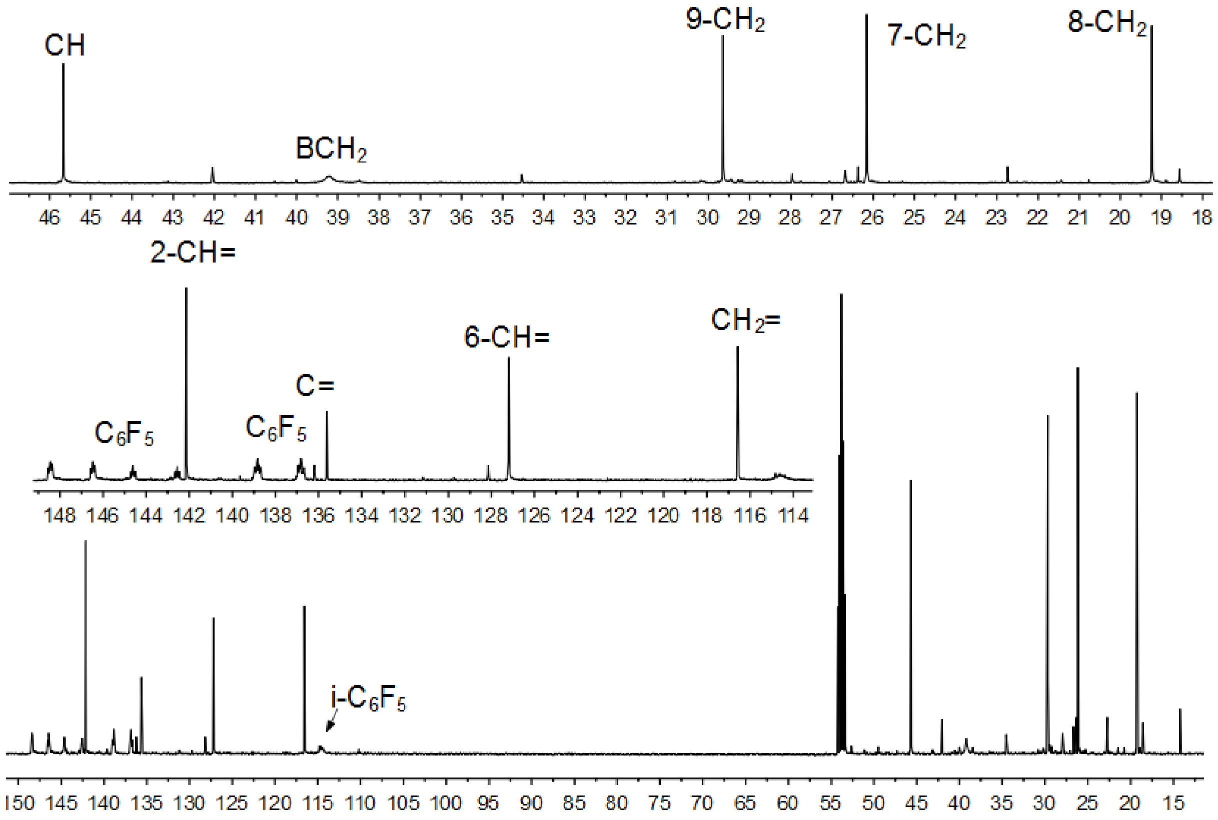

Figure S10. $^{13}C\{^{1}H\}$ NMR (126 MHz, 299 K, CD_2Cl_2) spectrum of the reaction mixture.

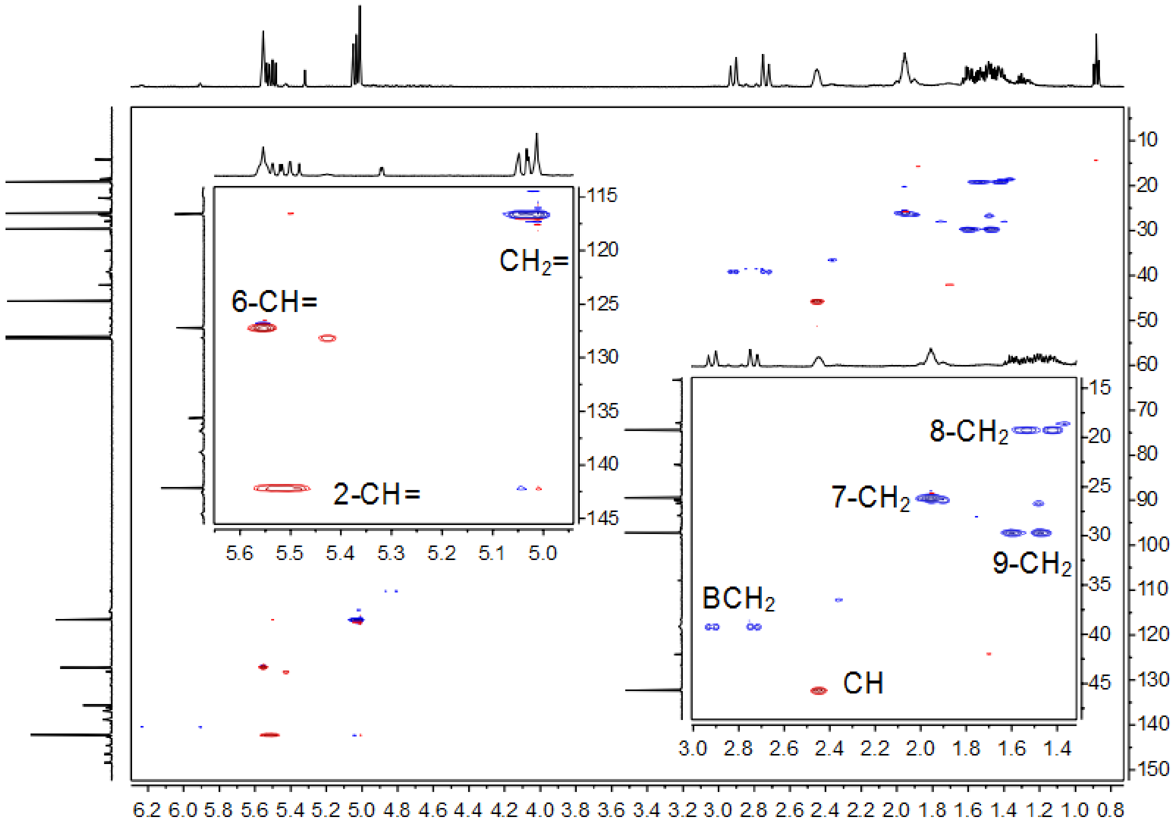

Figure S11. $^{1}H,^{13}C$ GHSQC (500/126 MHz, CD_2Cl_2, 299K) spectrum of reaction mixture.

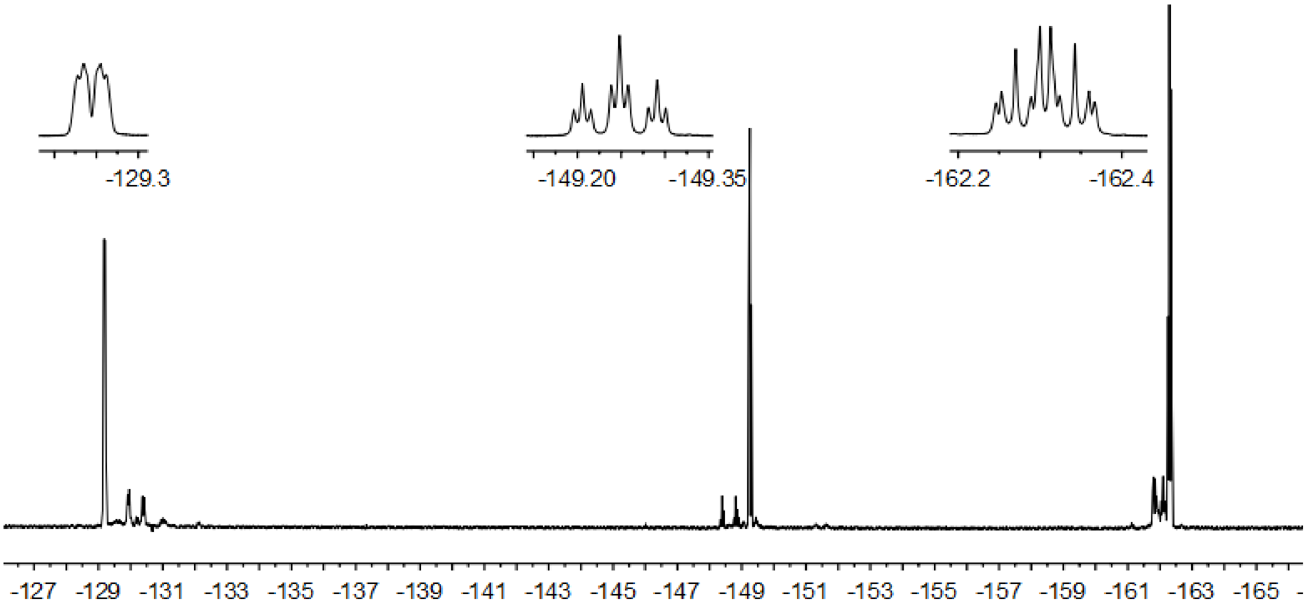

Figure S12. ^{19}F NMR (470 MHz, 299 K, CD_2Cl_2) spectrum of the reaction mixture.

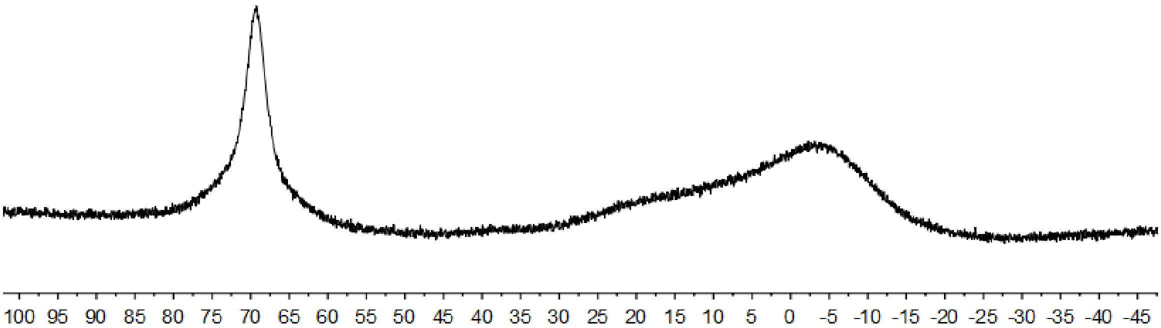

Figure S13. $^{11}B\{^1H\}$ NMR (160 MHz, 299 K, CD_2Cl_2) spectrum of the reaction mixture.

D) Synthesis of compound **6a**

Scheme S6.

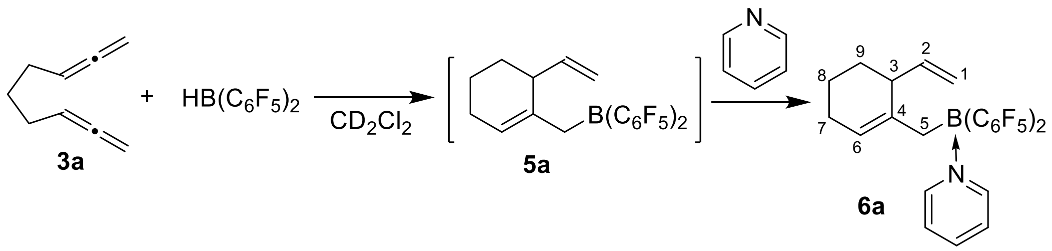

A suspension of $HB(C_6F_5)_2$ (103.8 mg, 0.30 mmol) in CH_2Cl_2 (1 mL) was added to a solution of bisallene **3a** (40.0 mg, 0.33 mmol) in CH_2Cl_2 (1 mL) at room temperature. Subsequently, pyridine (32 mg, 0.40 mmol) was added to the resulting reaction mixture. Then all volatiles were removed in vacuo and the residue was washed with pentane (1 mL × 10). Slow evaporation of the combined pentane solution gave white crystalline materials, part of which were used for the X-ray crystal structure analysis. The rest were carefully washed with pentane (1 mL× 3) and dried in vacuo giving compound **6a** (120 mg, 0.22 mmol, 73%) as a white solid.

Anal. Calc. for $C_{26}H_{18}BF_{10}N$: C, 57.28; H, 3.33; N, 2.57. Found: C, 56.89; H, 3.13; N, 2.50.

^{1}H NMR (600 MHz, 299 K, CD_2Cl_2): δ ^{1}H: 8.67 (m, 2H, o-Py), 8.11 (m, 1H, p-Py), 7.65 (m, 2H, m-Py), 5.77 (ddd, $^3J_{HH}$ = 17.2, 10.2, 7.6 Hz, 1H, 2-CH=), [5.06 (ddd, $^3J_{HH}$ = 10.2 Hz, $^2J_{HH}$ = 1.1 Hz, $^4J_{HH}$ = 0.8 Hz), 4.93 (ddd, $^3J_{HH}$ = 17.2 Hz, $^4J_{HH}$ = 2.1 Hz, $^2J_{HH}$ = 1.1 Hz)](each 1H, CH_2=), 4.75 (t, J_{HH} = 3.3 Hz, 1H, 6-CH=), 2.50/1.92 (each d, $^2J_{HH}$ = 14.3 Hz, each 1H, BCH_2), 2.17 (m, 1H, CH), 1.79/1.58 (each m, each 1H, 7-CH_2), 1.43/1.30 (each m, each 1H, 8-CH_2), 1.40/1.34 (each m, each 1H, 9-CH_2).

^{13}C{^{1}H} NMR (151 MHz, 299 K, CD_2Cl_2): δ ^{13}C: 146.4 (o-Py), 142.8 (2-CH=), 142.0 (p-Py), 139.5 (C=), 125.9 (m-Py), 122.5 (6-CH=), 114.8 (CH_2=), 43.5 (CH), 31.4 (br, BCH_2), 29.9 (9-CH_2), 26.1 (7-CH_2), 18.2 (8-CH_2), [C_6F_5 not listed].

^{19}F NMR (564 MHz, 299 K, CD_2Cl_2): δ ^{19}F: –130.9/–131.0 (each m, each 2F, *o*), –159.4/ –159.7 (each t, $^3J_{FF}$ = 20.3 Hz, each 1F, *p*), –164.9/–165.1 (m, 4F, *m*)(C_6F_5).

^{11}B{^{1}H} NMR (192 MHz, 299 K, CD_2Cl_2): δ ^{11}B: –0.6 ($\nu_{1/2}$ ~ 150 Hz).

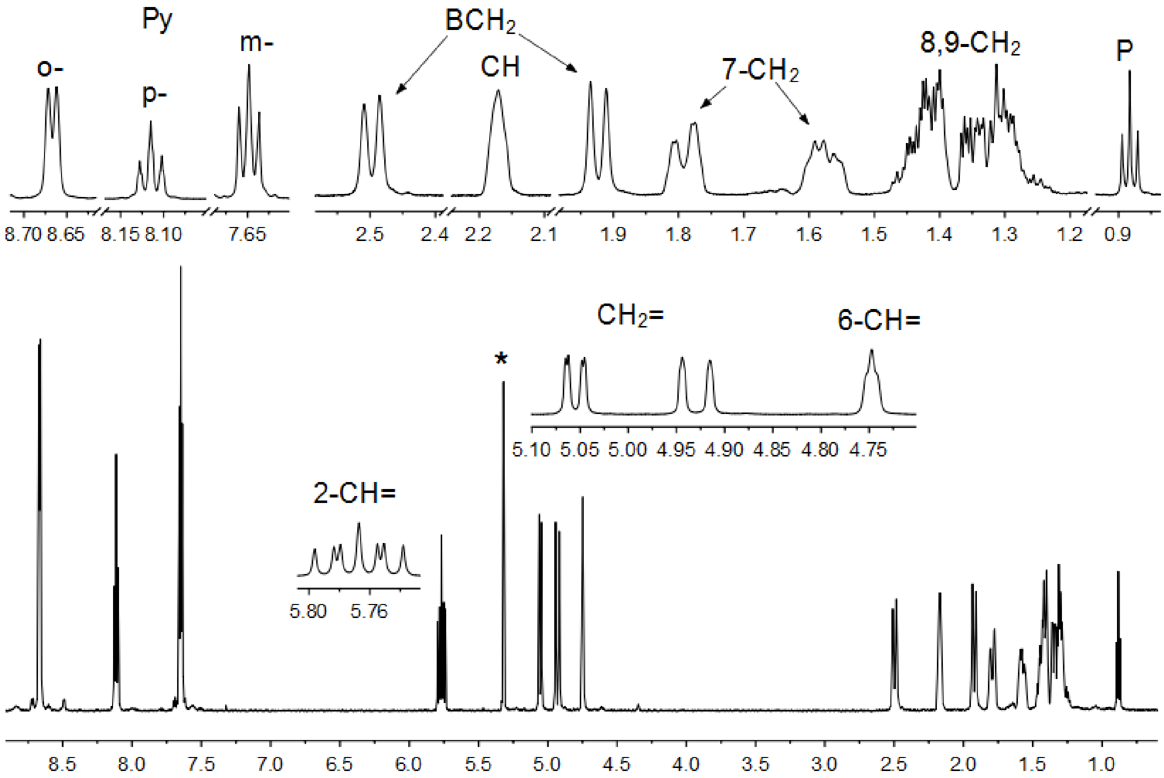

Figure S14. ^{1}H NMR (600 MHz, 299 K, CD_2Cl_2*) spectrum of compound **6a**. [P: pentane]

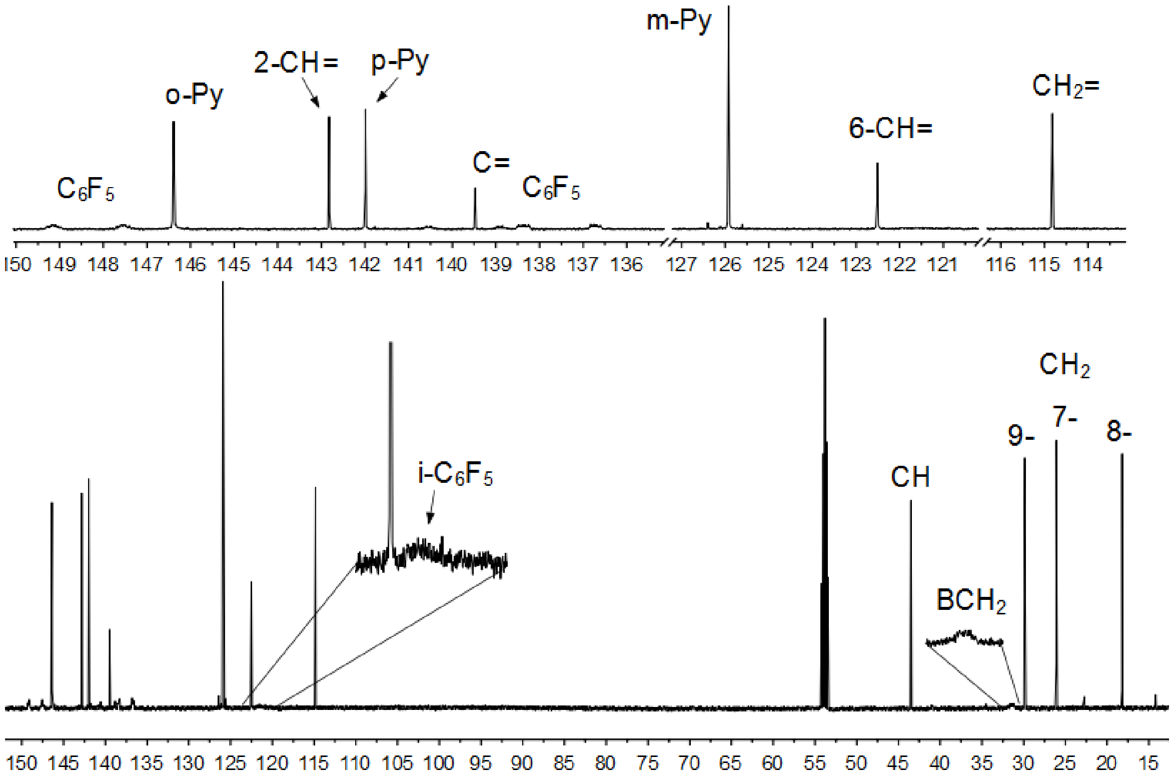

Figure S15. ^{13}C{^{1}H} NMR (151 MHz, 299 K, CD_2Cl_2) spectrum of compound **6a**.

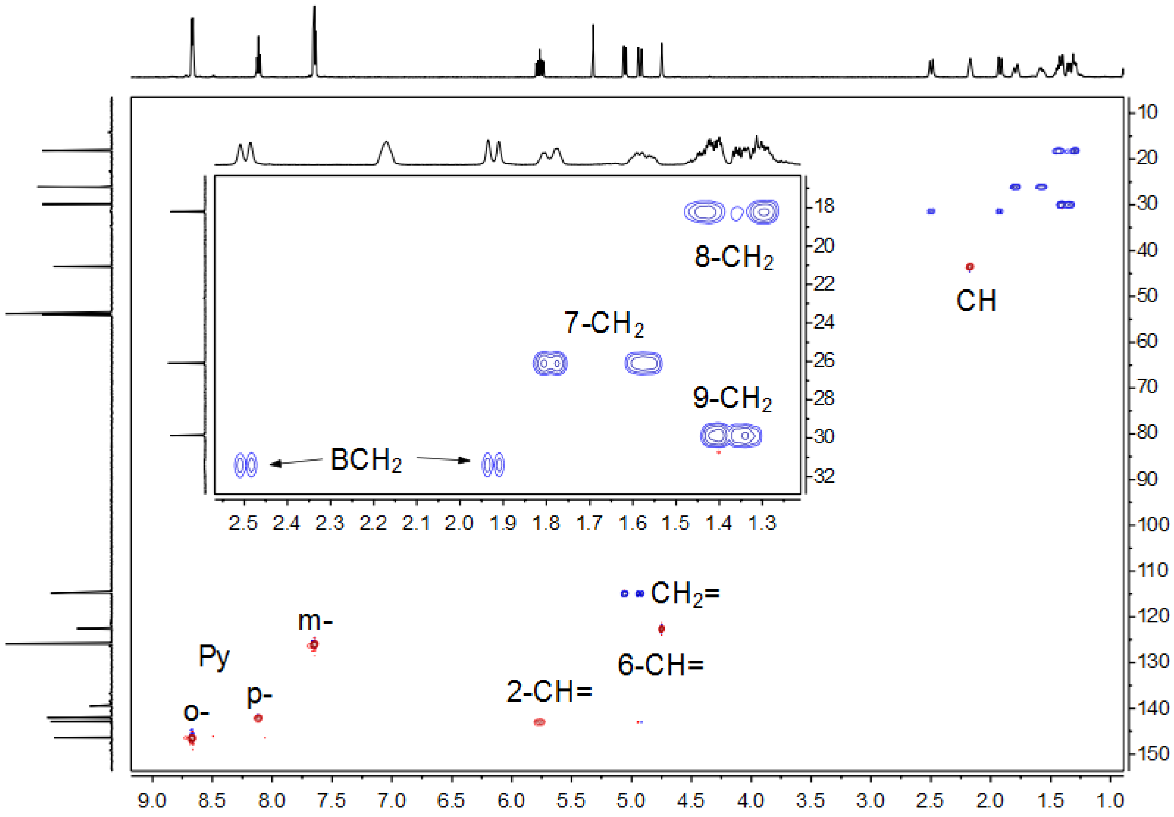

Figure S16. $^{1}H,^{13}C$ GHSQC (600/151 MHz, CD_2Cl_2, 299K) spectrum of compound **6a**.

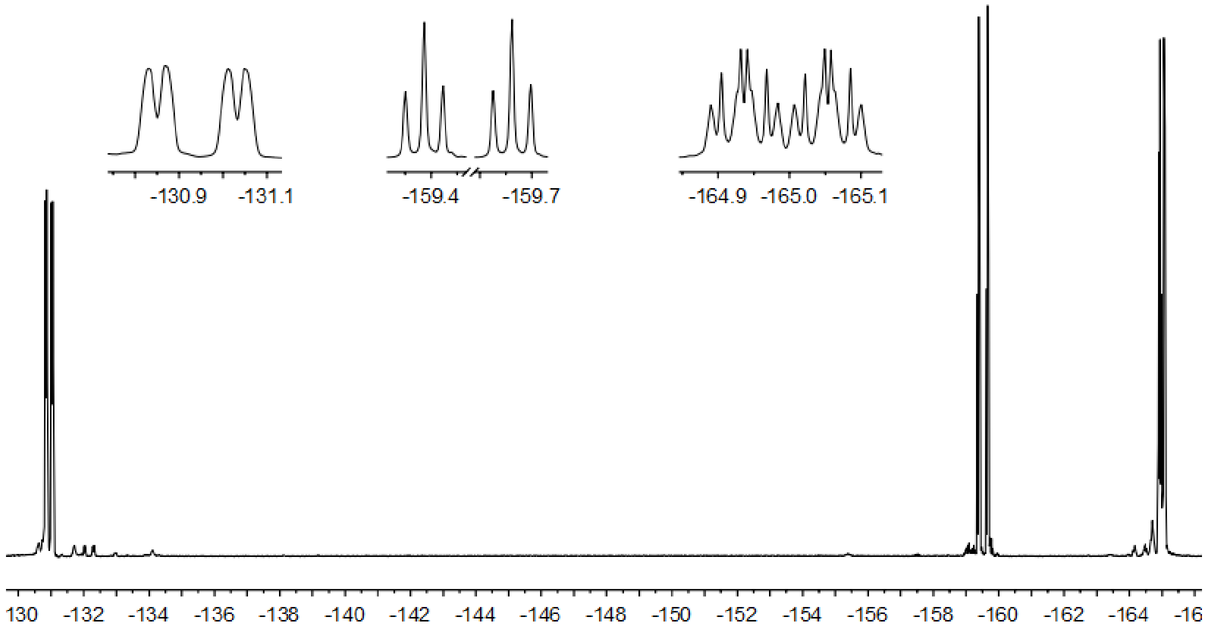

Figure S17. ^{19}F NMR (564 MHz, 299 K, CD_2Cl_2) spectrum of compound **6a**.

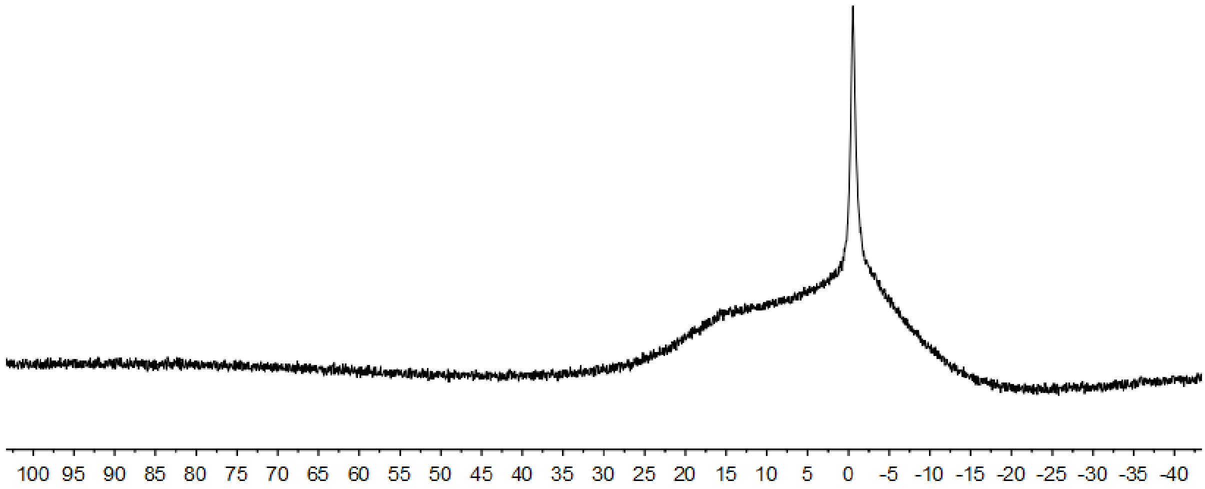

Figure S18. $^{11}B\{^{1}H\}$ NMR (192 MHz, 299 K, CD_2Cl_2) spectrum of compound **6a**.

X-ray crystal structure analysis of compound 6a (erk9382): A colorless prism-like specimen of $C_{26}H_{18}BF_{10}N$, approximate dimensions 0.116 mm x 0.197 mm x 0.220 mm, was used for the X-ray crystallographic analysis. The X-ray intensity data were measured. A total of 1226 frames were collected. The total exposure time was 21.50 hours. The frames were integrated with the Bruker SAINT software package using a wide-frame algorithm. The integration of the data using a monoclinic unit cell yielded a total of 36701 reflections to a maximum θ angle of 68.36° (0.83 Å resolution), of which 4296 were independent (average redundancy 8.543, completeness = 99.9%, R_{int} = 5.68%, R_{sig} = 2.89%) and 3652 (85.01%) were greater than 2σ(F^2). The final cell constants of a = 16.8585(5) Å, b = 9.6751(3) Å, c = 15.4623(5) Å, β = 111.8320(10)°, volume = 2341.14(13) $Å^3$, are based upon the refinement of the XYZ-centroids of 9950 reflections above 20 σ(I) with 5.647° < 2θ < 136.7°. Data were corrected for absorption effects using the multi-scan method (SADABS). The ratio of minimum to maximum apparent transmission was 0.921. The calculated minimum and maximum transmission coefficients (based on crystal size) are 0.7650 and 0.8650. The structure was solved and refined using the Bruker SHELXTL Software Package, using the space group $P2_1/c$, with Z = 4 for the formula unit, $C_{26}H_{18}BF_{10}N$. The final anisotropic full-matrix least-squares refinement on F^2 with 343 variables converged at R1 = 4.50%, for the observed data and wR2 = 10.24% for all data. The goodness-of-fit was 1.099. The largest peak in the final difference electron density synthesis was 0.300 $e^-/Å^3$ and the largest hole was -0.236 $e^-/Å^3$ with an RMS deviation of 0.050 $e^-/Å^3$. On the basis of the final model, the calculated density was 1.547 g/cm^3 and F(000), 1104 e^-. CCDC number: 1922906.

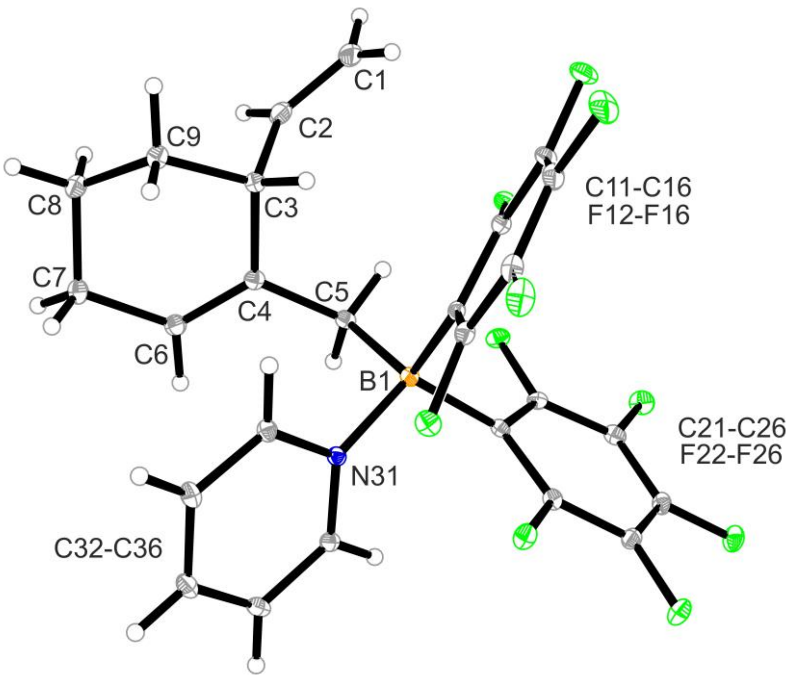

Figure S19. Crystal structure of compound **6a** (thermal ellipsoids: 15% probability).

E) Generation of compound **5b**

Scheme S7.

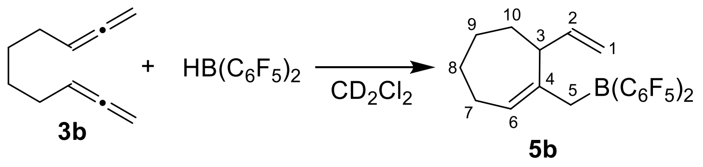

A suspension of $HB(C_6F_5)_2$ (34.6 mg, 0.10 mmol) in CD_2Cl_2 (0.5 mL) was added to a solution of bisallene **3b** (13.4 mg, 0.10 mmol) in CD_2Cl_2 (0.5 mL) at room temperature. Subsequently, the resulting reaction mixture was characterized by NMR experiments.

NMR data of compound **5b** from the reaction mixture:

^{1}H NMR (600 MHz, 299 K, CD_2Cl_2): δ ^{1}H: 5.76 (ddd, $^3J_{HH}$ = 7.6, 10.2, 17.2 Hz, 1H, 2-CH=), 5.64 (t, $^3J_{HH}$ = 6.3 Hz, 1H, 6-CH=), 5.00/4.96 (each dm, $^3J_{HH}$ = 10.2, 17.2 Hz, each 1H, CH_2=), 2.95/2.86 (each d, $^2J_{HH}$ = 16.5 Hz, each 1H, BCH_2), 2.74 (m, 1H, CH), 2.05 (m, 2H, 7-CH_2), 1.65/1.56 (each m, each 1H, 9-CH_2), 1.52/1.30 (each m, each 1H, 8-CH_2), 1.52/1.34 (each m, each 1H, 10-CH_2).

^{13}C{^{1}H} NMR (151 MHz, 299 K, CD_2Cl_2): δ ^{13}C: 147.2 (dm, $^1J_{FC}$ ~ 250 Hz, C_6F_5), 143.6 (dm, $^1J_{FC}$ ~ 260 Hz, C_6F_5), 140.7 (C=), 140.0 (2-CH=), 137.8 (dm, $^1J_{FC}$ ~ 250 Hz, C_6F_5), 131.0 (6-CH=), 114.7 (br, i-C_6F_5), 115.4 (CH_2=), 51.2 (CH), 42.5 (br, BCH_2), 32.4 (10-CH_2), 28.2 (7-CH_2), 27.7 (8-CH_2), 27.0 (9-CH_2).

^{19}F NMR (564 MHz, 299 K, CD_2Cl_2): δ ^{19}F: –129.3 (m, 2F, *o*), –149.2 (tt, $^3J_{FF}$ = 20.0 Hz, $^4J_{FF}$ = 4.3 Hz, 1F, *p*), –162.2 (m, 2F, *m*)(C_6F_5)[$\Delta\delta^{19}F_{m,p}$ = 13.0].

^{11}B{^{1}H} NMR (192 MHz, 299 K, CD_2Cl_2): δ ^{11}B: 70.4 ($\nu_{1/2}$ ~ 700 Hz).

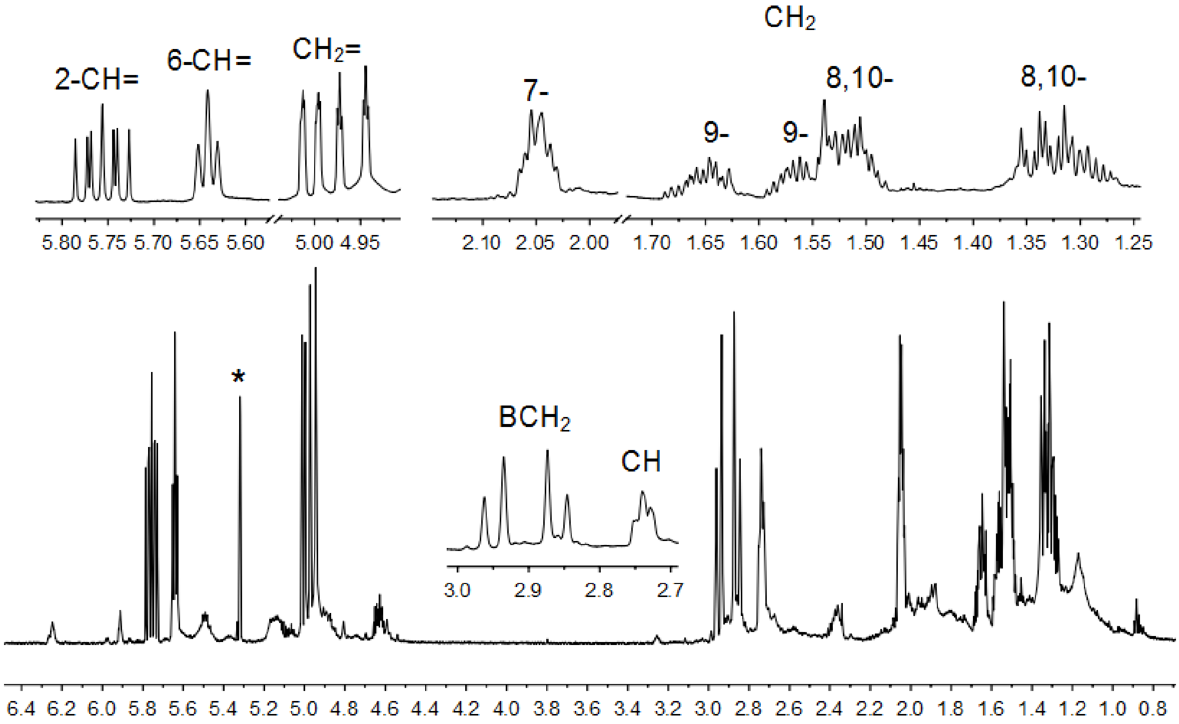

Figure S20. ^{1}H NMR (600 MHz, 299 K, CD_2Cl_2) spectrum of the reaction mixture.

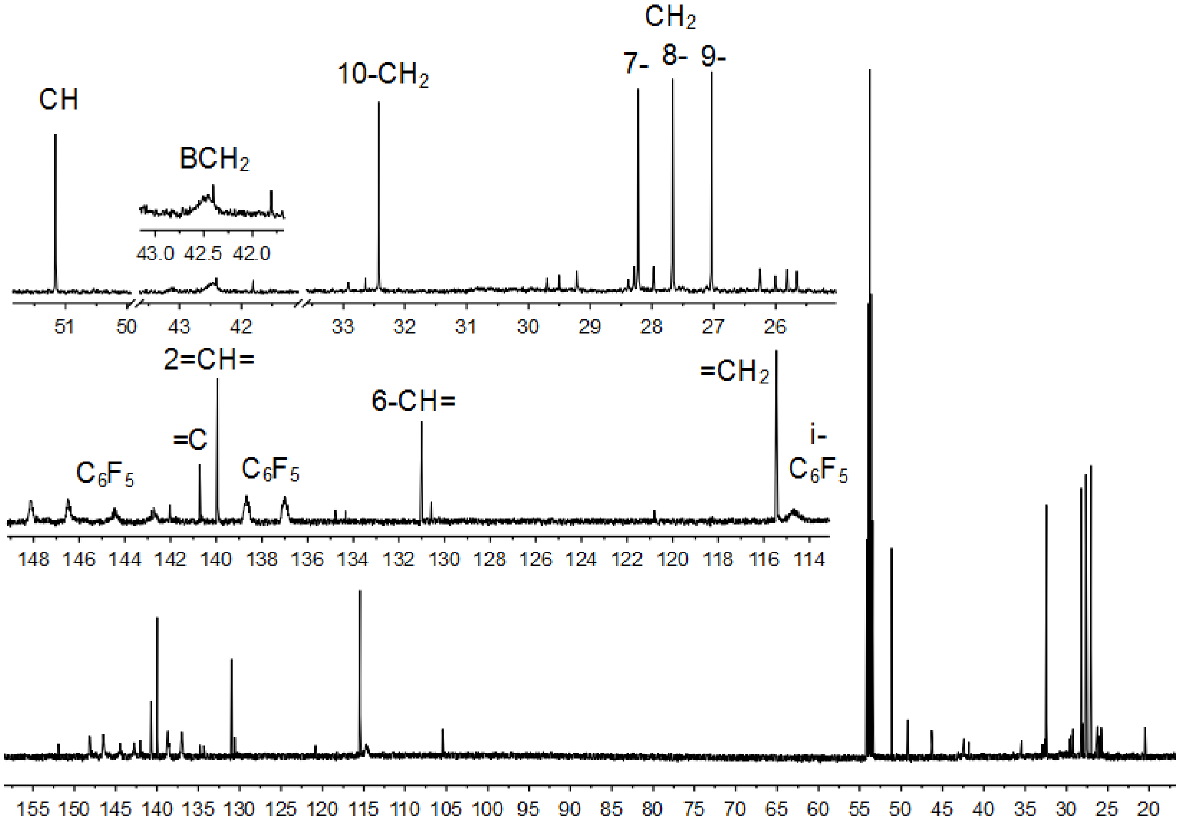

Figure S21. ^{13}C{^{1}H} NMR (151 MHz, 299 K, CD_2Cl_2) spectrum of the reaction mixture.

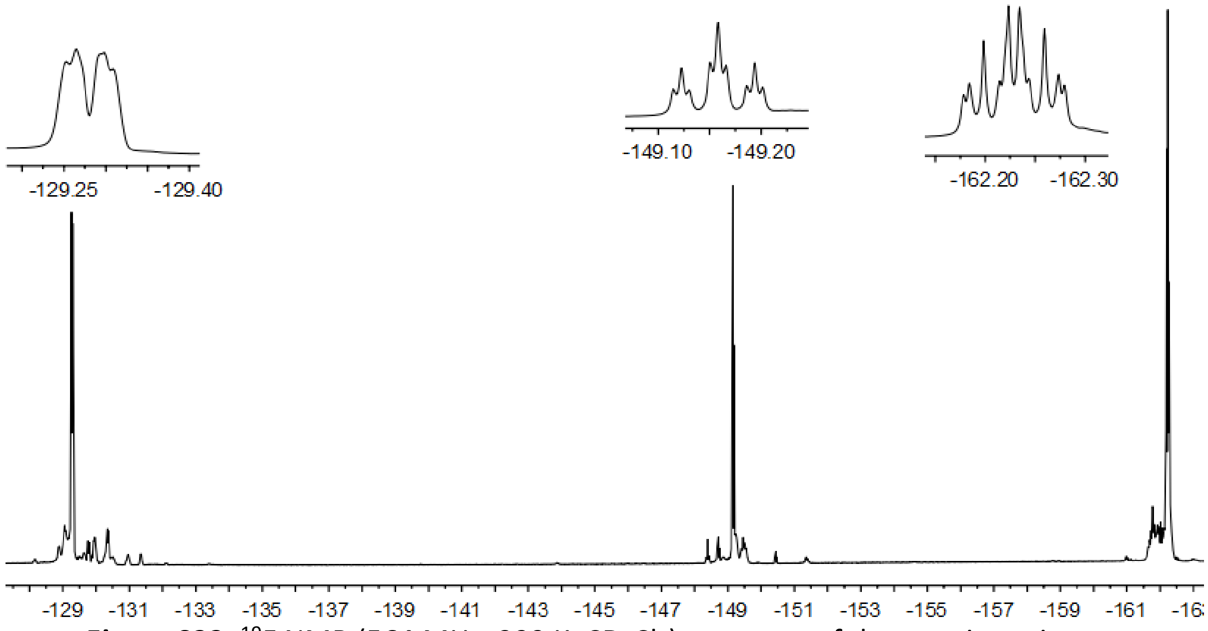

Figure S22. ^{19}F NMR (564 MHz, 299 K, CD_2Cl_2) spectrum of the reaction mixture.

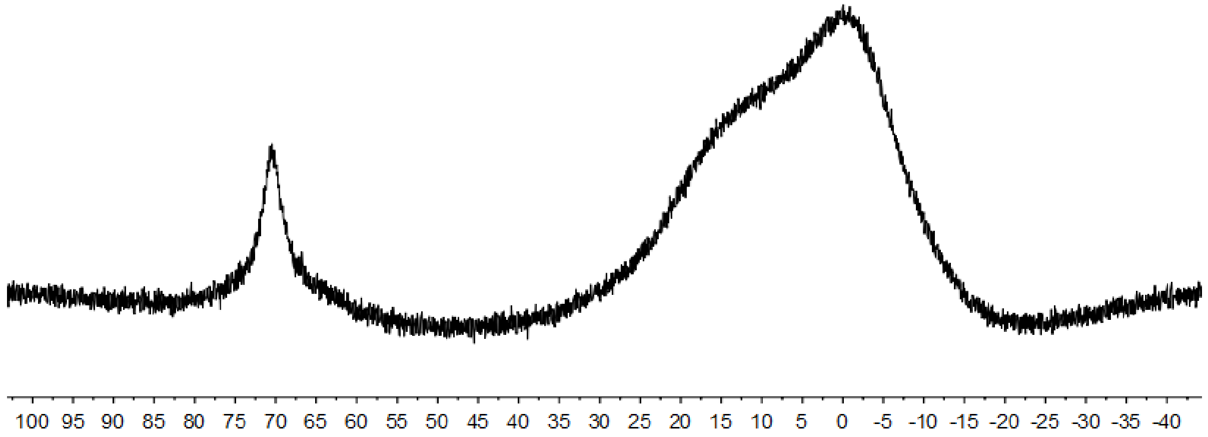

Figure S23. $^{11}B\{^{1}H\}$ NMR (192 MHz, 299 K, CD_2Cl_2) spectrum of the reaction mixture.

F) Synthesis of compound **6b**

Scheme S8.

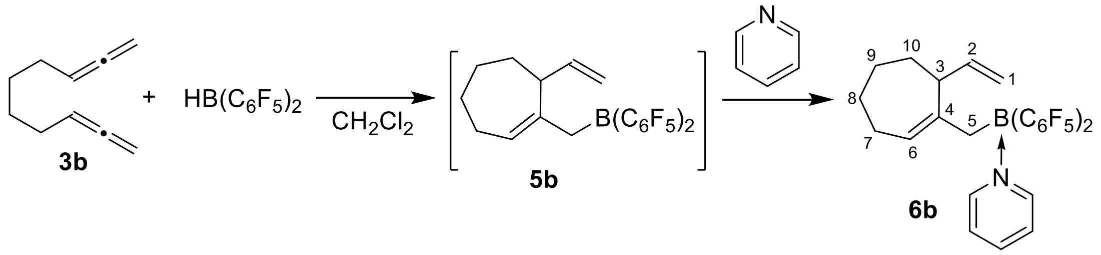

A suspension of $HB(C_6F_5)_2$ (103.8 mg, 0.30 mmol) in CH_2Cl_2 (2 mL) was added to a solution of bisallene **3b** (40.0 mg, 0.30 mmol) in CH_2Cl_2 (0.5 mL) at room temperature. Subsequently, pyridine

(24.0 mg, 0.30 mmol) was added to the reaction mixture. Then all volatiles were removed in vacuo and the residue was dissolved in a solvent mixture of pentane (5 mL) and CH_2Cl_2 (0.5 mL). The resulting solution was then stored at –35 °C for 1h. The sticky precipitate was removed by decantation. Slow evaporation of the remaining solution at room temperature gave a colorless crystalline material. Part of the crystals were used for the X-ray structure analysis, the rest was carefully washed with pentane (1 mL × 3) and dried in vacuo giving compound **6b** (65 mg, 0.12 mmol, 39%) as a white crystalline material.

Anal. Calc. for $C_{27}H_{20}BF_{10}N$: C, 57.99; H, 3.60; N, 2.50. Found: C, 58.20; H, 3.55; N, 2.55.

NMR data of compound **6b**:

^{1}H NMR (600 MHz, 299 K, CD_2Cl_2): δ ^{1}H: 8.63 (m, 2H, o-Py), 8.11 (m, 1H, p-Py), 7.65 (m, 2H, m-Py), 5.82 (ddd, $^3J_{HH}$ = 17.1, 10.2, 7.0 Hz, 1H, 2-CH=), [5.00 (ddd, $^3J_{HH}$ = 10.2 Hz, $^2J_{HH}$ = 2.2 Hz, $^4J_{HH}$ = 1.3 Hz), 4.96 (ddd, $^3J_{HH}$ = 17.1 Hz, $^2J_{HH}$ = 2.2 Hz, $^4J_{HH}$ = 1.6 Hz)](each 1H, CH_2=), 4.85 (dd, $^3J_{HH}$ = 7.4, 5.2 Hz, 1H, 6-CH=), 2.65 (m, 1H, CH), 2.49/2.04 (each d, $^2J_{HH}$ = 14.6 Hz, each 1H, BCH_2), 1.86/1.77 (each m, each 1H, 7-CH_2), 1.53 (m, 2H, 9-CH_2), 1.52/1.10 (each m, each 1H, 8-CH_2), 1.50/1.25 (each m, each 1H, 10-CH_2).

^{13}C{^{1}H} NMR (151 MHz, 299 K, CD_2Cl_2): δ ^{13}C: 146.4 (o-Py), 145.1 (C=), 141.9 (p-Py), 140.1 (2-CH=), 126.5 (6-CH=), 125.8 (m-Py), 114.1 (CH_2=), 50.1 (CH), 35.3 (br, BCH_2), 32.4 (10-CH_2), 28.2 (7-CH_2), 28.0 (8-CH_2), 26.3 (9-CH_2), [C_6F_5 not listed].

^{19}F NMR (564 MHz, 299 K, CD_2Cl_2): δ ^{19}F: –130.4/–130.7 (each m, each 2F, *o*), –159.1/ –159.3 (each t, $^3J_{FF}$ = 20.3 Hz, each 1F, *p*), –164.6/-164.7 (each m, each 2F, *m*)(C_6F_5)[$\Delta\delta^{19}F_{m,p}$ = 5.5, 5.3].

^{11}B{^{1}H} NMR (192 MHz, 299 K, CD_2Cl_2): δ ^{11}B: –0.6 ($\nu_{1/2}$ ~ 150 Hz).

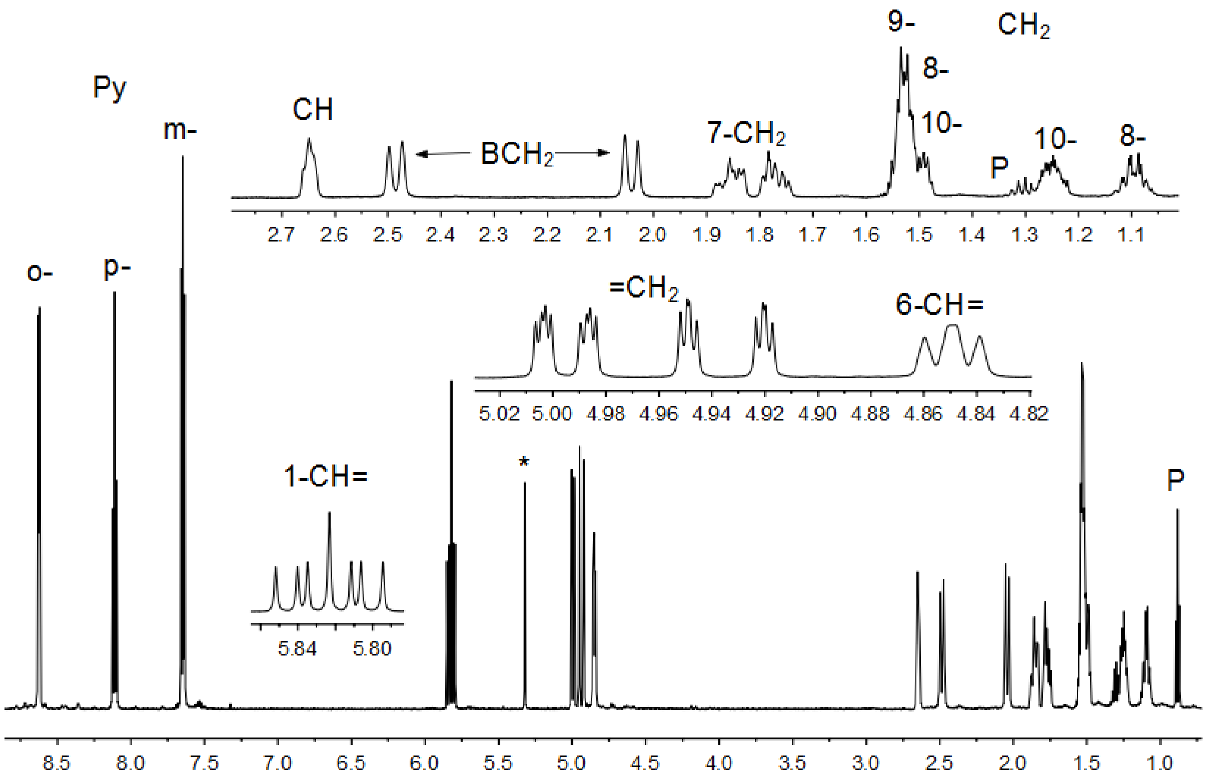

Figure S24. ^{1}H NMR (600 MHz, 299 K, CD_2Cl_2*) spectrum of compound **6b**.

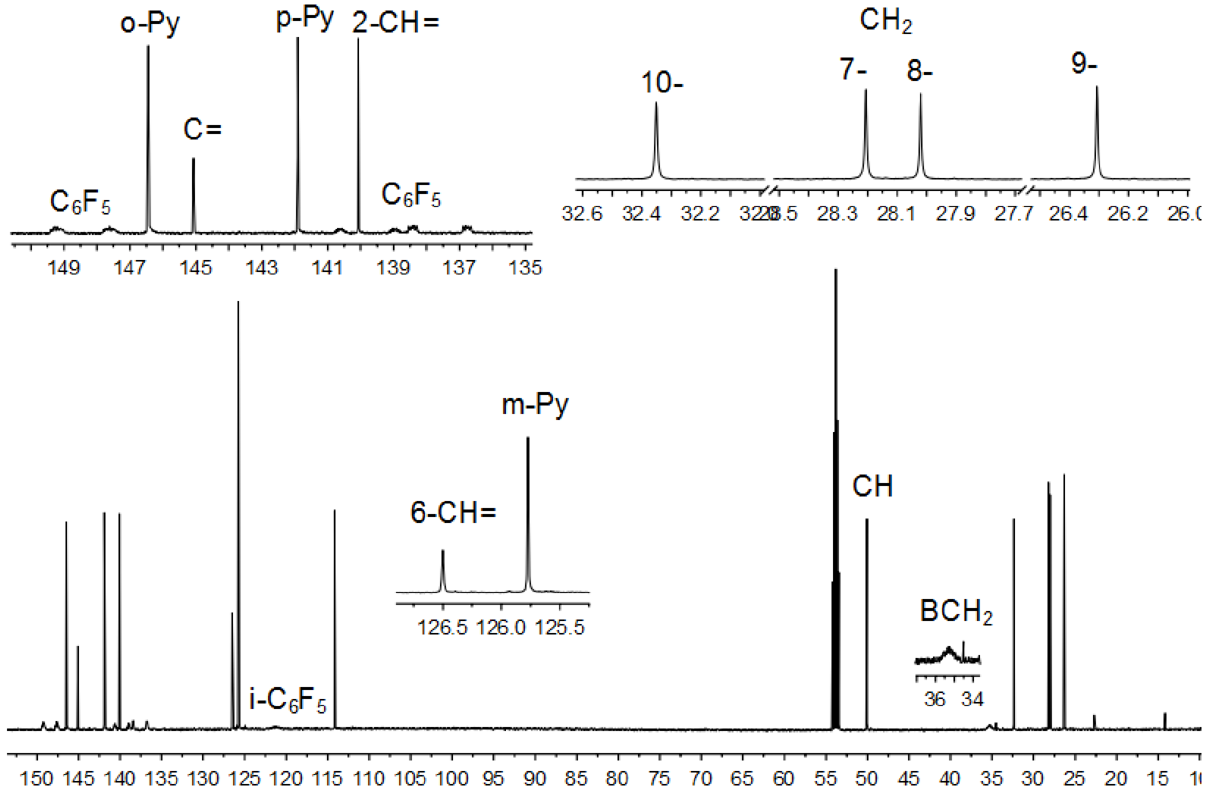

Figure S25. $^{13}C\{^1H\}$ NMR (151 MHz, 299 K, CD_2Cl_2) spectrum of compound **6b**.

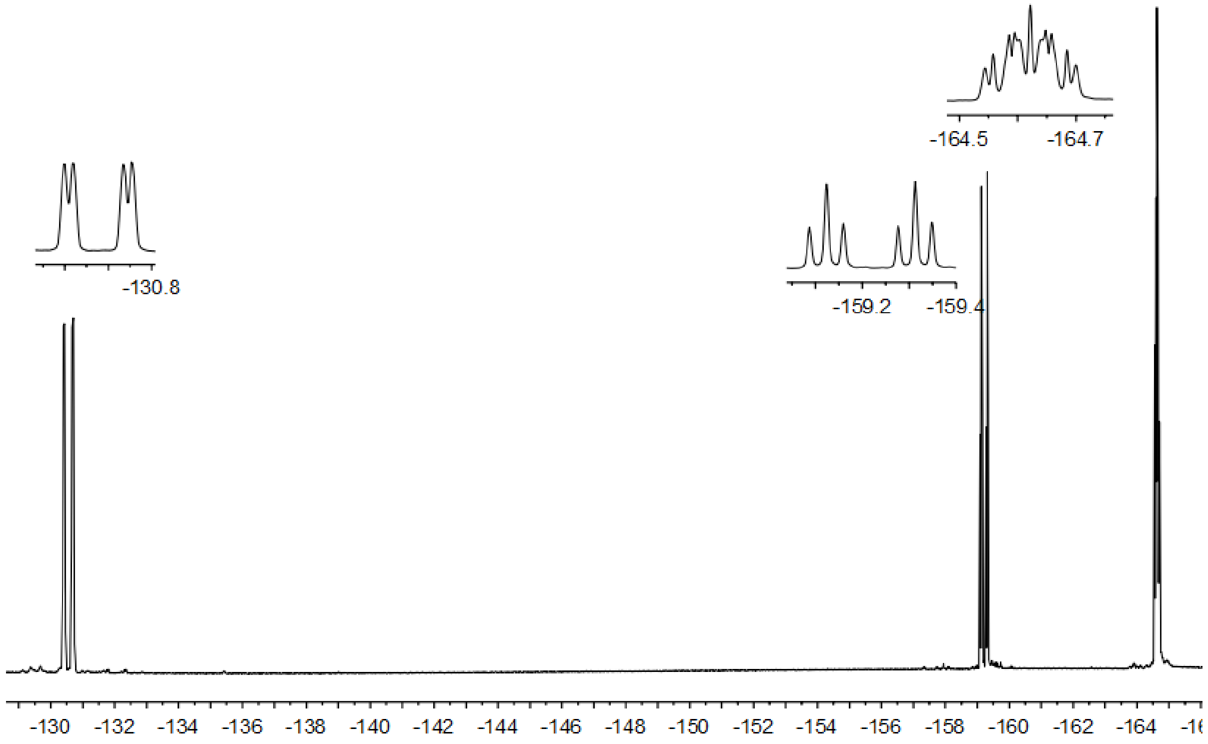

Figure S26. ^{19}F NMR (564 MHz, 299 K, CD_2Cl_2) spectrum of compound **6b**.

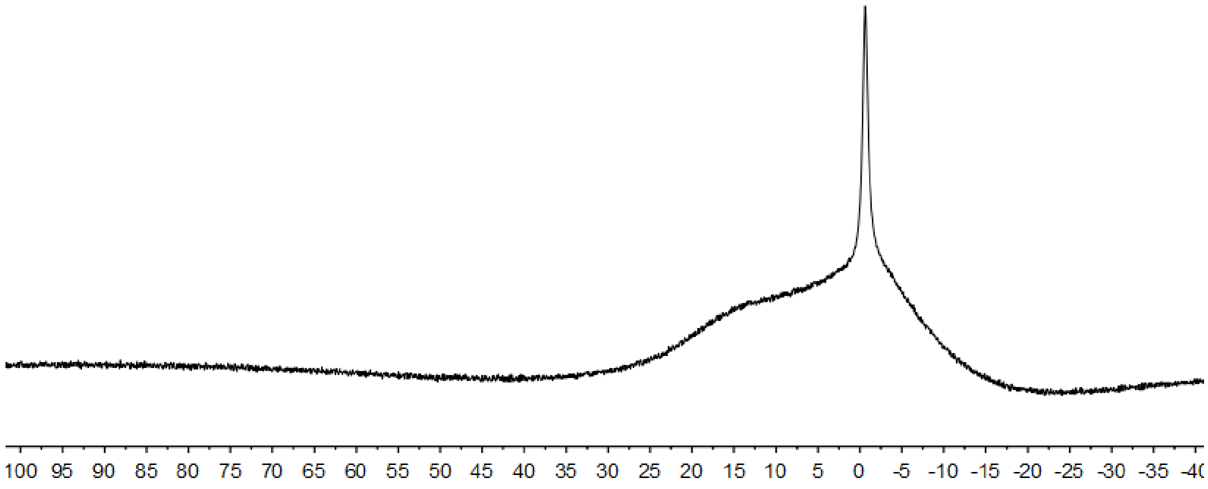

Figure S27. $^{11}B\{^1H\}$ NMR (192 MHz, 299 K, CD_2Cl_2) spectrum of compound **6b**.

X-ray crystal structure analysis of compound 6b (erk9368): A colorless prism-like specimen of $C_{27}H_{20}BF_{10}N$, approximate dimensions 0.087 mm x 0.119 mm x 0.173 mm, was used for the X-ray crystallographic analysis. The X-ray intensity data were measured. A total of 972 frames were collected. The total exposure time was 21.27 hours. The frames were integrated with the Bruker SAINT software package using a wide-frame algorithm. The integration of the data using an orthorhombic unit cell yielded a total of 22711 reflections to a maximum θ angle of 68.38° (0.83 Å

resolution), of which 4353 were independent (average redundancy 5.217, completeness = 99.2%, R_{int} = 7.27%, R_{sig} = 4.96%) and 3722 (85.50%) were greater than 2σ(F^2). The final cell constants of a = 9.7240(3) Å, b = 14.9525(4) Å, c = 16.4897(5) Å, volume = 2397.57(12) $Å^3$, are based upon the refinement of the XYZ-centroids of 9961 reflections above 20 σ(I) with 7.981° < 2θ < 136.5°. Data were corrected for absorption effects using the multi-scan method (SADABS). The ratio of minimum to maximum apparent transmission was 0.888. The calculated minimum and maximum transmission coefficients (based on crystal size) are 0.8100 and 0.8970. The structure was solved and refined using the Bruker SHELXTL Software Package, using the space group $P2_12_12_1$, with Z = 4 for the formula unit, $C_{27}H_{20}BF_{10}N$. The final anisotropic full-matrix least-squares refinement on F^2 with 380 variables converged at R1 = 4.57%, for the observed data and wR2 = 9.46% for all data. The goodness-of-fit was 1.060. The largest peak in the final difference electron density synthesis was 0.273 $e^-/Å^3$ and the largest hole was -0.219 $e^-/Å^3$ with an RMS deviation of 0.050 $e^-/Å^3$. On the basis of the final model, the calculated density was 1.549 g/cm^3 and F(000), 1136 e^-. CCDC number: 1922907.

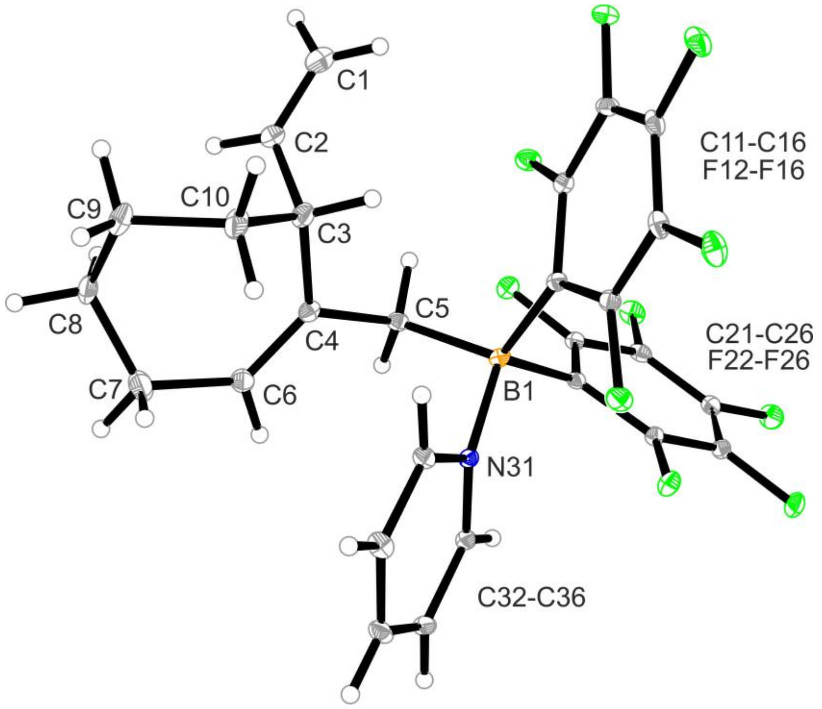

Figure S28. Crystal structure of compound **6b** (thermal ellipsoids: 15% probability).

G) Generation of compound **5c**

Scheme S9.

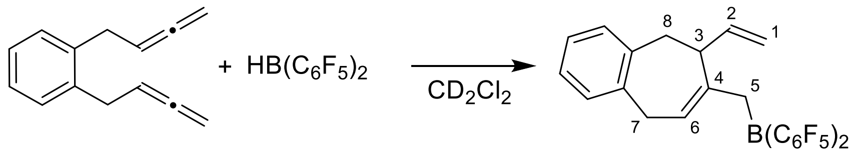

A suspension of $HB(C_6F_5)_2$ (34.6 mg, 0.10 mmol) in CD_2Cl_2 (0.5 mL) was added to a solution of bisallene **3c** (18.2 mg, 0.10 mmol) in CD_2Cl_2 (0.5 mL) at room temperature. Subsequently, the resulting reaction mixture was characterized by NMR experiments.

NMR data of compound **5c** from the reaction mixture:

^{1}H NMR (600 MHz, 299 K, CD_2Cl_2): δ ^{1}H: [7.09 (2H), 7.00 (1H), 6.97 (1H)](each m, $CH^{phenylene}$), 5.72 (br dd, $^3J_{HH}$ = 7.3 Hz, $^3J_{HH}$ = 5.2 Hz, 1H, 6-CH=), 5.48 (ddd, $^3J_{HH}$ = 17.2 Hz, $^3J_{HH}$ = 9.9 Hz, $^3J_{HH}$ = 9.1 Hz, 1H, 2-CH=), [5.13 (dd, $^3J_{HH}$ = 17.2 Hz, $^2J_{HH}$ = 1.6 Hz), 5.08 (dd, $^3J_{HH}$ = 9.9 Hz, $^2J_{HH}$ = 1.6 Hz)](each 1H, H_2C=), [3.49 (dd, $^2J_{HH}$ = 16.1 Hz, $^3J_{HH}$ = 5.2 Hz), 3.14 (dd, $^2J_{HH}$ = 16.1 Hz, $^3J_{HH}$ = 7.3 Hz)](each 1H, 7-CH_2), [2.93 (dd, $^2J_{HH}$ = 13.6 Hz, $^3J_{HH}$ = 9.7 Hz), 2.86 (dd, $^2J_{HH}$ = 13.6 Hz, $^3J_{HH}$ = 4.2 Hz)](each 1H, 8-CH_2), [2.85, 2.75](each d, $^2J_{HH}$ = 17.6 Hz, each 1H, 5-CH_2B), 2.64 (m, 1H, 3-CH).

^{13}C{^{1}H} NMR (151 MHz, 299 K, CD_2Cl_2): δ ^{13}C: [147.0 (dm, $^1J_{FC}$ ~ 240 Hz), 143.3 (dm, $^1J_{FC}$ ~ 250 Hz), 137.7 (dm, $^1J_{FC}$ ~ 250 Hz), 114.3 (br, *i*)](C_6F_5), [142.6, 138.8]($C^{phenylene}$), 141.4 (2-CH=), 138.7 (C=), [129.0, 127.1, 126.5, 126.4]($CH^{phenylene}$)], 125.4 (6-CH=), 117.0 (H_2C=), 50.5 (3-CH), 41.7 (br, 5-CH_2B), 38.4 (8-CH_2), 33.0 (7-CH_2).

^{19}F NMR (564 MHz, 299 K, CD_2Cl_2): δ ^{19}F: –129.5 (m, 2F, *o*), –148.8 (tt, $^3J_{FF}$ = 20.3 Hz, $^4J_{FF}$ = 4.5 Hz, 1F, *p*), –162.0 (m, 2F, *m*)(C_6F_5)[$\Delta\delta^{19}F_{m,p}$ = 13.2].

^{11}B{^{1}H} NMR (192 MHz, 299 K, CD_2Cl_2): δ ^{11}B: 71.9 ($\nu_{1/2}$ ~ 900 Hz).

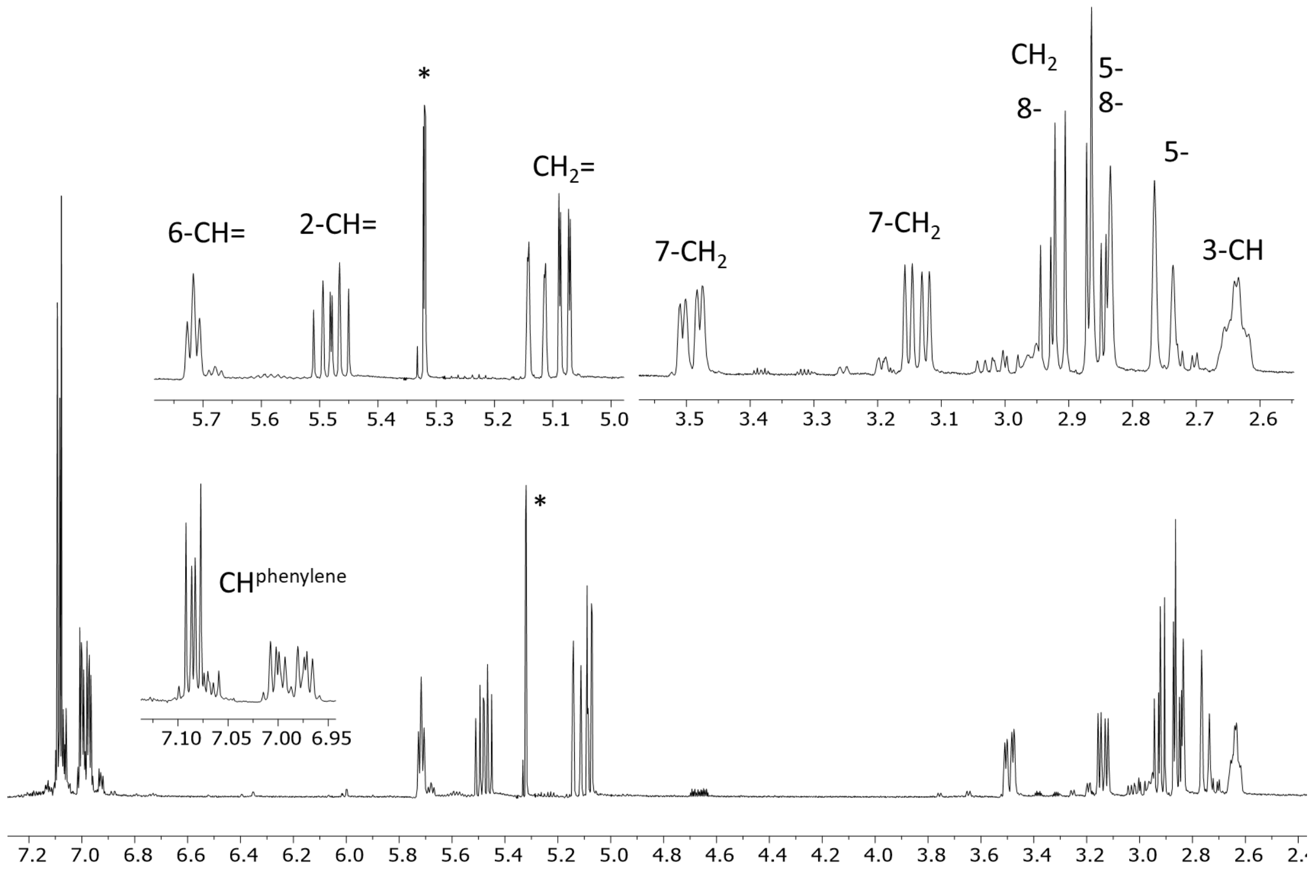

Figure S29. ^{1}H NMR (600 MHz, 299 K, CD_2Cl_2*) spectrum of reaction mixture.

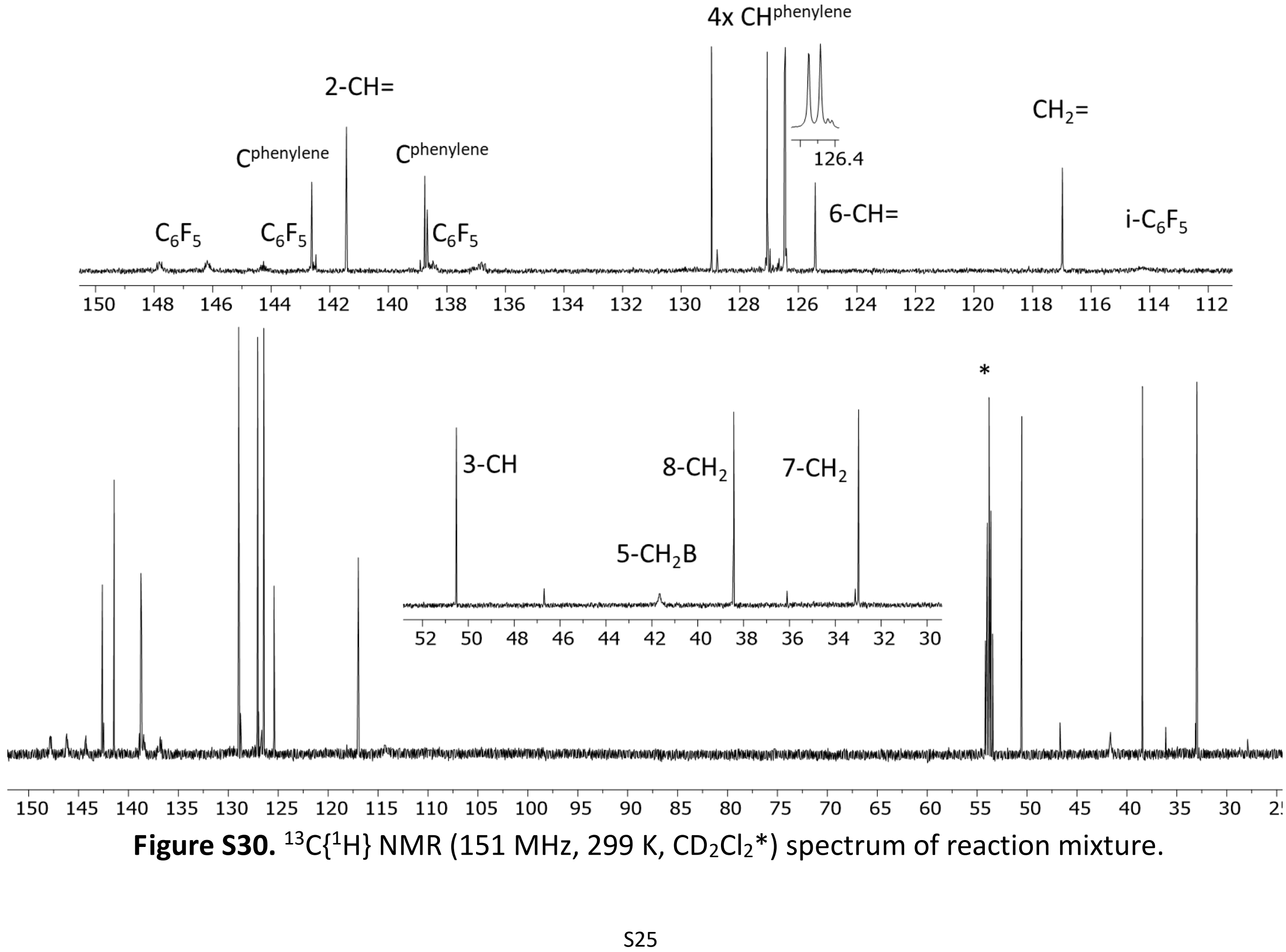

Figure S30. ^{13}C{^{1}H} NMR (151 MHz, 299 K, CD_2Cl_2*) spectrum of reaction mixture.

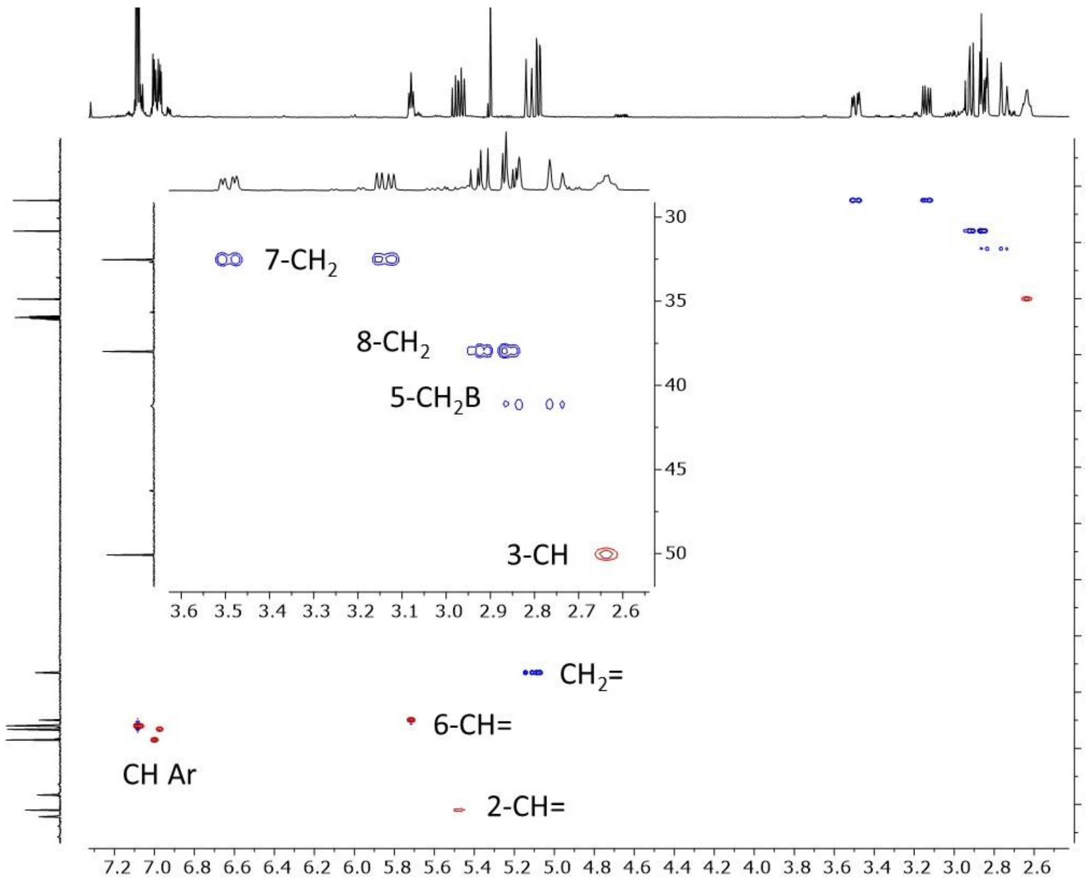

Figure S31. ^{1}H,^{13}C GHSQC (500/151 MHz, CD_2Cl_2, 299K) spectrum of reaction mixture.

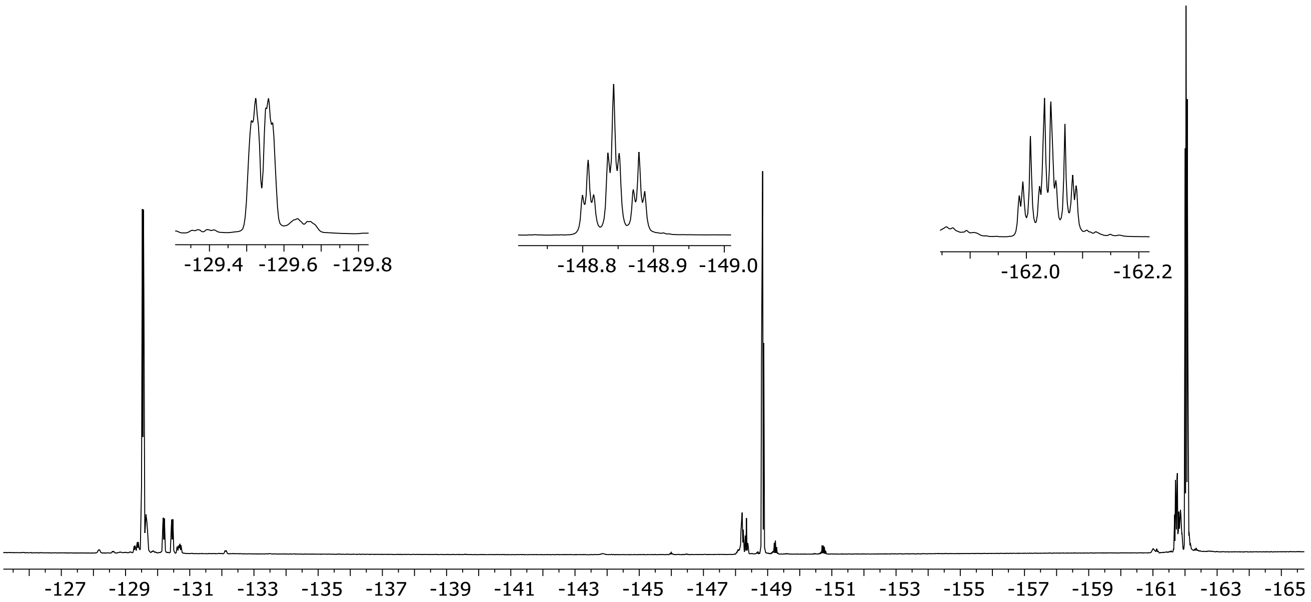

Figure S32. ^{19}F NMR (564 MHz, 299 K, CD_2Cl_2) spectrum of reaction mixture.

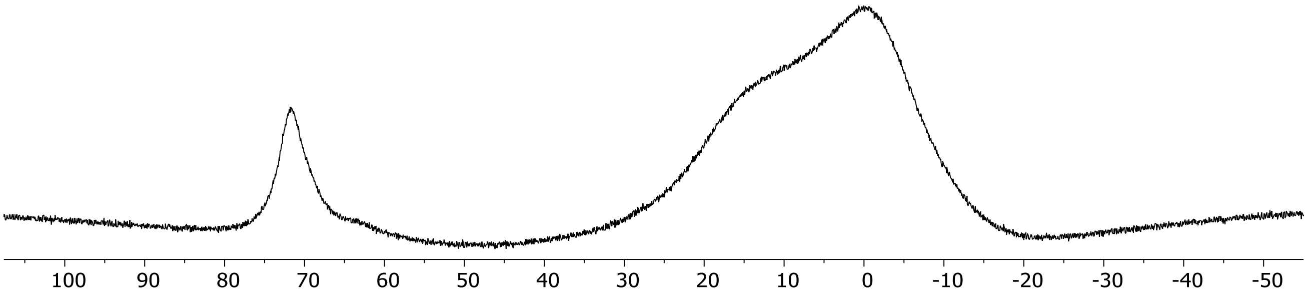

Figure S33. $^{11}B\{^1H\}$ NMR (192 MHz, 299 K, CD_2Cl_2) spectrum of reaction mixture.

H) Synthesis of compound **6c**

Scheme S10

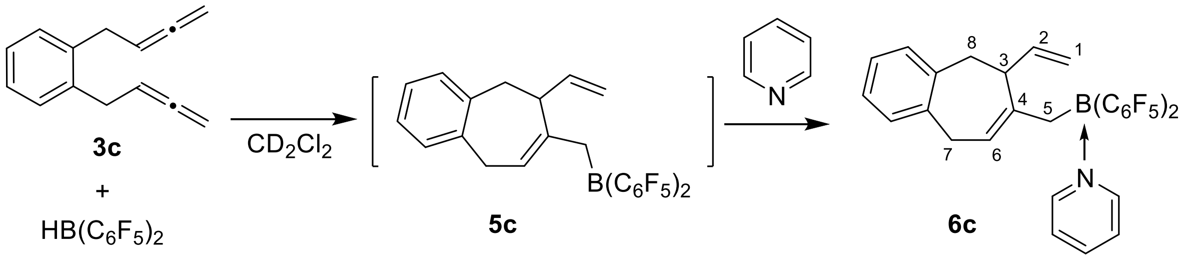

A suspension of $HB(C_6F_5)_2$ (103.8 mg, 0.30 mmol) in CH_2Cl_2 (3 mL) was added to a solution of bisallene **3c** (54.6 mg, 0.30 mmol) in CH_2Cl_2 (2 mL) at room temperature. The resulting mixture was stirred for 30 minutes at room temperature. Subsequently a solution of pyridine (76.8 mg, 0.3 mmol) in CH_2Cl_2 (2 mL) was added. The mixture was stirred for 30 miunutes at r.t. and then all volatilities were removed in vacuo to give a white foamy semisolid. The residue was extracted with pentane (1 mL × 5). The combined extracts were dried in vacuo to give compound **6c** (67.4 mg, 0.11 mmol 37%) as a white solid.

HRMS (ESI) m/z: calc. for $C_{17}H_5BF_{10}N$ $[B(C_6F_5)_2py]^+$: 424.0353 Found: 424.0348

NMR data of compound **6c**

^{1}H NMR (600 MHz, 299 K, CD_2Cl_2): δ ^{1}H: [8.57 (m, 2H, *o*), 8.06 (tt, $^3J_{HH}$ = 7.7 Hz, $^4J_{HH}$ = 1.4 Hz, 1H, *p*), 7.54 (m, 2H, *m*)](py), [7.08 (2H), 6.97 (1H), 6.91 (m)](each m, $CH^{phenylene}$), 5.60 (ddd, $^3J_{HH}$ = 17.2 Hz, $^3J_{HH}$ = 10.1 Hz, $^3J_{HH}$ = 8.2 Hz, 1H, 2-CH=), [5.05 (ddd, $^3J_{HH}$ = 10.1 Hz, $^2J_{HH}$ = 1.9 Hz, $^4J_{HH}$ = 1.0 Hz), 4.96 (ddd, $^3J_{HH}$ = 17.2 Hz, $^2J_{HH}$ = 1.9 Hz, $^4J_{HH}$ = 0.9 Hz)](each 1H, 1-CH_2), 5.03 (t, $^3J_{HH}$ = 6.1 Hz, 6-CH=), [3.22, 3.00](each dd, $^2J_{HH}$ = 16.3 Hz, $^3J_{HH}$ = 6.1 Hz, each 1H, 7-CH_2), [2.85 (dd, $^2J_{HH}$ = 13.5 Hz, $^3J_{HH}$ = 4.1 Hz), 2.73 (dd, $^2J_{HH}$ = 13.5 Hz, $^3J_{HH}$ = 8.6 Hz)](each 1H, 8-CH_2), 2.45 (m, 1H, 3-CH), [2.44, 1.98](each d, $^2J_{HH}$ = 14.8 Hz, each 1H, 5-CH_2).

$^{13}C\{^1H\}$ NMR (151 MHz, 299 K, CD_2Cl_2): δ ^{13}C: [146.3 (*o*), 141.9 (*p*), 125.9 (*p*)](py), 142.8 (2-CH=),

[142.7, 139.3]($C^{phenylene}$), 142.4 (4-C=), [129.3, 127.2, 126.20, 126.17]($CH^{phenylene}$), 121.5 (6-CH=), 115.2 (1-CH_2=), 48.2 (3-CH), 39.5 (8-CH_2), 33.5 (7-CH_2), 33. 1 (br, 5-CH_2), [C_6F_5 not listed].

^{19}F NMR (564 MHz, 299 K, CD_2Cl_2): δ ^{19}F: [–131.06, -131.09](each m, each 2F, *o*-C_6F_5), [–158.7, –159.4](each t, $^3J_{FF}$ = 20.5 Hz, each 1F, *p*-C_6F_5), [–164.5, –164.8](each m, each 2F, *m*-C_6F_5).

^{11}B{^{1}H} NMR (192 MHz, 299 K, CD_2Cl_2): δ ^{11}B: –0.5 ($\nu_{1/2}$ ~ 250 Hz).

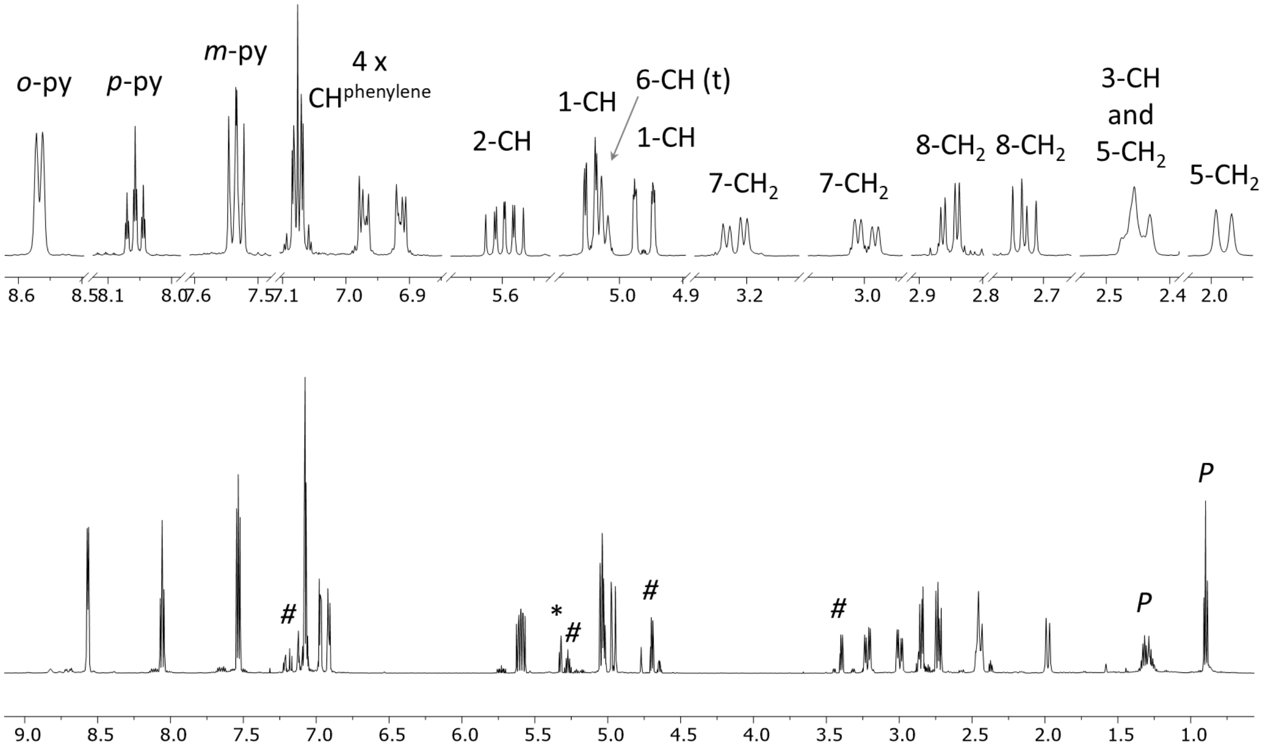

Figure S34. ^{1}H NMR (600 MHz, 299 K, CD_2Cl_2*) spectrum of compound **6c**.
[P: pentane, #: bisallene **3c**].

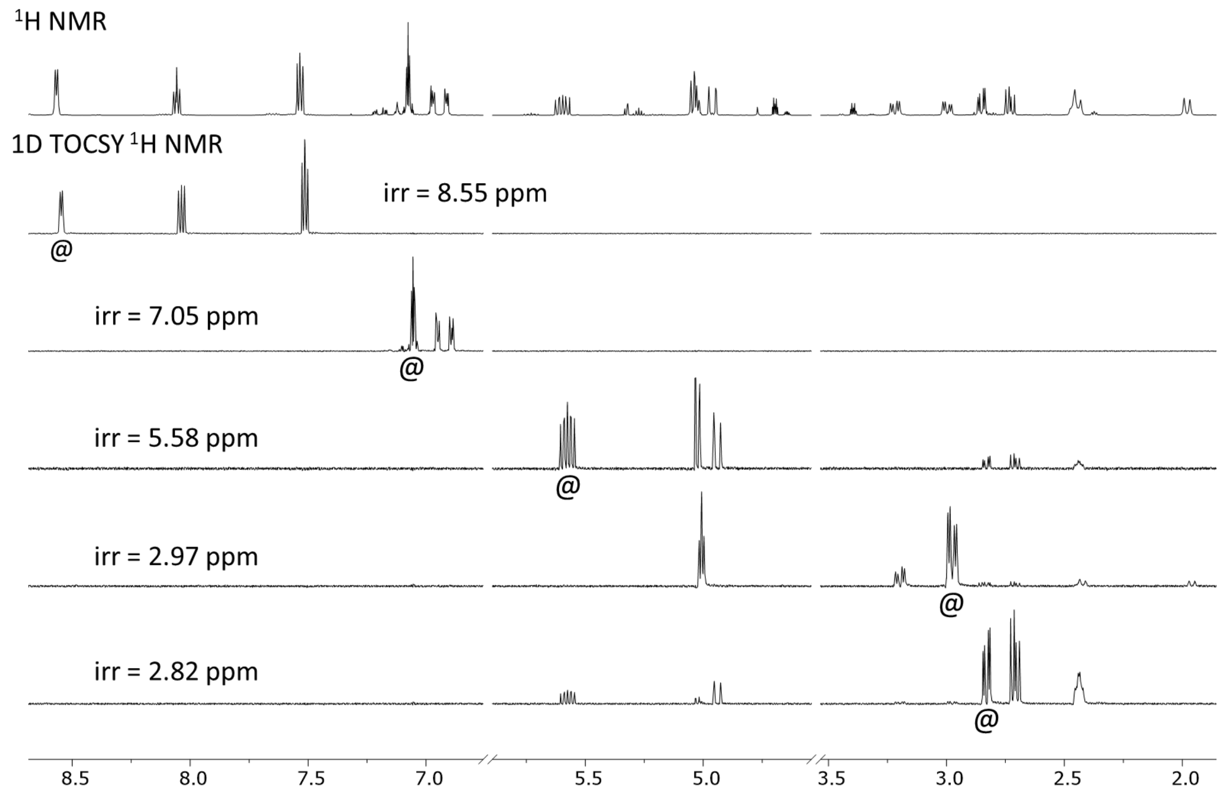

Figure S35. Excerpts from ^{1}H NMR (600 MHz, 299 K, CD_2Cl_2) and 1D TOCSY (600 MHz, 299 K, CD_2Cl_2) spectra of compound **6c** [@: irradiation peaks].

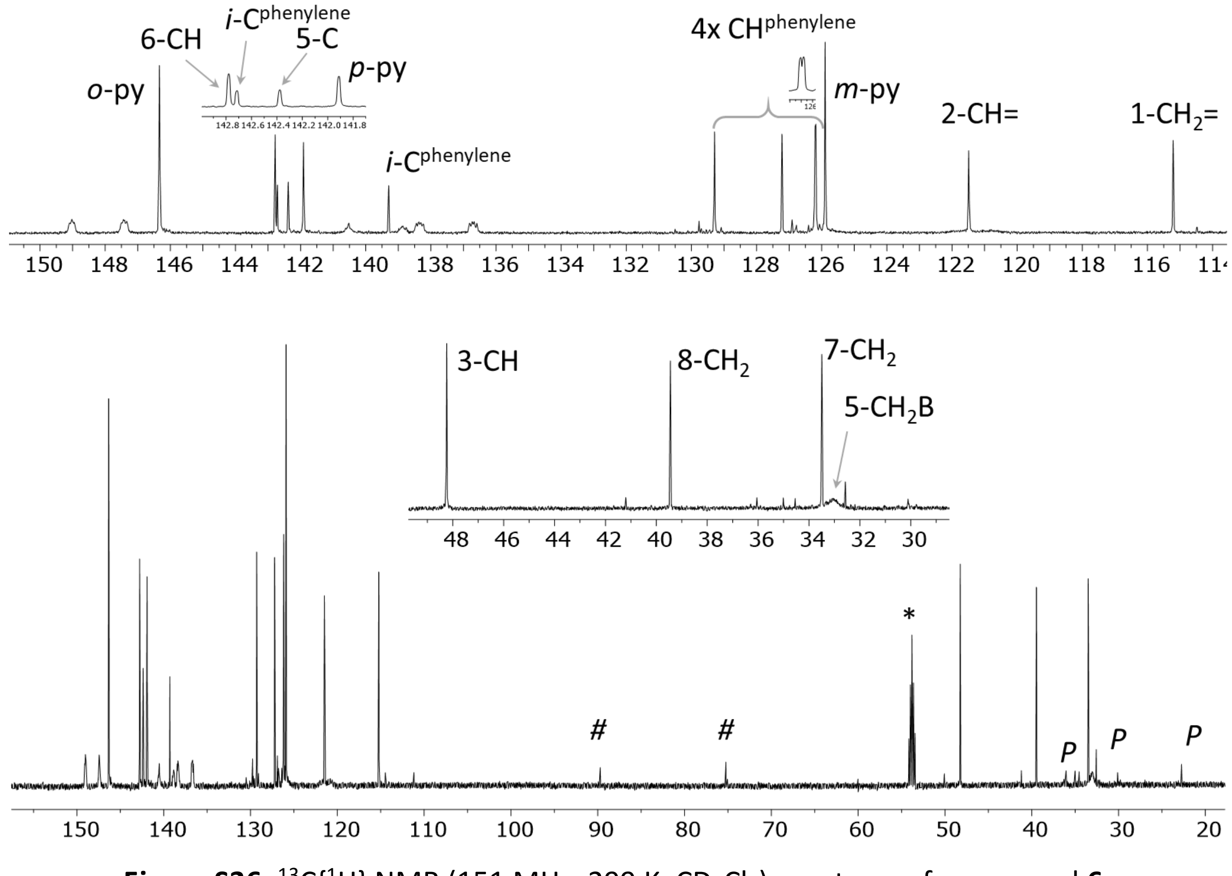

Figure S36. ^{13}C{^{1}H} NMR (151 MHz, 299 K, CD_2Cl_2) spectrum of compound **6c**.
[P: pentane, #: bisallene **3c**]

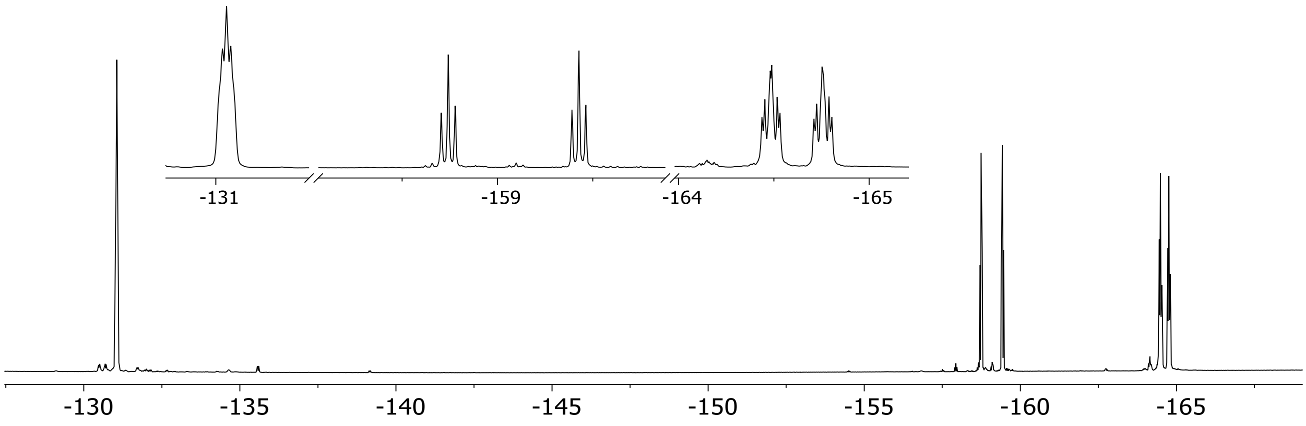

Figure S37. ^{19}F NMR (564 MHz, 299 K, CD_2Cl_2) spectrum of compound **6c**.

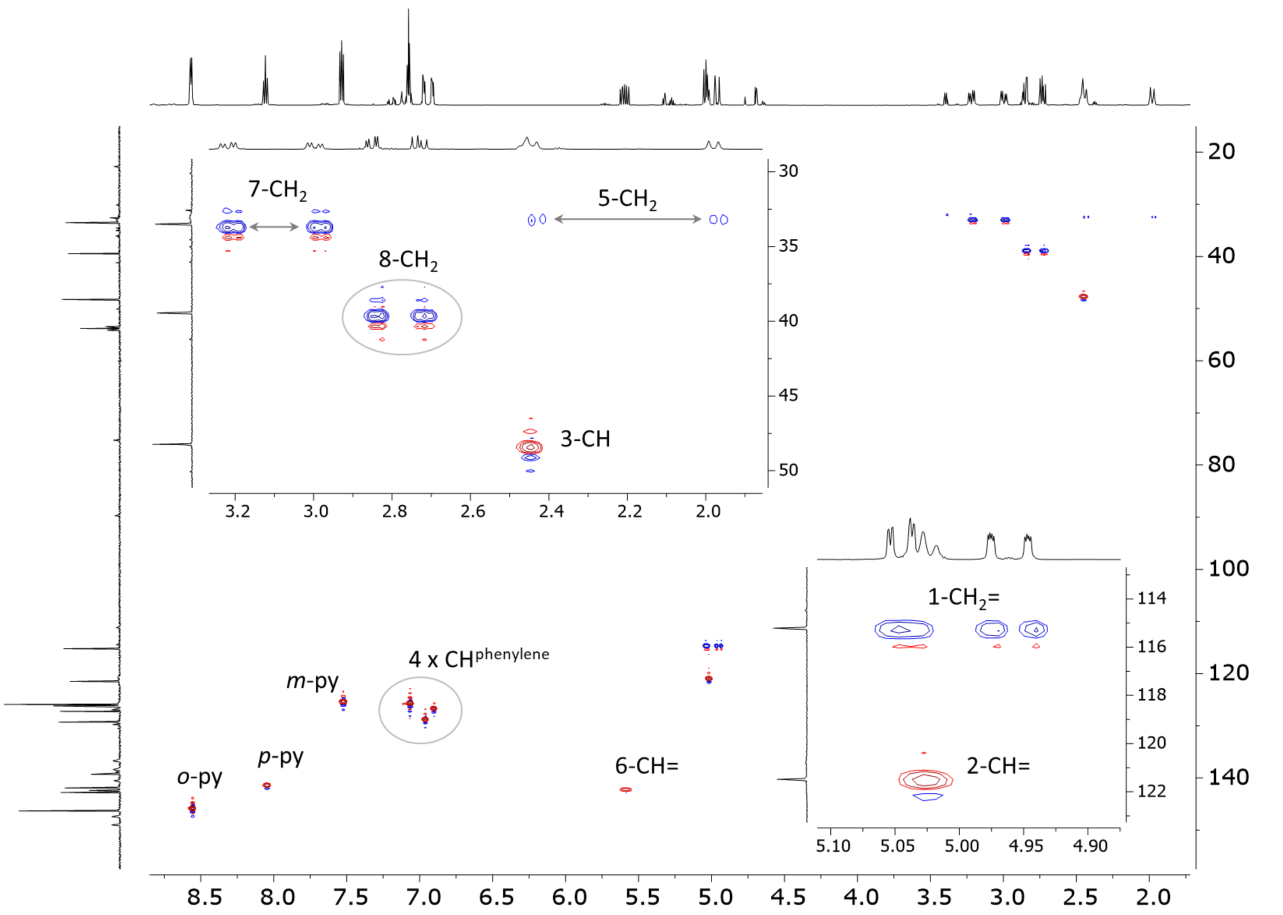

Figure S38. ^{1}H,^{13}C GHSQC (600/151 MHz, CD_2Cl_2, 299K) spectrum of compound **6c**.

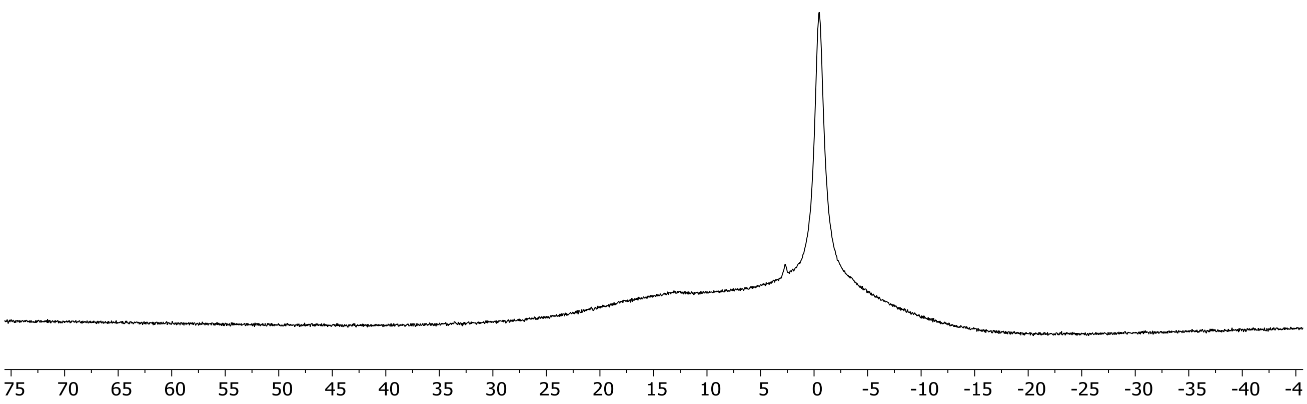

Figure S39. $^{11}B\{^1H\}$ NMR (192 MHz, 299 K, CD_2Cl_2) spectrum of compound **6c**.

I) Generation of compound **7a**

Scheme S11.

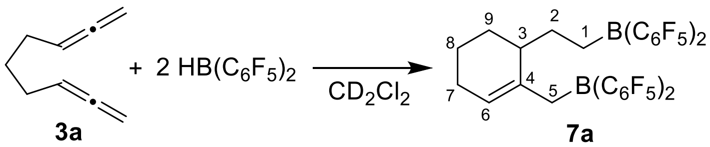

A suspension of $HB(C_6F_5)_2$ (69.2 mg, 0.20 mmol) in CD_2Cl_2 (0.5 mL) was added to a solution of bisallene **3a** (12.0 mg, 0.10 mmol) in CD_2Cl_2 (0.5 mL) at room temperature. The resulting reaction mixture was characterized by NMR experiments after storage for 1 h at room temperature.

NMR data of compound **7a** in the mixture:

^{1}H NMR (600 MHz, 299 K, CD_2Cl_2): δ ^{1}H: 5.43 (t, $^3J_{HH}$ = 3.7 Hz, 1H, 6-CH=), 2.87/2.78 (each d, $^2J_{HH}$ = 15.9 Hz, each 1H, 5-CH_2), 2.13/1.99 (each m, each 1H, 1-CH_2), 1.90 (m, 2H, 7-CH_2), 1.76/1.41 (each m, each 1H, 2-CH_2), 1.71 (m, 1H, CH), 1.49 (m, 2H, 9-CH_2), 1.37 (m, 2H, 8-CH_2).

$^{13}C\{^1H\}$ NMR (151 MHz, 299 K, CD_2Cl_2): δ ^{13}C: 136.2 (C=), 128.2 (6-CH=), 42.1 (CH), 38.5 (br, 5-CH_2), 30.2 (br, 1-CH_2), 28.0 (2-CH_2), 26.7 (9-CH_2), 26.4 (7-CH_2), 18.6 (8-CH_2), [C_6F_5 not listed].

^{19}F NMR (564 MHz, 299 K, CD_2Cl_2): δ ^{19}F: **[**–130.0/–130.5](each m, each 2F, *o*-C_6F_5), [–148.5/–148.9] (each tt, $^3J_{FF}$ = 20.0 Hz, $^4J_{FF}$ = 4.4 Hz, each 1F, *p*-C_6F_5), [–161.9/–162.2](each m, each 2F, *m*-C_6F_5).

$^{11}B\{^1H\}$ NMR (192 MHz, 299 K, CD_2Cl_2): δ ^{11}B: 73.3 ($\nu_{1/2}$ ~ 1000 Hz), 69.4 ($\nu_{1/2}$ ~ 1000 Hz).

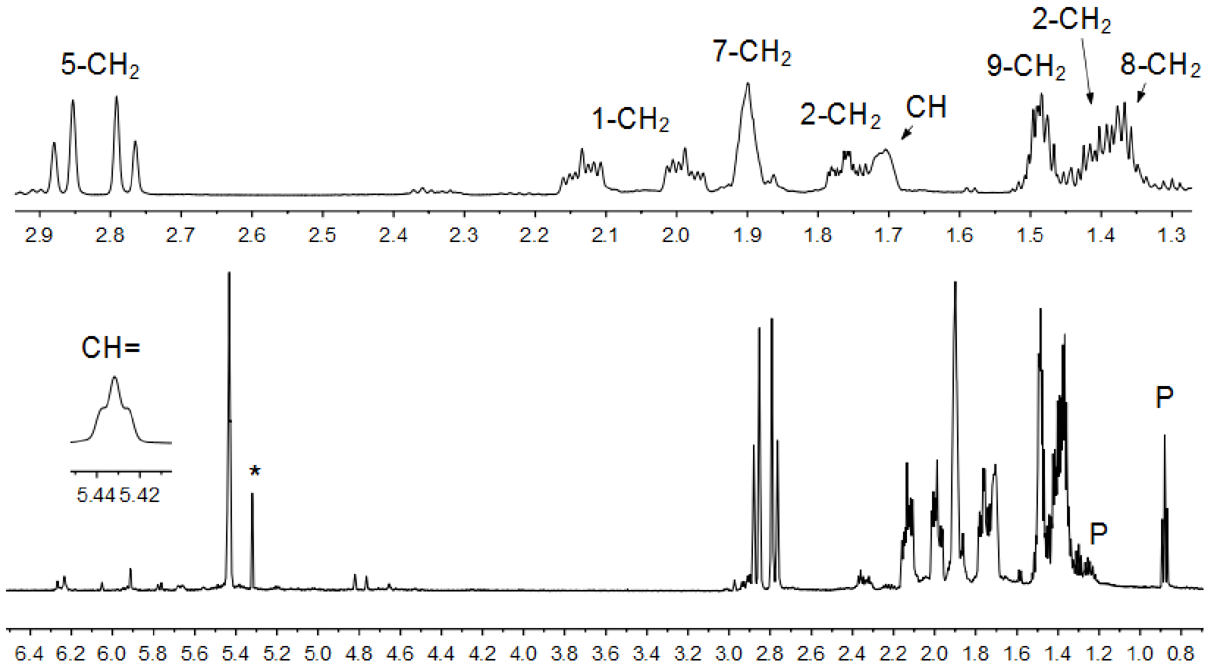

Figure S40. ^{1}H NMR (600 MHz, 299 K, CD_2Cl_2*) spectrum of the reaction mixture. [P: pentane]

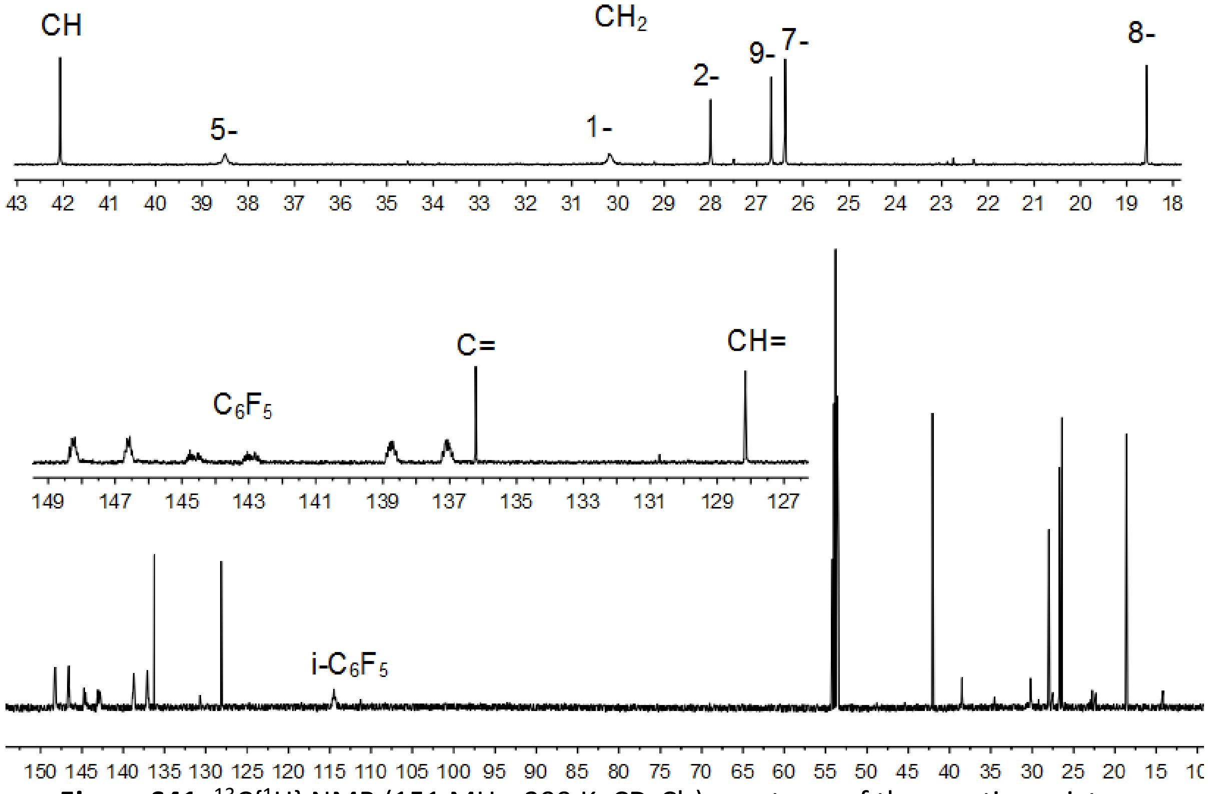

Figure S41. $^{13}C\{^{1}H\}$ NMR (151 MHz, 299 K, CD_2Cl_2) spectrum of the reaction mixture.

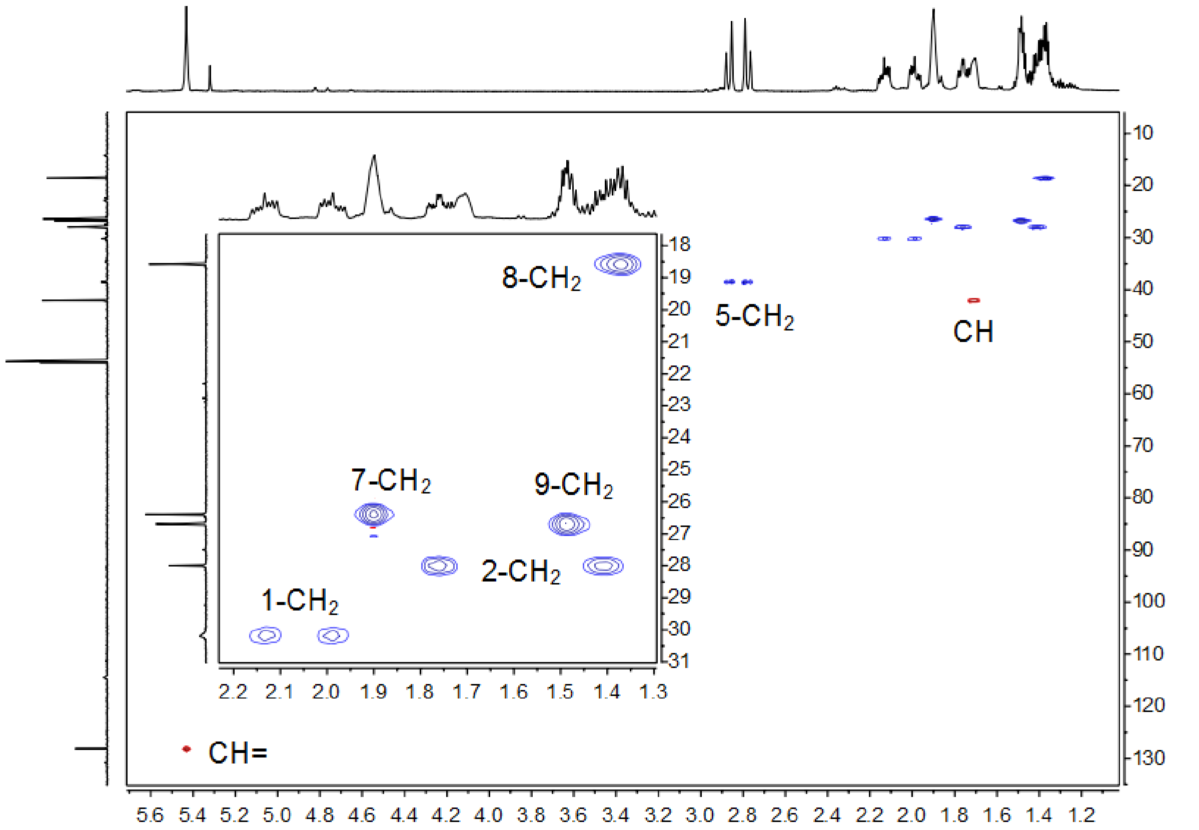

Figure S42. ^{1}H,^{13}C GHSQC (600/151 MHz, CD_2Cl_2, 299K) spectrum of the reaction mixture.

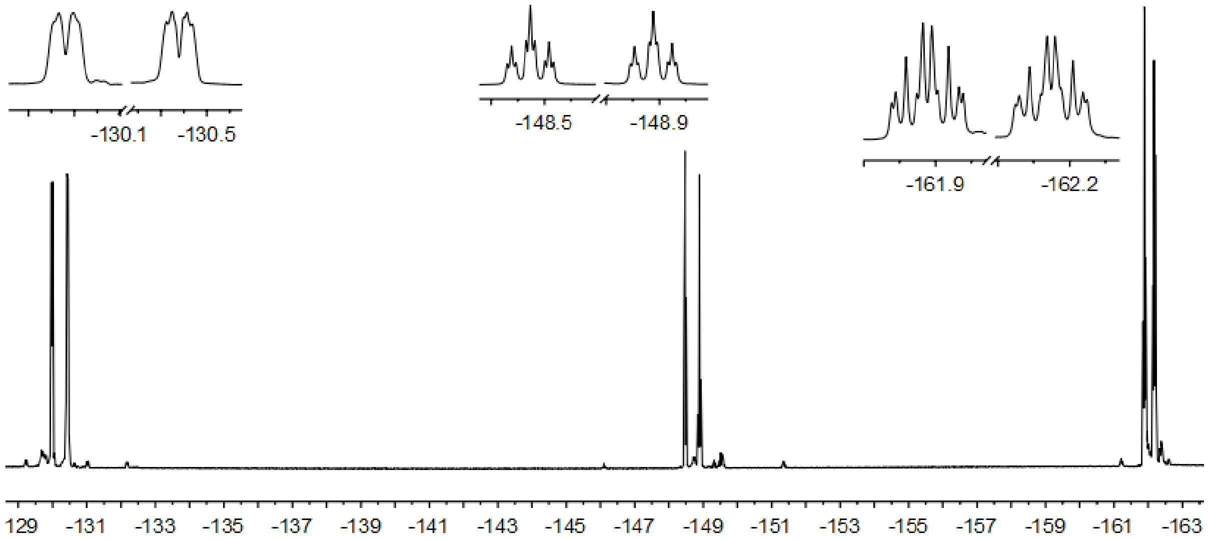

Figure S43. ^{19}F NMR (564 MHz, 299 K, CD_2Cl_2) spectrum of the reaction mixture.

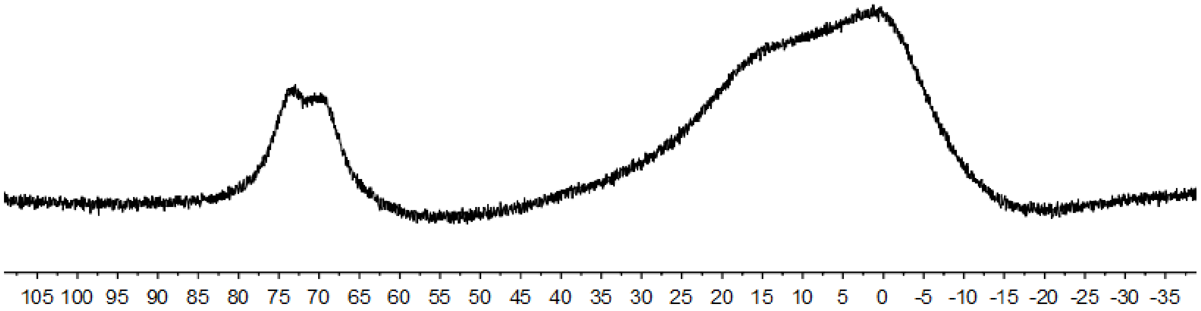

Figure S44. $^{11}B\{^1H\}$ NMR (192 MHz, 299 K, CD_2Cl_2) spectrum of the reaction mixture.

J) Synthesis of compound **8a**

Scheme S12.

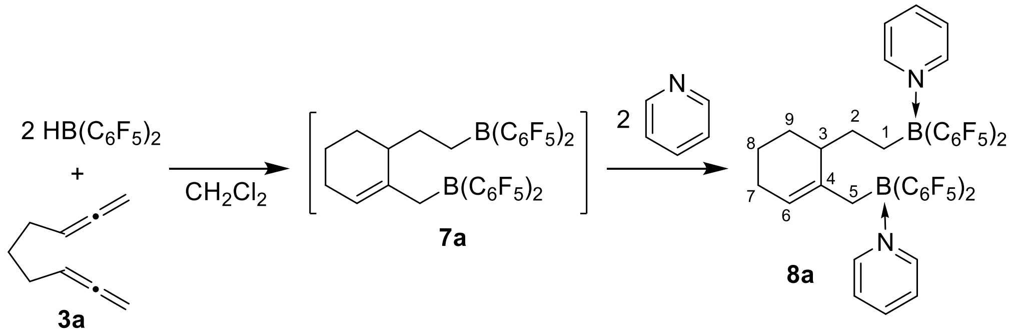

A suspension of $HB(C_6F_5)_2$ (103.8 mg, 0.30 mmol) in CH_2Cl_2 (1 mL) was added to a solution of bisallene **3a** (18.0 mg, 0.15 mmol) in CH_2Cl_2 (1 mL) at room temperature. Subsequently, pyridine (24 mg, 0.30 mmol) was added to the resulting reaction mixture. Then all volatile were removed in vacuo and the residue was washed with pentane (1 mL × 3). After the obtained crude white powder was dissolved in CH_2Cl_2 (0.5 mL), pentane (3 mL) was added dropwise to the stirring solution. The resulting suspension was filtrated. The residual solid was washed with pentane (1 mL × 3) and dried in vacuo giving compound **8a** (105 mg, 0.11 mmol, 72%) as a white solid.

Anal. Calc. for $C_{43}H_{24}B_2F_{20}N_2$: C, 53.23; H, 2.49; N, 2.89. Found: C, 53.07; H, 2.53; N, 2.90.

^{1}H NMR (600 MHz, 299 K, CD_2Cl_2): δ ^{1}H: [8.72 (2H, o), 8.14 (1H, p), 7.69 (2H, m)](each m, Py), [8.49 (2H, o), 8.10 (1H, p), 7.63 (2H, m)](Py'), 4.35 (t, $^3J_{HH}$ = 3.3 Hz, 1H, 6-CH=), 2.45/1.63 (each d, $^2J_{HH}$ = 14.0 Hz, each 1H, 5-CH_2), [1.66 (2H), 1.47 (1H), 1.33/0.87 (each 1H), 1.27 (2H), 1.17 (1H)](each m, CH_2), 1.47/1.05 (each m, each 1H, 1-CH_2), 1.36 (m, 1H, CH),.

^{13}C{^{1}H} NMR (151 MHz, 299 K, CD_2Cl_2): δ ^{13}C: [146.3 (o), 142.0 (p), 126.4 (m)](Py), [146.1 (o), 141.8 (p), 125.6 (m)](Py'), 143.1 (C=), 120.9 (6-CH=), 41.0 (CH), 31.9 (br, 5-CH_2), [29.5, 26.18, 26.16, 18.0](CH_2), 22.3 (br, 1-CH_2), [C_6F_5 not listed].

^{19}F NMR (564 MHz, 299 K, CD_2Cl_2): δ ^{19}F: [−130.8, −130.9, −132.1, −132.3](each m, each 2F, *o*-C_6F_5), [−159.1, −159.5, −159.77, −159.81](each t, $^3J_{FF}$ = 20.0 Hz, each 1F, *p*-C_6F_5), [−164.7, −164.8, −164.9, −165.1] (each m, each 2F, *m*-C_6F_5).

$^{11}B\{^1H\}$ NMR (192 MHz, 299 K, CD_2Cl_2): δ ^{11}B: –0.7 ($\nu_{1/2}$ ~ 350 Hz).

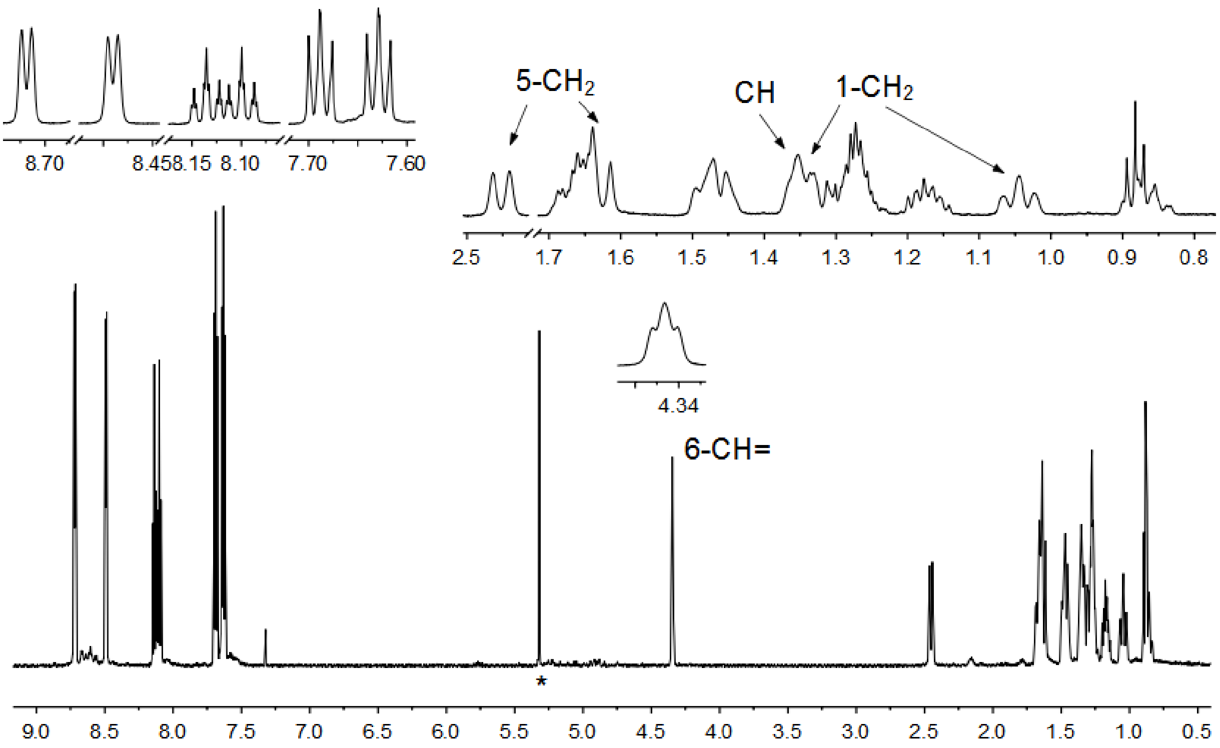

Figure S45. ^1H NMR (600 MHz, 299 K, CD_2Cl_2*) spectrum of compound **8a**.

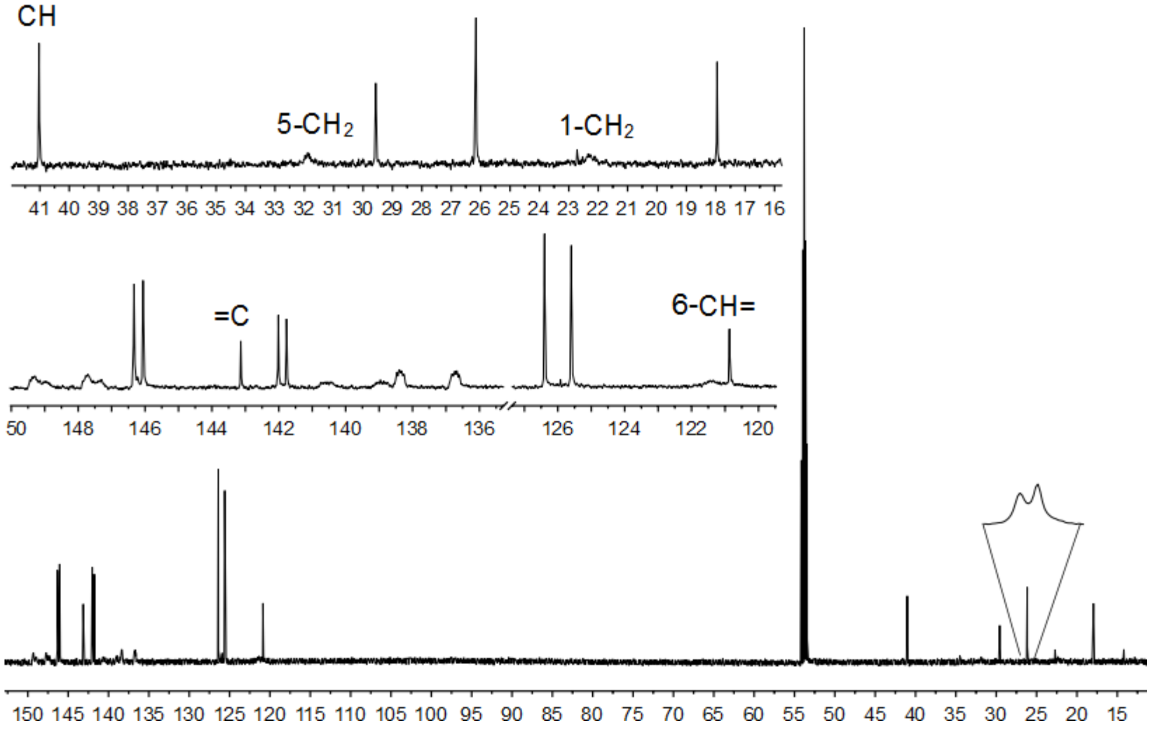

Figure S46. $^{13}C\{^1H\}$ NMR (151 MHz, 299 K, CD_2Cl_2) spectrum of compound **8a**.

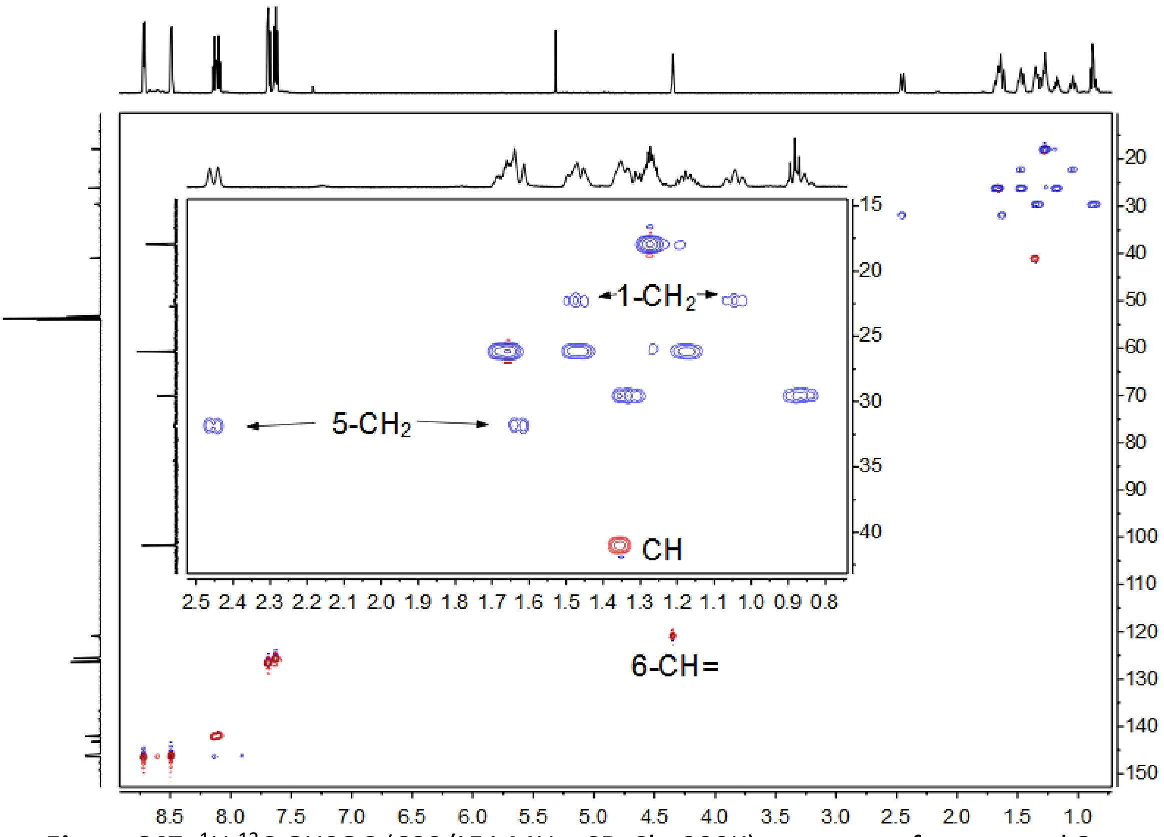

Figure S47. ^{1}H,^{13}C GHSQC (600/151 MHz, CD_2Cl_2, 299K) spectrum of compound **8a**.

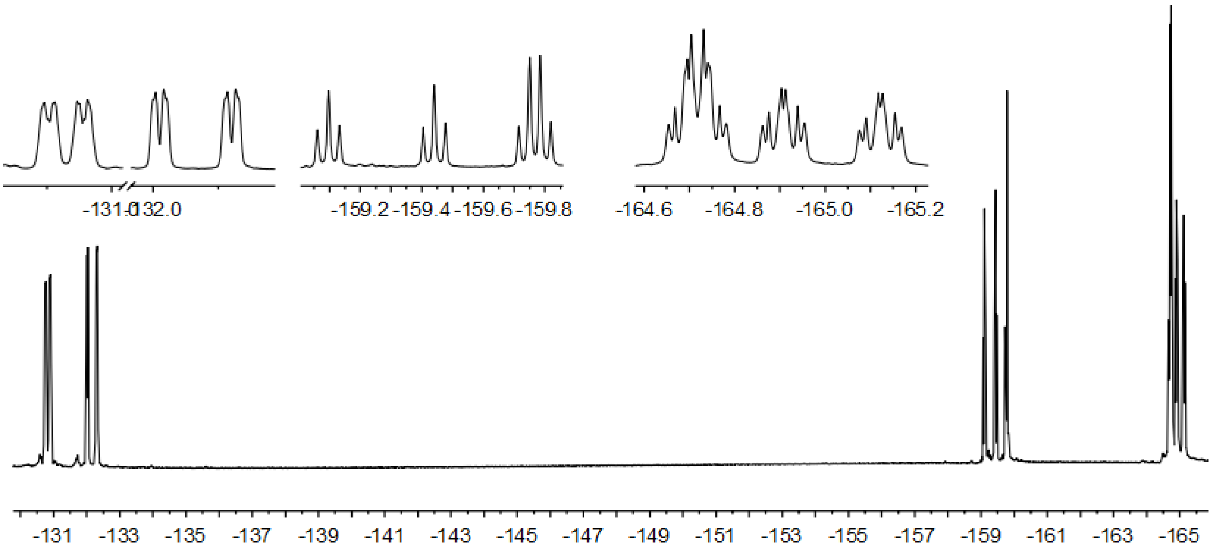

Figure S48. ^{19}F NMR (564 MHz, 299 K, CD_2Cl_2) spectrum of compound **8a**.

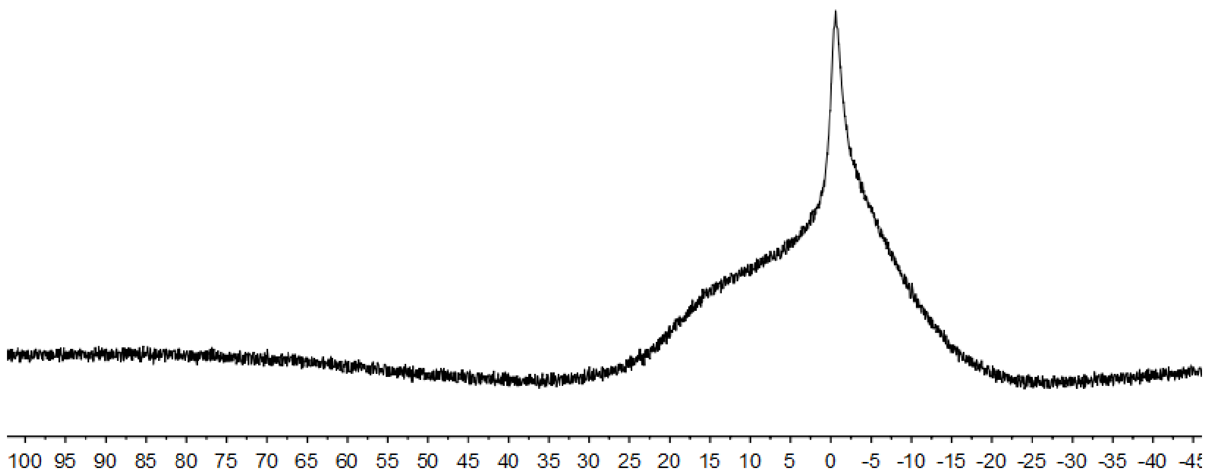

Figure S49. $^{11}B\{^{1}H\}$ NMR (192 MHz, 299 K, CD_2Cl_2) spectrum of compound **8a**.

Crystals suitable for the X-ray crystal structure analysis were obtained from diffusion of pentane vapor to a solution of compound **8a** in CH_2Cl_2 at room temperature.

X-ray crystal structure analysis of compound 8a (erk9360): A colorless needle-like specimen of $C_{43}H_{24}B_2F_{20}N_2$, approximate dimensions 0.020 mm x 0.050 mm x 0.080 mm, was used for the X-ray crystallographic analysis. The X-ray intensity data were measured. A total of 1607 frames were collected. The total exposure time was 37.06 hours. The frames were integrated with the Bruker SAINT software package using a wide-frame algorithm. The integration of the data using a triclinic unit cell yielded a total of 30895 reflections to a maximum θ angle of 66.94° (0.84 Å resolution), of which 7137 were independent (average redundancy 4.329, completeness = 98.9%, R_{int} = 11.47%, R_{sig} = 10.08%) and 4021 (56.34%) were greater than $2\sigma(F^2)$. The final cell constants of a = 9.0564(10) Å, b = 10.3497(9) Å, c = 21.915(2) Å, α = 91.873(6)°, β = 96.630(7)°, γ = 96.996(6)°, volume = 2022.9(4) $Å^3$, are based upon the refinement of the XYZ-centroids of 3416 reflections above 20 σ(I) with 8.132° < 2θ < 128.3°. Data were corrected for absorption effects using the multi-scan method (SADABS). The ratio of minimum to maximum apparent transmission was 0.841. The calculated minimum and maximum transmission coefficients (based on crystal size) are 0.8950 and 0.9720. The structure was solved and refined using the Bruker SHELXTL Software Package, using the space group *P*-1, with Z = 2 for the formula unit, $C_{43}H_{24}B_2F_{20}N_2$. The final anisotropic full-matrix least-squares refinement on F^2 with 605 variables converged at R1 = 5.97%, for the observed data and wR2 = 17.03% for all data. The goodness-of-fit was 1.021. The largest peak in the final difference electron density synthesis was 0.273 $e^-/Å^3$ and the largest hole was -0.277 $e^-/Å^3$ with an RMS deviation of 0.065 $e^-/Å^3$. On the basis of the final model, the calculated density was 1.593 g/cm^3 and F(000), 972 e^-. CCDC number: 1922908.

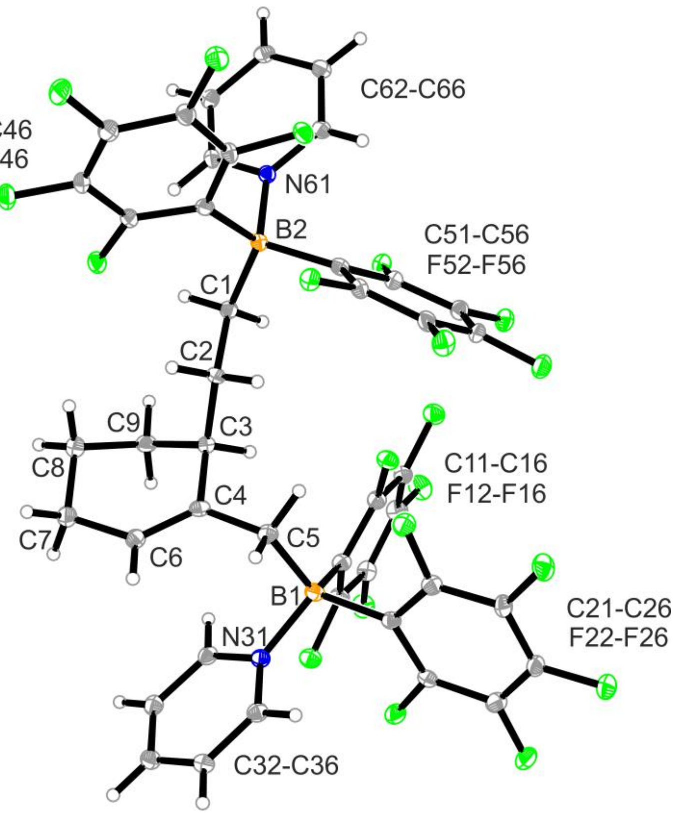

Figure S50. Crystal structure of compound **8a** (thermal ellipsoids: 15% probability).

K) Synthesis of compound 9a

Scheme S13.

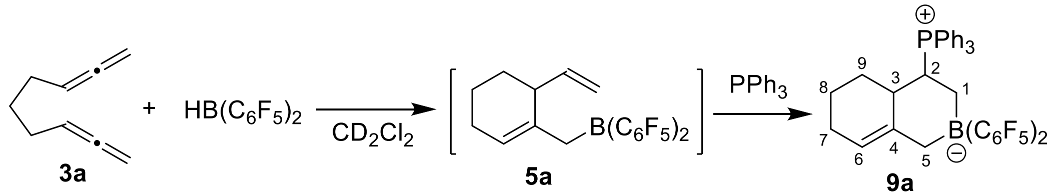

A suspension of $HB(C_6F_5)_2$ (103.8 mg, 0.30 mmol) in CH_2Cl_2 (1 mL) was added to a solution of bisallene **3a** (36.0 mg, 0.30 mmol) in CH_2Cl_2 (1 mL) at room temperature. Subsequently, triphenylphosphane (78.9 mg, 0.30 mmol) was added to the reaction mixture. Then all the volatiles were removed in vacuo and the residue was washed with pentane (1 mL × 3). Drying of the remaining solid gave compound **9a** (167 mg, 0.23 mmol, 76%) as a white powder.

Anal. Calc. for $C_{39}H_{28}BF_{10}P$: C, 64.31; H, 3.87. Found: C, 64.30; H, 4.01.

^{1}H NMR (600 MHz, 299 K, CD_2Cl_2): δ ^{1}H: 7.75 (m, 6H, o-Ph), 7.73 (m, 3H, p-Ph), 7.63 (m, 6H, m-Ph), 5.17 (br, 1H, 6-CH=), 3.64 (m, 1H, PCH), 2.15/1.23 (each dm, $^2J_{HH}$ = 12.3 Hz, each 1H, 5-CH_2), 2.06 (m, 1H, 3-CH), [1.93 (t, J = 11.9 Hz)/0.68 (m, 1H)](each 1H, 1-CH_2), 1.82 (m, 2H, 7-CH_2), 1.43/1.22 (each m,

each 1H, 8-CH_2), 1.38 (m, 2H, 9-CH_2).

$^{13}C\{^1H\}$ NMR (151 MHz, 299 K, CD_2Cl_2): δ ^{13}C: 143.3 (d, $^3J_{PC}$ = 13.4 Hz, C=), 134.2 (d, $^4J_{PC}$ = 3.1 Hz, p-Ph), 133.6 (d, $^2J_{PC}$ = 8.4 Hz, o-Ph), 130.2 (d, $^3J_{PC}$ = 11.7 Hz, m-Ph), 121.6 (d, $^1J_{PC}$ = 79 Hz, i-Ph), 118.4 (d, $^4J_{PC}$ = 2.3 Hz, 6-CH=), 41.1 (d, $^2J_{PC}$ = 0.9 Hz, 3-CH), 39.1 (dm, $^1J_{PC}$ = 25.0 Hz, PCH), 36.0 (br m, 5-CH_2), 30.5 (d, $^3J_{PC}$ = 2.8 Hz, 9-CH_2), 25.9 (7-CH_2), 24.8 (br m, 1-CH_2), 21.0 (8-CH_2), [C_6F_5 not listed].

^{19}F NMR (564 MHz, 299 K, CD_2Cl_2): δ ^{19}F: [–132.6 (m, 2F, *o*), –163.7 (t, $^3J_{FF}$ = 20.3 Hz, 1F, *p*), –166.6 (m, 2F, *m*)](C_6F_5)[$\Delta\delta^{19}F_{m,p}$ = 2.9], [–134.1 (m, 2F, *o*), –164.4 (t, $^3J_{FF}$ = 20.3 Hz, 1F, *p*), –166.9 (m, 2F, *m*)](C_6F_5)[$\Delta\delta^{19}F_{m,p}$ = 2.5].

$^{11}B\{^1H\}$ NMR (192 MHz, 299 K, CD_2Cl_2): δ ^{11}B: –12.3 ($\nu_{1/2}$ ~ 70 Hz).

$^{31}P\{^1H\}$ NMR (243 MHz, 299 K, CD_2Cl_2): δ ^{31}P: 27.9 ($\nu_{1/2}$ ~ 30 Hz).

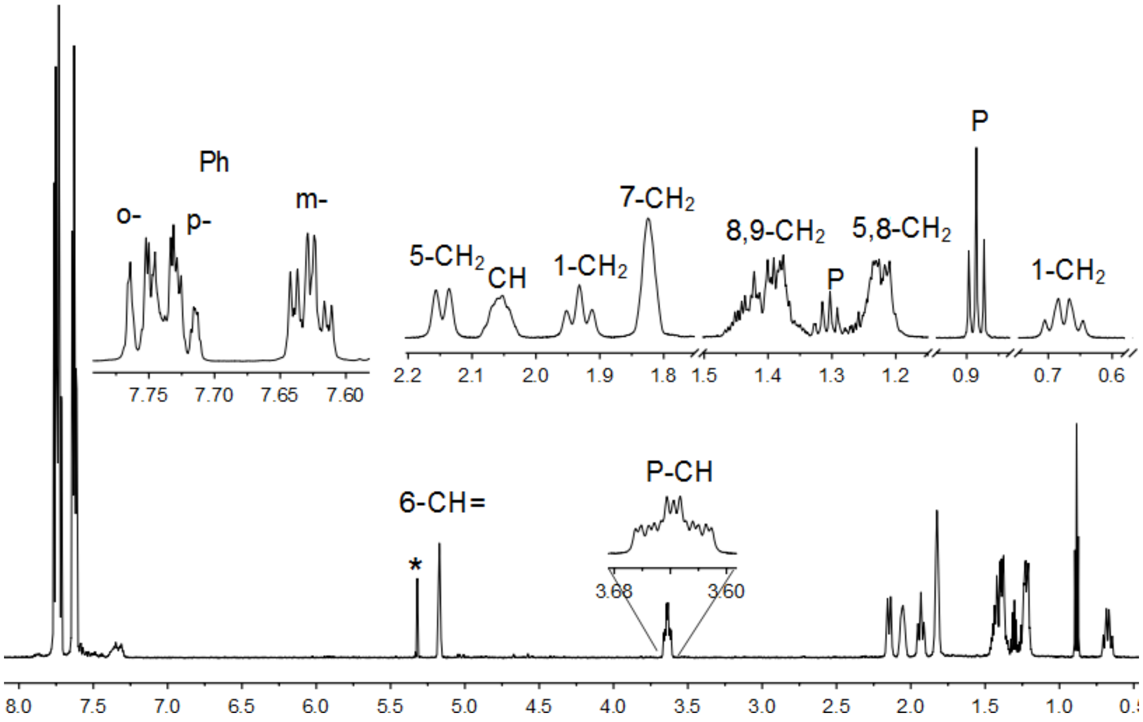

Figure S51. ^{1}H NMR (600 MHz, 299 K, CD_2Cl_2*) spectrum of compound **9a**. [P: pentane]

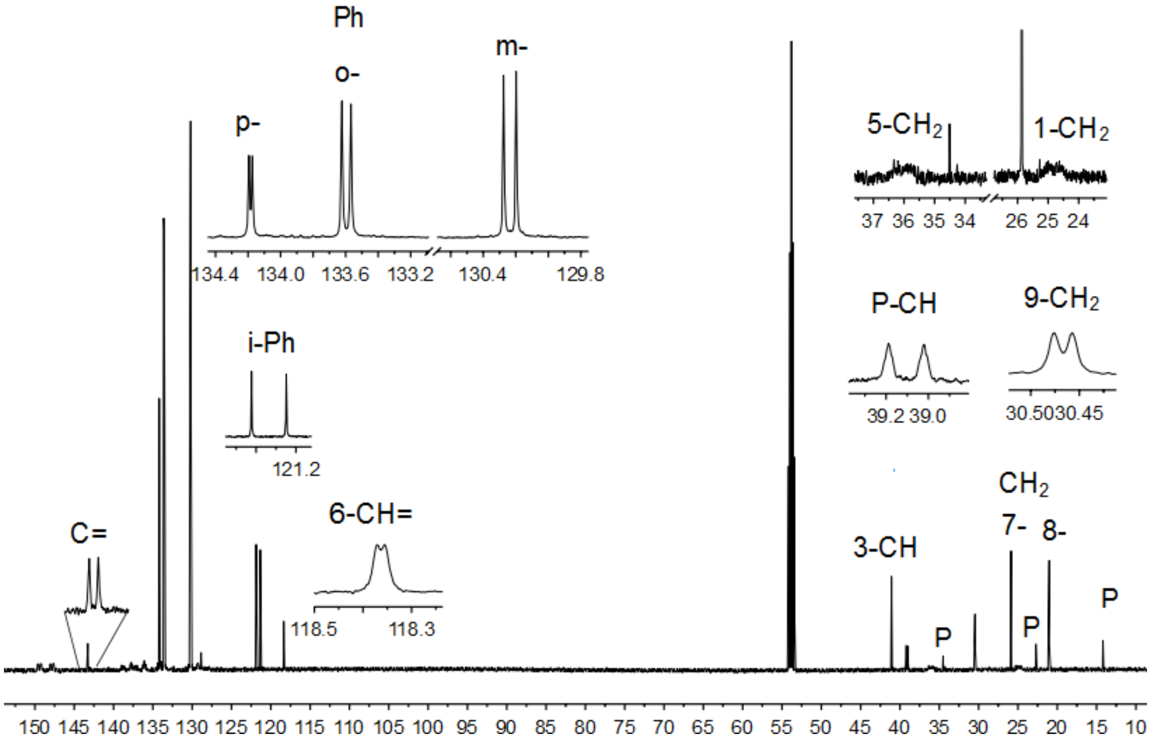

Figure S52. $^{13}C\{^1H\}$ NMR (151 MHz, 299 K, CD_2Cl_2) spectrum of compound **9a**. [P: pentane]

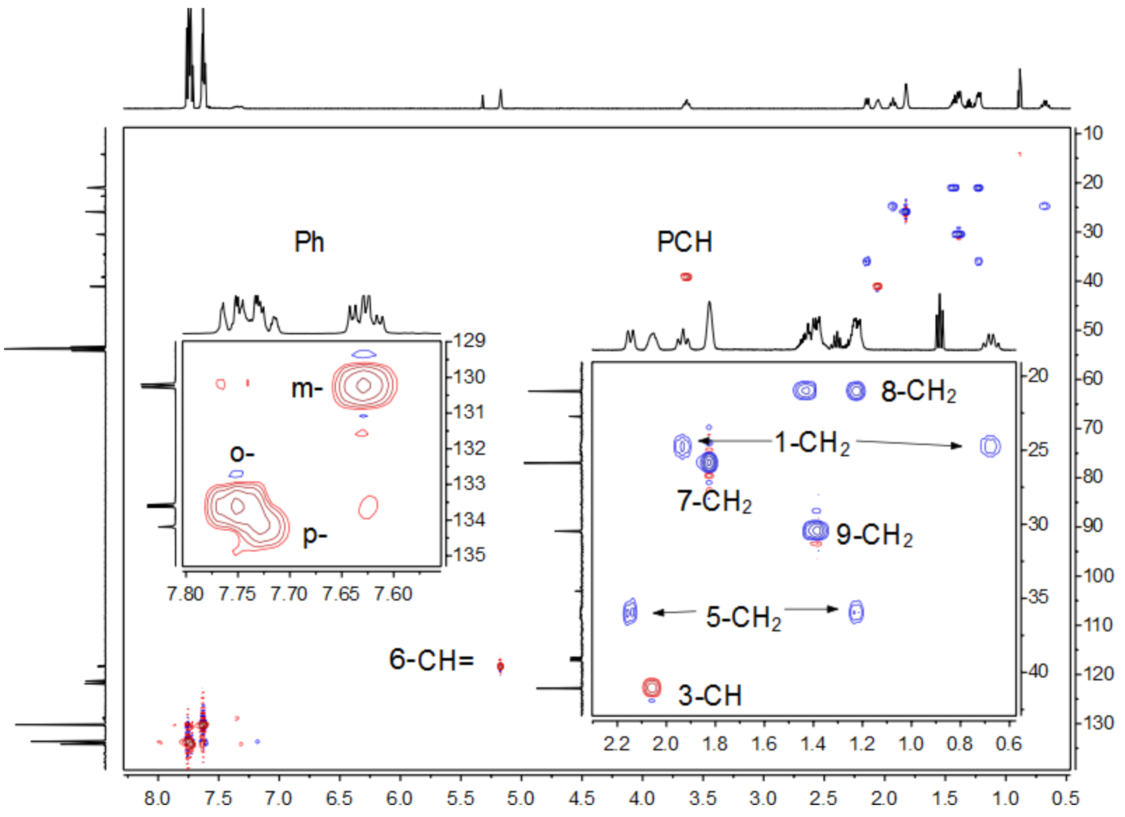

Figure S53. $^1H,^{13}C$ GHSQC (600/151 MHz, CD_2Cl_2, 299K) spectrum of compound **9a**.

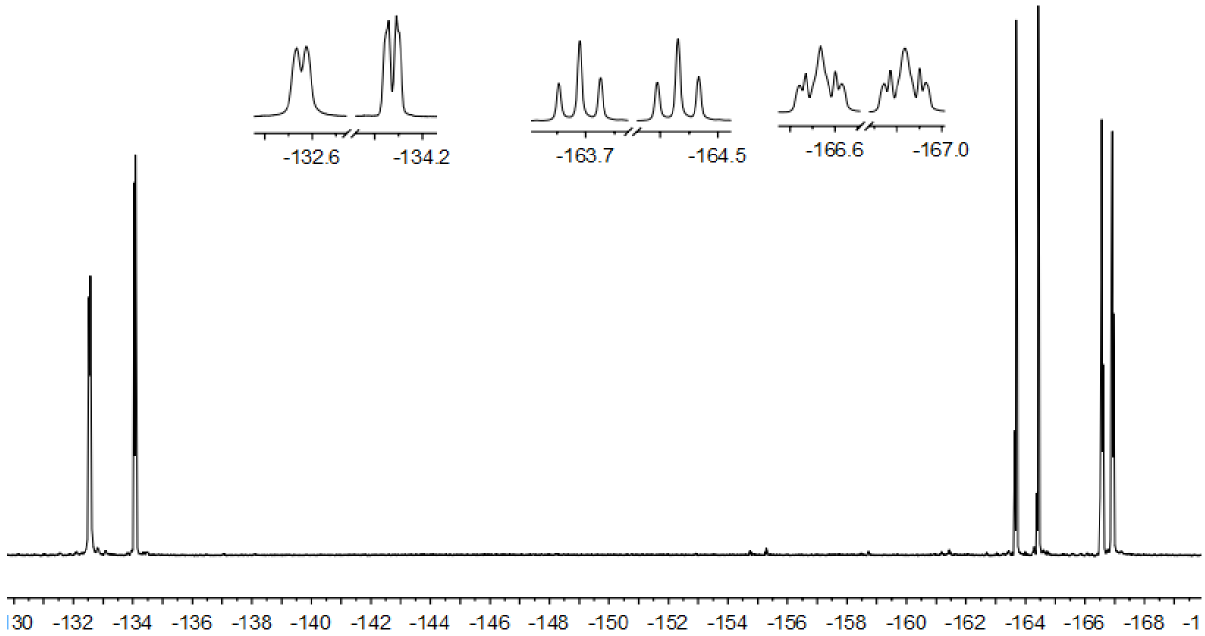

Figure S54. ^{19}F NMR (564 MHz, 299 K, CD_2Cl_2) spectrum of compound **9a**.

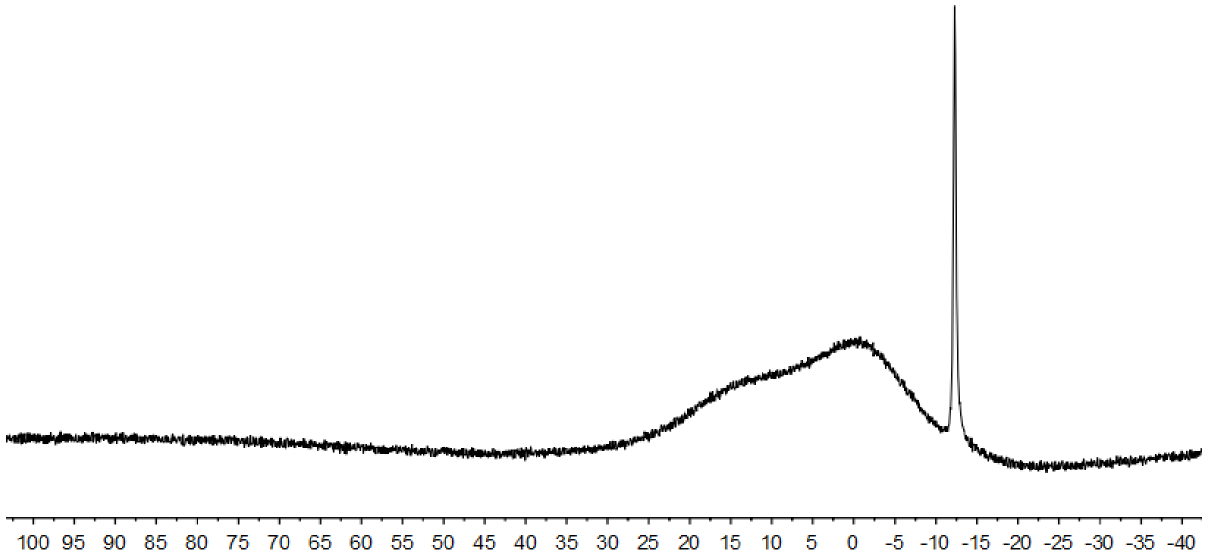

Figure S55. $^{11}B\{^1H\}$ NMR (192 MHz, 299 K, CD_2Cl_2) spectrum of compound **9a**.

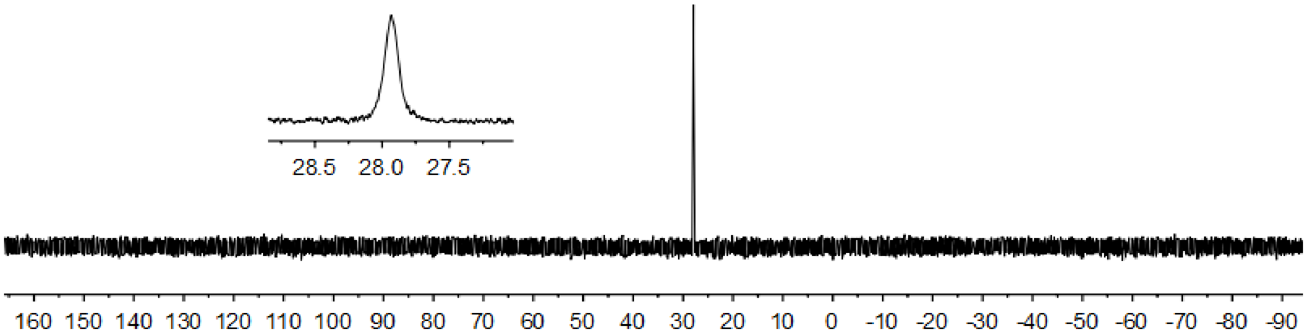

Figure S56. $^{31}P\{^1H\}$ NMR (243 MHz, 299 K, CD_2Cl_2) spectrum of compound **9a**.

Crystals suitable for the X-ray crystal structure analysis was obtained from two-layer diffusion of pentane to a solution of the isolated white powder in CH_2Cl_2 at room temperature.

X-ray crystal structure analysis of compound 9a (erk9369): A colorless prism-like specimen of $C_{39}H_{28}BF_{10}P$, approximate dimensions 0.120 mm x 0.200 mm x 0.240 mm, was used for the X-ray crystallographic analysis. The X-ray intensity data were measured. A total of 1769 frames were collected. The total exposure time was 18.18 hours. The frames were integrated with the Bruker SAINT software package using a wide-frame algorithm. The integration of the data using a monoclinic unit cell yielded a total of 52800 reflections to a maximum θ angle of 66.76° (0.84 Å resolution), of which 6392 were independent (average redundancy 8.260, completeness = 99.8%, R_{int} = 4.95%, R_{sig} = 2.54%) and 5636 (88.17%) were greater than $2\sigma(F^2)$. The final cell constants of a = 12.2953(3) Å, b = 14.3056(3) Å, c = 20.8746(5) Å, β = 100.3000(10)°, volume = 3612.50(15) Å^3, are based upon the refinement of the XYZ-centroids of 9851 reflections above 20 σ(I) with 7.307° < 2θ < 133.4°. Data were corrected for absorption effects using the multi-scan method (SADABS). The ratio of minimum to maximum apparent transmission was 0.869. The calculated minimum and maximum transmission coefficients (based on crystal size) are 0.7340 and 0.8520. The structure was solved and refined using the Bruker SHELXTL Software Package, using the space group $P2_1/c$, with Z = 4 for the formula unit, $C_{39}H_{28}BF_{10}P$. The final anisotropic full-matrix least-squares refinement on F^2 with 460 variables converged at R1 = 3.59%, for the observed data and wR2 = 9.09% for all data. The goodness-of-fit was 1.022. The largest peak in the final difference electron density synthesis was 0.380 e^-/Å^3 and the largest hole was -0.383 e^-/Å^3 with an RMS deviation of 0.046 e^-/Å^3. On the basis of the final model, the calculated density was 1.339 g/cm^3 and F(000), 1488 e^-. CCDC number: 1922909.

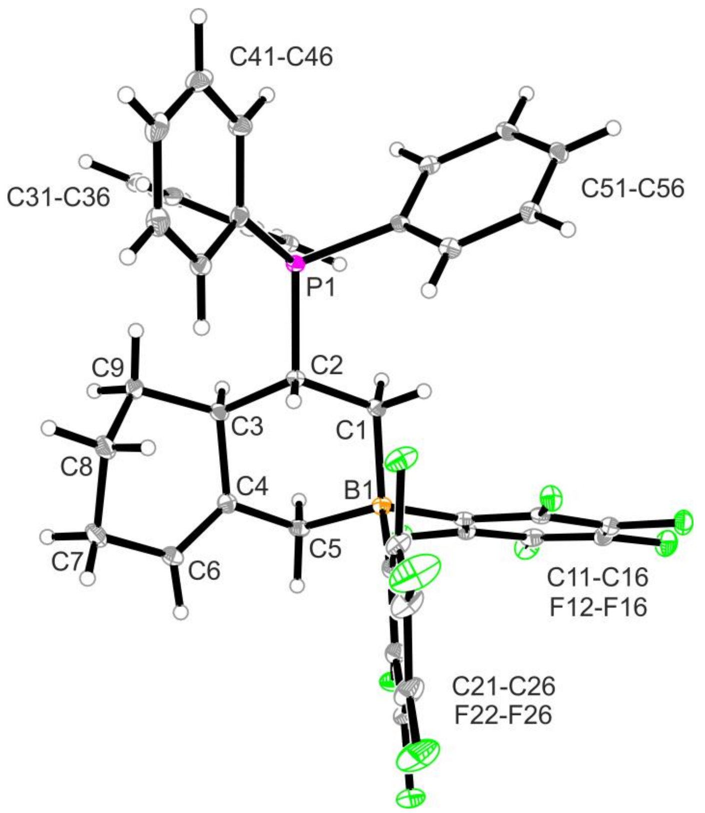

Figure S57. Crystal structure of compound **9a** (thermal ellipsoids: 30% probability).

L) Synthesis of compound **9b**

Scheme S14.

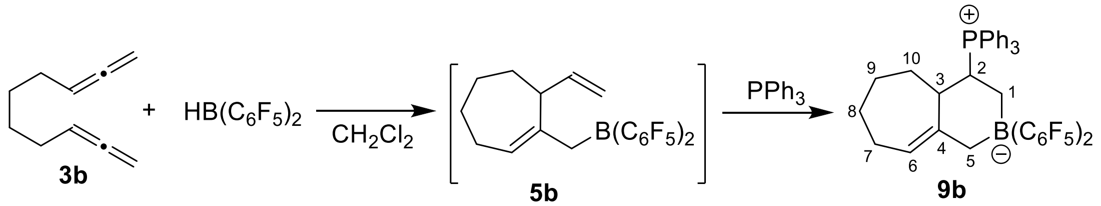

A suspension of $HB(C_6F_5)_2$ (69.2 mg, 0.20 mmol) in CH_2Cl_2 (1 mL) was added to a solution of bisallene **3b** (26.8 mg, 0.20 mmol) in CH_2Cl_2 (1 mL) at room temperature. Subsequently, triphenylphosphane (52.6 mg, 0.20 mmol) was added to the reaction mixture. Then all the volatiles were removed in vacuo and the residue was washed with pentane (1 mL × 3). The obtained crude product was dissolved in CH_2Cl_2 (0.5 mL), the resulting solution was covered by pentane (2 mL) and stored at –35 °C for 3d. The solution was removed by decantation and the remaining solid was washed with pentane (1 mL × 3). Drying of the solid in vacuo gave pure compound **9b** (91 mg, 0.12 mmol, 61%) as a white powder.

Anal. Calc. for $C_{40}H_{30}BF_{10}P$: C, 64.71; H, 4.07. Found: C, 64.46; H, 4.02.

NMR data of compound **9b**:

^{1}H NMR (600 MHz, 299 K, CD_2Cl_2): δ ^{1}H: 7.75 (m, 3H, p-Ph), 7.66 (m, 6H, o-Ph), 7.61 (m, 6H, m-Ph), 5.21 (t, $^3J_{HH}$ = 7.3 Hz, 1H, 6-CH=), 3.64 (m, 1H, PCH), 2.46 (q, J = 10.8 Hz, 1H, 3-CH), 2.14/1.77 (each d, $^2J_{HH}$ = 13.3 Hz, each 1H, 5-CH_2), 1.82/1.67 (each m, each 1H, 7-CH_2), [1.52/0.71 (each 1H), 1.35 (2H), 1.35/1.04 (each 1H)](each m, CH_2), 1.51/0.83 (each m, each 1H, 1-CH_2).

^{13}C{^{1}H} NMR (151 MHz, 299 K, CD_2Cl_2): δ ^{13}C: 149.5 (d, $^3J_{PC}$ = 11.6 Hz, C=), 134.4 (d, $^4J_{PC}$ = 3.0 Hz, p-Ph), 134.1 (d, $^2J_{PC}$ = 8.4 Hz, o-Ph), 130.2 (d, $^3J_{PC}$ = 11.2 Hz, m-Ph), 121.2 (d, $^1J_{PC}$ = 80.5 Hz, i-Ph), 119.8 (6-CH=), 45.6 (3-CH), 36.5 (d, $^1J_{PC}$ = 33.2 Hz, PCH), [31.9, 30.9, 26.9](CH_2), 26.9 (7-CH_2), 30.2 (br, 5-CH_2), 24.2 (br, 1-CH_2), [C_6F_5 not listed].

^{19}F NMR (564 MHz, 299 K, CD_2Cl_2): δ ^{19}F: [–133.0 (m, 2F, *o*), –164.1 (t, $^3J_{FF}$ = 20.3 Hz, 1F, *p*), –166.6 (m, 2F, *m*)](C_6F_5)[$\Delta\delta^{19}F_{m,p}$ = 2.5], [–133.5 (m, 2F, *o*), –164.8 (t, $^3J_{FF}$ = 20.3 Hz, 1F, *p*), –167.4 (m, 2F, *m*)](C_6F_5)[$\Delta\delta^{19}F_{m,p}$ = 2.6].

^{11}B{^{1}H} NMR (192 MHz, 299 K, CD_2Cl_2): δ ^{11}B: –13.3 ($\nu_{1/2}$ ~ 60 Hz).

^{31}P{^{1}H} NMR (243 MHz, 299 K, CD_2Cl_2): δ ^{31}P: 30.5 ($\nu_{1/2}$ ~ 40 Hz).

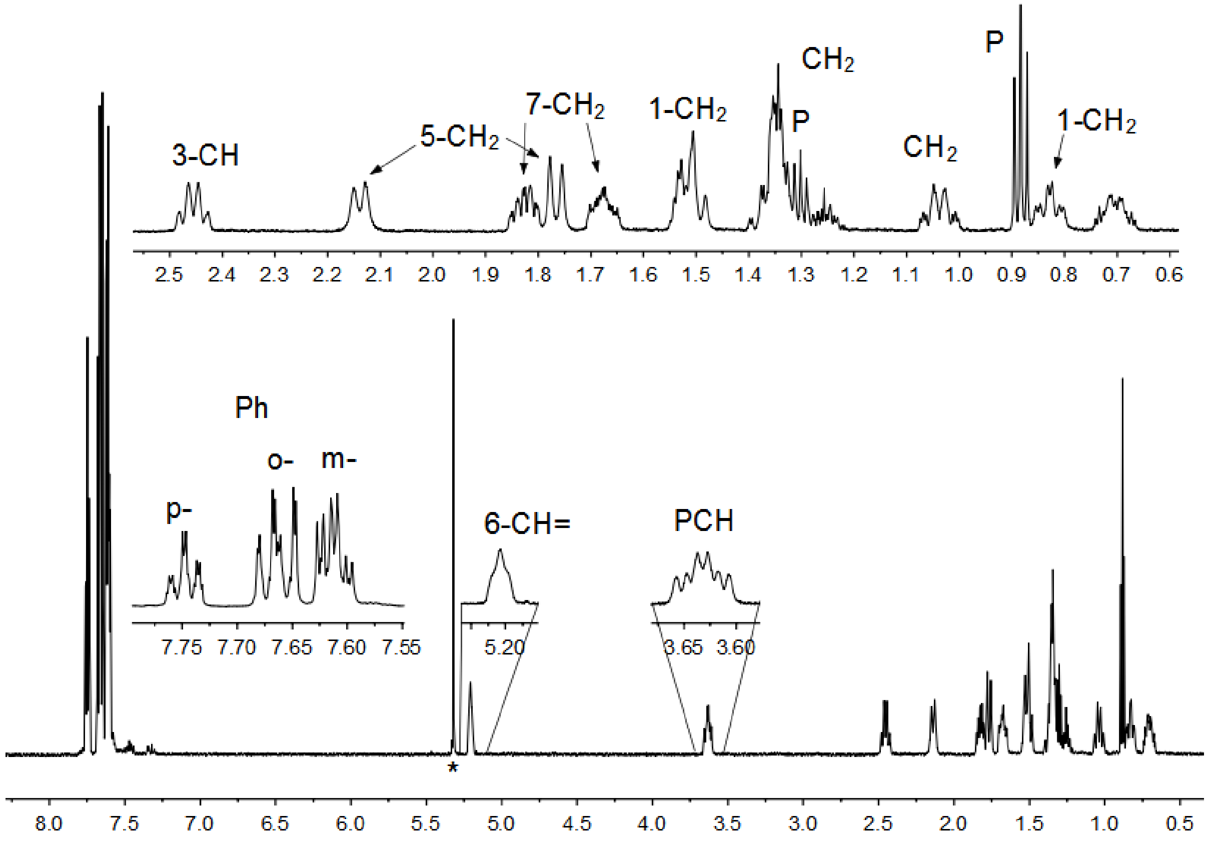

Figure S58. ^{1}H NMR (600 MHz, 299 K, CD_2Cl_2*) spectrum of compound **9b**.

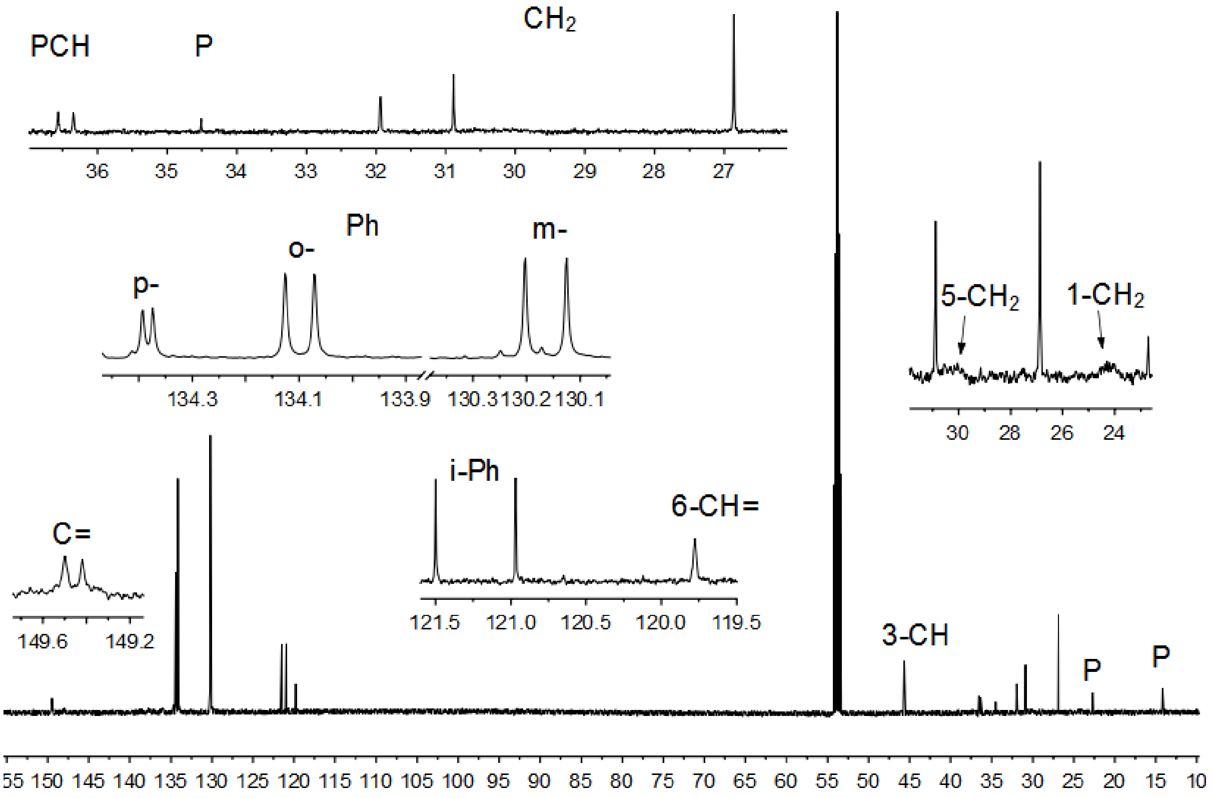

Figure S59. $^{13}C\{^{1}H\}$ NMR (151 MHz, 299 K, CD_2Cl_2) spectrum of compound **9b**. [P: pentane]

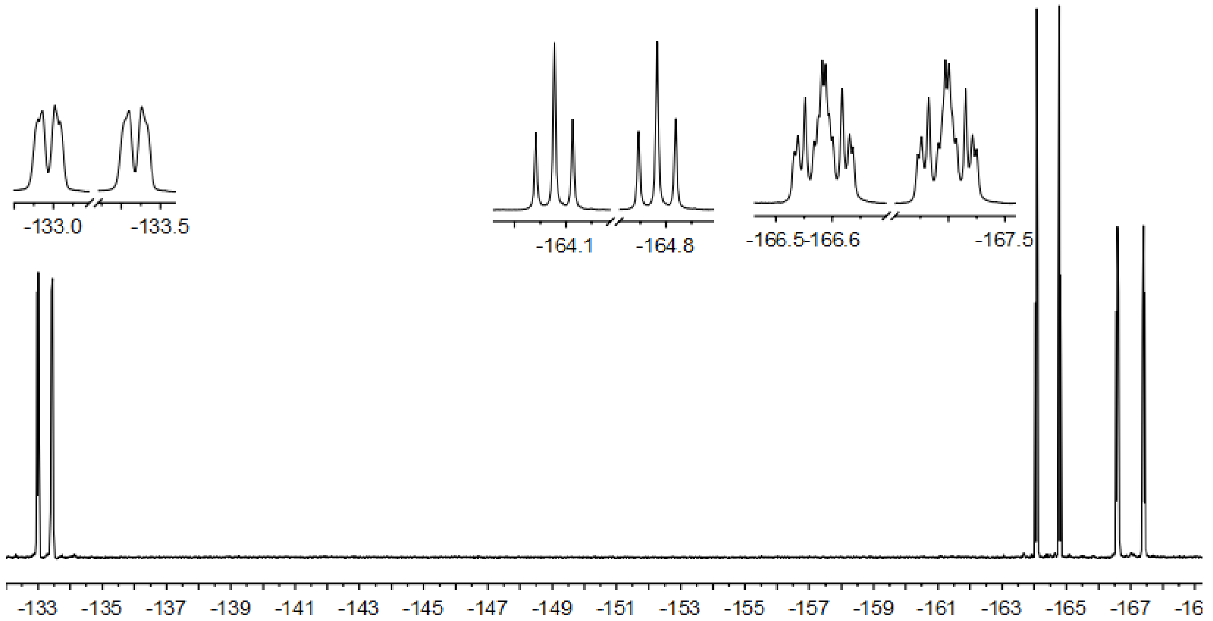

Figure S60. ^{19}F NMR (564 MHz, 299 K, CD_2Cl_2) spectrum of compound **9b**.

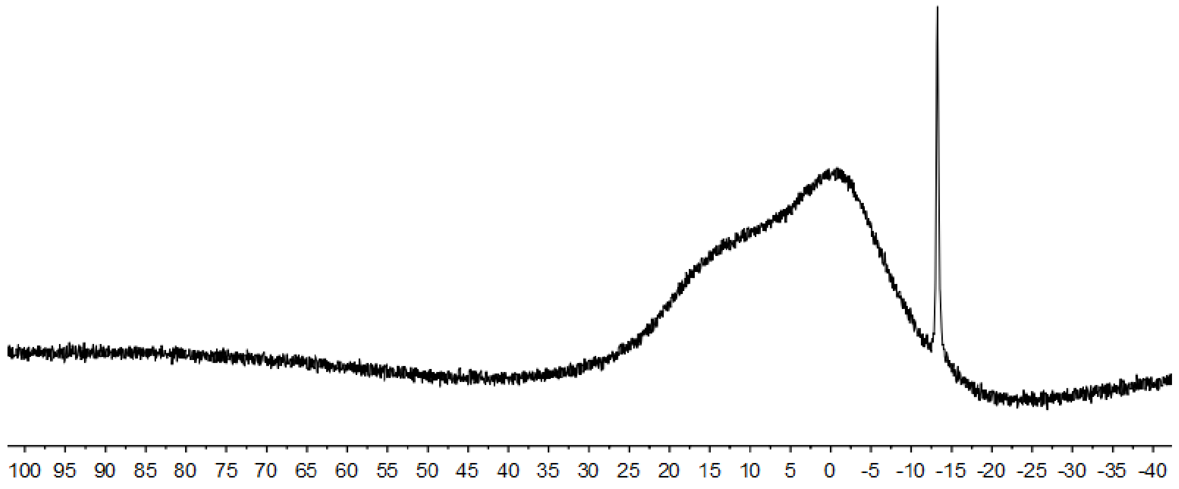

Figure S61. $^{11}B\{^1H\}$ NMR (192 MHz, 299 K, CD_2Cl_2) spectrum of compound **9b**.

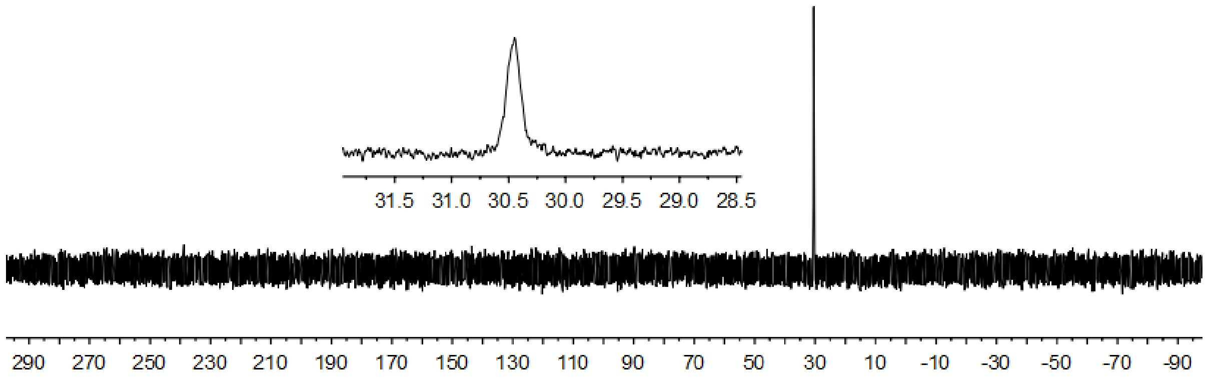

Figure S62. $^{31}P\{^1H\}$ NMR (243 MHz, 299 K, CD_2Cl_2) spectrum of compound **9b**.

Crystals suitable for the X-ray crystal structure analysis were obtained from two-layer diffusion of pentane to a solution of the isolated white powder in CH_2Cl_2 at room temperature.

X-ray crystal structure analysis of compound 9b (erk9381): A colorless plate-like specimen of $C_{40}H_{30}BF_{10}P$, approximate dimensions 0.080 mm x 0.120 mm x 0.240 mm, was used for the X-ray crystallographic analysis. The X-ray intensity data were measured. A total of 1732 frames were collected. The total exposure time was 20.63 hours. The frames were integrated with the Bruker SAINT software package using a wide-frame algorithm. The integration of the data using a triclinic unit cell yielded a total of 57658 reflections to a maximum θ angle of 66.95° (0.84 Å resolution), of which 12835 were independent (average redundancy 4.492, completeness = 98.8%, R_{int} = 7.71%, R_{sig} = 6.05%) and 9341 (72.78%) were greater than 2σ(F^2). The final cell constants of a = 12.4783(5) Å, b = 15.7635(6) Å, c = 20.7617(7) Å, α = 102.312(2)°, β = 93.184(2)°, γ = 112.263(2)°, volume = 3650.3(2) $Å^3$, are based upon the refinement of the XYZ-centroids of 8015 reflections above 20 σ(I) with 6.263° < 2θ < 133.1°. Data were corrected for absorption effects using the multi-scan method (SADABS). The ratio of minimum to maximum apparent transmission was 0.831. The calculated minimum and maximum transmission coefficients (based on crystal size) are 0.7340 and 0.8980. The structure was solved and refined using the Bruker SHELXTL Software Package, using the space group *P*-1, with Z = 4

for the formula unit, $C_{40}H_{30}BF_{10}P$. The final anisotropic full-matrix least-squares refinement on F^2 with 956 variables converged at R1 = 4.75%, for the observed data and wR2 = 12.27% for all data. The goodness-of-fit was 1.035. The largest peak in the final difference electron density synthesis was 0.383 $e^-/Å^3$ and the largest hole was -0.310 $e^-/Å^3$ with an RMS deviation of 0.057 $e^-/Å^3$. On the basis of the final model, the calculated density was 1.351 g/cm^3 and F(000), 1520 e^-. CCDC number: 1922910.

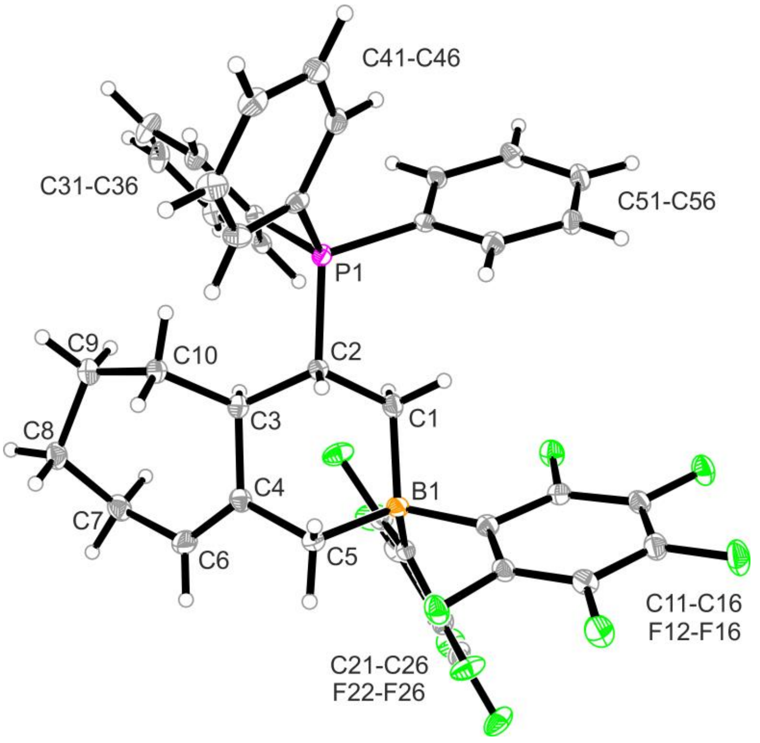

Figure S63. Crystal structure of compound **9b** (thermal ellipsoids: 30% probability. Only one molecule (molecule A) of two found in the asymmetric unit is shown).

M) Synthesis of compound **9c**

Scheme S15.

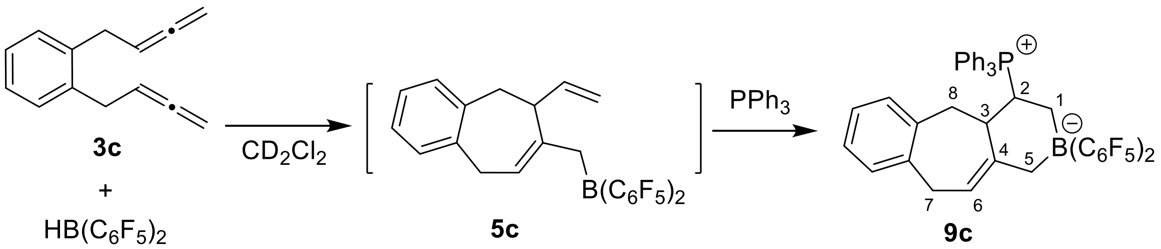

A suspension of $HB(C_6F_5)_2$ (103.8 mg, 0.30 mmol) in CH_2Cl_2 (3 mL) was added to a solution of bisallene **3c** (54.6 mg, 0.30 mmol) in CH_2Cl_2 (2 mL) at room temperature. After the resulting mixture was stirred for 30 minutes at room temperature, a solution of PPh_3 (76.8 mg, 0.3 mmol) in CH_2Cl_2 (2 mL) was added. The mixture was stirred overnight at room temperature. Then all volatilities were removed in vacuo and the remaining residue was triturated by addition of pentane (1 mL × 3). The

residual solid was dried in vacuo giving compound **9c** (180 mg, 0.23 mmol 77%) as a white solid.

HRMS (ESI) m/z: calc. for $C_{44}H_{30}BF_{10}P$ $[M+H]^+$: 791.2099. Found: 791.2096

NMR data of compound **9c**:

^{1}H NMR (600 MHz, 299 K, CD_2Cl_2): δ ^{1}H: [7.83 (m, 6H, *o*), 7.78 (m, 3H, *p*), 7.68 (m, 6H, *m*)](PPh_3), [6.99 (td, $^3J_{HH}$ = 7.4 Hz, $^4J_{HH}$ = 1.4 Hz), 6.95 (dd, $^3J_{HH}$ = 7.4 Hz, $^4J_{HH}$ = 1.4 Hz), 6.92 (td, $^3J_{HH}$ = 7.4 Hz, $^4J_{HH}$ = 1.3 Hz), 6.36 (dd, $^3J_{HH}$= 7.4 Hz, $^4J_{HH}$ = 1.3 Hz)](each 1H, $CH^{phenylene}$), 5.47 (m, 1H, 6-CH=), 3.81 (m, 1H, 2-CH), [3.63 (dm, $^2J_{HH}$ = 15.5 Hz), 2.74 (dd, $^2J_{HH}$ = 15.5 Hz, $^3J_{HH}$ = 3.5 Hz)](each 1H, 7-CH_2), [3.14 (t, $^2J_{HH}$ ~ $^3J_{HH}$ = 13.7 Hz), 2.40 (m)](each 1H, 8-CH_2), 2.40 (m, 1H, 3-CH), [2.17 (d, $^2J_{HH}$ = 12.8 Hz), 1.29 (m)](each 1H, 5-CH_2), [1.94 (t, $^2J_{HH}$ ~ $^3J_{HH}$ = 12.5 Hz), 0.81 (dt, $^2J_{HH}$ ~ $^3J_{PH}$ = 12.5 Hz, $^3J_{HH}$ = 9.0 Hz)](each 1H, 1-CH_2).

^{13}C{^{1}H} NMR (151 MHz, 299 K, CD_2Cl_2): δ ^{13}C: 145.2 (d, $^3J_{PC}$ = 11.3 Hz, 4-C=), [144.9, 138.3]($C^{phenylene}$), [134.5 (*p*), 134.1 (d, $^2J_{PC}$ = 8.1 Hz, *o*), 130.3 (d, $^3J_{PC}$ = 8.1 Hz, *m*), 121.4 (d, $^1J_{PC}$ = 78.8 Hz, *i*)](PPh_3), [127.9, 126.6, 126.5, 126.0]($CH^{phenylene}$), 118.9 (6-CH=), 45.7 (3-CH), 41.2 (d, $^1J_{PC}$ = 25.0 Hz, 2-CH), 40.1 (8-CH_2), 38.4 (br, 5-CH_2), 32.5 (7-CH_2), 26.5 (br, 1-CH_2), [C_6F_5 not listed].

^{19}F NMR (564 MHz, 299 K, CD_2Cl_2): δ ^{19}F: [–133.2, –134.2](each m, each 2F, *o*-C_6F_5), [–163.8, –164.5](each t, $^3J_{FF}$ = 20.3 Hz, each 1F, *p*-C_6F_5), [–166.6, –167.1](each m, each 2F, *m*-C_6F_5).

^{11}B{^{1}H} NMR (192 MHz, 299 K, CD_2Cl_2): δ ^{11}B: –12.2 ($\nu_{1/2}$ ~ 90 Hz).

^{31}P{^{1}H} NMR (243 MHz, 299 K, CD_2Cl_2): δ ^{31}P: 28.8 ($\nu_{1/2}$ ~ 25 Hz).

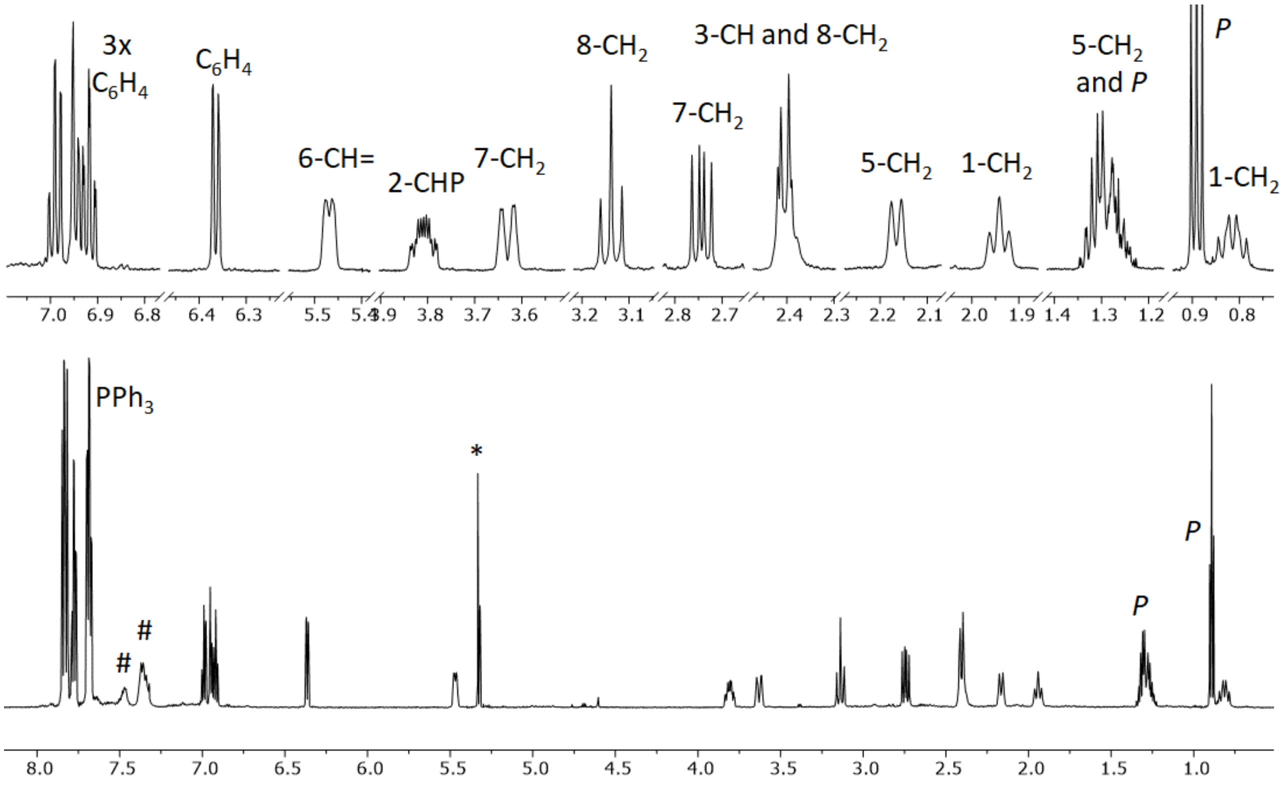

Figure S64. ^{1}H NMR (600 MHz, 299 K, CD_2Cl_2*) spectrum of compound **9c**.
[P: pentane, #: unidentified impurity]

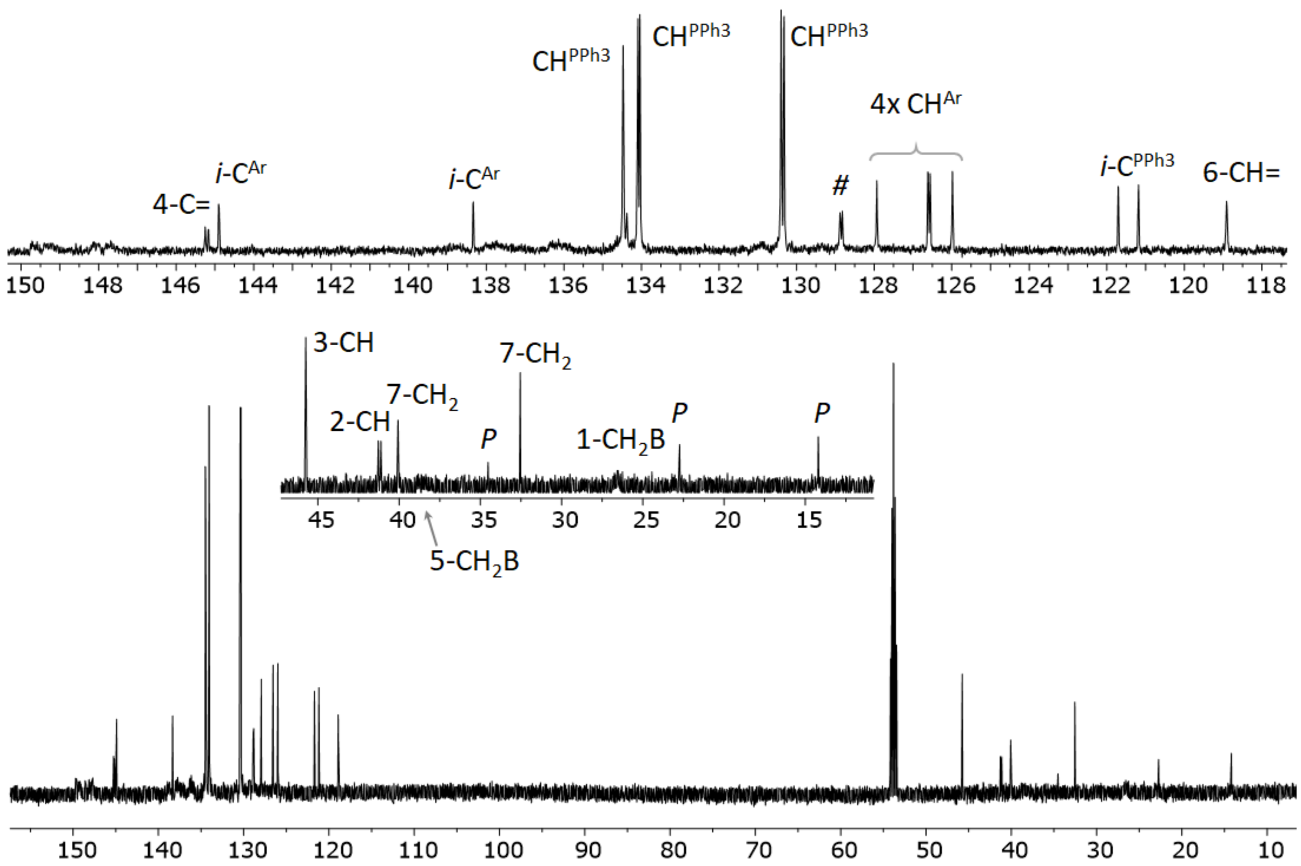

Figure S65. $^{13}C\{^{1}H\}$ NMR (151 MHz, 299 K, CD_2Cl_2) spectrum of compound **9c**.
[P: pentane, #: unidentified impurity]

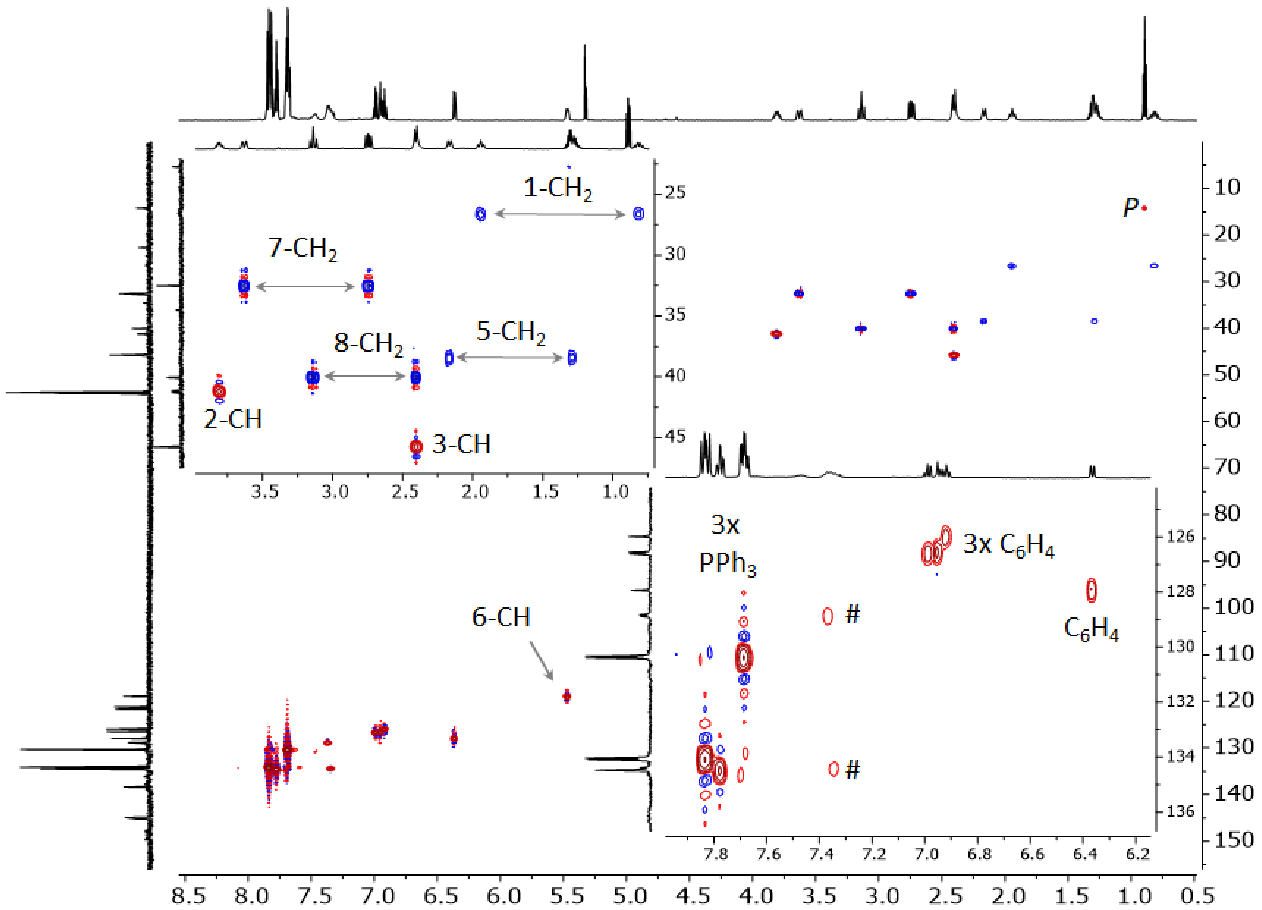

Figure S66. ^{1}H,^{13}C GHSQC (500/126 MHz, CD_2Cl_2, 299K) spectrum of compound **9c**.
[P: pentane, #: unidentified impurity]

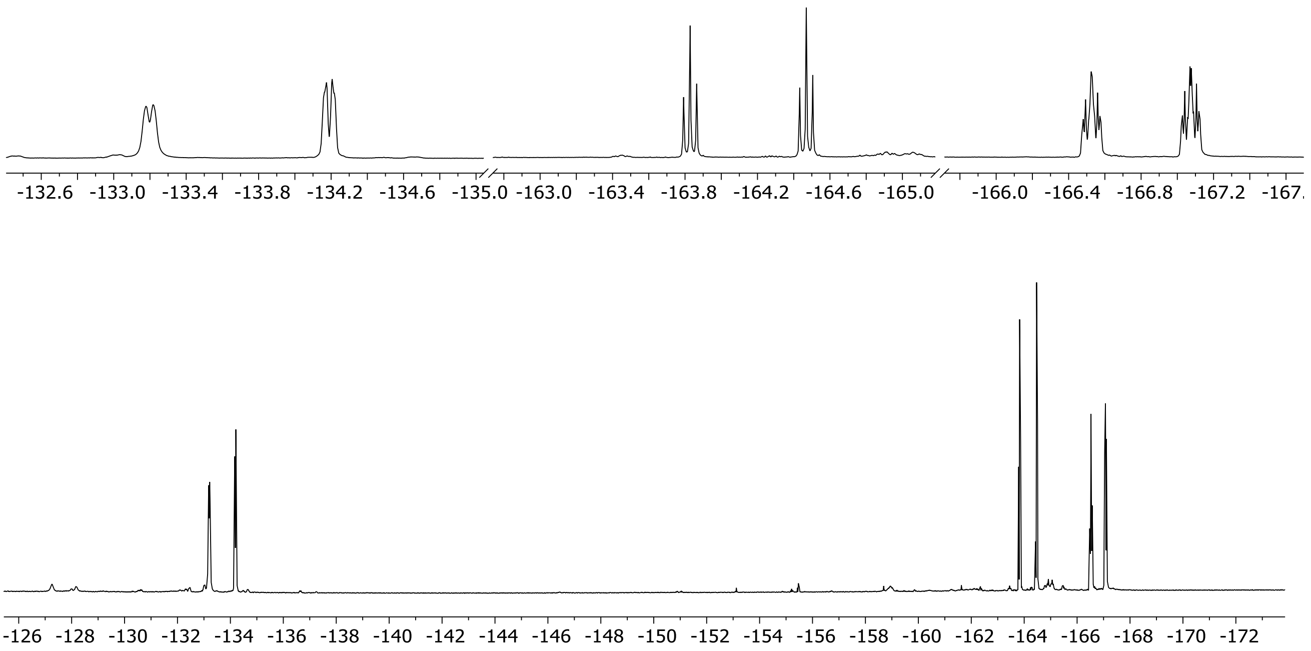

Figure S67. ^{19}F NMR (564 MHz, 299 K, CD_2Cl_2) spectrum of compound **9c**.

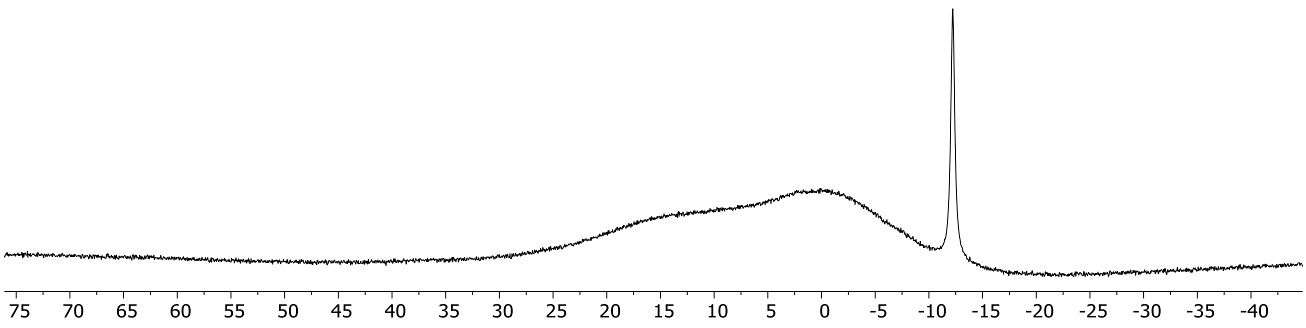

Figure S68. $^{11}B\{^{1}H\}$ NMR (192 MHz, 299 K, CD_2Cl_2) spectrum of compound **9c**.

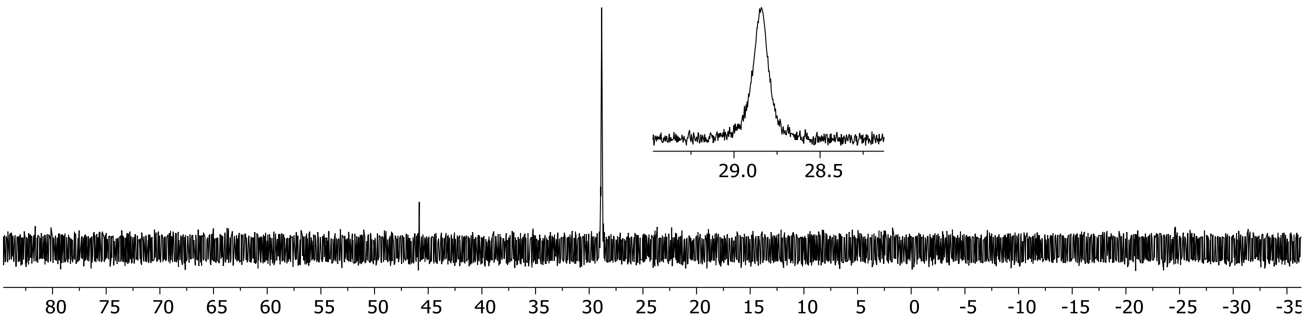

Figure S69. $^{31}P\{^{1}H\}$ NMR (243 MHz, 299 K, CD_2Cl_2) spectrum of compound **9c**.

Crystals suitable for the X-ray crystal structure analysis were obtained from two-layer diffusion of pentane to a solution of the isolated white powder in CH_2Cl_2 at room temperature.

X-ray crystal structure analysis of compound 9c (erk9636): A colorless plate-like specimen of $C_{44}H_{30}BF_{10}P$, approximate dimensions 0.033 mm x 0.106 mm x 0.148 mm, was used for the X-ray crystallographic analysis. The X-ray intensity data were measured. A total of 1084 frames were collected. The total exposure time was 18.07 hours. The frames were integrated with the Bruker SAINT software package using a narrow-frame algorithm. The integration of the data using a triclinic unit cell yielded a total of 31008 reflections to a maximum θ angle of 26.78° (0.79 Å resolution), of which 7446 were independent (average redundancy 4.164, completeness = 99.4%, R_{int} = 4.66%, R_{sig} = 4.07%) and 6191 (83.15%) were greater than $2\sigma(F^2)$. The final cell constants of a = 8.3209(3) Å, b = 11.3190(5) Å, c = 19.7803(8) Å, α = 99.3990(10)°, β = 98.0400(10)°, γ = 104.0510(10)°, volume = 1751.34(12) $Å^3$, are based upon the refinement of the XYZ-centroids of 8323 reflections above 20 σ(I) with 4.710° < 2θ < 53.37°. Data were corrected for absorption effects using the multi-scan method (SADABS). The ratio of minimum to maximum apparent transmission was 0.942. The calculated minimum and maximum transmission coefficients (based on crystal size) are 0.9760 and 0.9950. The structure was solved and refined using the Bruker SHELXTL Software Package, using the space group *P*-1, with Z = 2 for the formula unit, $C_{44}H_{30}BF_{10}P$. The final anisotropic full-matrix least-squares refinement on F^2 with 505 variables converged at R1 = 5.36%, for the observed data and wR2 = 11.07% for all data. The goodness-of-fit was 1.106. The largest peak in the final difference electron density synthesis was 0.440 $e^-/Å^3$ and the largest hole was -0.381 $e^-/Å^3$ with an RMS deviation of

0.059 $e^-/Å^3$. On the basis of the final model, the calculated density was 1.499 g/cm^3 and F(000), 808 e^-. CCDC number: 1957134.

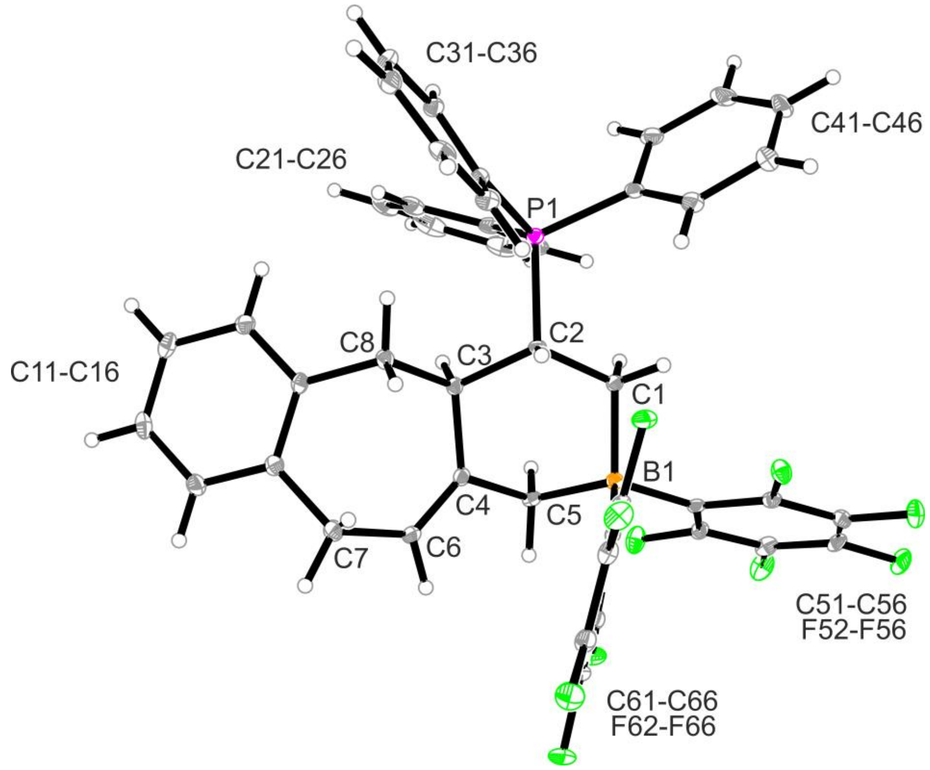

Figure S70. Crystal structure of compound **9c** (thermal ellipsoids: 30% probability).

N) Generation and synthesis of compound **12a**

Experiment 1: (NMR scale, *in situ* generation of compound **12a**)

Scheme S16.

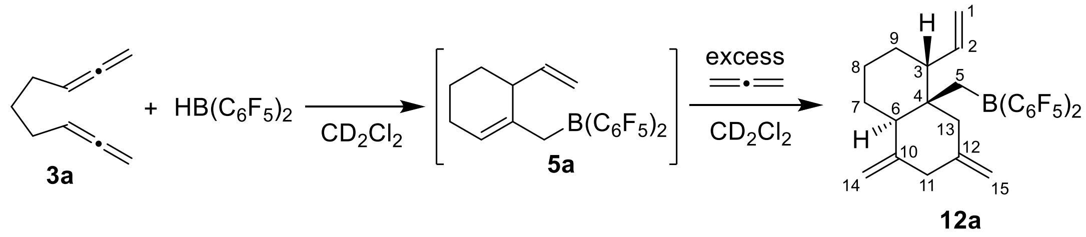

<u>*Step 1:*</u> A suspension of $HB(C_6F_5)_2$ (34.6 mg, 0.10 mmol) in CD_2Cl_2 (0.3 mL) was added to a solution of bisallene **3a** (12.0 mg, 0.10 mmol) in CD_2Cl_2 (0.3 mL) at room temperature in a Young NMR tube. The resulting reaction mixture was characterized by NMR experiments after 2 h at room temperature.

<u>*Step 2:*</u> After evacuating the Young NMR tube carefully, the reaction mixture was exposed to allene gas at room temperature. The resulting reaction mixture was stored at room temperature for ca. 24 h and then characterized by NMR experiments. [Comment: only one diastereomer could be clearly characterized: ca. 90 mol%. There are some minor as yet unidentified compounds: overall ca. 10 mol%]

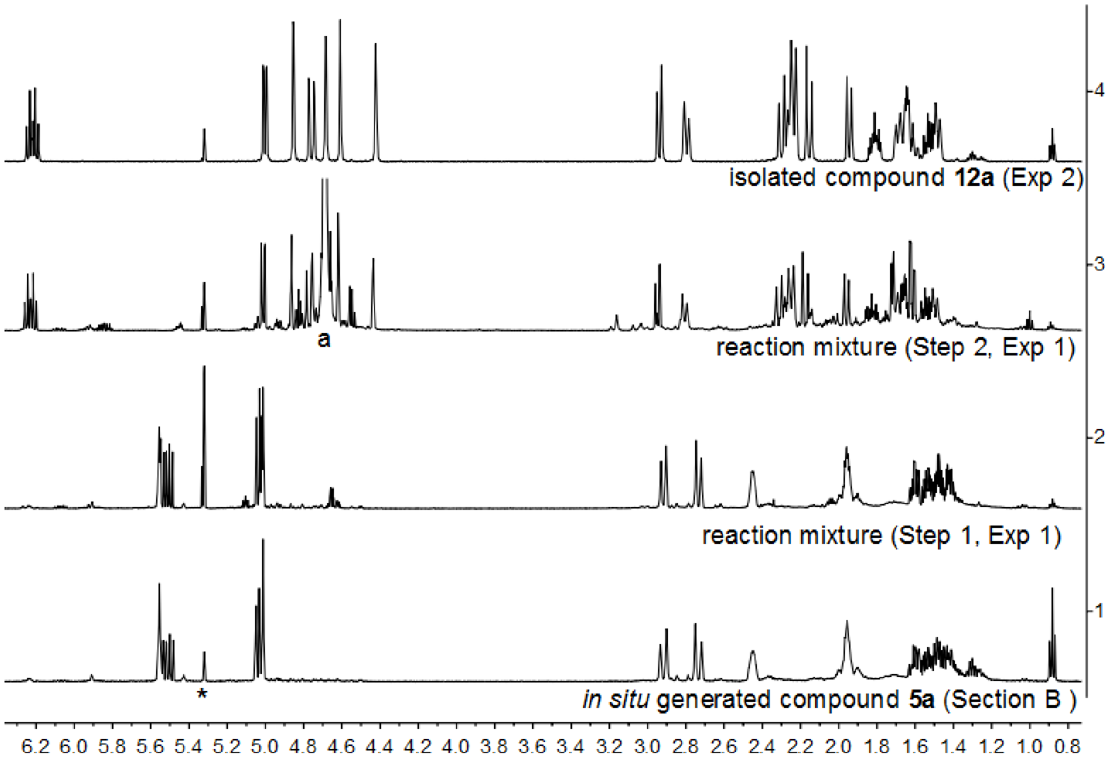

Figure S71. ^{1}H NMR (600 MHz, 299 K, CD_2Cl_2*) spectra of (1) the in situ generated compound **5a** (Section B), (2) the reaction mixture as described in the Step 1 of Experiment 1, (3) the reaction mixture as described in the Step 2 of Experiment 1 and (4) the isolated compound **12a**.

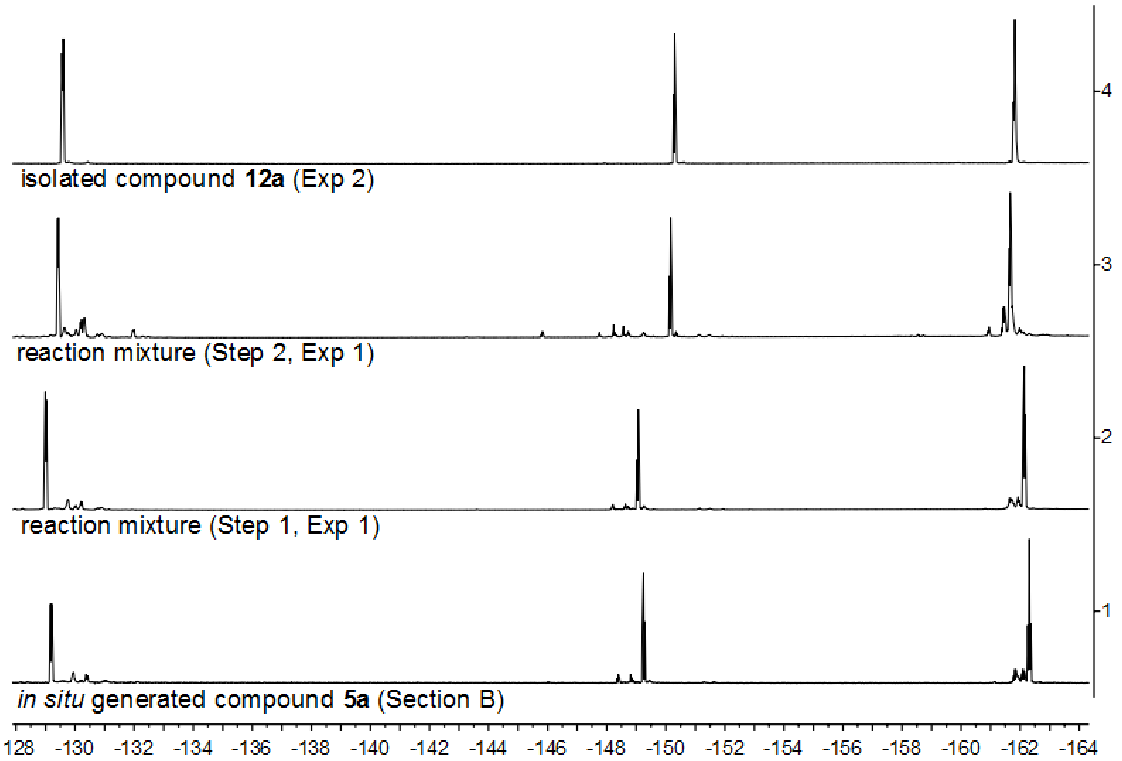

Figure S72. ^{19}F NMR (564 MHz, 299 K, CD_2Cl_2*) spectra of (1) the in situ generated compound **5a** (Section B), (2) the reaction mixture as described in the Step 1 of Experiment 1, (3) the reaction mixture as described in the Step 2 of Experiment 1 and (4) the isolated compound **12a**.

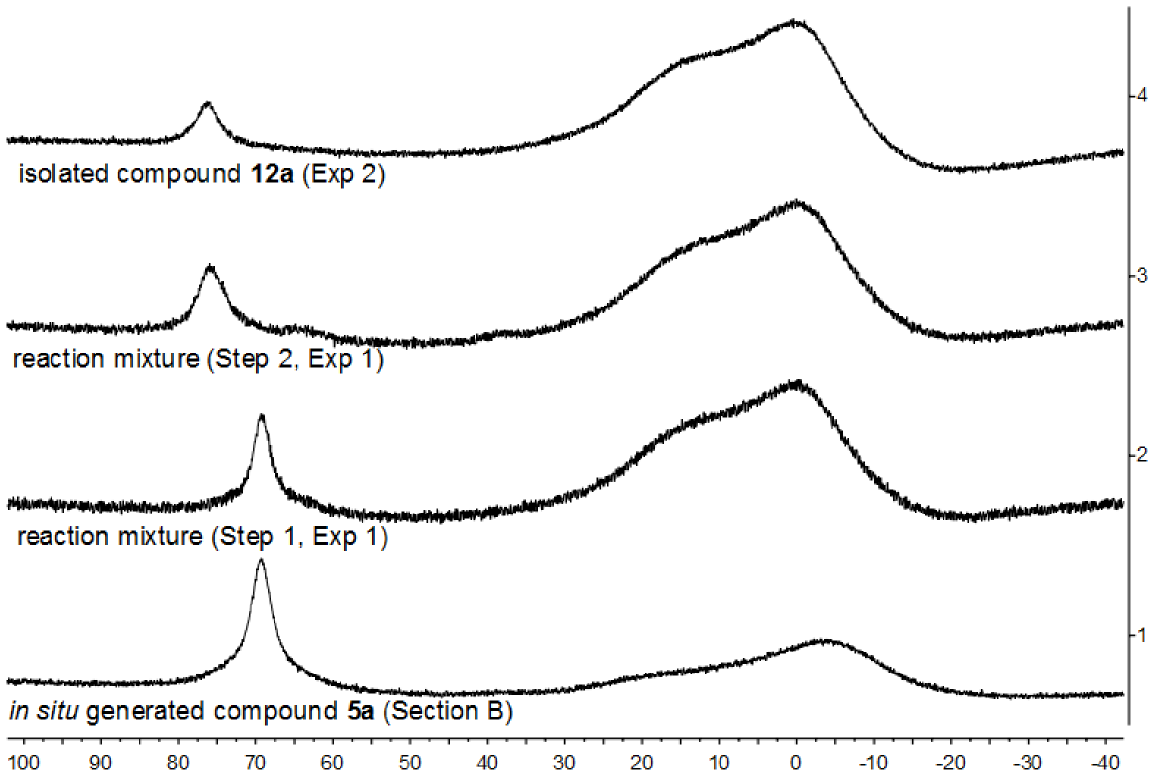

Figure S73. $^{11}B\{^{1}H\}$ NMR (192 MHz, 299 K, CD_2Cl_2) spectra of (1) the in situ generated compound **5a** (Section B), (2) the reaction mixture as described in the Step 1 of Experiment 1, (3) the reaction mixture as described in the Step 2 of Experiment 1 and (4) the isolated compound **12a**.

Experiment 2: (preparative scale, isolation and characterization of compound **12a**)

Scheme S17.

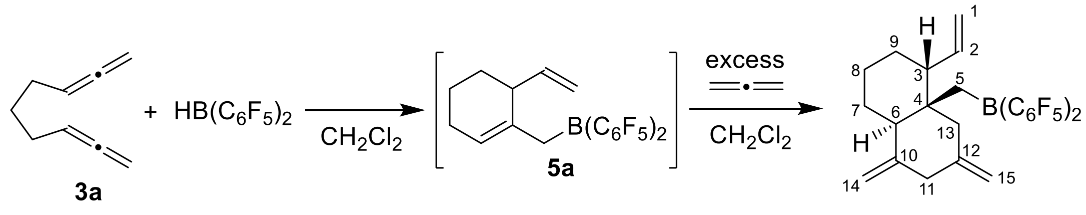

A suspension of $HB(C_6F_5)_2$ (208 mg, 0.60 mmol) in CH_2Cl_2 (2 mL) was added to a solution of bisallene **3a** (80.0 mg, 0.66 mmol) in CD_2Cl_2 (2 mL) at room temperature in a Schlenk tube and the resulting reaction mixture was stirred for 1h at room temperature. After evacuating the Schlenk tube carefully, the reaction mixture was exposed to allene gas at room temperature. The resulting reaction mixture was stirred at room temperature for ca. 24 h. Then all the volatile were removed in vacuo and the residue was dissolved in pentane (2 mL). After storage of the obtained solution at -35 °C for 3 d, colorless crystals precipitated from the solution. Part of the crystals were used for the X-ray crystal structure analysis, the rest was carefully washed with cold (-35 °C) pentane (0.5 mL × 1) and dried in vacuo giving compound **12a** (118 mg, 0.22 mmol, 37%) as a white crystalline material.

Anal. Calc. for $C_{27}H_{21}BF_{10}$: C, 59.37; H, 3.88. Found: C, 59.34; H, 3.87.

NMR data of the isolated compound **12a**:

^{1}H NMR (600 MHz, 299 K, CD_2Cl_2): δ ^{1}H: 6.22 (dt, $^{3}J_{HH}$ = 16.8, 10.0 Hz, 1H, CH=), [5.00 (dd, $^{3}J_{HH}$ = 16.8 Hz, $^{2}J_{HH}$ = 2.0 Hz), 4.76 (dd, $^{3}J_{HH}$ = 10.0 Hz, $^{2}J_{HH}$ = 2.0 Hz)](each 1H, 1-CH_2=], 4.85/4.61 (each m, each 1H, 14-CH_2=), 4.68/4.42 (each m, each 1H, 15-CH_2=), 2.94/2.80 (each dm, $^{2}J_{HH}$ = 14.2 Hz, each 1H, 11-CH_2), 2.30/2.15 (each d, $^{2}J_{HH}$ = 17.2 Hz, each 1H, BCH_2), 2.26 (m, 1H, 3-CH), 2.24/1.95 (each dm, $^{2}J_{HH}$ = 13.7 Hz, each 1H, 13-CH_2), 2.23 (m, 1H, 6-CH), 1.81/1.48 (each m, each 1H, 9-CH_2), 1.69/1.52 (each m, each 1H, 7-CH_2), 1.64 (m, 2H, 8-CH_2).

^{13}C{^{1}H} NMR (151 MHz, 299 K, CD_2Cl_2): δ ^{13}C: 149.0 (10-C=), 146.3 (12-C=), 145.9 (dm, $^{1}J_{FC}$ ~ 250 Hz, C_6F_5), 143.0 (dm, $^{1}J_{FC}$ ~ 260 Hz, C_6F_5), 139.0 (CH=), 137.9 (dm, $^{1}J_{FC}$ ~ 250 Hz, C_6F_5), 116.5 (1-CH_2=), 115.8 (br, i-C_6F_5), 110.0 (15-CH_2=), 108.2 (14-CH_2=), 49.1 (3-CH), 46.6 (13-CH_2), [45.68, 45.65](6-CH,11-CH_2), 45.3 (C), 36.7 (br, BCH_2), 28.9 (9-CH_2), 24.8 (7-CH_2), 21.0 (8-CH_2).

^{19}F NMR (564 MHz, 299 K, CD_2Cl_2): δ ^{19}F: −129.6 (m, 2F, *o*), −150.3 (m, 1F, *p*), −161.8 (m, 2F, *m*)(C_6F_5)[$\Delta\delta^{19}F_{m,p}$ = 11.5].

^{11}B{^{1}H} NMR (192 MHz, 299 K, CD_2Cl_2): δ ^{11}B: 76.4 ($\nu_{1/2}$ ~ 800 Hz).

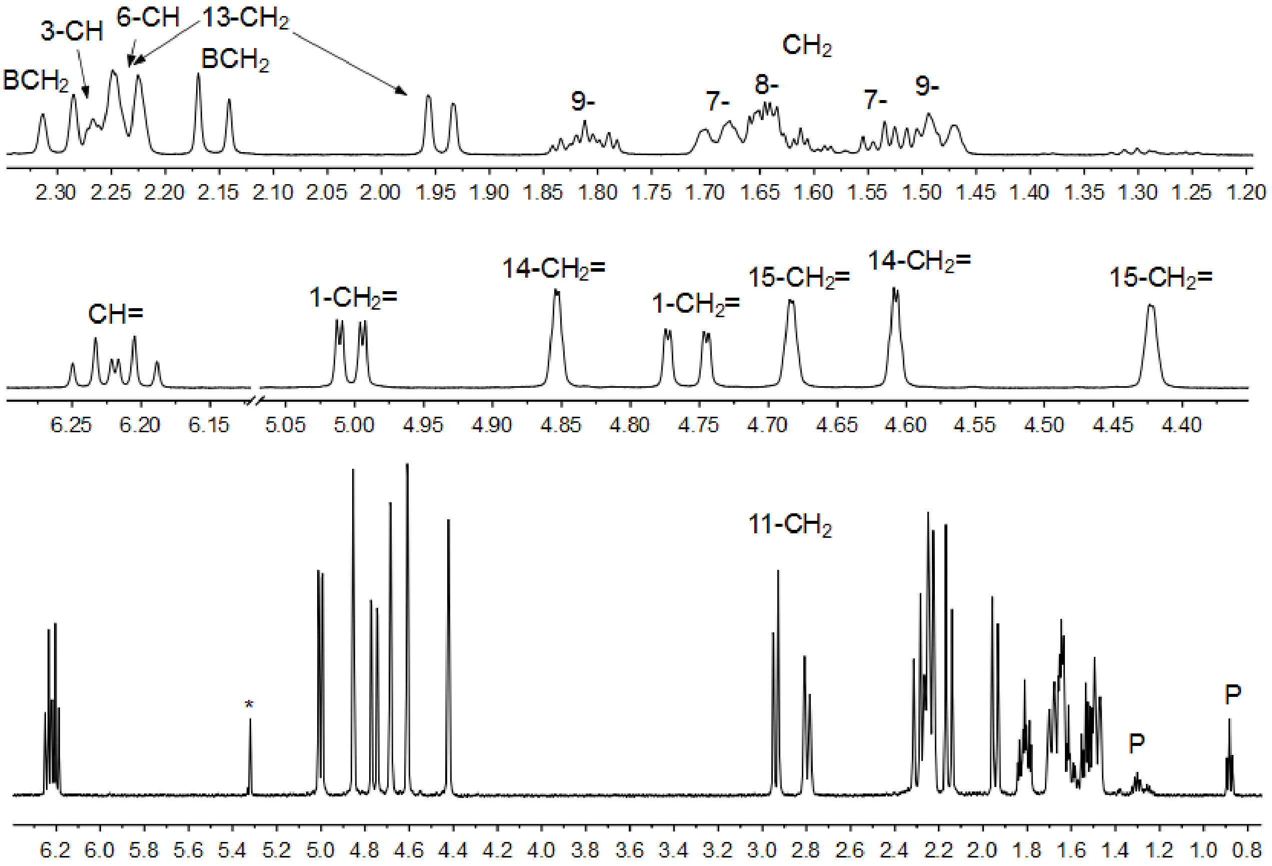

Figure S74. ^{1}H NMR (600 MHz, 299 K, CD_2Cl_2*) spectrum of compound **12a**. [P: pentane]

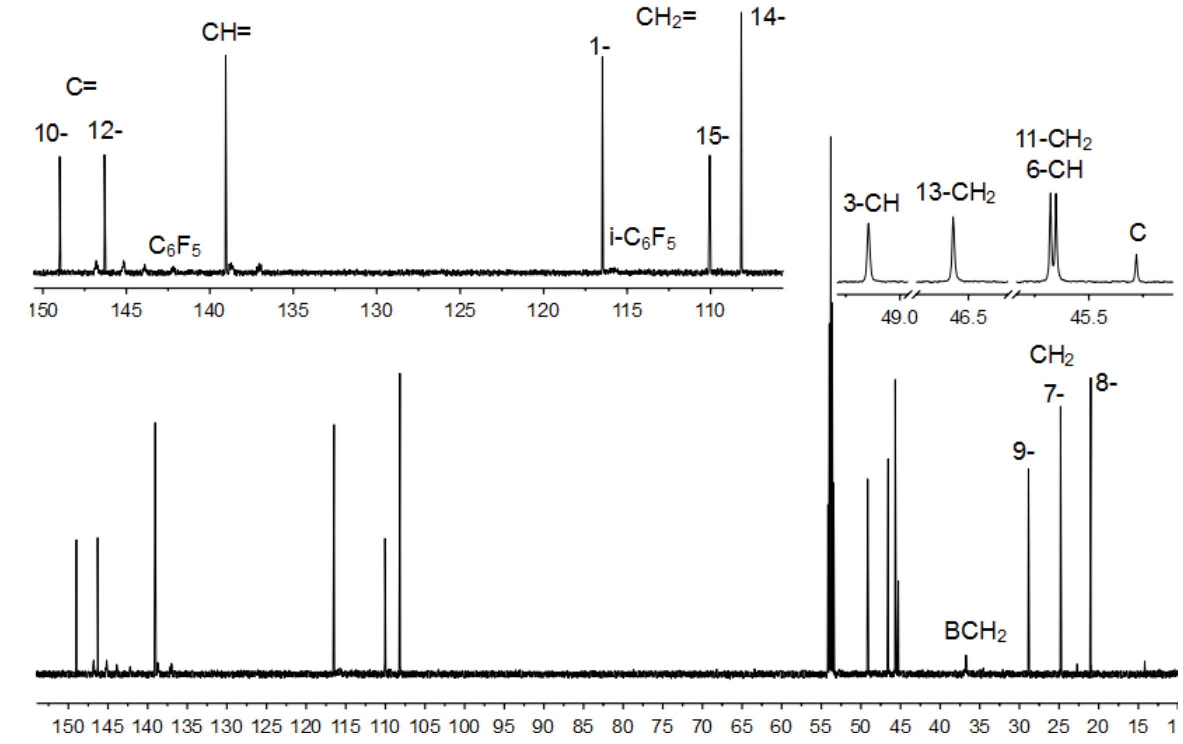

Figure S75. $^{13}C\{^{1}H\}$ NMR (151 MHz, 299 K, CD_2Cl_2) spectrum of compound **12a**.

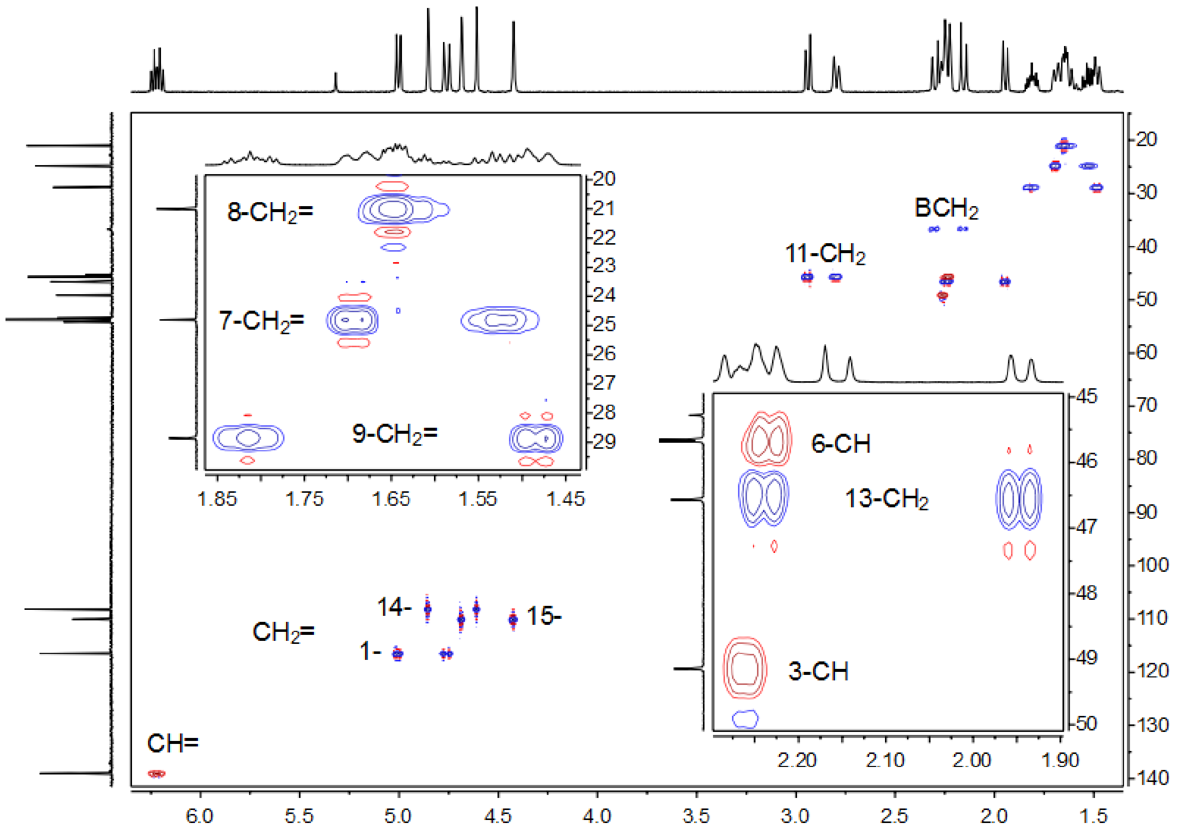

Figure S76. $^{1}H,^{13}C$ GHSQC (600/151 MHz, CD_2Cl_2, 299K) spectrum of compound **12a**.

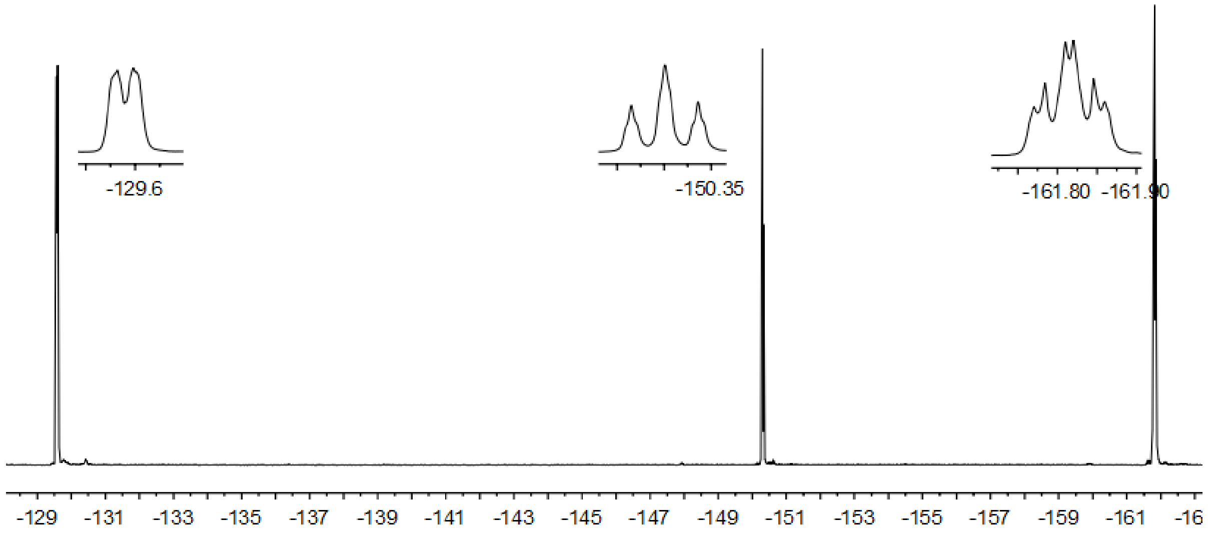

Figure S77. ^{19}F NMR (564 MHz, 299 K, CD_2Cl_2) spectrum of compound **12a**.

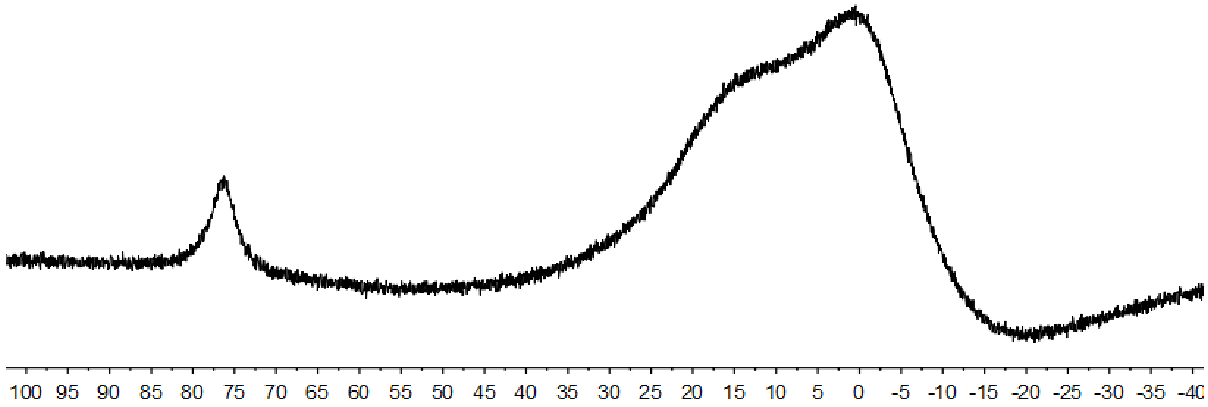

Figure S78. $^{11}B\{^1H\}$ NMR (192 MHz, 299 K, CD_2Cl_2) spectrum of compound **12a**.

X-ray crystal structure analysis of compound 12a (erk9518): A colorless plate-like specimen of $C_{27}H_{21}BF_{10}$, approximate dimensions 0.070 mm x 0.120 mm x 0.200 mm, was used for the X-ray crystallographic analysis. The X-ray intensity data were measured. A total of 1551 frames were collected. The total exposure time was 20.20 hours. The frames were integrated with the Bruker SAINT software package using a wide-frame algorithm. The integration of the data using a monoclinic unit cell yielded a total of 11802 reflections to a maximum θ angle of 66.74° (0.84 Å resolution), of which 4116 were independent (average redundancy 2.867, completeness = 97.5%, R_{int} = 4.39%, R_{sig} = 4.40%) and 3248 (78.91%) were greater than 2σ(F^2). The final cell constants of a = 6.4255(4) Å, b = 17.4610(10) Å, c = 21.2763(13) Å, β = 95.037(4)°, volume = 2377.9(2) Å^3, are based upon the refinement of the XYZ-centroids of 9956 reflections above 20 σ(I) with 6.56° < 2θ < 133.3°. Data were corrected for absorption effects using the multi-scan method (SADABS). The ratio of minimum to maximum apparent transmission was 0.842. The calculated minimum and maximum transmission coefficients (based on crystal size) are 0.7870 and 0.9170. The structure was solved and refined using the Bruker SHELXTL Software Package, using the space group $P2_1/c$, with Z = 4 for the formula unit, $C_{27}H_{21}BF_{10}$. The final anisotropic full-matrix least-squares refinement on F^2 with 343 variables

converged at R1 = 4.02%, for the observed data and wR2 = 10.07% for all data. The goodness-of-fit was 1.056. The largest peak in the final difference electron density synthesis was 0.307 $e^-/Å^3$ and the largest hole was -0.215 $e^-/Å^3$ with an RMS deviation of 0.049 $e^-/Å^3$. On the basis of the final model, the calculated density was 1.526 g/cm^3 and F(000), 1112 e^-. CCDC number: 1922911.

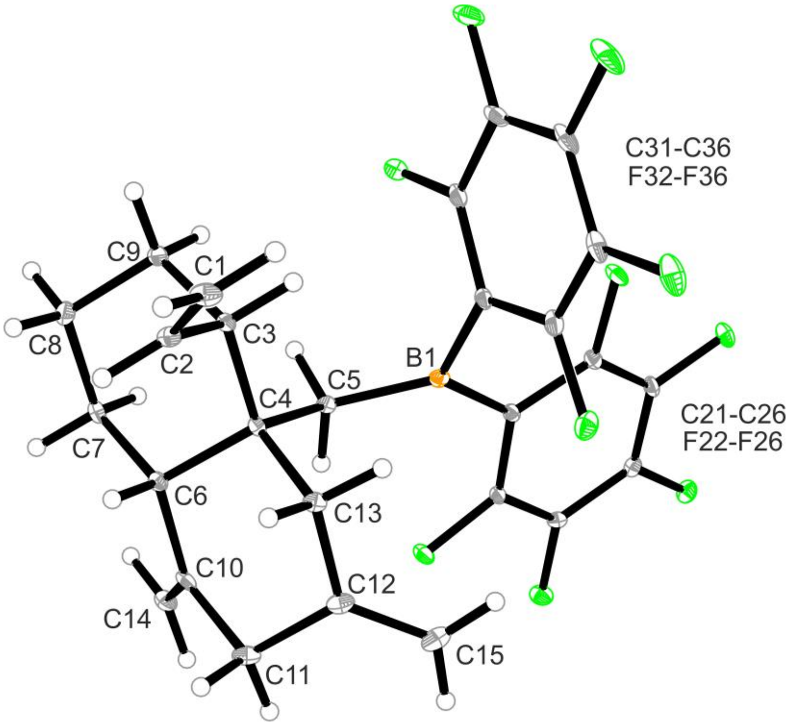

Figure S79. Crystal structure of compound **12a** (thermal ellipsoids: 15% probability).

O) Synthesis of compounds **13a** and **14a**

Experiment 1: (isolation and characterizations of compound **13a**)

Scheme S18.

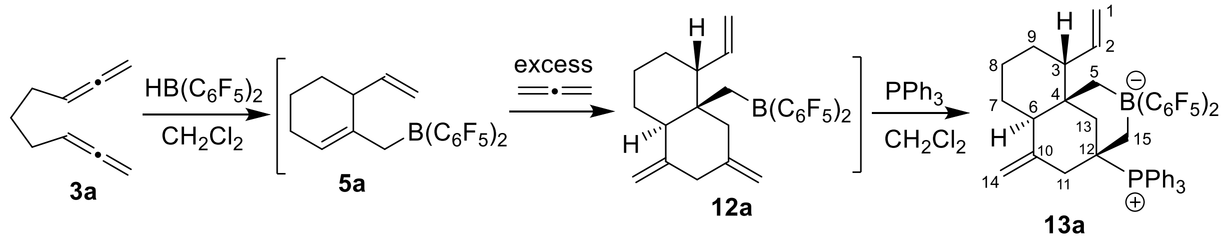

A suspension of $HB(C_6F_5)_2$ (103.8 mg, 0.30 mmol) in CH_2Cl_2 (1 mL) was added to a solution of bisallene **3a** (12.0 mg, 0.10 mmol) in CH_2Cl_2 (1 mL) at room temperature in a Schlenk tube. After evacuating the Schlenk NMR tube carefully, the reaction mixture was exposed to allene gas at room temperature. The resulting reaction mixture was stirred at room temperature for ca. 24 h. Then PPh_3 (78.9 mg, 0.30 mmol) was added to the reaction mixture. Subsequently, all the volatile were removed in vacuo. The residue was washed with pentane (1 mL × 3) and dried in vacuo giving compound **13a** (135 mg, 0.17 mmol, 56%) as a white powder.

Anal. Calc. for $C_{45}H_{36}BF_{10}P$: C, 66.85; H, 4.49. Found: C, 66.81; H, 5.03.

NMR data of compound **13a** (273K)

[*Comment*: at room temperature we observed a mixture of compounds **13a**, **12a**, **14a** (later) and PPh_3 (see below, Experiment 3)]

^{1}H NMR (600 MHz, 273 K, CD_2Cl_2): δ ^{1}H: [7.69, 7,59, 7.51](each br, 15H, Ph), 6.05 (dt, $^3J_{HH}$ = 16.7, 10.1 Hz, 1H, CH=), [5.03 (dd, $^3J_{HH}$ = 16.7 Hz, $^2J_{HH}$ = 2.7 Hz), 4.86 (dd, $^3J_{HH}$ = 10.1 Hz, $^2J_{HH}$ = 2.7 Hz)](each 1H, 1-CH_2=), 4.82/4.57 (each m, each 1H, 14-CH_2=), 2.58/2.54 (each m, each 1H, 11-CH_2), 2.50 (m, 1H, 3-CH), 2.08/1.31 (each m, each 1H, 9-CH_2), 2.07/1.94 (each m, each 1H, 15-CH_2) 1.93/1.44 (each m, each 1H, 13-CH_2), 1.79 (m, 1H, 6-CH), [1.45 (2H), 1.44/1.36 (each 1H)](7,8-CH_2), 1.34/0.34 (each m, each 1H, 5-CH_2).

^{13}C{^{1}H} NMR (151 MHz, 273 K, CD_2Cl_2): δ ^{13}C: 146.8 (d, $^3J_{PC}$ = 15.3 Hz, C=), 140.9 (CH=), [135.1, 134.1, 129.6](each br, Ph), 119.5 (dm, $^1J_{PC}$ = 79.4 Hz, i-Ph), 114.8 (1-CH_2=), 110.0 (14-CH_2=), 50.8 (br, 3-CH), 47.4 (11-CH_2), 46.3 (6-CH), 44.0 (d, $^1J_{PC}$ = 31.5 Hz, PC), 40.1 (d, $^3J_{PC}$ = 13.3 Hz, 4-C), 38.4 (13-CH_2), 27.4 (9-CH_2), 25.4 (br, 15-CH_2), [24.6, 20.9](7,8-CH_2), 23.9 (br, 5-CH_2), [C_6F_5 not listed].

^{19}F NMR (564 MHz, 273 K, CD_2Cl_2): δ ^{19}F: –132.3/–132.6 (each m, each 2F, *o*), –165.0/–165.3 (each t, $^3J_{FF}$ = 20.3 Hz, each 1F, *p*), –166.9/–167.2 (each m, 2F, *m*)(C_6F_5).

^{11}B{^{1}H} NMR (192 MHz, 273 K, CD_2Cl_2): δ ^{11}B: –15.1 ($\nu_{1/2}$ ~ 130 Hz).

^{31}P{^{1}H} NMR (243 MHz, 273 K, CD_2Cl_2): δ ^{31}P: 31.1 ($\nu_{1/2}$ ~ 2 Hz).

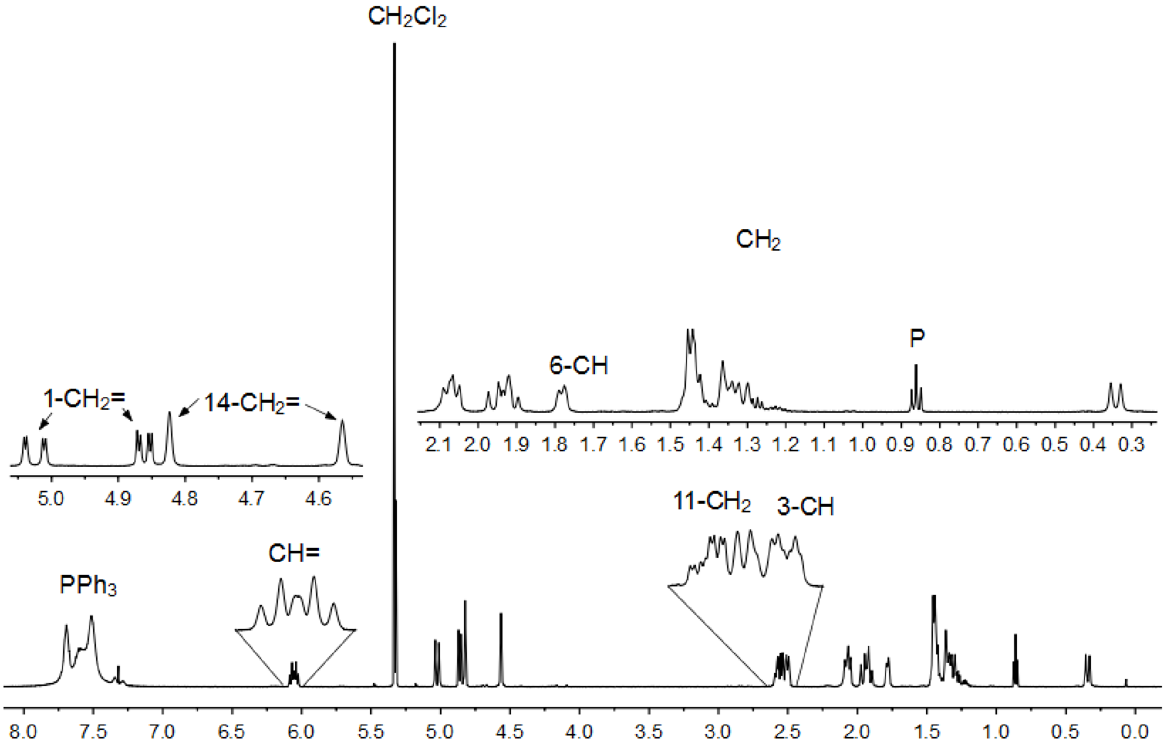

Figure S80. ^{1}H NMR (600 MHz, 273 K, CD_2Cl_2) spectrum of compound **13a**. [P: pentane]

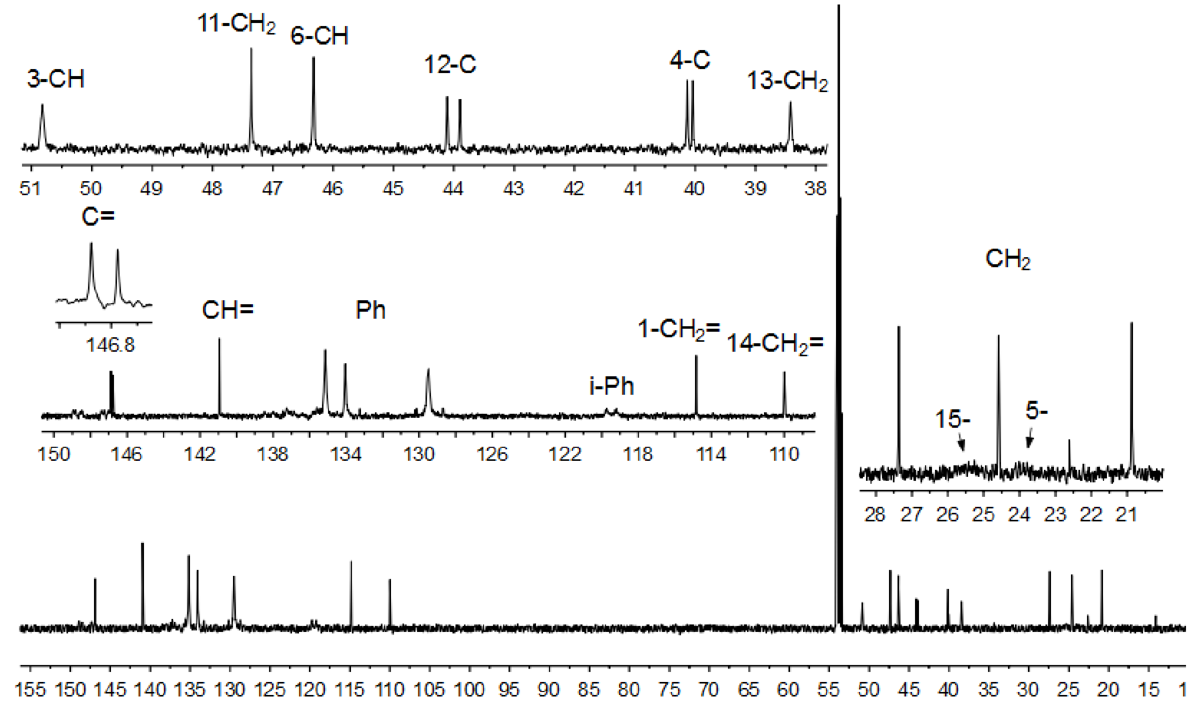

Figure S81. $^{13}C\{^{1}H\}$ NMR (151 MHz, 273 K, CD_2Cl_2) spectrum of compound **13a**.

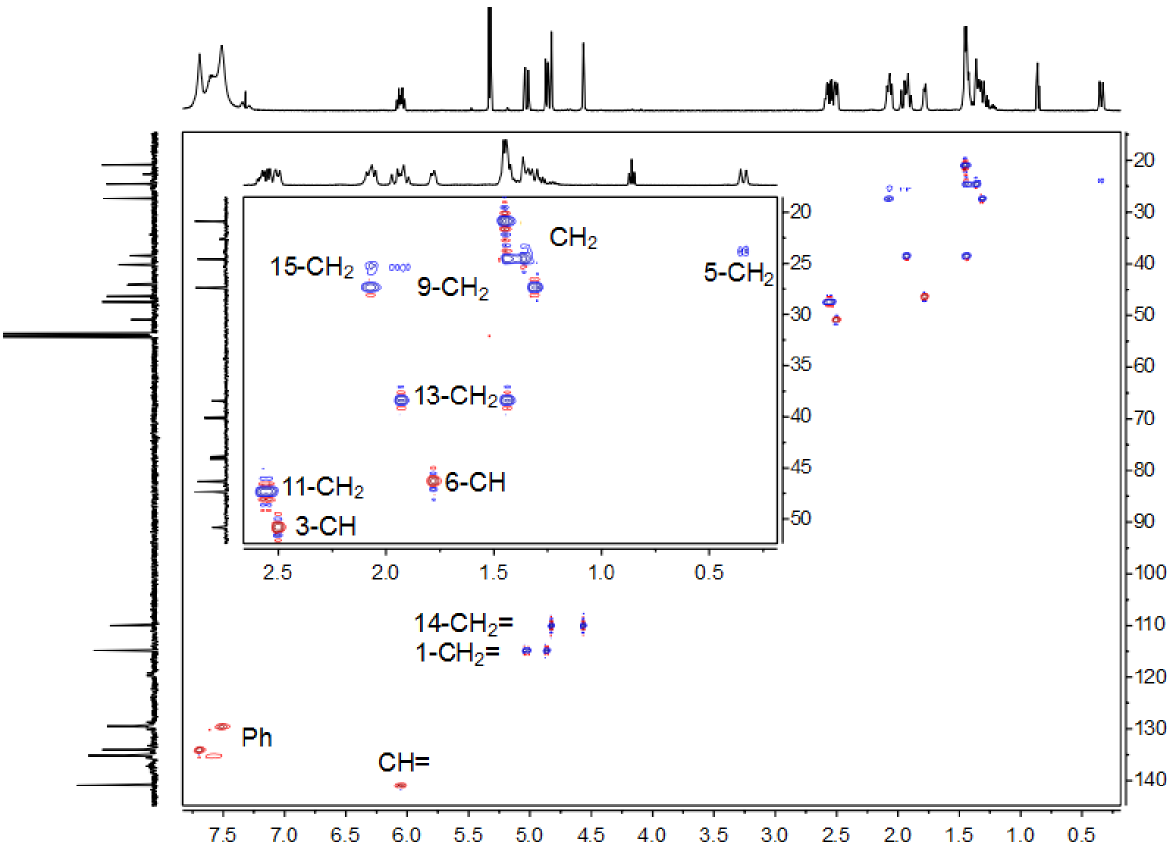

Figure S82. $^{1}H,^{13}C$ GHSQC (600/151 MHz, CD_2Cl_2, 273K) spectrum of compound **13a**.

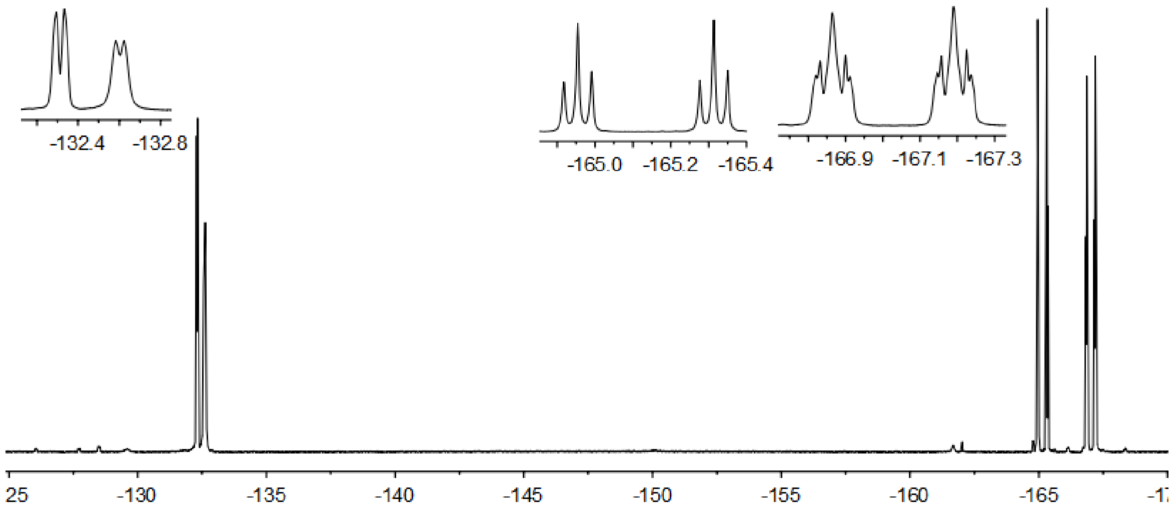

Figure S83. ^{19}F NMR (564 MHz, 273 K, CD_2Cl_2) spectrum of compound **13a**.

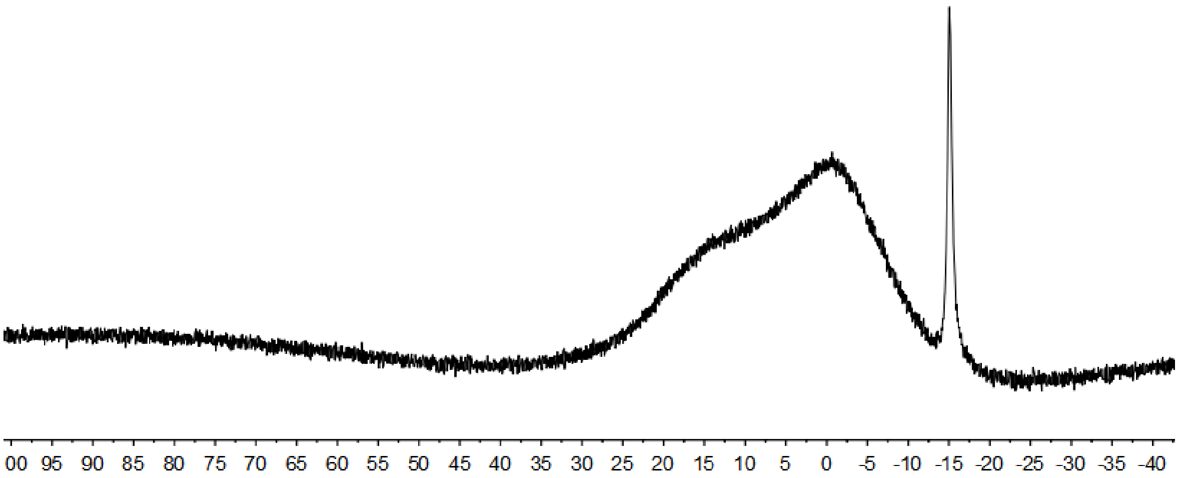

Figure S84. $^{11}B\{^1H\}$ NMR (192 MHz, 299 K, CD_2Cl_2) spectrum of compound **13a**.

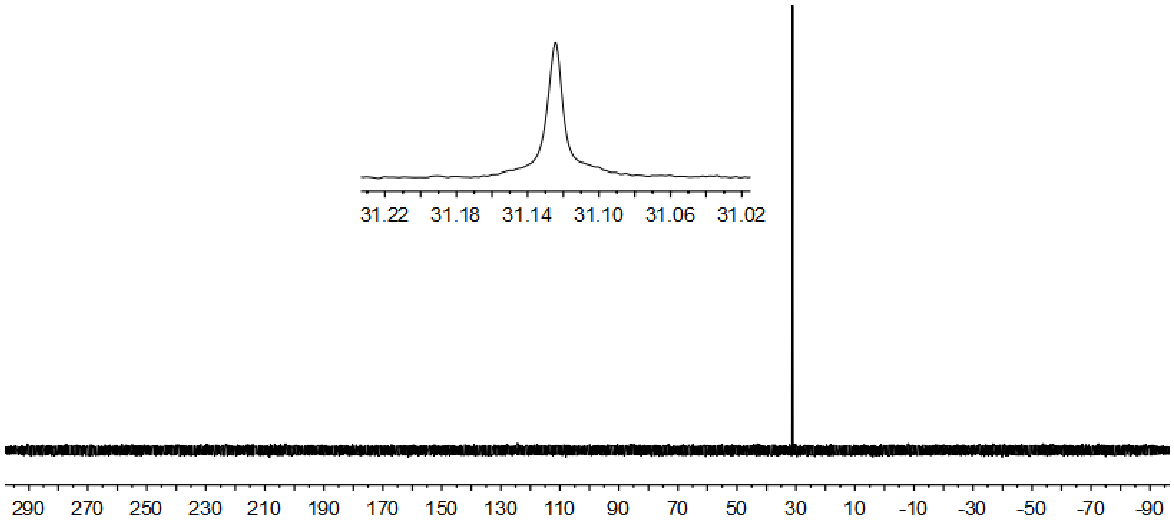

Figure S85. $^{31}P\{^1H\}$ NMR (243 MHz, 299 K, CD_2Cl_2) spectrum of compound **13a**.

Crystals suitable for the X-ray structure analysis were obtained from two-layer diffusion of pentane with a solution of the isolated white powder in dichlormethane solution at -35 °C.

X-ray crystal structure analysis of compound 13a (erk9509): A colorless plate-like specimen of $C_{45}H_{36}BF_{10}P \cdot CH_2Cl_2$, approximate dimensions 0.020 mm x 0.080 mm x 0.120 mm, was used for the X-ray crystallographic analysis. The X-ray intensity data were measured. A total of 1505 frames were collected. The total exposure time was 23.63 hours. The frames were integrated with the Bruker SAINT software package using a wide-frame algorithm. The integration of the data using a triclinic unit cell yielded a total of 24246 reflections to a maximum θ angle of 67.21° (0.84 Å resolution), of which 6850 were independent (average redundancy 3.540, completeness = 95.0%, R_{int} = 7.60%, R_{sig} = 8.01%) and 4461 (65.12%) were greater than 2σ(F^2). The final cell constants of a = 11.1982(12) Å, b = 11.5411(13) Å, c = 16.5533(16) Å, α = 96.789(6)°, β = 96.943(7)°, γ = 106.576(7)°, volume = 2009.0(4) $Å^3$, are based upon the refinement of the XYZ-centroids of 3179 reflections above 20 σ(I) with 8.098° < 2θ < 133.0°. Data were corrected for absorption effects using the multi-scan method (SADABS). The ratio of minimum to maximum apparent transmission was 0.829. The calculated minimum and maximum transmission coefficients (based on crystal size) are 0.7500 and 0.9510. The structure was solved and refined using the Bruker SHELXTL Software Package, using the space group *P*-1, with Z = 2 for the formula unit, $C_{45}H_{36}BF_{10}P \cdot CH_2Cl_2$. The final anisotropic full-matrix least-squares refinement on F^2 with 542 variables converged at R1 = 5.99%, for the observed data and wR2 = 16.85% for all data. The goodness-of-fit was 1.090. The largest peak in the final difference electron density synthesis was 0.374 $e^-/Å^3$ and the largest hole was -0.520 $e^-/Å^3$ with an RMS deviation of 0.079 $e^-/Å^3$. On the basis of the final model, the calculated density was 1.477 g/cm^3 and F(000), 916 e^-. CCDC number: 1922912.

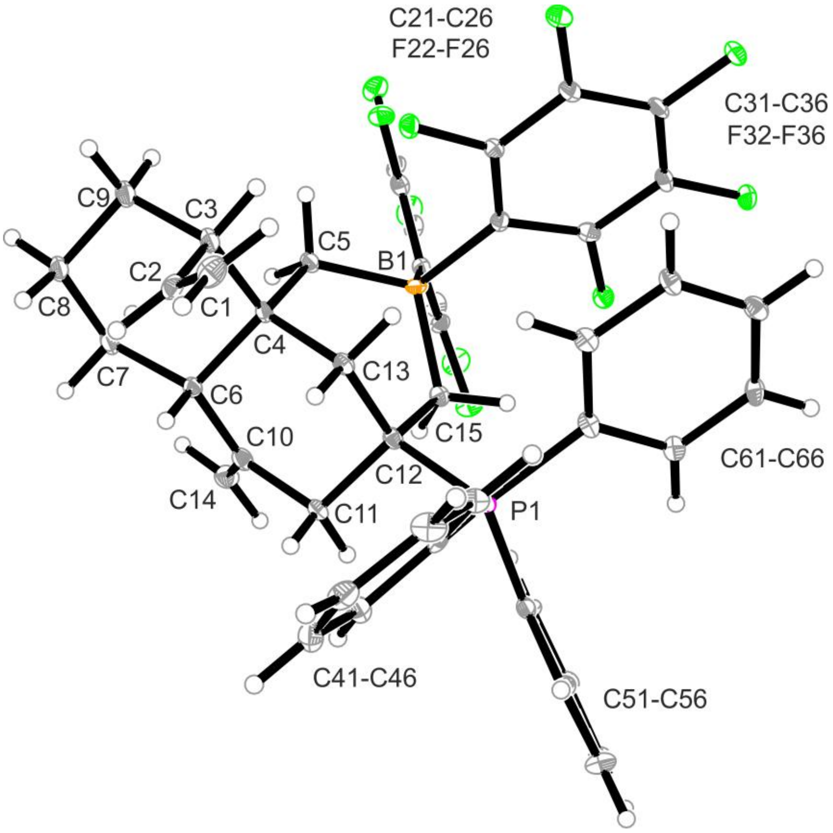

Figure S86. Crystal structure of compound **13a** (thermal ellipsoids: 15% probability).

Experiment 2: (Isolation and characterizations of compound **14a**)

Scheme S19.

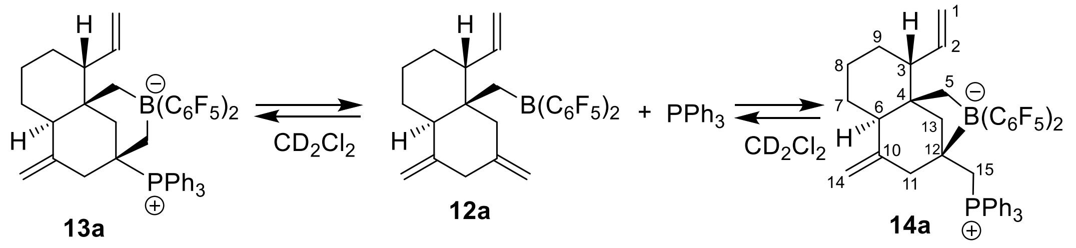

A solution of the isolated compound **13a** (40 mg, 0.05 mmol) in CH_2Cl_2 (0.5 mL) was layered with pentane (1.5 mL). While the resulting mixture was stored at room temperature for 24 h, colorless crystals precipitated. Part of the crystals were used for the X-ray crystal structure analysis, and the rest was washed with pentane (0.5 mL) and dried in vacuo giving compound **14a** (24 mg, 0.03 mmol, 60%) as a white crystalline material.

Anal. Calc. for $C_{45}H_{36}BF_{10}P{\cdot}CH_2Cl_2$: C, 61.84; H, 4.29. Found: C, 61.37; H, 4.23.

NMR data of compound **14a**:

^{1}H NMR (600 MHz, 299 K, CD_2Cl_2): δ ^{1}H: [7.74 (3H, p), 7.69 (6H, o), 7.62 (6H, m)](each m, Ph), 5.76 (dt, $^3J_{HH}$ = 16.9, 10.0 Hz, 1H, CH=), [4.71 (dd, $^3J_{HH}$ = 16.9 Hz, $^2J_{HH}$ = 2.4 Hz), 4.59 (dd, $^3J_{HH}$ = 10.0 Hz, $^2J_{HH}$ = 2.4 Hz)](each 1H, 1-CH_2=), [4.18 (dd, $^2J_{HH}$ = 15.2, $^2J_{PH}$ = 10.1 Hz), 2.70 (dd, $^2J_{HH}$ = 15.2, $^2J_{PH}$ = 12.8 Hz)](each 1H, PCH_2), 4.17/4.12 (each m, each 1H, 14-CH_2=), 2.85/1.89 (each dm, $^2J_{HH}$ = 12.6 Hz, each 1H, 11-CH_2), 2.17 (m, 1H, 3-CH), 1.73/1.33 (each m, each 1H, 9-CH_2), 1.57 (d, $^3J_{HH}$ = 11.6 Hz, 1H, 6-CH), 1.38/1.22 (each m, each 1H, 7-CH_2), 1.37 (m, 2H, 8-CH_2), 1.32/0.35 (each m, each 1H, BCH_2), 1.14/0.55 (each dm, $^2J_{HH}$ = 11.3 Hz, 13-CH_2).

^{13}C{^{1}H} NMR (151 MHz, 299 K, CD_2Cl_2): δ ^{13}C: 152.9 (C=), 142.0 (CH=), [134.7 (d, $^4J_{PC}$ = 3.1 Hz, p), 133.6 (d, $^2J_{PC}$ = 9.4 Hz, o), 130.4 (d, $^3J_{PC}$ = 12.0 Hz, m), 122.2 (d, $^1J_{PC}$ = 80.7 Hz, i)](Ph), 113.3 (1-CH_2=), 105.8 (14-CH_2=), 53.1 (br, 13-CH_2), 50.9 (3-CH), 48.5 (dd, J_{FC} = 10.5 Hz, $^3J_{PC}$ = 4.6 Hz, 11-CH_2), 46.4 (d, J_{FC} = 2.7 Hz, 4-C), 45.5 (6-CH), 38.0 (br m, 12-C), 33.9 (br dd, $^1J_{PC}$ = 32.7 Hz, J_{FC} = 6.8 Hz, PCH_2), 29.7 (9-CH_2), 29.3 (br, BCH_2), 25.5 (7-CH_2), 21.0 (8-CH_2), [C_6F_5 not listed].

^{13}C{^{1}H,^{19}F} NMR (151 MHz, 299 K, CD_2Cl_2): δ ^{13}C: 152.9 (C=), 142.0 (CH=), [134.7 (d, $^4J_{PC}$ = 3.1 Hz, p), 133.6 (d, $^3J_{PC}$ = 9.4 Hz, o), 130.4 (d, $^2J_{PC}$ = 12.0 Hz, m), 122.2 (d, $^1J_{PC}$ = 80.7 Hz, i)](Ph), [129.2, 129.5](each br, i-C_6F_5), 113.3 (1-CH_2=), 105.8 (14-CH_2=), 53.1 (br, 13-CH_2), 50.9 (3-CH), 48.5 (d, $^3J_{PC}$ = 4.6 Hz, 11-CH_2), 46.4 (s, 4-C), 45.5 (6-CH), 38.0 (br m, 12-C), 33.9 (d, $^1J_{PC}$ = 32 Hz, PCH_2), 29.7 (9-CH_2), 29.3 (br, BCH_2), 25.5 (7-CH_2), 21.0 (8-CH_2). [C_6F_5 not listed]

^{19}F NMR (564 MHz, 299 K, CD_2Cl_2): δ ^{19}F: [–126.1 (m, 1F, o), –127.6 (m, 1F, o’), –165.0 (t, $^3J_{FF}$ = 20.4 Hz, 1F, p), –166.9 (m, 1F, m’), –168.7 (m, 1F, m)](C_6F_5)[$\Delta\delta^{19}F_{m,p}$ = 1.9, 3.7], [–128.4 (m, 2F, o), –162.2 (t, $^3J_{FF}$ = 20.5 Hz, 1F, p), –166.2 (m, 2F, m)](C_6F_5)[$\Delta\delta^{19}F_{m,p}$ = 4.0].

$^{11}B\{^1H\}$ NMR (192 MHz, 299 K, CD_2Cl_2): δ ^{11}B: –6.8 ($\nu_{1/2}$ ~ 80 Hz).

$^{31}P\{^1H\}$ NMR (243 MHz, 299 K, CD_2Cl_2): δ ^{31}P: 19.9 ($\nu_{1/2}$ ~ 20 Hz).

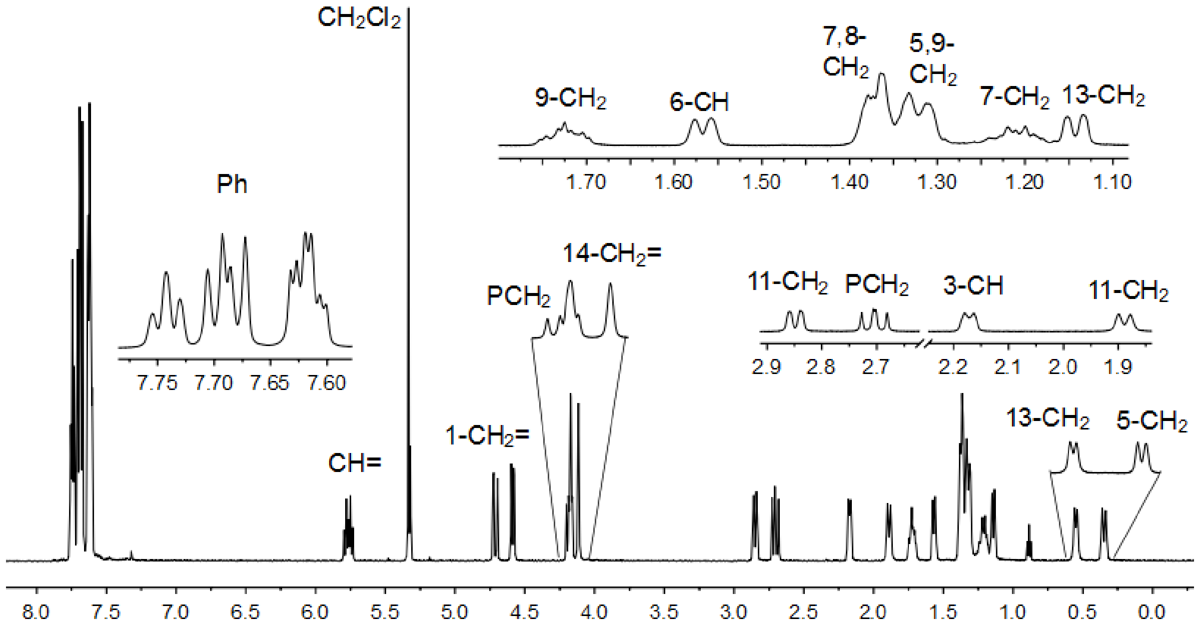

Figure S87. ^{1}H NMR (600 MHz, 299 K, CD_2Cl_2) spectrum of compound **14a**.

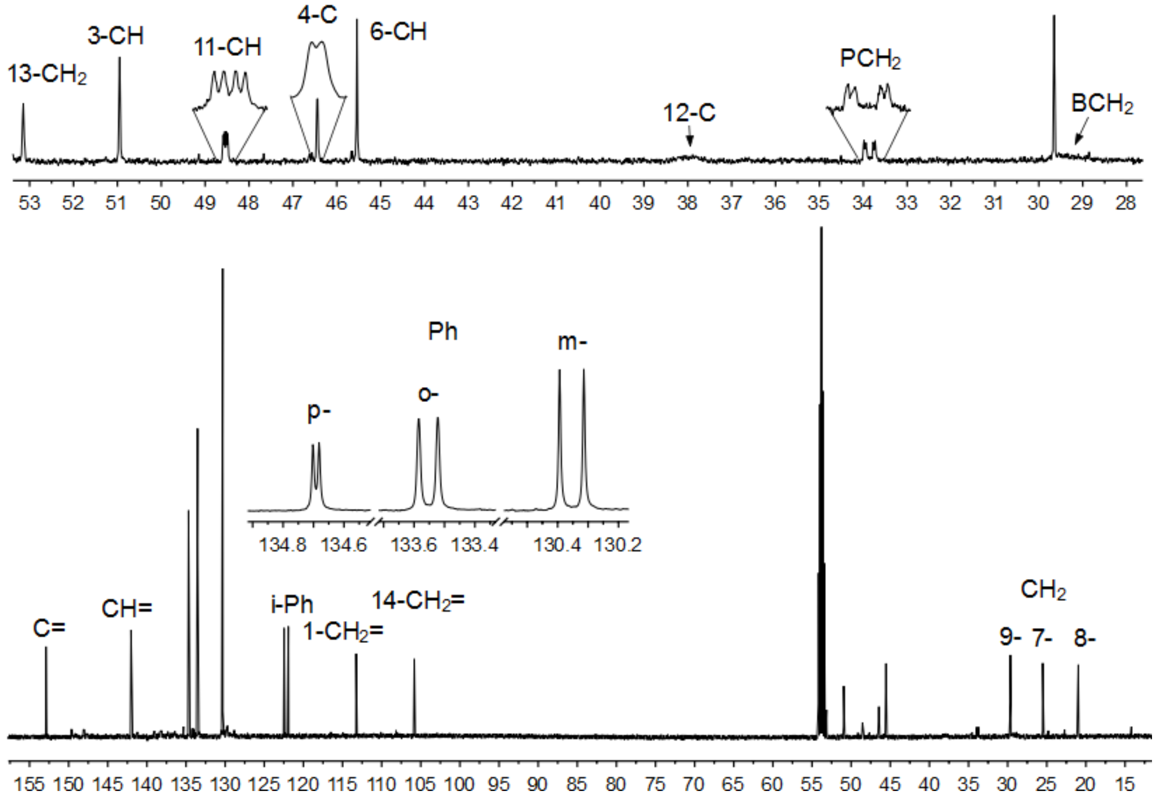

Figure S88. $^{13}C\{^1H\}$ NMR (151 MHz, 299 K, CD_2Cl_2) spectrum of compound **14a**.

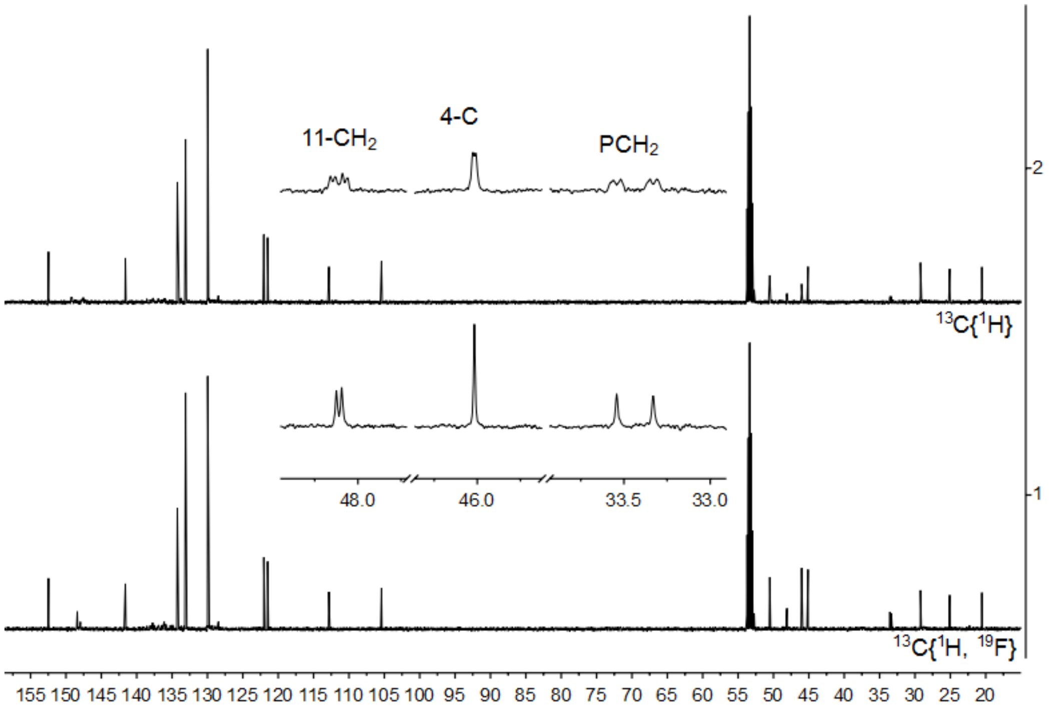

Figure S89. $^{13}C\{^1H,^{19}F\}$ NMR spectrum (1) and $^{13}C\{^1H\}$ NMR spectrum (2) (151 MHz, 299 K, CD_2Cl_2) of compound **14a**.

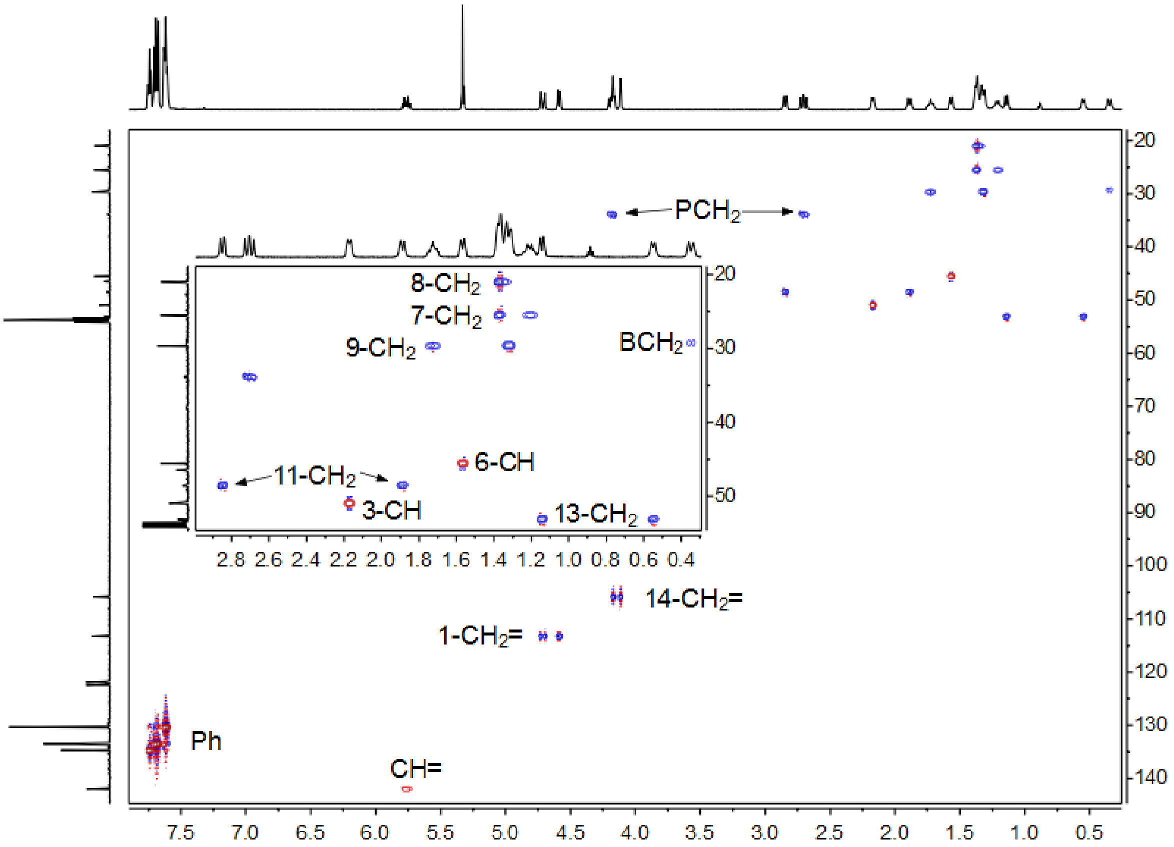

Figure S90. $^1H,^{13}C$ GHSQC (600/151 MHz, 299K, CD_2Cl_2) spectrum of compound **14a**.

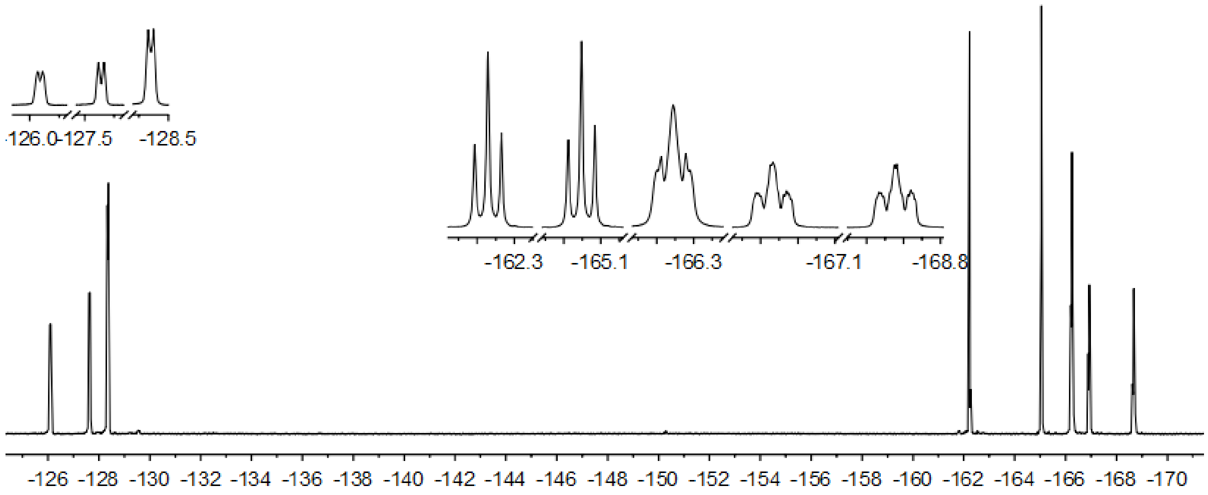

Figure S91. ^{19}F NMR (564 MHz, 299 K, CD_2Cl_2) spectrum of compound **14a**.

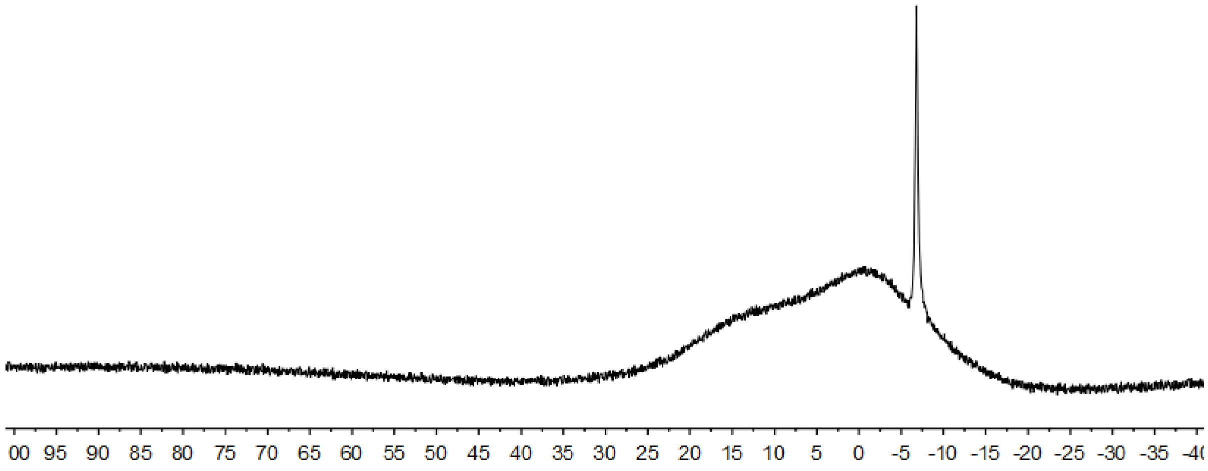

Figure S92. $^{11}B\{^1H\}$ NMR (192 MHz, 299 K, CD_2Cl_2) spectrum of compound **14a**.

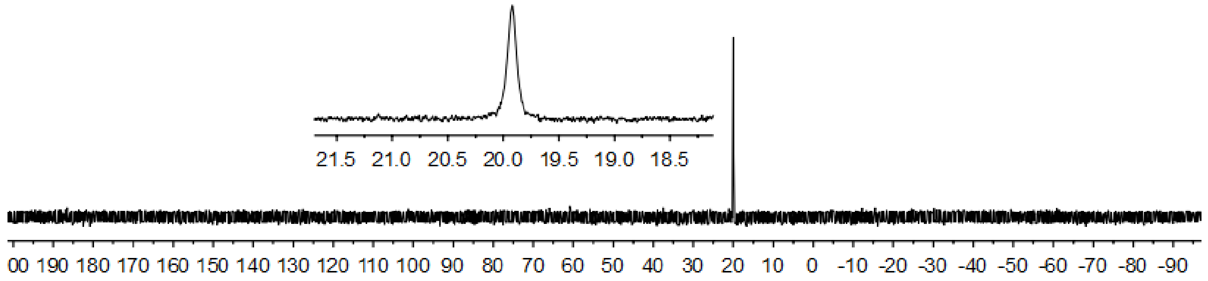

Figure S93. $^{31}P\{^1H\}$ NMR (243 MHz, 299 K, CD_2Cl_2) spectrum of compound **14a**.

X-ray crystal structure analysis of compound 14a (erk9502): A colorless prism-like specimen of $C_{45}H_{36}BF_{10}P \cdot CH_2Cl_2$, approximate dimensions 0.060 mm x 0.140 mm x 0.180 mm, was used for the X-ray crystallographic analysis. The X-ray intensity data were measured. A total of 1808 frames were collected. The total exposure time was 22.07 hours. The frames were integrated with the Bruker SAINT software package using a wide-frame algorithm. The integration of the data using a monoclinic unit cell yielded a total of 46629 reflections to a maximum θ angle of 66.67° (0.84 Å resolution), of which 7186 were independent (average redundancy 6.489, completeness = 99.7%, R_{int} = 7.22%, R_{sig} = 4.42%) and 5465 (76.05%) were greater than 2σ(F^2). The final cell constants of <u>a</u> = 11.6050(3) Å, <u>b</u> = 20.1373(4) Å, <u>c</u> = 17.8823(4) Å, β = 102.818(2)°, volume = 4074.83(16) $Å^3$, are based upon the refinement of the XYZ-centroids of 6466 reflections above 20 σ(I) with 6.705° < 2θ < 131.9°. Data were corrected for absorption effects using the multi-scan method (SADABS). The ratio of minimum to maximum apparent transmission was 0.824. The calculated minimum and maximum transmission coefficients (based on crystal size) are 0.6610 and 0.8640. The structure was solved and refined using the Bruker SHELXTL Software Package, using the space group $P2_1/c$, with Z = 4 for the formula unit, $C_{45}H_{36}BF_{10}P \cdot CH_2Cl_2$. The final anisotropic full-matrix least-squares refinement on F^2 with 588 variables converged at R1 = 5.10%, for the observed data and wR2 = 14.72% for all data. The goodness-of-fit was 1.026. The largest peak in the final difference electron density synthesis was 0.581 $e^-/Å^3$ and the largest hole was -0.395 $e^-/Å^3$ with an RMS deviation of 0.061 $e^-/Å^3$. On the basis of the final model, the calculated density was 1.456 g/cm^3 and F(000), 1832 e^-. CCDC number: 1922913.

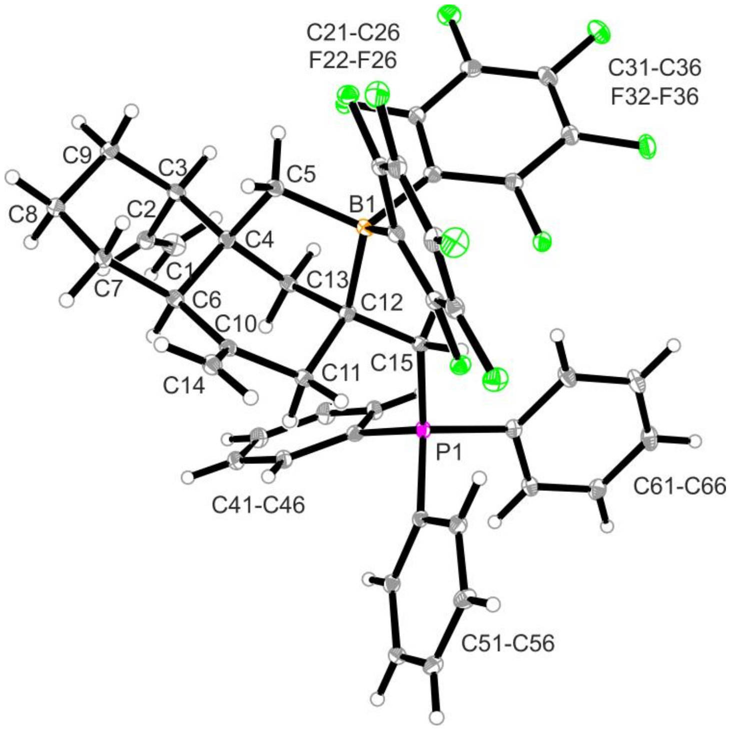

Figure S94. Crystal structure of compound **14a** (thermal ellipsoids: 15% probability).

Experiment 3a: (equilibration of the compounds **12a**, PPh_3, **13a**, and **14a**:
starting from compound **13a**)

Step 1: The isolated compounds **13a** was characterized by NMR experiments at 0 °C in CD_2Cl_2 (see above, see **Experiment 1**).

Step 2: Then the NMR tube was heated to 26 °C. After ca. 20 min. at room temperature a mixture of PPh_3 (ca. 13 mmol%, ^{1}H), compounds **12a** (ca. 13 mmol%, ^{1}H) and **13a** (ca. 74 mmol%, ^{1}H) was observed.

Step 3: Then the same sample was cooled down to 0 °C again. Only compound **13a** was observed.

Step 4: After storage of the sample for 7d at room temperature, it was characterized by NMR expriments at 26 °C: a mixture of PPh_3 (ca. 8 mmol%, ^{1}H), compounds **12a** (ca. 7 mmol%, ^{1}H), **13a** (ca. 20 mmol%, ^{1}H) and **14a** (ca. 65 mmol%, ^{1}H) was observed.

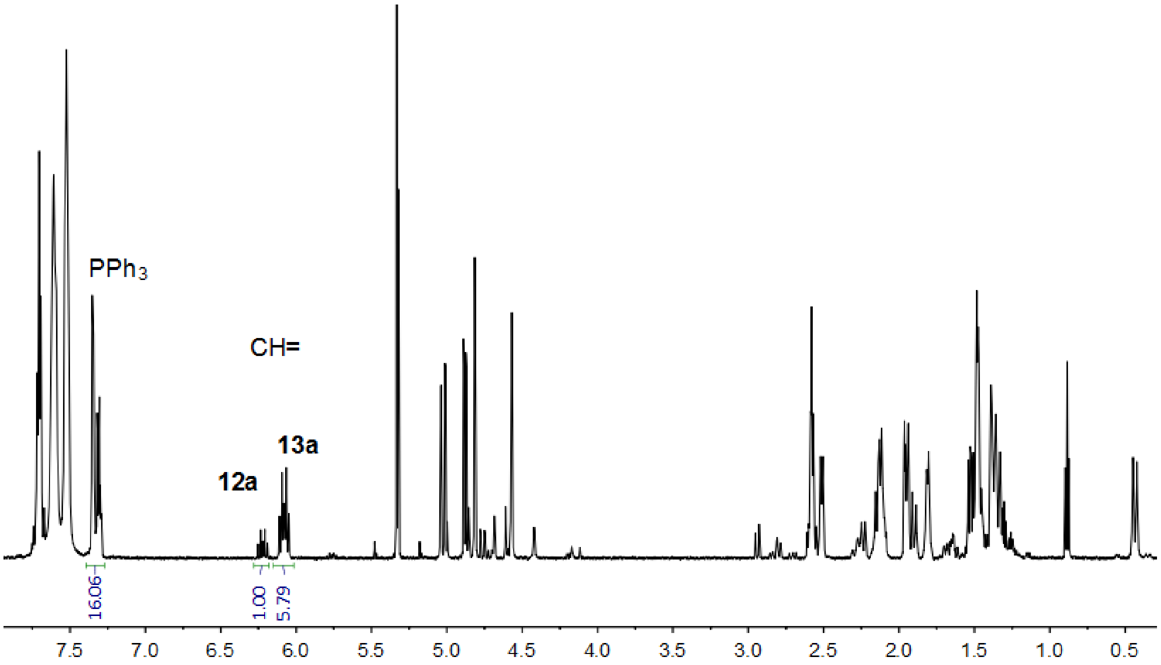

Figure S95. ^{1}H NMR (600 MHz, 299 K, CD_2Cl_2) spectrum of compound **13a** (see **Experiment 3a** *Step 2*).

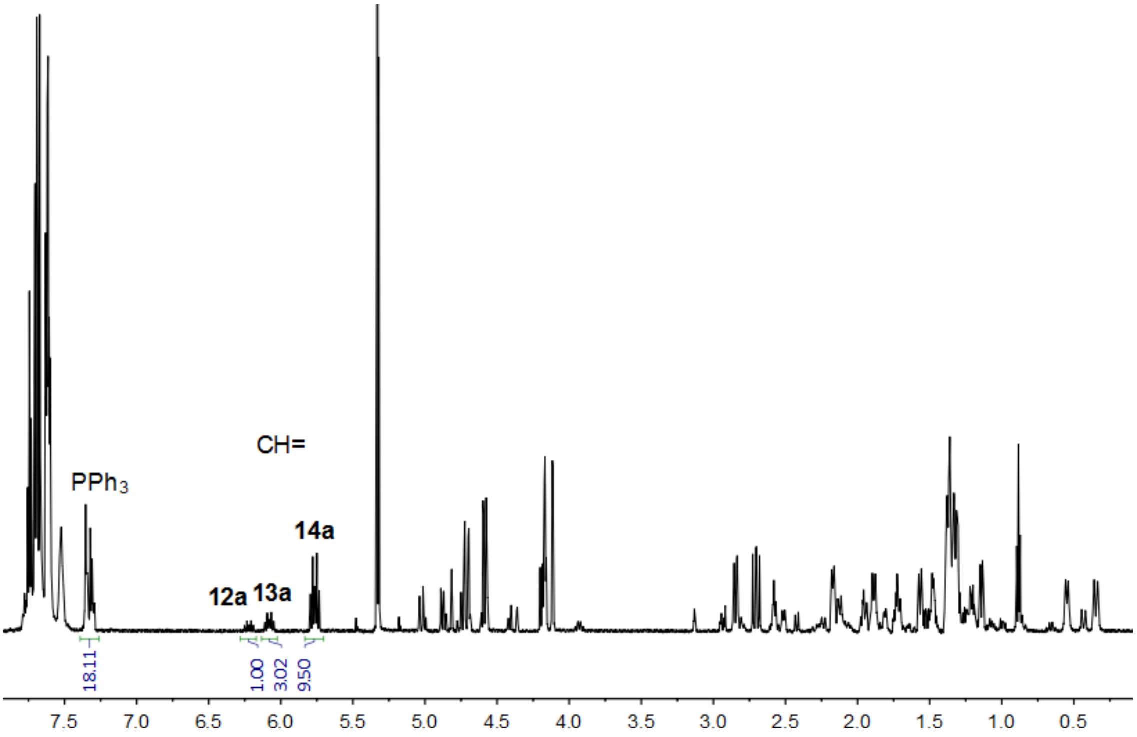

Figure S96. ^{1}H NMR (600 MHz, 299 K, CD_2Cl_2) spectrum of compound **13a** after storage for 7 days at room temperature (see **Experiment 3a** *Step 4*).

Experiment 3b: (equilibration of the compounds **12a**, PPh_3, **13a**, and **14a**: starting from compound **14a**)

Step 1: The isolated compounds **14a** was characterized by NMR experiments at 26 °C (see above: see **Experiment 2**).

Step 2: After storage of the sample for 7d at room temperature, it was characterized by NMR expriments at 26 °C: a mixture of PPh_3 (ca. 6 mmol%, ^{1}H), compounds **12a** (ca. 6 mmol%, ^{1}H), **13a** (ca. 20 mmol%, ^{1}H) and **14a** (ca. 68 mmol%, ^{1}H)].

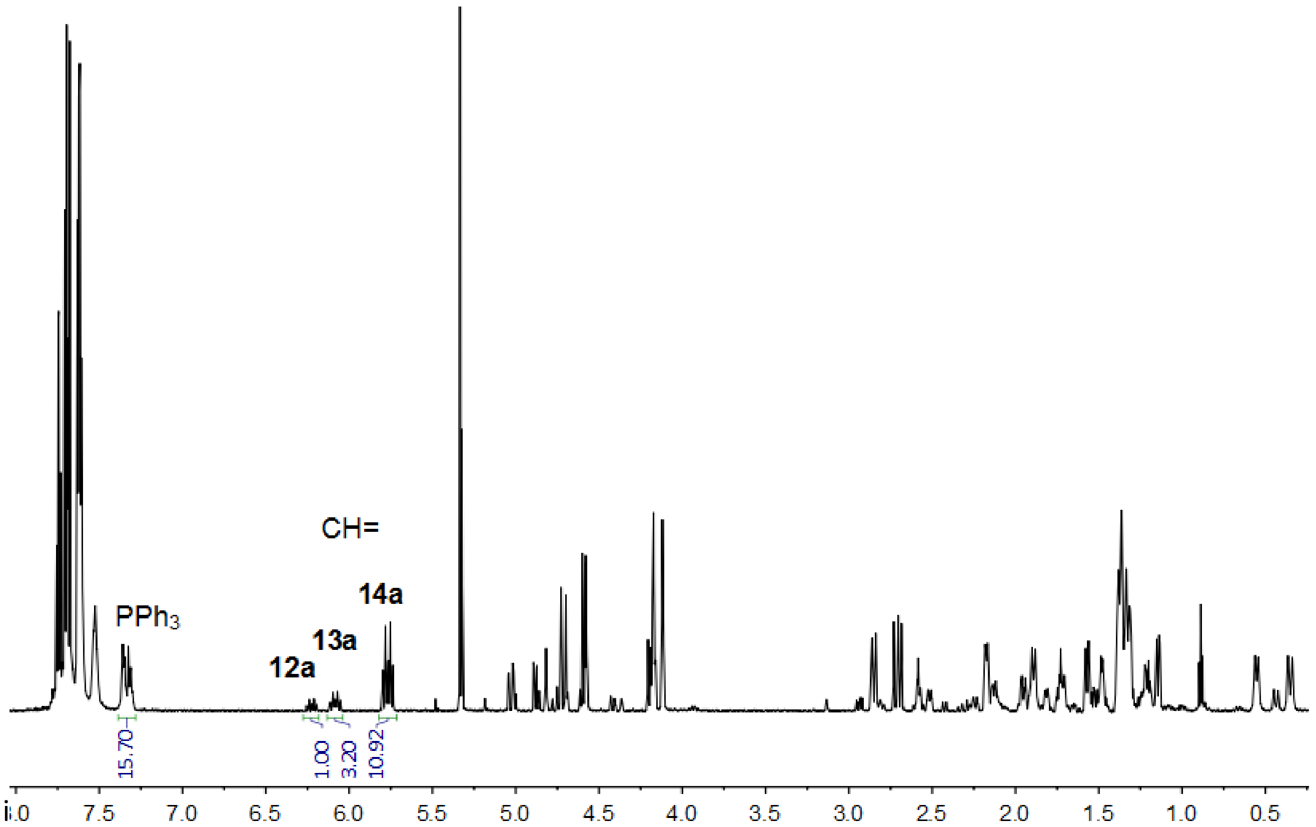

Figure S97. ^{1}H NMR (600 MHz, 299 K, CD_2Cl_2) spectrum of the isolated compound **14a** after storage for 7 days at room temperature (see **Experiment 3b** *Step 2*).

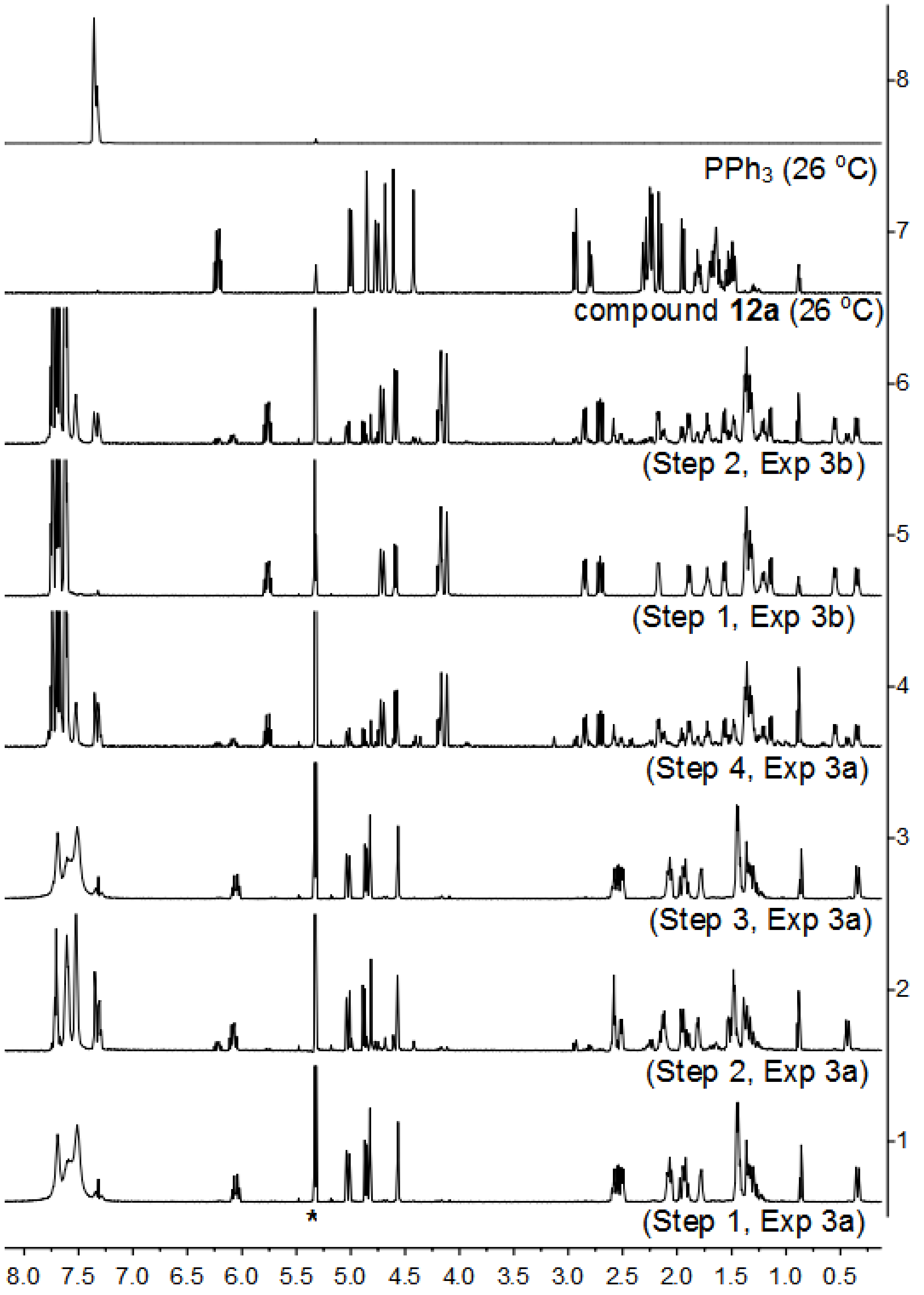

Figure S98. ^{1}H NMR (600 MHz, CD_2Cl_2) spectra of (1) (273 K) the isolated compound **13a** as decribed in Step 1, Experiment 3a, (2) (299 K) the reaction mixture as decribed in Step 2, Experiment 3a, (3) (273 K) the reaction mixture as decribed in Step 3, Experiment 3a, (4) (299 K) the reaction mixture as decribed in Step 4, Experiment 3a, (5) (299 K) the isolated compound **14a** as decribed in Step 1, Experiment 3b, (6) (299 K) the reaction mixture as decribed in Step 2, Experiment 3b, (7) (299 K) the isolated compound **12a** and (8) (299 K) PPh_3 for comparison.

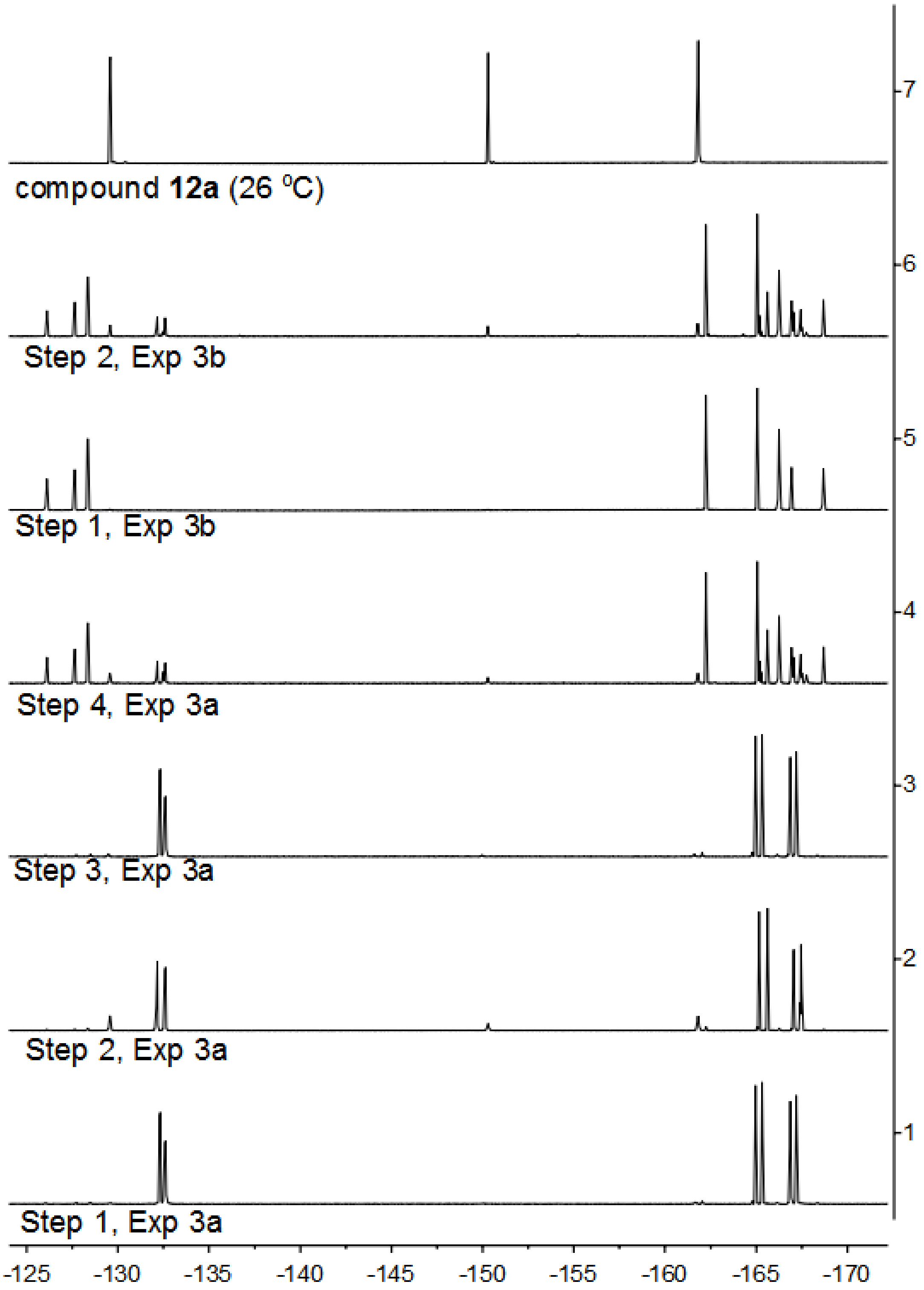

Figure S99. ^{19}F NMR (564 MHz, CD_2Cl_2) spectra of (1) (273 K) the isolated compound **13a** as decribed in Step 1, Experiment 3a, (2) (299 K) the reaction mixture as decribed in Step 2, Experiment 3a, (3) (273 K) the reaction mixture as decribed in Step 3, Experiment 3a, (4) (299 K) the reaction mixture as decribed in Step 4, Experiment 3a, (5) (299 K) the isolated compound **14a** as decribed in Step 1, Experiment 3b, (6) (299 K) the reaction mixture as decribed in Step 2, Experiment 3b, (7) (299 K) the isolated compound **12a** for comparison.

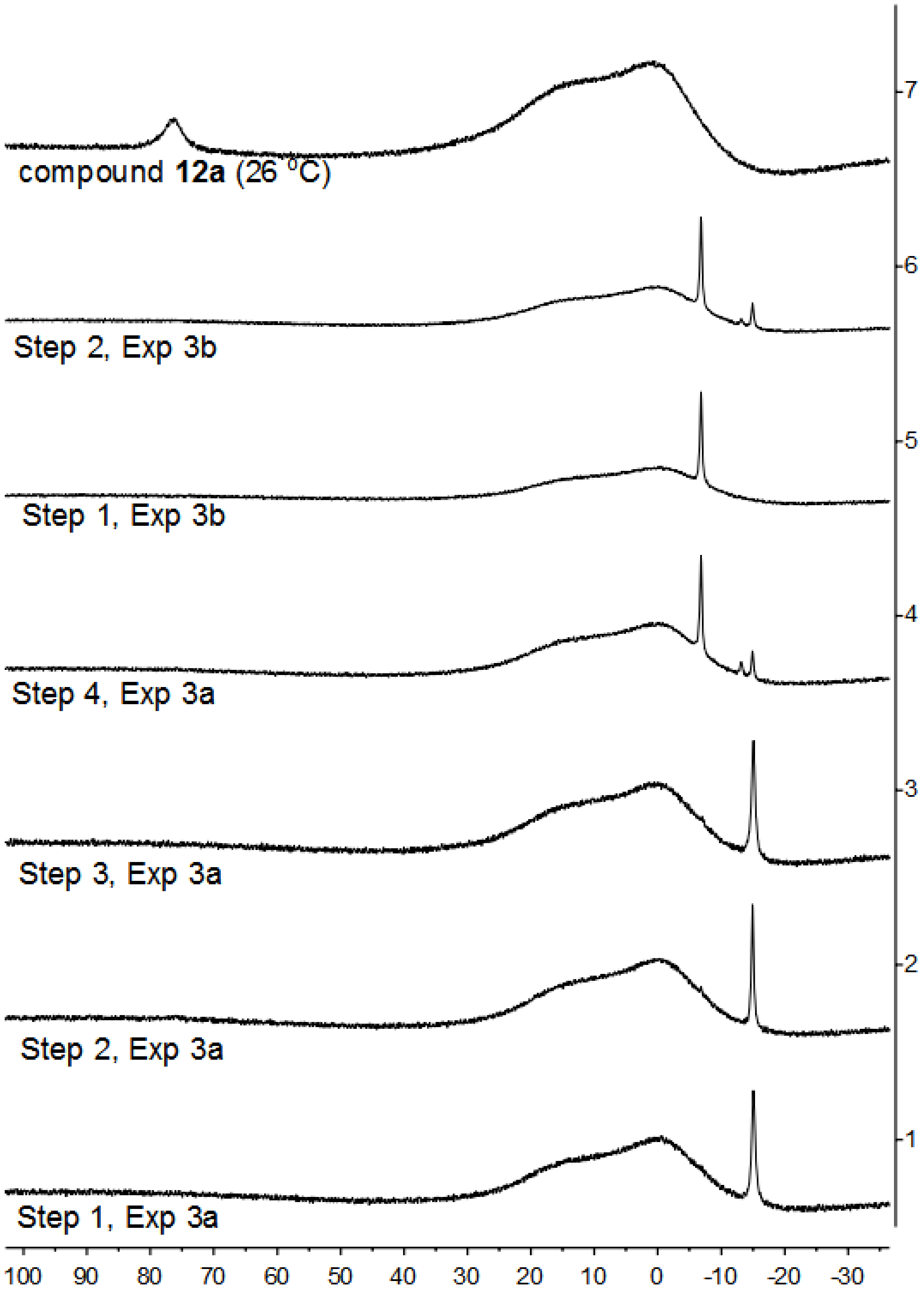

Figure S100. $^{11}B\{^1H\}$ NMR (192 MHz, CD_2Cl_2) spectra of (273 K) the isolated compound **13a** as decribed in Step 1, Experiment 3a, (2) (299 K) the reaction mixture as decribed in Step 2, Experiment 3a, (3) (273 K) the reaction mixture as decribed in Step 3, Experiment 3a, (4) (299 K) the reaction mixture as decribed in Step 4, Experiment 3a, (5) (299 K) the isolated compound **14a** as decribed in Step 1, Experiment 3b, (6) (299 K) the reaction mixture as decribed in Step 2, Experiment 3b, (7) (299 K) the isolated compound **12a** for comparison.

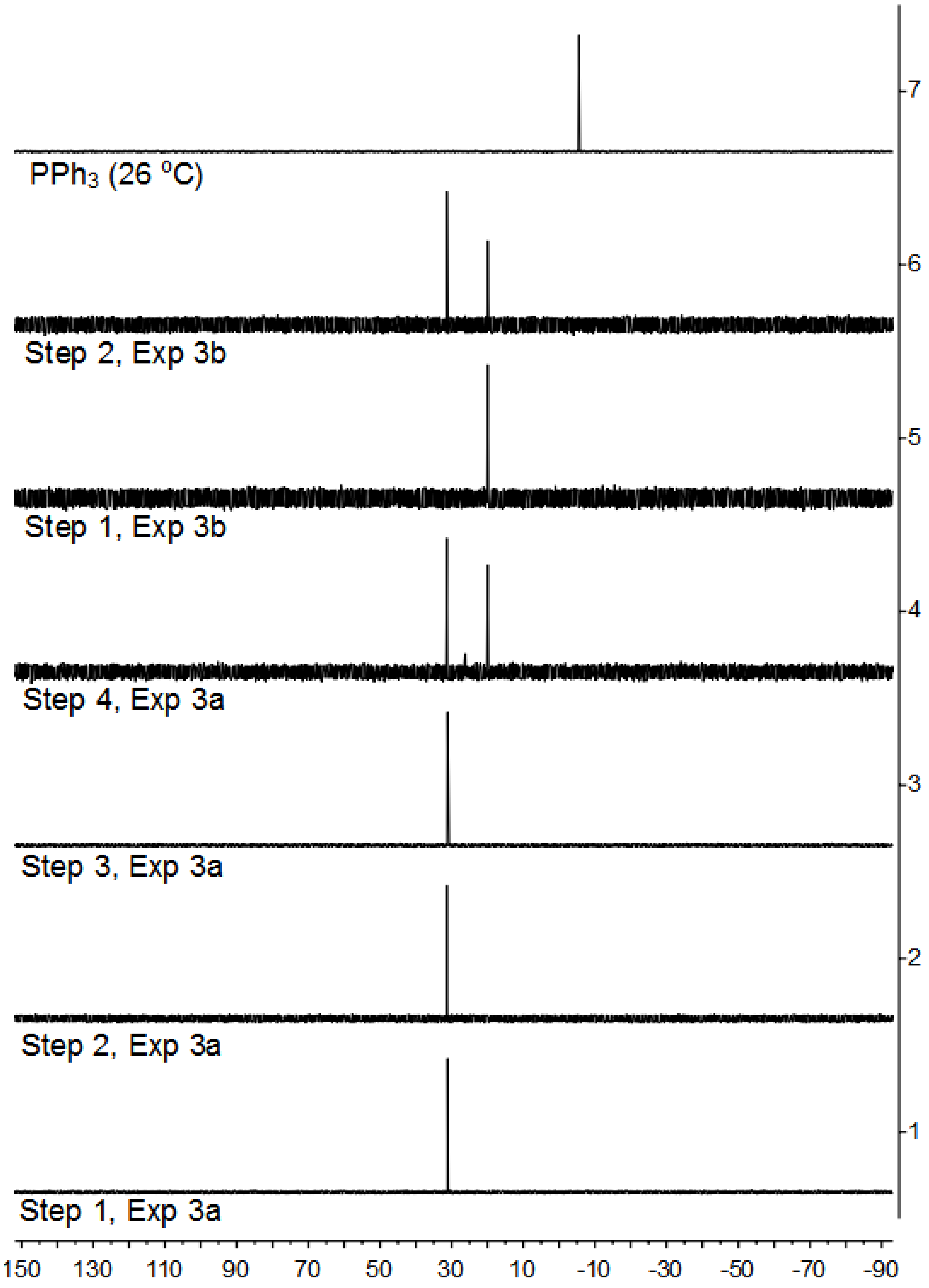

Figure S101. $^{31}P\{^1H\}$ NMR (243 MHz, CD_2Cl_2) spectra of (1) (273 K) the isolated compound **13a** as decribed in Step 1, Experiment 3a, (2) (299 K) the reaction mixture as decribed in Step 2, Experiment 3a, (3) (273 K) the reaction mixture as decribed in Step 3, Experiment 3a, (4) (299 K) the reaction mixture as decribed in Step 4, Experiment 3a, (5) (299 K) the isolated compound **14a** as decribed in Step 1, Experiment 3b, (6) (299 K) the reaction mixture as decribed in Step 2, Experiment 3b, (7) (299 K) the isolated compound **12a** and (8) (299 K) PPh_3 for comparison.

P) Synthesis of compound **15a**

Scheme S20.

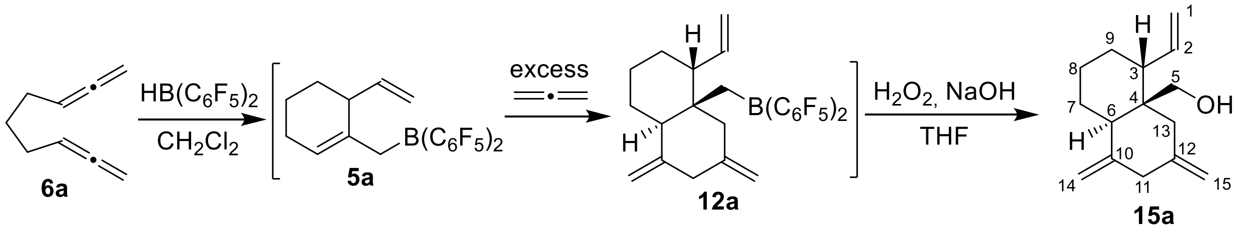

A suspension of $HB(C_6F_5)_2$ (208 mg, 0.60 mmol) in CH_2Cl_2 (2 mL) was added to a solution of bisallene **6a** (80.0 mg, 0.66 mmol) in CH_2Cl_2 (2 mL) at room temperature in a Schlenk tube. After evacuating the Schlenk tube carefully, the reaction mixture was exposed to allene gas at room temperature. The resulting reaction mixture was stirred at room temperature for ca. 24 h. Then all the volatile were removed in vacuo. Then the obtained oily residue was dissolved in THF (2 mL) and subsequently aqueous NaOH (3 M, 0.2 mL) and H_2O_2 (35%, 0.2 mL) were added. The resulting mixture was stirred for 1 h at room temperature. Afterwards, K_2CO_3 was added to saturate the reaction mixture and then ether (2 mL) was added. The organic phase was separated and the aqueous layer was extracted with ether (5 mL × 3). The organic phases were combined, washed with brine and dried with $MgSO_4$. All the volatile were removed by rotary evaporator and the residue was purified via column chromatography (silica gel, eluent: ethyl acetate / pentane (v:v) = 1 / 20) giving compound **15a** (55 mg, 0.25 mmol, 42%) as a white solid.

HRMS (ESI) m/z: calc. for $C_{15}H_{22}O$ $[M+Na]^+$: 241.1563. Found: 241.1564.

NMR data of compound **15a**:

^{1}H NMR (600 MHz, 299 K, CD_2Cl_2): δ ^{1}H: 6.24 (dt, $^3J_{HH}$ = 16.9, 10.3 Hz, 1H, CH=), 5.09 (m, 2H, 1-CH$_2$=), 4.75/4.52 (each m, each 1H, 14-CH$_2$=), 4.71/4.62 (each m, each 1H, 15-CH$_2$=), 3.63/3.35 (each d, $^2J_{HH}$ = 11.4 Hz, each 1H, OCH$_2$), [2.95 (dd, $^2J_{HH}$ = 13.9 Hz, J = 2.1 Hz), 2.82 (dm, $^2J_{HH}$ = 13.9 Hz)](each 1H, 11-CH$_2$), 2.52 (ddd, $^3J_{HH}$ = 10.3, 4.1, 2.4 Hz, 1H, 3-CH), 2.30 (dm, $^3J_{HH}$ = 12.6 Hz, 1H, 6-CH), [2.17 (dd, $^2J_{HH}$ = 13.7 Hz, J = 2.0 Hz), 2.02 (dm, $^2J_{HH}$ = 13.7 Hz)](each 1H, 13-CH$_2$), 1.89/1.46 (each m, each 1H, 9-CH$_2$), 1.64/1.44 (each m, each 1H, 7-CH$_2$), 1.64 (m, 2H, 8-CH$_2$), 1.10 (br s, 1H, OH).

^{13}C{^{1}H} NMR (151 MHz, 299 K, CD_2Cl_2): δ ^{13}C: 149.5 (10-C=), 147.0 (12-C=), 139.4 (CH=), 116.2 (1-CH$_2$=), 108.7 (15-CH$_2$=), 107.0 (14-CH$_2$=), 60.2 (OCH$_2$), 45.8 (11-CH$_2$), 43.1 (6-CH), 42.6 (3-CH), 42.1 (C), 41.7 (13-CH$_2$), 28.2 (9-CH$_2$), 24.3 (7-CH$_2$), 21.2 (8-CH$_2$).

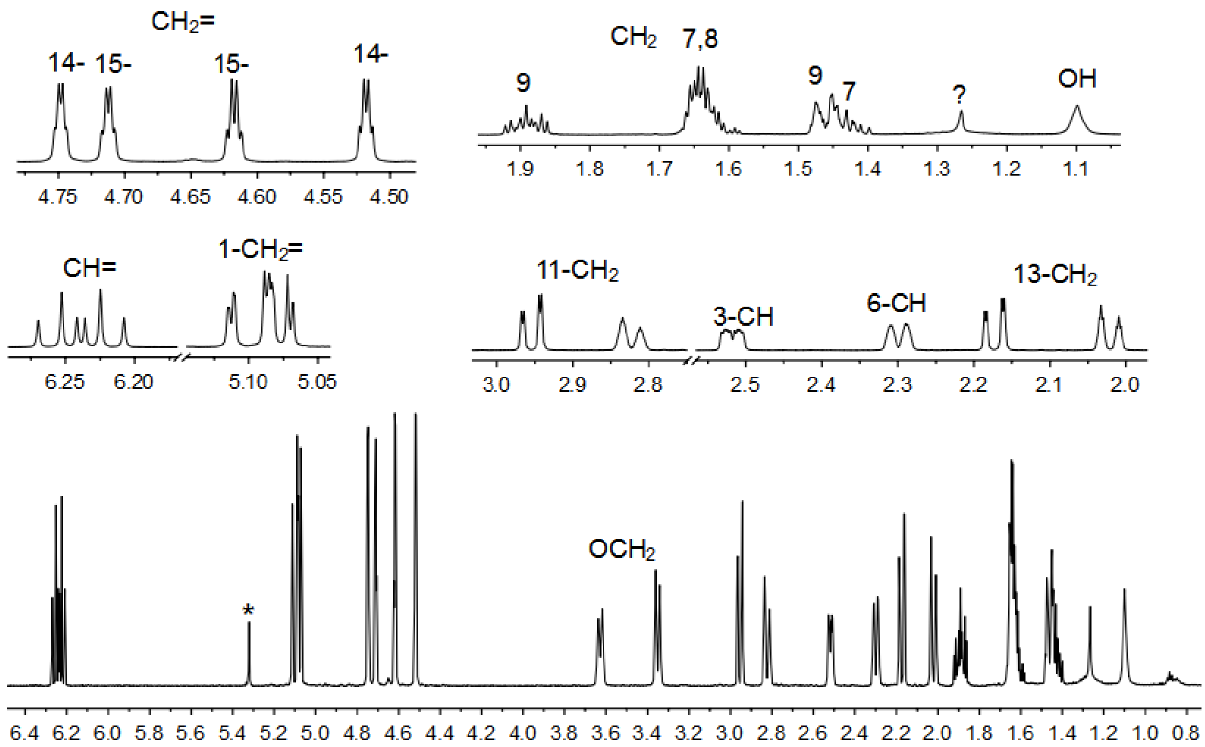

Figure S102. ^{1}H NMR (600 MHz, 299 K, CD_2Cl_2*) spectrum of the isolated compound **15a**.

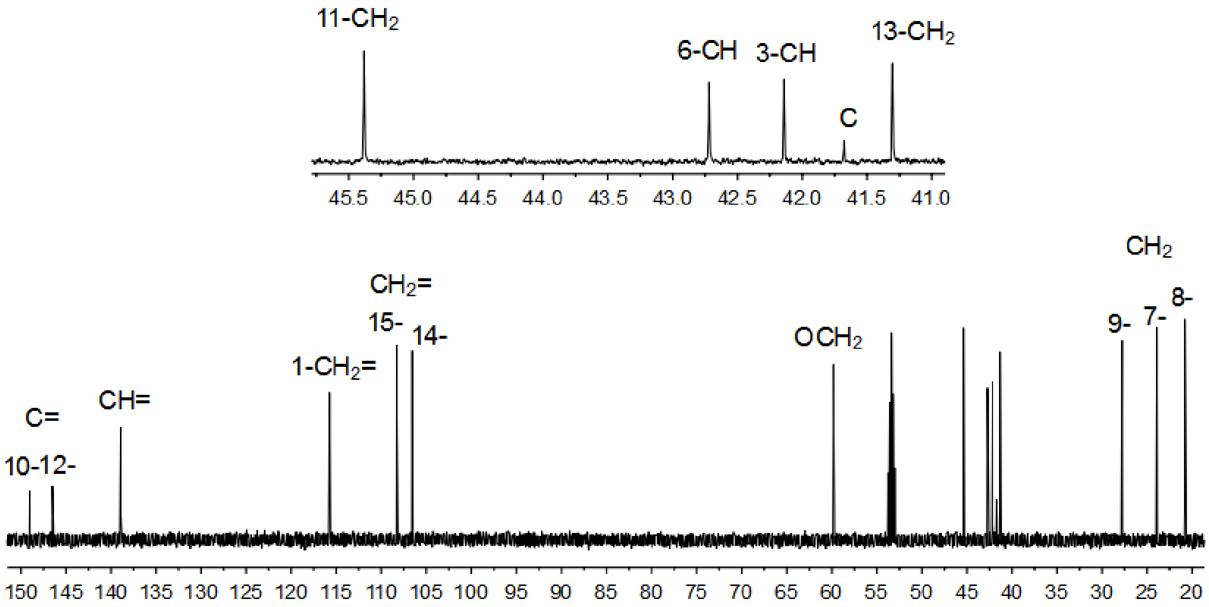

Figure S103. $^{13}C\{^{1}H\}$ NMR (151 MHz, 299 K, CD_2Cl_2) spectrum of the isolated compound **15a**.

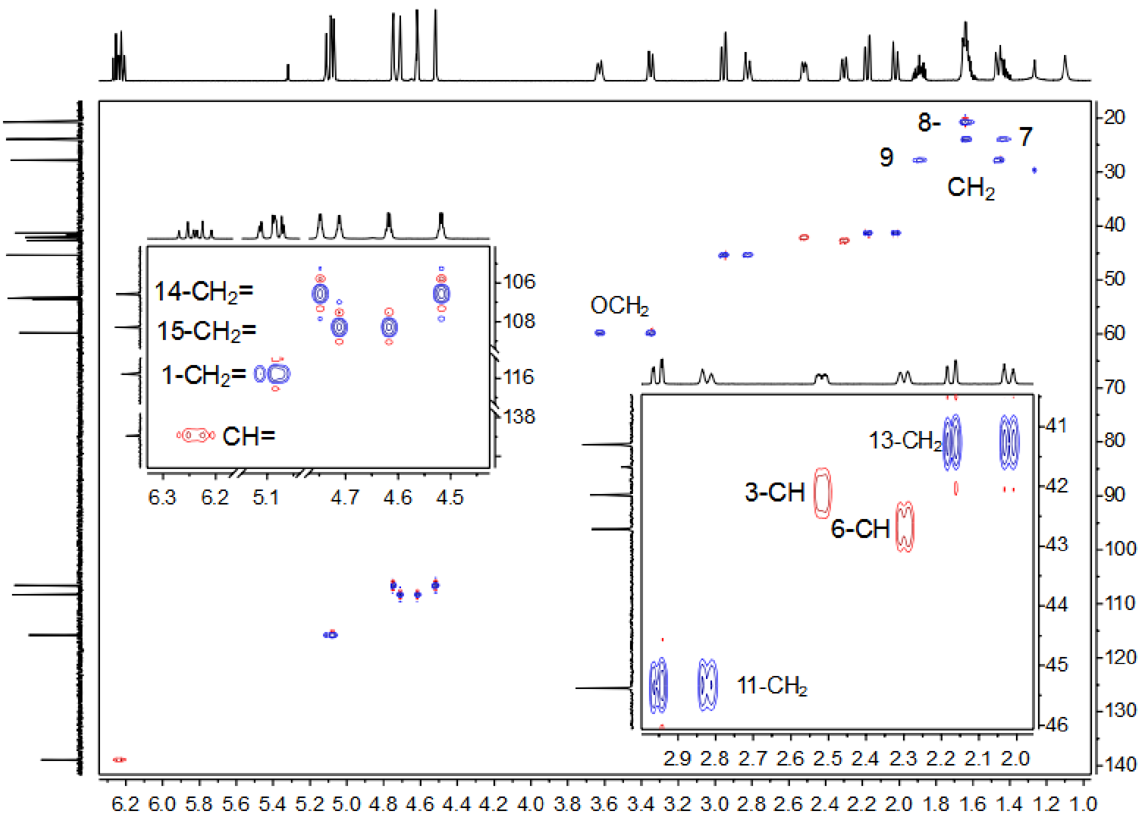

Figure S104. $^{1}H,^{13}C$ GHSQC (600/151 MHz, CD_2Cl_2, 299K) spectrum of the isolated compound **15a**.

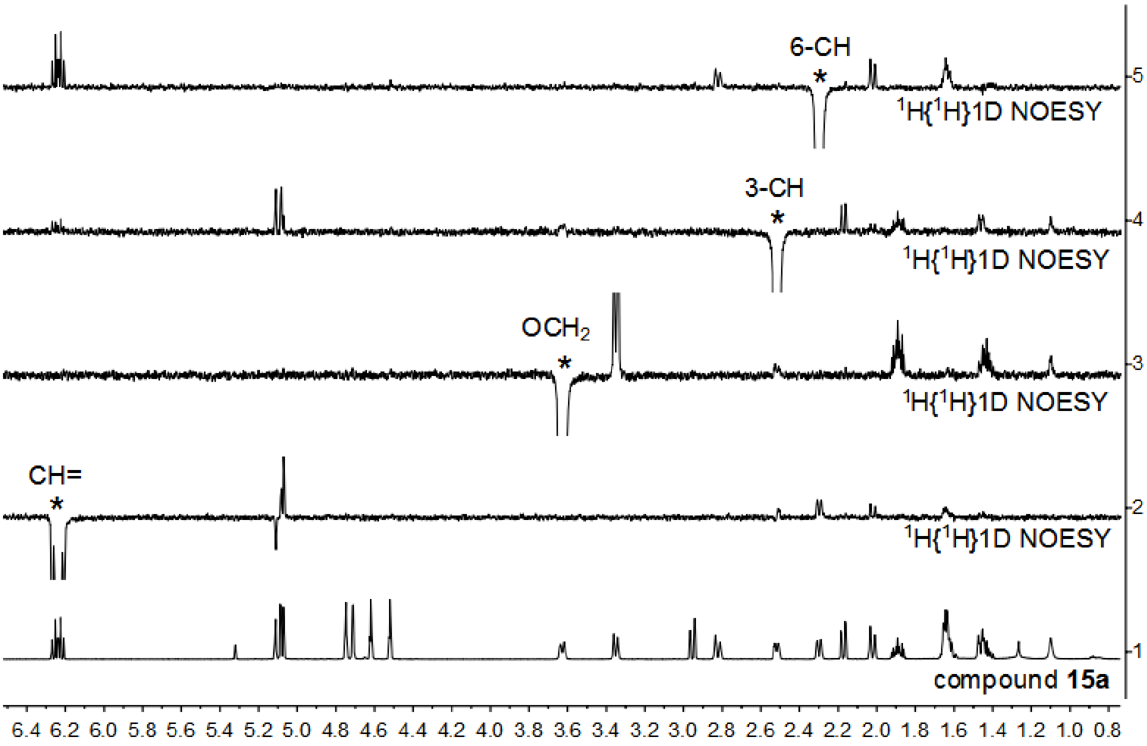

Figure S105. (1) ^{1}H NMR and (2 to 5) $^{1}H\{^{1}H\}$ 1D NOESY (600 MHz, 299 K, CD_2Cl_2) spectra of the isolated compound **15a**. Irradiation points (*): (2) $\delta^{1}H$ 6.24 (CH=); (3) $\delta^{1}H$ 3.63 (OCH_2); (4) $\delta^{1}H$ 2.52 (3-CH); (5) $\delta^{1}H$ 2.30 (6-CH).

Q) Attempted cyclization of bisallene **16**

Scheme S21.

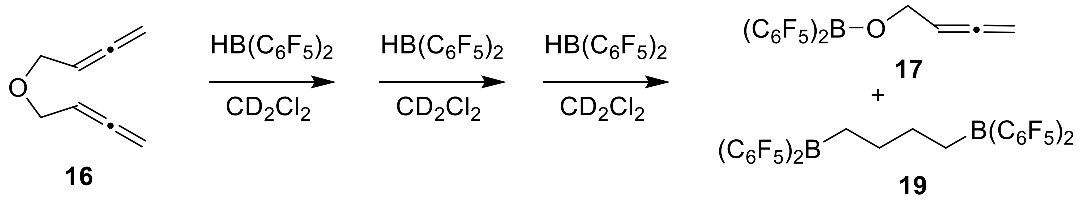

A suspension of $HB(C_6F_5)_2$ (34.6 mg, 0.10 mmol) in CD_2Cl_2 (0.3 mL) was added to a solution of bisallene **16** (12.2 mg, 0.10 mmol) in CD_2Cl_2 (0.3 mL) at room temperature. The resulting reaction mixture was transferred into NMR Young tube and characterized by NMR measurements. Subsequently an additional equivalent of $HB(C_6F_5)_2$ (34.6 mg, 0.10 mmol) in CD_2Cl_2 (0.2 mL) was added, mixture was vigorously shaken and characterized by NMR experiments. After addition of a third equivalent of $HB(C_6F_5)_2$ (34.6 mg, 0.10 mmol) in CD_2Cl_2 (0.2 mL) NMR measurement revealed complete consumption of starting material **16**.

NMR data of compound **17**:

^{1}H NMR (600 MHz, 299 K, CD_2Cl_2): δ ^{1}H: 5.38 (quint, $^3J_{HH}$ = $^4J_{HH}$ = 6.6 Hz, 1H, =CH), 4.86 (dt, $^3J_{HH}$ = 6.6 Hz, $^5J_{HH}$ = 2.6 Hz, 2H, OCH_2), 4.72 (dt, $^3J_{HH}$ = 6.6 Hz, $^5J_{HH}$ = 2.6 Hz, 2H, $=CH_2$).

^{19}F NMR (564 MHz, 299 K, CD_2Cl_2): δ ^{19}F: –132.0 (m, 2F, *o*-C_6F_5), –150.0 (br, 1F, *p*-C_6F_5), –161.8 (br,2F, *m*-C_6F_5).

^{11}B{^{1}H} NMR (192 MHz, 299 K, CD_2Cl_2): δ ^{11}B: 40.6 ($\nu_{1/2}$ ~ 300 Hz).

NMR data of compound **19**:

^{1}H NMR (600 MHz, 299 K, CD_2Cl_2): δ ^{1}H: 2.09 (br, 1H, CH_2B), 1.64 (m, 1H, C*H*$_2$$CH_2B$).

^{19}F NMR (564 MHz, 299 K, CD_2Cl_2): δ ^{19}F: –130.5 (m, 2F), –148.5 (tt, $^3J_{FF}$ = 19.9 Hz, $^4J_{FF}$ = 4.6 Hz, 1F, *p*-C_6F_5), –161.8 (m, 2F, *m*-C_6F_5).

^{11}B{^{1}H} NMR (192 MHz, 299 K, CD_2Cl_2): δ ^{11}B: 73.5 ($\nu_{1/2}$ ~ 700Hz).

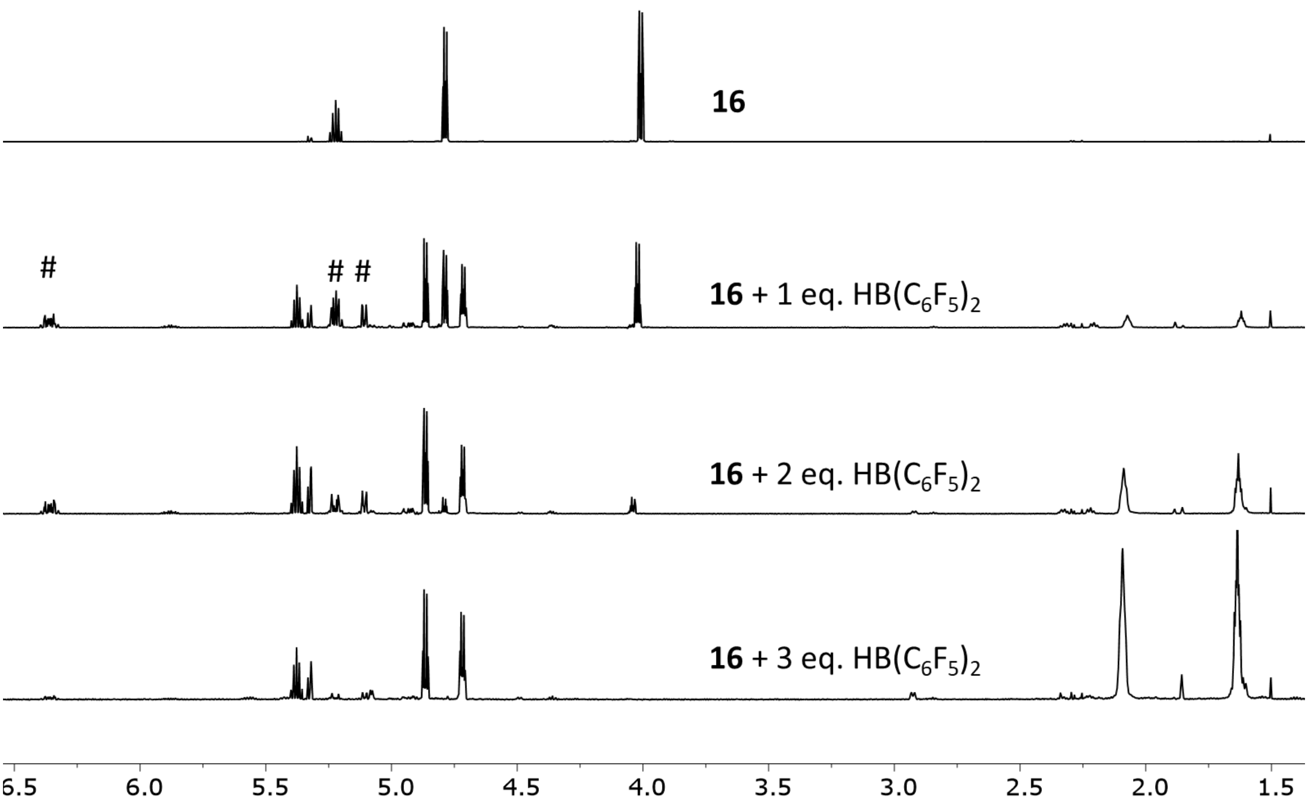

Figure S106. ^{1}H NMR (600 MHz, CD_2Cl_2, 299K) spectra monitoring the reaction of compound **16** after addition of one, a second and a third equivalent of $HB(C_6F_5)_2$. [#: 1,3-butadiene (**18**)].

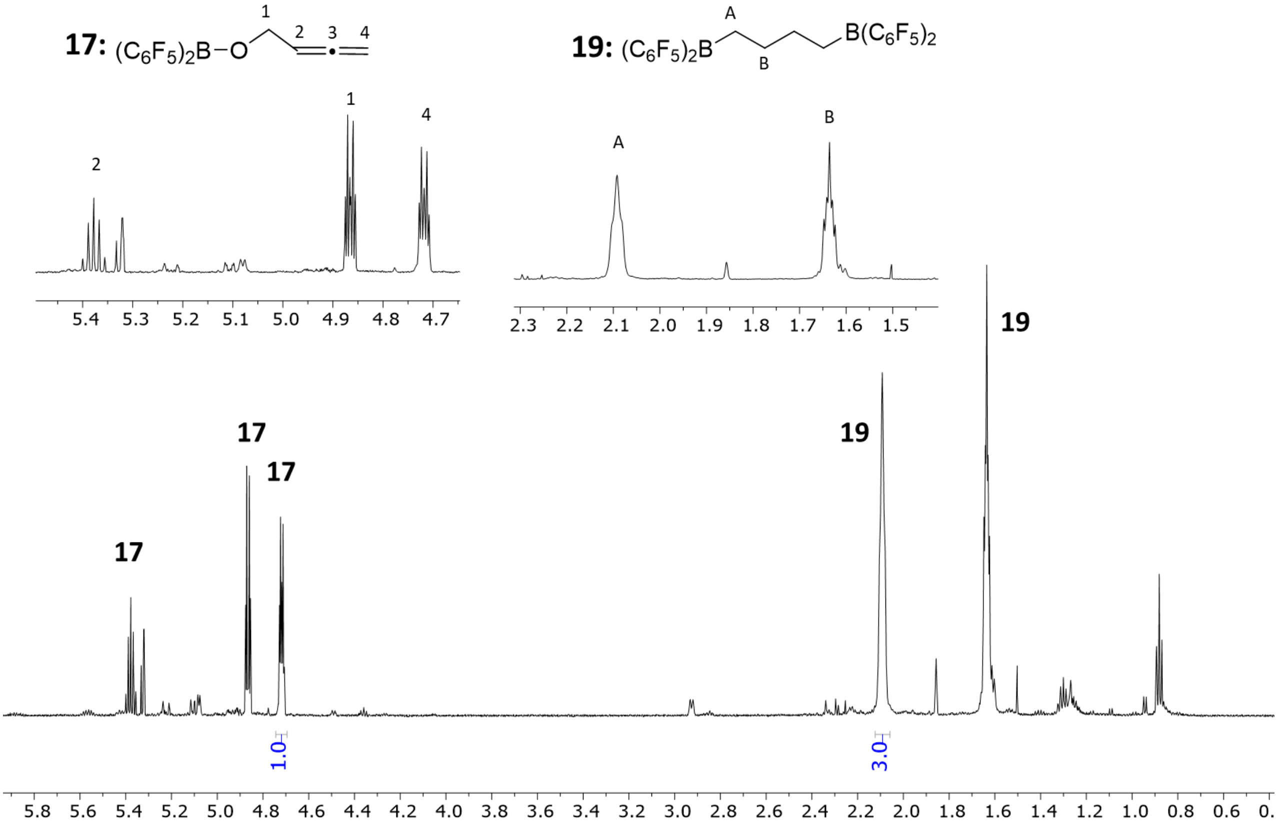

Figure S107. ^{1}H NMR (600 MHz, CD_2Cl_2, 299K) spectrum of the reaction mixture after addition of a third equivalent of $HB(C_6F_5)_2$.

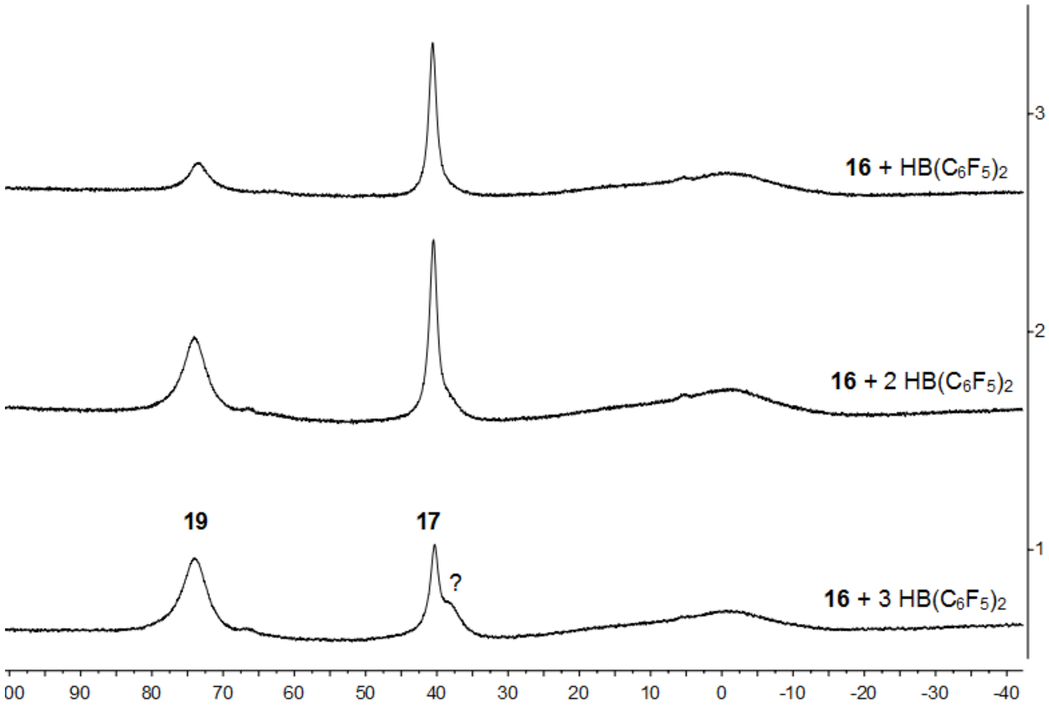

Figure S108. $^{11}B\{^{1}H\}$ NMR (192 MHz, 299 K, CD_2Cl_2) spectra of the reaction mixture after addition of (3) one equivalent of $HB(C_6F_5)_2$, (2) a second equivalent of $HB(C_6F_5)_2$, (1) a third equivalent of $HB(C_6F_5)_2$.[?: tentatively assigned as BOB species by cleavage of compound **17**]

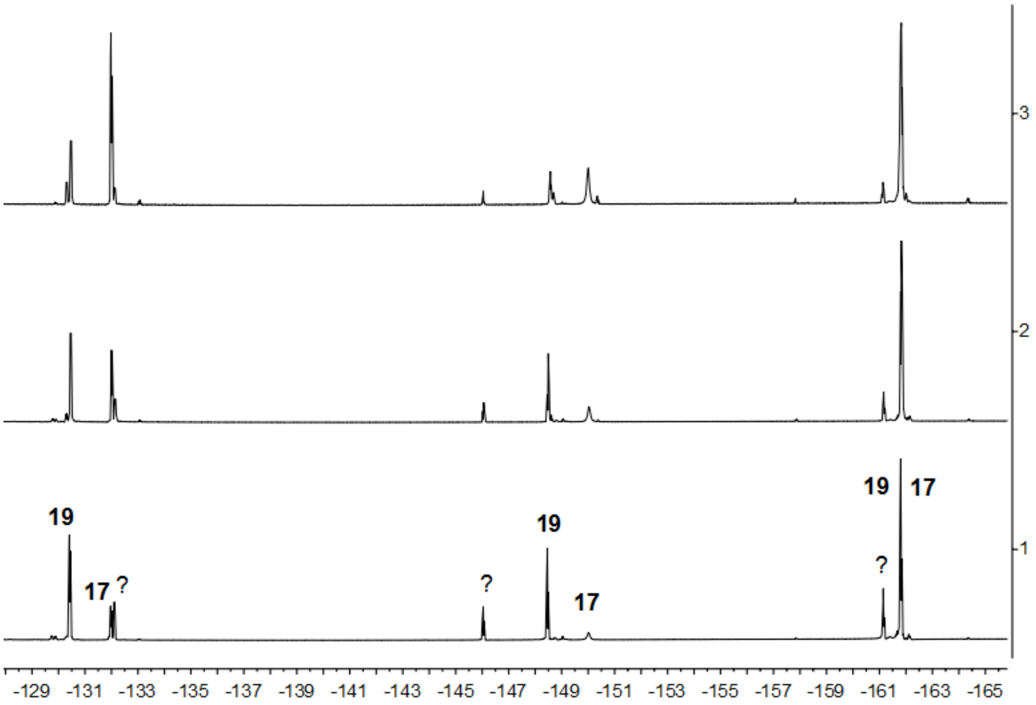

Figure S109. ^{19}F NMR (564 MHz, CD_2Cl_2, 299K) spectra of the reaction mixture after addition of (3) one equivalent of $HB(C_6F_5)_2$, (2) a second equivalent of $HB(C_6F_5)_2$, (1) a third equivalent of $HB(C_6F_5)_2$.[?: tentatively assigned as BOB species by cleavage of compound **17**]
